# The ERA Registry Annual Report 2022: Epidemiology of Kidney Replacement Therapy in Europe, with a focus on sex comparisons

**DOI:** 10.1093/ckj/sfae405

**Published:** 2024-12-12

**Authors:** Rianne Boenink, Marjolein Bonthuis, Brittany A Boerstra, Megan E Astley, Iris R Montez de Sousa, Jaakko Helve, Kirill S Komissarov, Jordi Comas, Danilo Radunovic, Lukas Buchwinkler, Kristine Hommel, Nikola Gjorgjievski, Ana A Galvão, Nicos Mitsides, Maria Marques Vidas, Alicja M Dębska-Ślizień, Csaba Ambrus, Maria F Slon-Roblero, Marc A G J ten Dam, Mathilde Lassalle, Rebecca Guidotti, Inmaculada Marín Sánchez, Viktorija Kuzema, Sara Trujillo Alemán, Edita Ziginskiene, Shalini Santhakumaran, Maria O Valentin, Antonio Sarrión Auñón, Olafur S Indridason, Nurhan Seyahi, Marta Artamendi Larrañaga, Milica Kravljaca, Adrián Okša, Héctor García López, Anders Åsberg, Ivan Rychlik, Mai Ots-Rosenberg, Pazit Beckerman, Vjollca Godanci-Kelmendi, Maria Stendahl, Joe Lakey, Kitty J Jager, Alberto Ortiz, Anneke Kramer, Vianda S Stel

**Affiliations:** ERA Registry, Amsterdam UMC location University of Amsterdam, Department of Medical Informatics, Meibergdreef 9, Amsterdam, The Netherlands; Amsterdam Public Health, Quality of Care and Ageing & Later Life, Amsterdam, The Netherlands; ERA Registry, Amsterdam UMC location University of Amsterdam, Department of Medical Informatics, Meibergdreef 9, Amsterdam, The Netherlands; Amsterdam Public Health, Quality of Care and Methodology, Amsterdam, The Netherlands; ERA Registry, Amsterdam UMC location University of Amsterdam, Department of Medical Informatics, Meibergdreef 9, Amsterdam, The Netherlands; Amsterdam Public Health, Quality of Care and Ageing & Later Life, Amsterdam, The Netherlands; ERA Registry, Amsterdam UMC location University of Amsterdam, Department of Medical Informatics, Meibergdreef 9, Amsterdam, The Netherlands; Amsterdam Public Health, Quality of Care and Methodology, Amsterdam, The Netherlands; ERA Registry, Amsterdam UMC location University of Amsterdam, Department of Medical Informatics, Meibergdreef 9, Amsterdam, The Netherlands; Amsterdam Public Health, Quality of Care and Ageing & Later Life, Amsterdam, The Netherlands; Finnish Registry for Kidney Diseases, Finnish Institute for Health and Welfare, Helsinki, Finland; Abdominal Center Nephrology, University of Helsinki and Helsinki University Hospital, Helsinki, Finland; State Institution “Minsk Scientific and Practical Center for Surgery, Transplantology and Hematology”, Minsk, Belarus; Catalan Renal Registry, Catalan Transplant Organization, Health Department, Generalitat of Catalonia, Barcelona, Spain; Clinic for Nephrology, Clinical Center of Montenegro, Podgorica, Montenegro; Austrian Dialysis and Transplantation Registry, Department of Internal Medicine IV (Nephrology and Hypertension), Medical University Innsbruck, Innsbruck, Austria; Department of Nephrology, Holbaek Hospital, Holbaek, Denmark; University Clinic of Nephrology, Skopje, North Macedonia; Faculty of Medicine Ss, Cyril and Methodius, Skopje, North Macedonia; Portuguese Society of Nephrology, Nephrology Service at the University Hospitals of Coimbra, Coimbra, Portugal; Cyprus Renal Registry, Health Monitoring Unit, Ministry of Health, Nicosia, Cyprus; University of Cyprus, Shacolas Education Centre for Clinical Medicine, Nicosia, Cyprus; Nephrology department, Nicosia General Hospital, State Healthcare Services Organisation, Nicosia, Cyprus; Clinical Nephrology, Puerta de Hierro University Hospital, Majadahonda, Spain; Faculty of Medicine, Autonoma University of Madrid, Madrid, Spain; Medical University of Gdańsk, Department of Nephrology, Transplantology and Internal Diseases, Gdańsk, Poland; St Imre Teaching Hospital, Div Nephrology-Hypertension, Budapest, Hungary; B. Braun Avitum Hungary Zrt, Dialysis Center Budapest, Budapest, Hungary; Hospital Universitario de Navarra, Pamplona, Navarra, Spain; Nefrodata Dutch Renal Registry, Utrecht, the Netherlands; REIN registry (Renal Epidemiology and Information Network), Agence de la Biomédecine, Saint-Denis La Plaine, Saint-Denis, France; Institute of Nephrology, Stadtspital Zürich, Zürich, Switzerland; Murcia Renal Registry, Department of Epidemiology, Murcia Regional Health Council, Murcia, Spain; Department of Nephrology, Pauls Stradins Clinical University Hospital, Riga, Latvia; Department of Internal Diseases, Riga Stradins University, Riga, Latvia; Health Quality Assessment and Information System Service, Dirección General de Programas Asistenciales, Servicio Canario de la Salud, Canary Islands, Spain; Lithuanian Nephrology, Dialysis and Transplantation Association, Lithuania; Nephrology Department, Medical Academy, Lithuanian University of Health Sciences, Kaunas, Lithuania; UK Renal Registry, Bristol, UK; Nephrology Department, Valdecilla Hospital, University of Cantabria, IDIVAL, Santander, Spain; Registry of Renal Patients of the Valencian Community, Dirección General de Salud Pública, Valencia, Spain; Division of Nephrology, Internal Medicine Services, Landspitali University Hospital, Reykjavik, Iceland; Istanbul Üniversity-Cerrahpasa, Cerrahpasa Medical Faculty, Istanbul, Turkey; Registry of La Rioja, Department Nephrology, Hospital San Pedro, Logroño, La Rioja, Spain; Clinic of Nephrology, Clinical Center of Serbia, Belgrade, Serbia; Faculty of Medicine, University of Belgrade, Belgrade, Serbia; Slovak Medical University, Faculty of Medicine, Bratislava, Slovakia; Transplant Autonomic Coordination Department, Health Service of Castilla y León, Castilla y León, Spain; Department of Transplantation Medicine, Oslo University Hospital and Department of Pharmacy, University of Oslo, Oslo, Norway; Department of Medicine, Third Faculty of Medicine, Charles University, and University Hospital Kralovske Vinohrady, Prague, Czechia; Tartu University, Faculty of Medicine, Department of Internal Medicine, Tartu, Estonia; Tartu University Hospital, Department of Internal Medicine, Tartu, Estonia; Institute of Nephrology and Hypertension, Sheba Medical Center, Ramat Gan, Israel; Faculty of Medicine, Tel Aviv University, Tel Aviv, Israel; Nephrology Clinic, University Clinical Centre of Kosovo, Prishtina, Kosovo; Department of Medicine, Jönköping Regional Hospital, Jönköping, Sweden; Scottish Renal Registry, Public Health Scotland, Glasgow, UK; ERA Registry, Amsterdam UMC location University of Amsterdam, Department of Medical Informatics, Meibergdreef 9, Amsterdam, The Netherlands; Amsterdam Public Health, Quality of Care and Ageing & Later Life, Amsterdam, The Netherlands; Department of Nephrology and Hypertension, IIS-Fundacion Jimenez Diaz UAM, Madrid, Spain; RICORS2040, Madrid, Spain; ERA Registry, Amsterdam UMC location University of Amsterdam, Department of Medical Informatics, Meibergdreef 9, Amsterdam, The Netherlands; Amsterdam Public Health, Quality of Care and Ageing & Later Life, Amsterdam, The Netherlands; ERA Registry, Amsterdam UMC location University of Amsterdam, Department of Medical Informatics, Meibergdreef 9, Amsterdam, The Netherlands; Amsterdam Public Health, Quality of Care and Ageing & Later Life, Amsterdam, The Netherlands

**Keywords:** dialysis, ESKD, graft survival, kidney transplantation, patient survival

## Abstract

The European Renal Association (ERA) Registry collects data on kidney replacement therapy (KRT) in patients with end-stage kidney disease (ESKD). This paper summarizes the ERA Registry Annual Report 2022, with a special focus on comparisons by sex. The supplement of this paper contains the complete ERA Registry Annual Report 2022. Data was collected from 53 national and regional KRT registries from 35 countries. Using this data, incidence, and prevalence of KRT, kidney transplantation rates, survival probabilities, and expected remaining lifetimes were calculated. In 2022, 530 million people of the European general population were covered by the ERA Registry. The incidence of KRT was 152 per million population (pmp). In incident patients, 54% were 65 years or older, 64% were male, and the most common primary renal disease (PRD) was diabetes mellitus (22%). At KRT initiation, 83% of patients received haemodialysis, 12% received peritoneal dialysis, and 5% underwent pre-emptive kidney transplantation. On 31 December 2022, the prevalence of KRT was 1074 pmp. In prevalent patients, 48% were 65 years or older, 62% were male, the most common PRD was of miscellaneous origin (18%), 56% of patients received haemodialysis, 5% received peritoneal dialysis, and 39% were living with a functioning graft. In 2022, the kidney transplantation rate was 40 pmp, with most kidneys coming from deceased donors (66%). For patients starting KRT between 2013 to 2017, 5-year survival probability was 52%. Compared with the general population, the expected remaining lifetime was 66% and 68% shorter for males and females, respectively, receiving dialysis, and 46% and 49% shorter for males and females, respectively, living with a functioning graft.

## Graphical Abstract

**Graphical Abstract:**
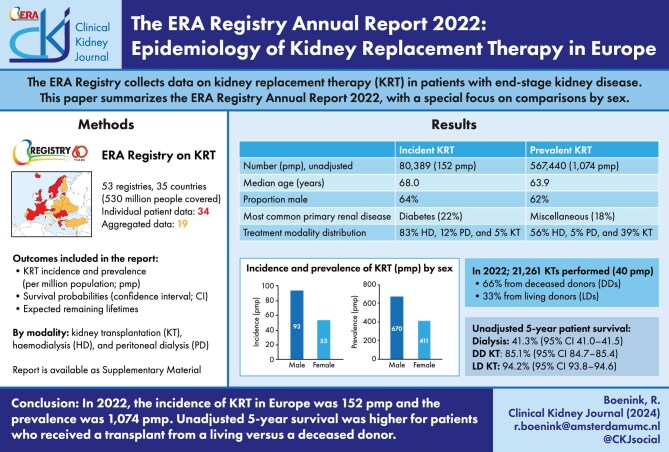

## INTRODUCTION

The European Renal Association (ERA) Registry Annual Report 2022 (Supplementary Data) reports the latest data on the epidemiology of kidney replacement therapy (KRT) for patients with end-stage kidney disease (ESKD) in Europe and countries bordering the Mediterranean Sea. Data were provided by 53 national or regional renal registries from 35 countries, of which 34 registries from 17 countries contributed individual patient data and 19 registries from 19 countries contributed aggregated data (Appendix 1). The participating registries covered ∼530 million people, corresponding to 62.3% of the total European population. This coverage was slightly higher than the 61.9% covered in 2021 [1]. Compared to the previous annual report, data from Hungary were included while data from Albania were not. In this year's annual report, for the first time, tables and figures on incident patients accepted for KRT in 2022 do not only include the distribution of primary renal diseases (PRD) data from the 1995 ERA PRD codes, but also from the updated 2012/2018 ERA PRD codes.

This paper summarizes the 2022 ERA Registry Annual Report, providing an overview of the incidence and prevalence of KRT, the kidney transplantation rates, and the patient and graft survival and expected remaining years of life of patients receiving KRT in Europe. This year's focus is the comparison by sex. The complete ERA Registry Annual Report 2022 can be found in the Supplementary Data, which also includes information on the methodology used.

## RESULTS

### KRT incidence

**Figure 1:**
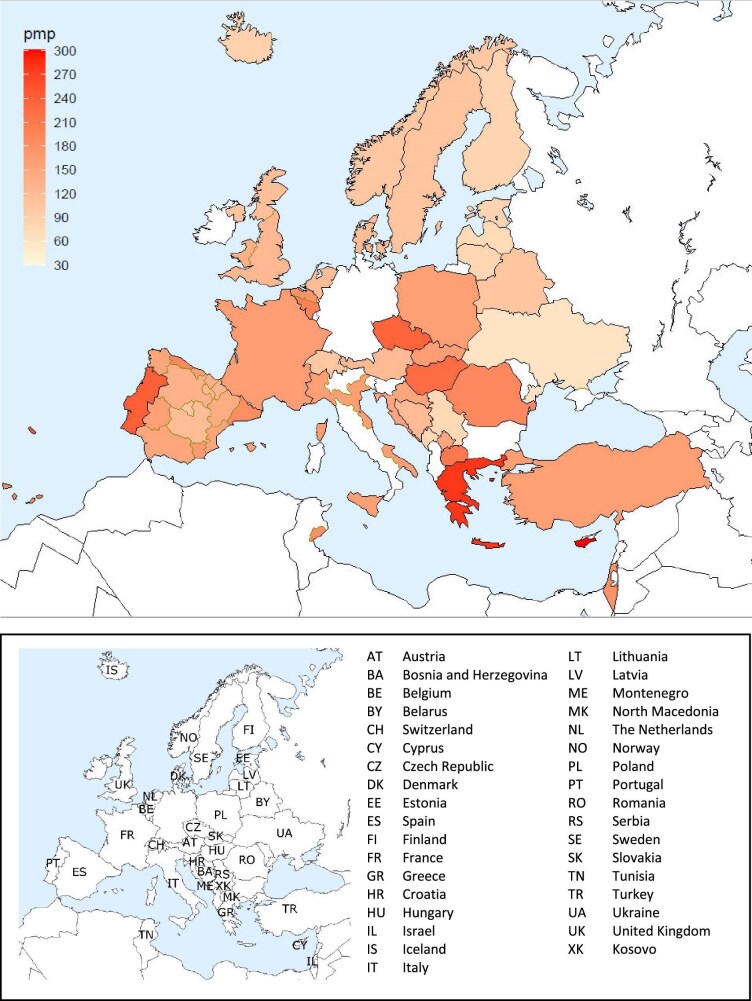
Incidence per million population (pmp) of KRT in 2022 on day 1 by country or region, unadjusted.

**Figure 2:**
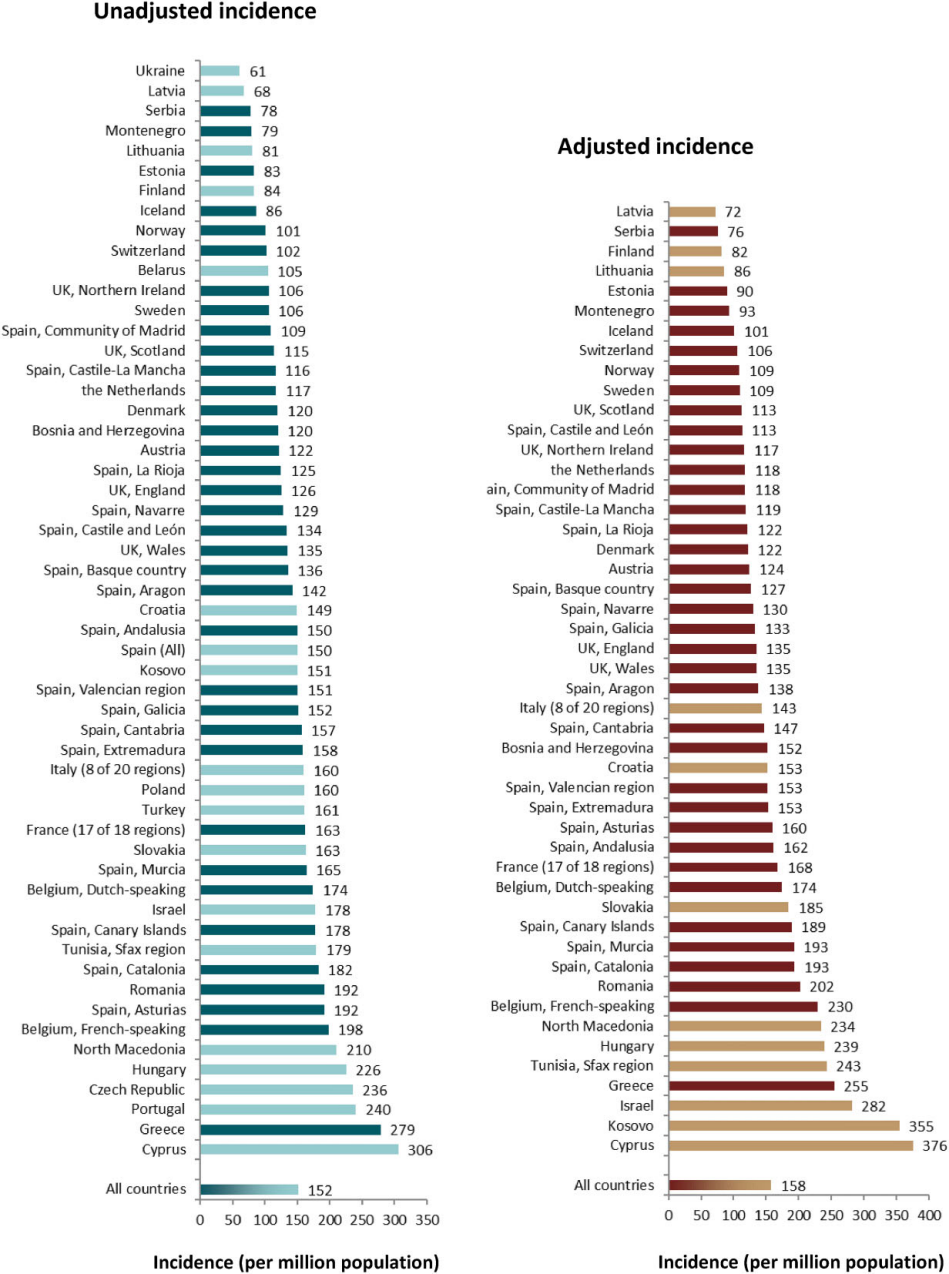
Incidence of KRT per million population in 2022 on day 1 by country or region, unadjusted (left panel), and adjusted (right panel). Registries providing individual patient data are shown as dark bars and registries providing aggregated data as light bars. Adjustment was performed by standardizing the incidence to the age and sex distribution of the EU27 population.

**Figure 3:**
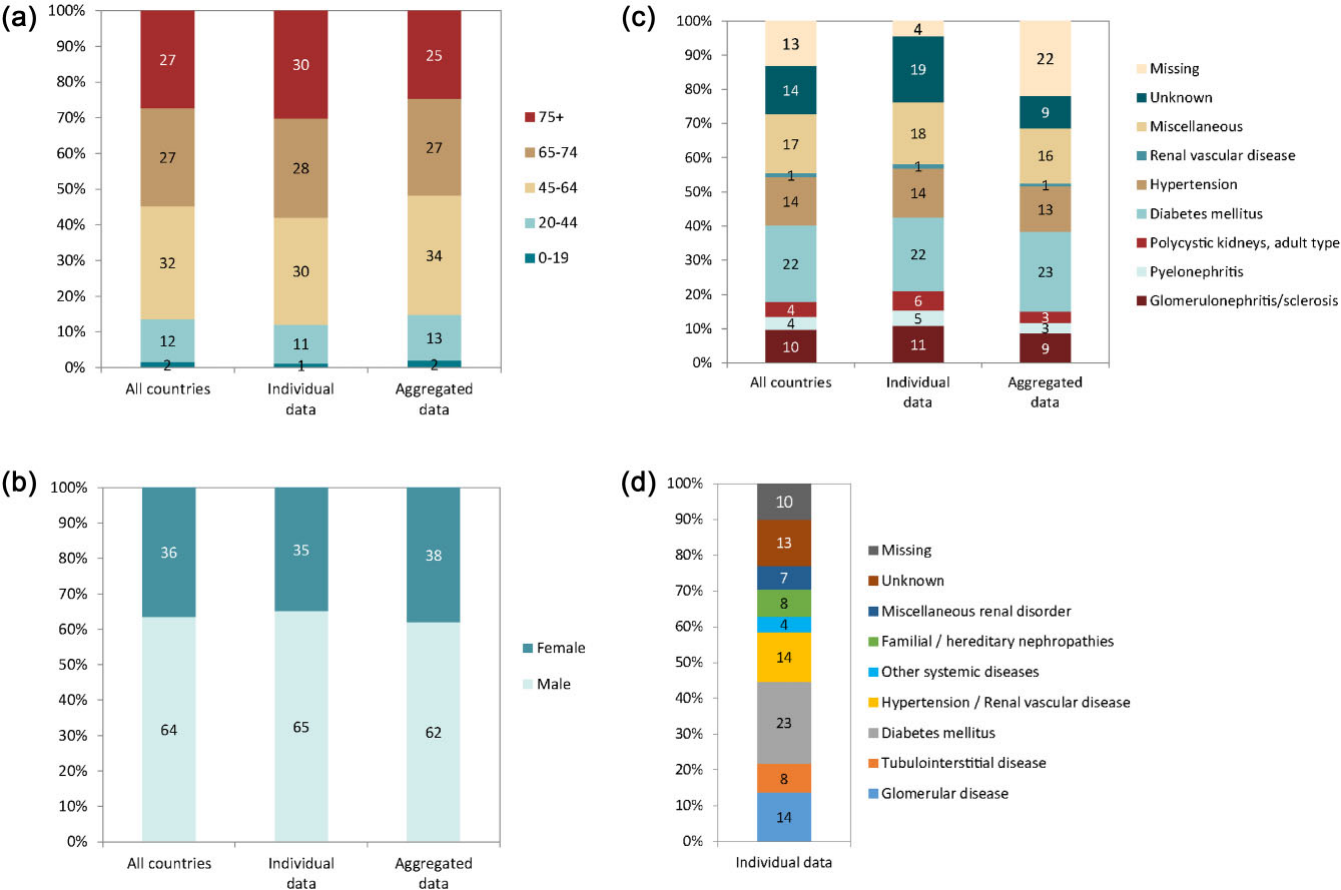
Distribution of (**a**) age, (**b**) sex, (**c**) PRD (1995 ERA codes), and (**d**) PRD (2012/2018 ERA codes), by type of data provided for incident patients accepted for KRT in 2022 on day 1, unadjusted. See Appendix 1 for a list of countries and regions providing individual patient or aggregated data. Panel (d) is only based on the data from registries providing individual patient data. Bars may not add up to 100% due to rounding.

**Figure 4:**
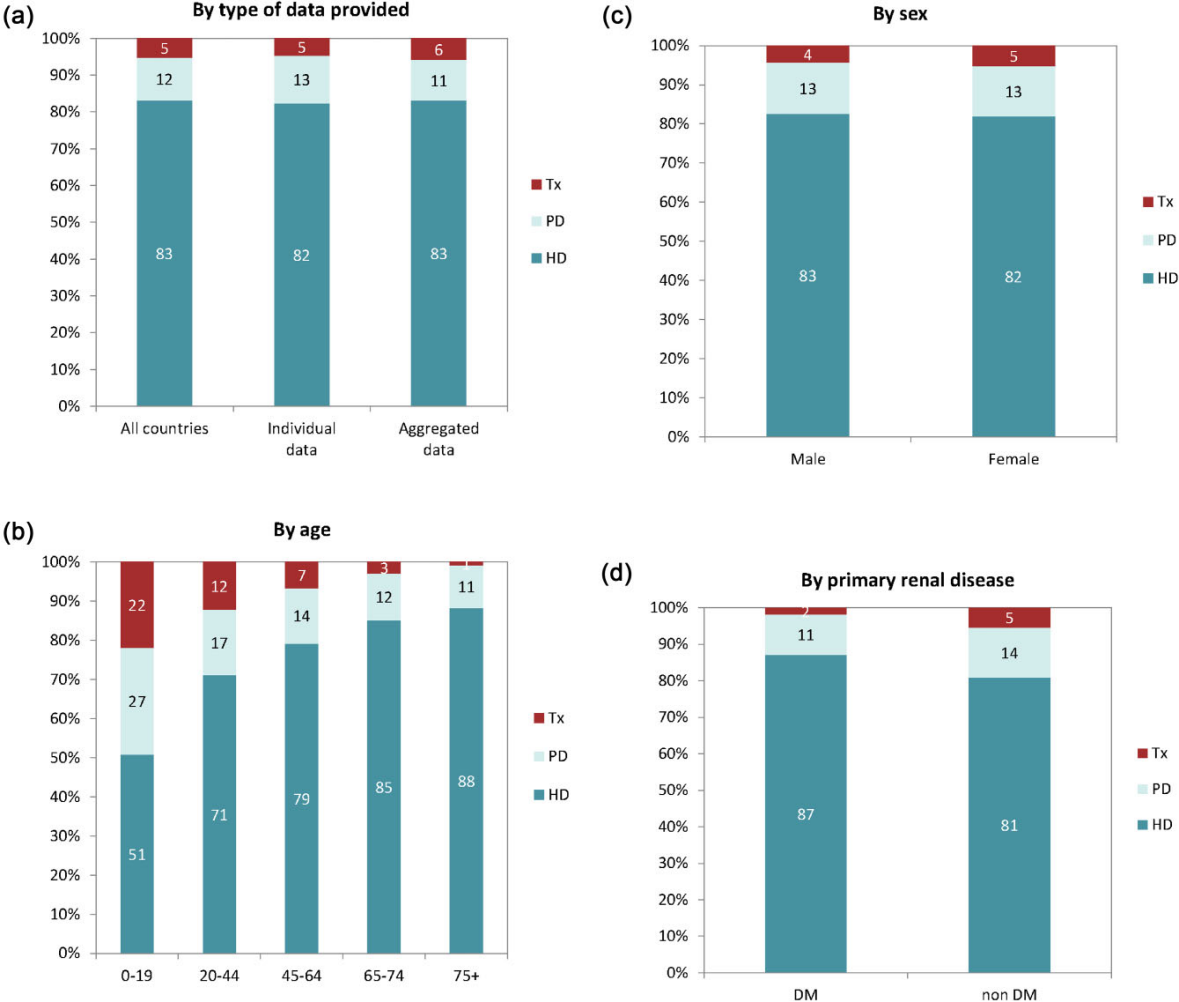
Distribution of treatment modality by (**a**) type of data provided, (**b**) age, (**c**) sex, and (**d**) PRD (DM and non-DM) for incident patients accepted for KRT in 2022 on day 1, unadjusted. Panels (b)–(d) are only based on the data from registries providing individual patient data. See Appendix 1 for a list of countries and regions providing individual patient or aggregated data. Abbreviation: Tx: transplant.

**Figure 5:**
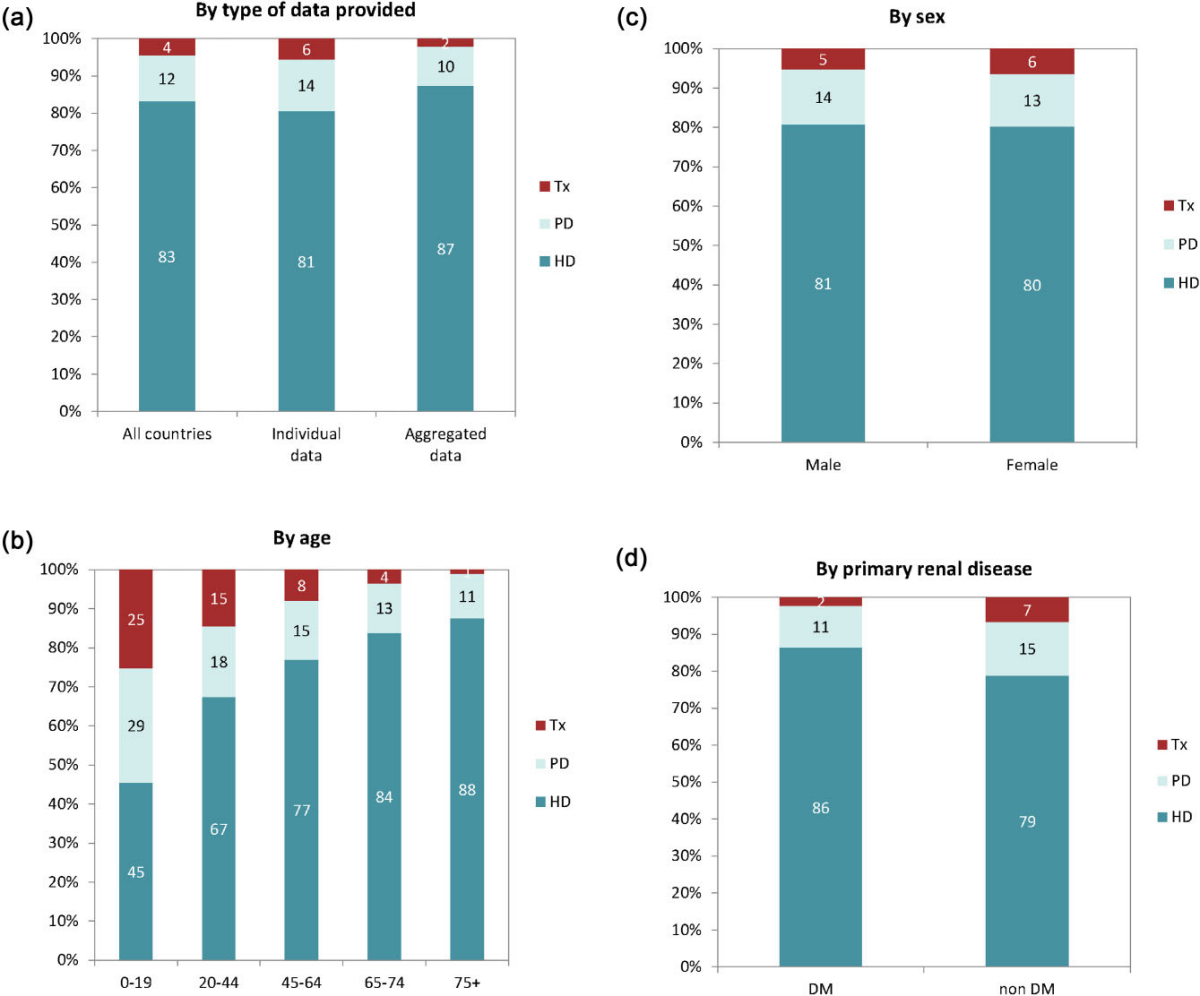
Distribution of treatment modality by (**a**) type of data provided, (**b**) age, (**c**) sex, and (**d**) PRD (DM and non-DM) for incident patients accepted for KRT in 2022 on day 91, unadjusted. Panels (b)–(d) are only based on the data from registries providing individual patient data. See Appendix 1 for a list of countries and regions providing individual patient or aggregated data. Turkey and Spain (aggregated data) did not provide data for day 91. Bars may not add up to 100% due to rounding. Abbreviation: Tx: transplant.

In 2022, 80 389 ESKD patients out of a population of 530 million people initiated KRT (Table 1). This corresponds to an unadjusted KRT incidence rate of 152 per million population (pmp) or 1 in 6600 inhabitants (Table 1), which was higher than the KRT incidence rate of 145 pmp in 2021 [1]. The unadjusted incidence rate ranged from 61 pmp in Ukraine (1 in 16 400 inhabitants) and 68 pmp in Latvia (1 in 14 700 inhabitants) to 279 pmp in Greece (1 in 3600 inhabitants) and 306 pmp in Cyprus (1 in 3300 inhabitants, Table 1 and Figs. 1 and 2). When adjusted for age and sex using the distribution of the European Union 27 countries (EU27) population [2], the differences between countries with the highest and lowest KRT incidence rates hardly changed (Fig. 2). The median age of patients starting KRT was 68.0 years, ranging from 56.0 years in Ukraine to 74.7 years in Greece (Table 1). Among all incident patients, 54% were aged 65 years or older, 64% were male, and the most common PRD was diabetes mellitus (DM) (22%, Fig. 3). At KRT initiation, 83% of patients received HD, 12% received PD, and 5% underwent pre-emptive kidney transplantation, with only minor differences between countries providing individual patient or aggregated data (Fig. 4). For countries providing individual patient data, initial treatment modality varied among age categories, with HD increasing in a step-wise manner from 51% in the age category 0 to 19 years to 88% in the age category 75 years or older (Fig. 4). On the contrary, PD and pre-emptive kidney transplantation decreased with age from 27% and 22% in the age category 0 to 19 years to 11% and 1% in the age category 75 years or older, respectively (Fig. 4). The distribution of initial treatment modalities was similar for males and females (Fig. 4). Patients with DM as PRD received a pre-emptive kidney transplant less frequently compared to patients with other PRDs (2% versus 5%, Fig. 4). On day 91 after the start of KRT, 83% of all incident patients were receiving HD, 12% were receiving PD, and 4% were living with a functioning kidney graft (excluding Turkey, with a high pre-emptive kidney transplantation rate, which did not provide data for day 91; Fig. 5).

**Table 1:** Summary data on the unadjusted incidence of KRT in 2022 on day 1 by country or region, the mean and median age at the start of KRT, and the incidence of KRT in patients with DM as PRD.

		Incidence of KRT in 2022 on day 1					
Country/region	General population covered by the registry in thousands	All (*n*)	All (pmp)	Mean age (years)	Median age (years)	DM (*n*)	DM (pmp)
Austria^a^	8799	1073	122	65.2	67.8	251	29
Belarus^b^	8490	888	105			160	19
Belgium, Dutch-speaking^c^	6749	1177	174	70.4	73.5	242	36
Belgium, French-speaking^c^	4931	978	198	68.4	71.4	189	38
Bosnia and Herzegovina	3531	424	120	63.5	65.9	125	35
Croatia^d^	3162	472	149	71.0	72.0	142	45
Cyprus	905	277	306	69.6	71.0	107	118
Czech Republic^d^	10 611	2502	236				
Denmark	5903	707	120	63.0	65.7	195	33
Estonia	1349	112	83	61.2	62.4	20	15
Finland	5564	465	84	62.1	65.1	143	26
France (17 of 18 regions)	67 614	10 989	163	66.9	70.4	2448	36
Greece	10 437	2907	279	72.0	74.7	660	63
Hungary	9689	2186	226	65.0	68.0	1070	110
Iceland	382	33	86	55.9	61.8	7	18
Israel	9557	1703	178	65.8	69.4	750	78
Italy (8 of 20 regions)	27 261	4362	160	68.7	71.4	577	21
Kosovo^b^	1688	254	151	62.4	66.0	97	57
Latvia	1670	114	68	59.8	62.0	24	14
Lithuania	2806	227	81	62.6	64.3	37	13
Montenegro^c^	617	49	79	63.4	67.4	14	23
North Macedonia	1830	385	210	63.6	66.0	85	46
Norway	5457	553	101	64.2	67.5	93	17
Poland	37 827	6068	160			1523	40
Portugal^e^	10 467	2515	240			731	70
Romania	19 049	3654	192	63.0	65.6	400	21
Serbia	6383	500	78	61.9	65.4	97	15
Slovakia^d^	4362	710	163	62.9	65.0	215	49
Spain (All)	47 475	7136	150	63.8	68.3	1574	33
Spain, Andalusia	8542	1281	150	64.8	68.2	321	38
Spain, Aragon	1343	191	142	64.6	68.2	42	31
Spain, Asturias	1006	193	192	68.6	71.6	47	47
Spain, Basque country	2213	300	136	64.2	66.7	62	28
Spain, Canary Islands	2199	392	178	63.9	66.6	123	56
Spain, Cantabria^c^	587	92	157	66.6	70.4	15	26
Spain, Castile, and León^c^	2373	318	134	68.6	71.4	76	32
Spain, Castile-La Mancha^c^	2069	241	116	66.7	67.6	56	27
Spain, Catalonia	7793	1422	182	66.4	69.7	270	35
Spain, Community of Madrid	6750	702	109	64.1	66.5	139	22
Spain, Extremadura	1055	167	158	65.5	69.0	31	29
Spain, Galicia	2696	409	152	66.3	67.9	93	34
Spain, La Rioja	321	40	125	65.9	66.5	5	16
Spain, Murcia	1532	252	165	65.6	69.6	64	42
Spain, Navarre^c^	668	86	129	62.8	64.8	24	36
Spain, Valencian region	5098	768	151	66.4	69.0	139	27
Sweden	10 487	1115	106	64.6	68.8	270	26
Switzerland	8689	889	102	65.7	69.8	185	21
the Netherlands	16 285	1902	117	62.5	66.0	370	23
Tunisia, Sfax region^d^	1023	184	179	61.9	65.0	56	55
Turkey^f^	85 280	13 725	161			2502	59
Ukraine^b^	20 647	1257	61	54.5	56.0	295	14
UK, England	52 823	6647	126	60.4	63.3	1663	31
UK, Northern Ireland	1911	203	106	60.3	63.2	35	18
UK, Scotland	5448	624	115	59.3	61.7	149	27
UK, Wales	3132	423	135	60.8	63.3	125	40
All countries	530 138	80 389	152	64.8	68.0	17 626	37

When cells are left empty, the data are unavailable and could not be used for the calculation of the summary data

a The incidence is underestimated by ∼2% due to one haemodialysis centre not submitting data

b Patients younger than 18 years of age are not reported

c Patients younger than 20 years of age are not reported

d Data include dialysis patients only

e Data on PRD are available for dialysis patients only (*N* = 2492, 99.1% of total)

f Data on DM are extrapolated from data of 6821 patients (49.7% of total)

### KRT prevalence

**Figure 6:**
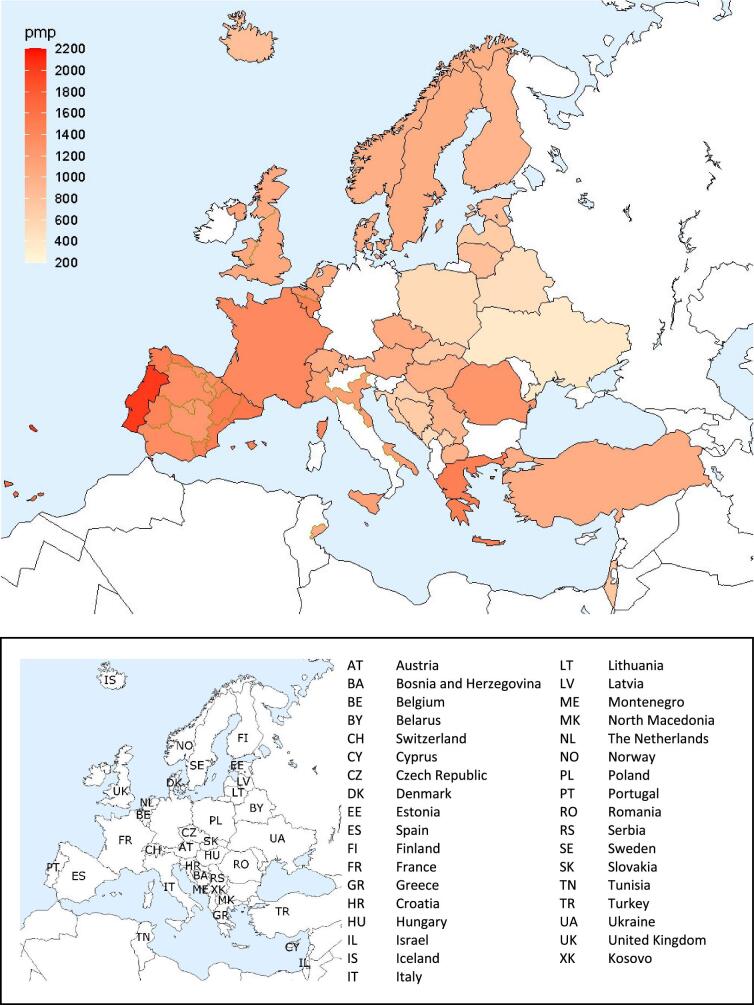
Prevalence per million population (pmp) of KRT on 31 December 2022 by country or region, unadjusted.

**Figure 7:**
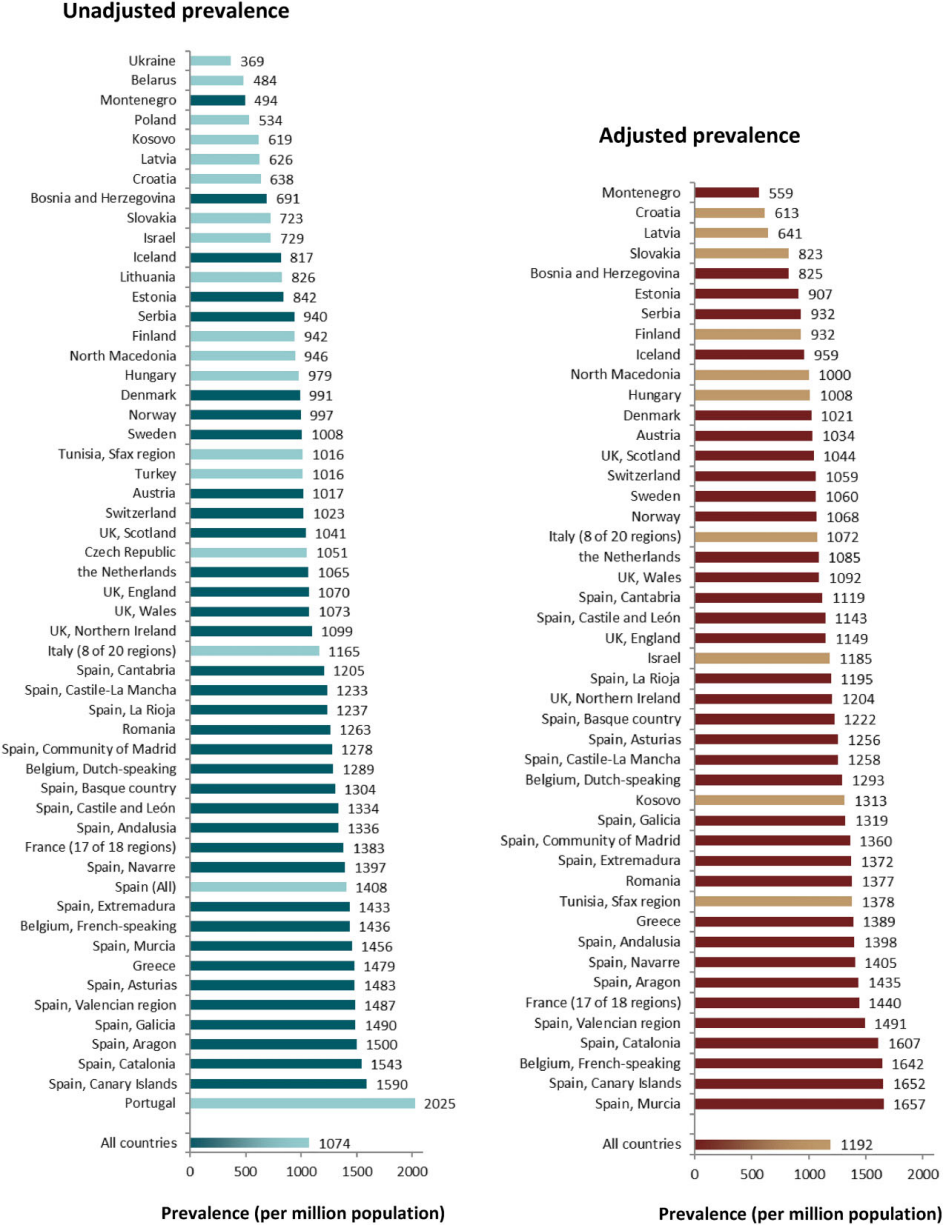
Prevalence per million population of KRT on 31 December 2022 by country or region, unadjusted (left panel) and adjusted (right panel). Registries providing individual patient data are shown as dark bars and registries providing aggregated data as light bars. Adjustment was performed by standardizing the prevalence to the age and sex distribution of the EU27 population.

**Figure 8:**
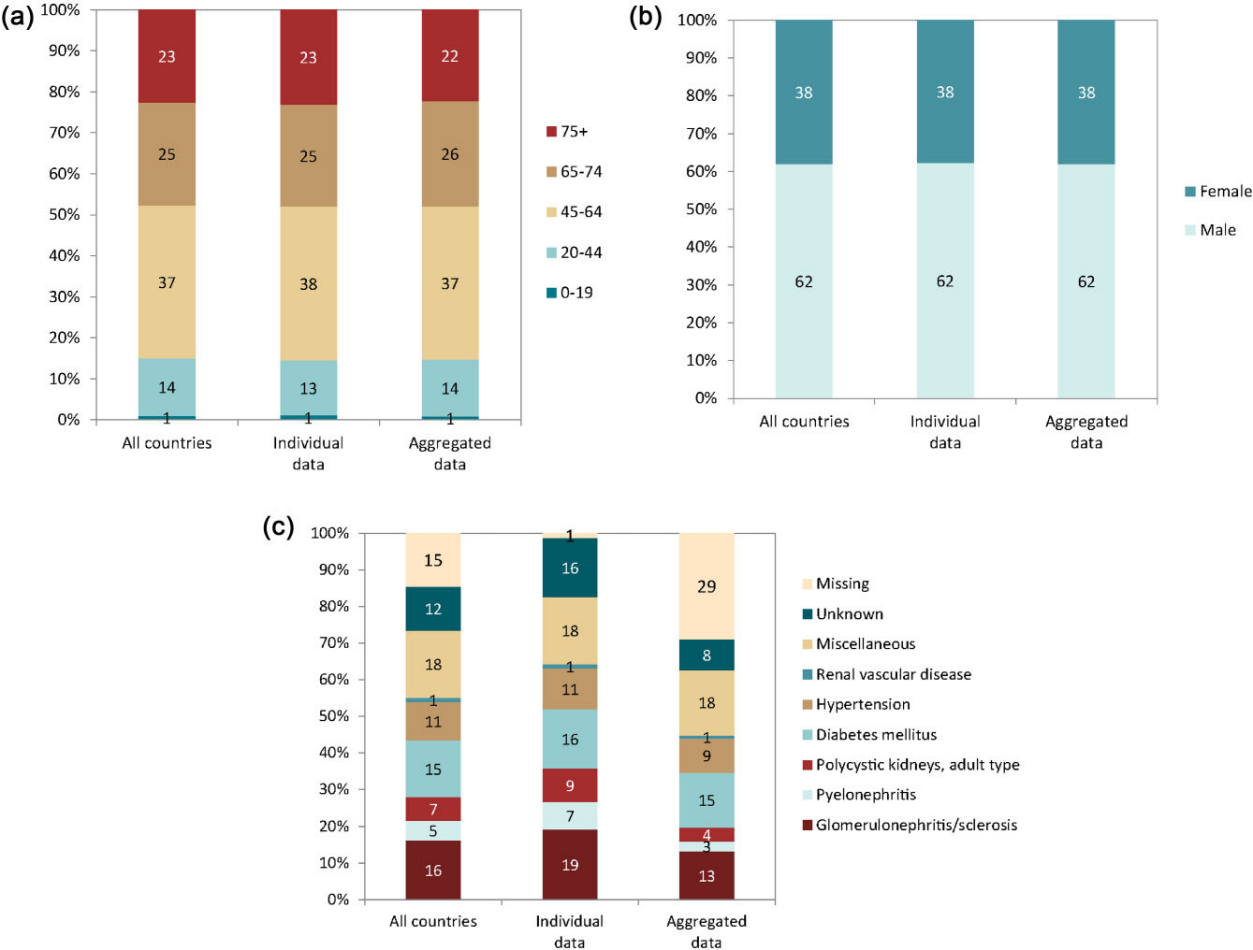
Distribution of (**a**) age, (**b**) sex, and (**c**) PRD (1995 ERA codes) by type of data provided for prevalent patients on KRT on 31 December 2022, unadjusted. See Appendix 1 for a list of countries and regions providing individual patient or aggregated data. Bars may not add up to 100% due to rounding.

**Figure 9:**
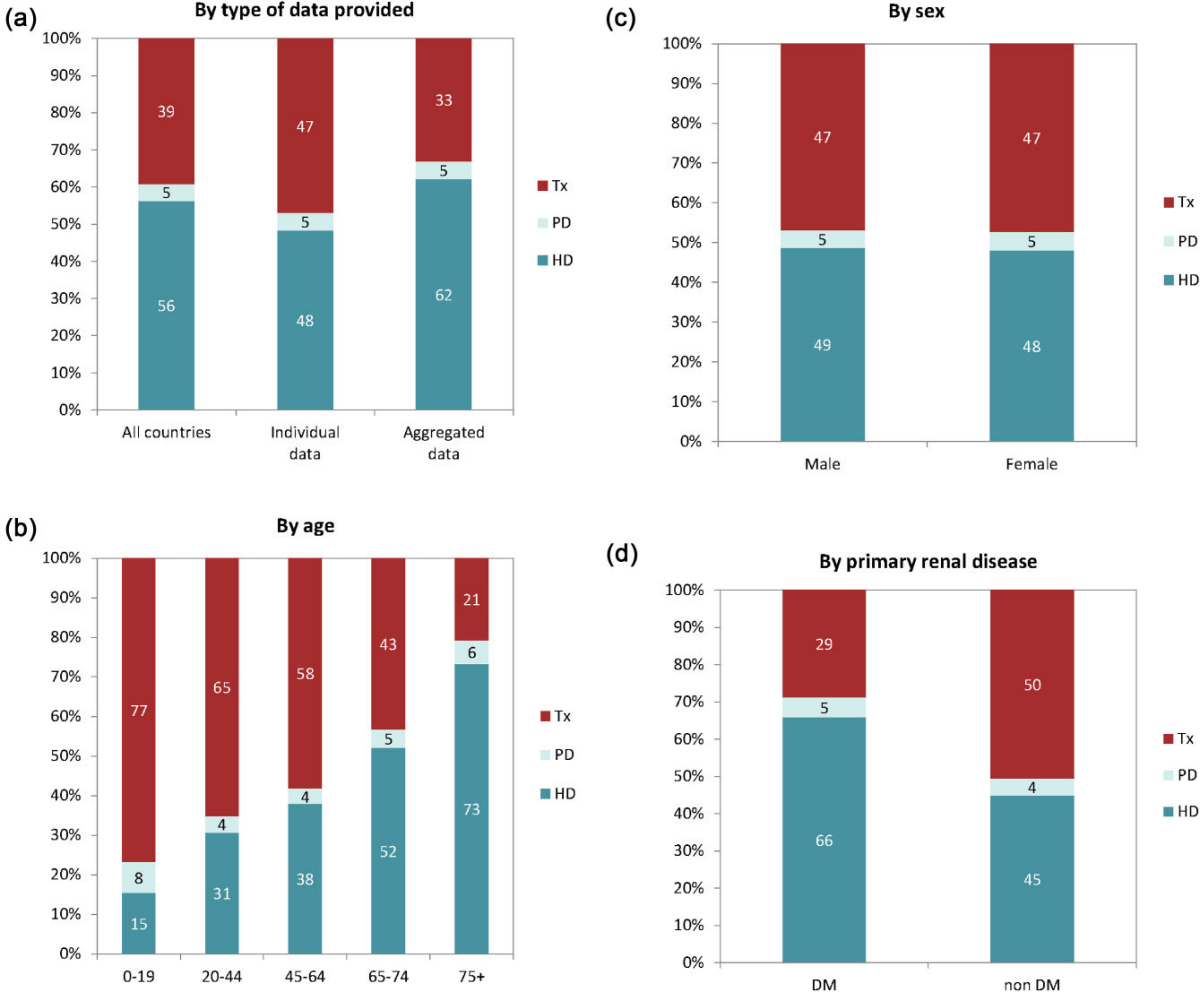
Distribution of treatment modality by (**a**) type of data provided, (**b**) age, (**c**) sex, and (**d**) PRD (DM and non-DM) for prevalent patients on KRT on 31 December 2022, unadjusted. Panels (b)–(d) are only based on the data from registries providing individual patient data. See Appendix 1 for a list of countries and regions providing individual patient or aggregated data. Bars may not add up to 100% due to rounding. Abbreviation: Tx: transplant.

On 31 December 2022, 567 440 patients with ESKD were receiving KRT, corresponding to a prevalence of 1074 pmp or 1 in 930 inhabitants (Table 2). The unadjusted prevalence ranged from 369 pmp in Ukraine (1 in 2700 inhabitants) and 484 pmp in Belarus (1 in 2100 inhabitants) to 1590 pmp in the Canary Islands, Spain (1 in 650 inhabitants) and 2025 pmp in Portugal (1 in 500 inhabitants, Table 2 and Figs. 6 and 7). When adjusted for age and sex using the EU27 distribution [2], these large country differences in KRT prevalence remained (Fig. 7). The median age of prevalent patients was 63.9 years, ranging from 54.0 years in Ukraine to 70.4 years in Israel (Table 2). Among prevalent patients, 48% were aged 65 years or older, 62% were male, and the most common PRD was of miscellaneous origin (18%, Fig. 8). While there was nearly no difference in the age and sex distribution between countries providing individual patient versus aggregated data, the PRD distribution varied by type of data provided, which was likely due to the higher proportion of missing PRDs (29%) for countries providing aggregated data (Fig. 8). Of prevalent patients, 56% received haemodialysis, 5% received peritoneal dialysis, and 39% were living with a functioning graft (Fig. 9). In addition, a larger proportion of patients was living with a functioning graft in countries providing individual data (47%) compared to countries providing aggregated data (33%, Fig. 9). For countries providing individual patient data, the distribution of treatment modalities varied across age groups, with the proportion of patients living with a functioning graft varying from 77% in the age category 0 to 19 years to 21% in patients aged 75 years or older (Fig. 9). For both males and females, 47% of patients were living with a functioning graft (Fig. 9). Only 29% of patients with DM as PRD were living with a functioning graft compared to 50% of patients with a different PRD (Fig. 9).

**Table 2:** Summary data on the unadjusted prevalence of KRT on 31 December 2022 by country or region, the mean and median age on 31 December 2022, and the prevalence of KRT in patients with DM as PRD.

		Prevalent patients on KRT in 2022					
Country/region	General population covered by the registry in thousands	All (*n*)	All (pmp)	Mean age (years)	Median age (years)	DM (*n*)	DM (pmp)
Austria^a^	8799	8950	1017	62.4	63.9	1655	188
Belarus^b^	8490	4108	484			461	54
Belgium, Dutch-speaking^c^	6749	8702	1289	66.6	68.5	1413	209
Belgium, French-speaking^c^	4931	7083	1436	66.2	68.0	1233	250
Bosnia and Herzegovina	3531	2441	691	60.3	62.0	451	128
Croatia^d^	3162	2018	638	66.8	70.0	513	162
Czech Republic	10 611	11 157	1051				
Denmark	5903	5848	991	59.6	60.9	991	168
Estonia	1349	1136	842	60.3	61.6	205	152
Finland	5564	5240	942	60.1	62.6	1256	226
France (17 of 18 regions)	67 614	93 486	1383	63.5	65.7	15 364	227
Greece	10 437	15 440	1479	66.4	68.4	2729	261
Hungary	9689	9485	979	59.4	61.0	1964	203
Iceland	382	312	817	57.5	58.6	41	107
Israel^d^	9557	6963	729	68.0	70.4	3213	336
Italy (8 of 20 regions)	27 261	31 758	1165	63.6	65.6	3329	122
Kosovo^b^	1688	1044	619	59.3	62.0	284	168
Latvia	1670	1046	626	56.1	58.0	123	74
Lithuania	2806	2318	826				
Montenegro^c^	617	305	494	60.7	63.5	57	92
North Macedonia	1830	1731	946	59.8	62.0	309	169
Norway	5457	5442	997	60.4	62.3	708	130
Poland^d^	37 827	20 198	534			4434	117
Portugal^e^	10 467	21 198	2025	67.6		3754	552
Romania	19 049	24 054	1263	64.4	66.4	2189	115
Serbia	6383	6000	940	62.1	64.4	1056	165
Slovakia^d^	4362	3154	723	63.9	66.0	853	196
Spain (All)	47 475	66 856	1408	60.1	63.5	11 102	234
Spain, Andalusia	8542	11 412	1336	62.0	63.4	1936	227
Spain, Aragon	1343	2014	1500	66.0	67.8	357	266
Spain, Asturias	1006	1492	1483	65.1	67.0	267	265
Spain, Basque country	2213	2887	1304	62.5	64.6	399	180
Spain, Canary Islands	2199	3497	1590	63.1	64.1	905	412
Spain, Cantabria^c^	587	707	1205	64.0	65.1	113	193
Spain, Castile, and León^c^	2373	3165	1334	66.2	67.2	518	218
Spain, Castile-La Mancha^c^	2069	2551	1233	64.3	65.0	443	214
Spain, Catalonia	7793	12 027	1543	63.5	65.1	1833	235
Spain, Community of Madrid	6750	8194	1278	62.8	64.2	1392	217
Spain, Extremadura	1055	1512	1433	64.5	65.3	253	240
Spain, Galicia	2696	4017	1490	64.1	65.5	677	251
Spain, La Rioja	321	397	1237	62.9	63.6	52	162
Spain, Murcia	1532	2230	1456	63.2	64.3	369	241
Spain, Navarre^c^	668	933	1397	63.6	65.5	157	235
Spain, Valencian region	5098	7582	1487	64.2	66.0	1112	218
Sweden	10 487	10 573	1008	60.6	62.6	1774	169
Switzerland	8689	8885	1023	63.2	65.3	1278	147
the Netherlands	16 993	18 096	1065	61.3	63.3	2381	140
Tunisia, Sfax region^d^	1023	1043	1016	58.0	60.0	220	214
Turkey^f^	85 280	86 665	1016			6188	359
Ukraine^b^	20 647	7625	369	53.0	54.0	1283	62
UK, England	52 309	55 950	1070	58.3	59.8	10 020	192
UK, Northern Ireland	1911	2099	1099	58.9	60.1	291	152
UK, Scotland	5448	5671	1041	57.7	59.5	935	172
UK, Wales	3132	3360	1073	58.4	59.5	626	200
All countries	529 427	567 440	1074	62.0	63.9	84 683	191

When cells are left empty, the data are unavailable and could not be used for the calculation of the summary data

a The prevalence is underestimated by approximately 2% due to one haemodialysis centre not submitting data

b Patients younger than 18 years of age are not reported

c Patients younger than 20 years of age are not reported

d Data on prevalence include dialysis patients only

e Data on DM are extrapolated from data of 13 759 patients (65.0% of total)

f Data on DM are extrapolated from data of 17 506 patients (20.2% of total)

### Kidney transplantation

**Figure 10:**
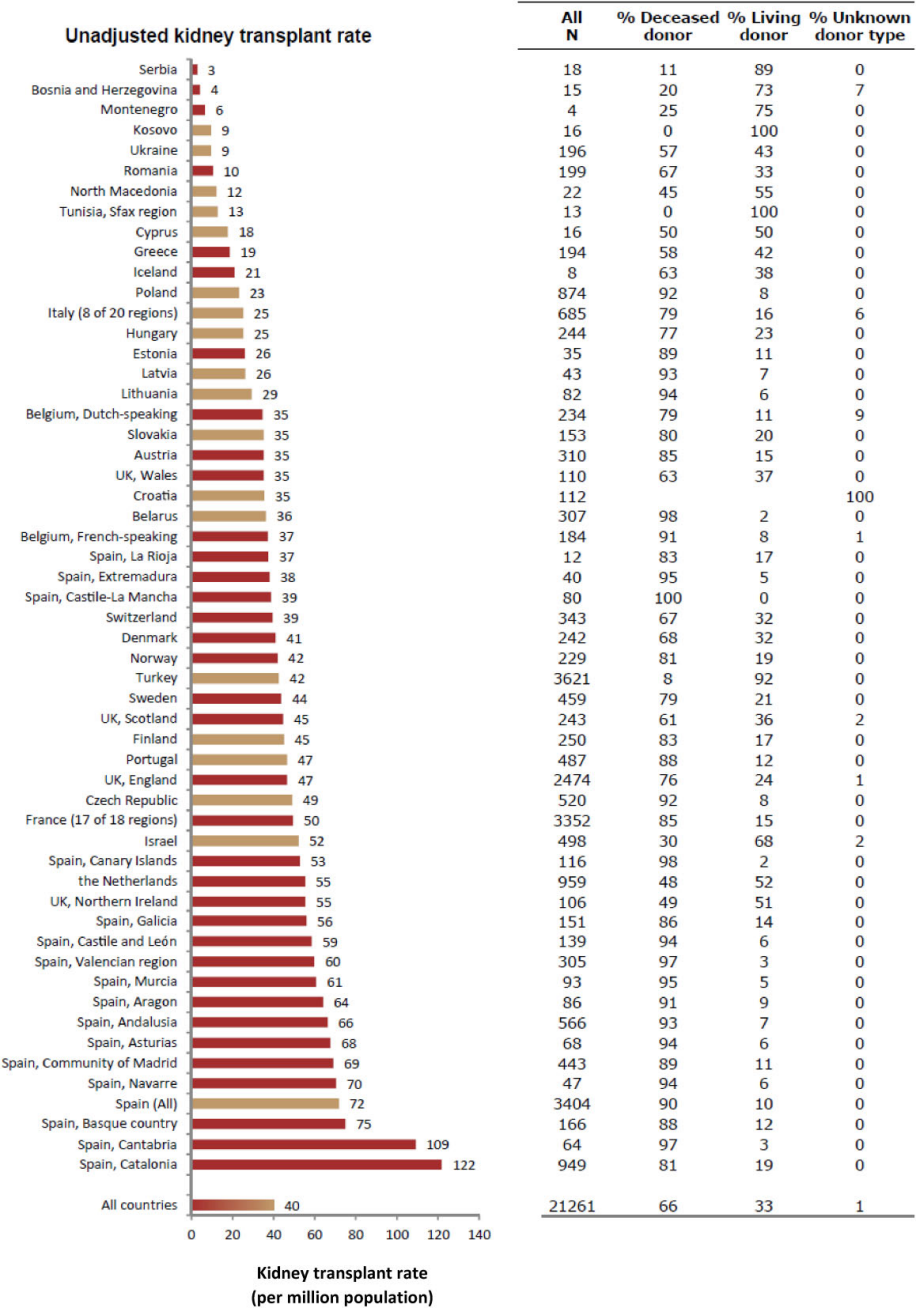
Kidney transplantations performed in 2022 counts (*N*) and per million population by country or region, unadjusted. Registries providing individual patient data are shown as red bars and registries providing aggregated data as orange bars.

**Figure 11:**
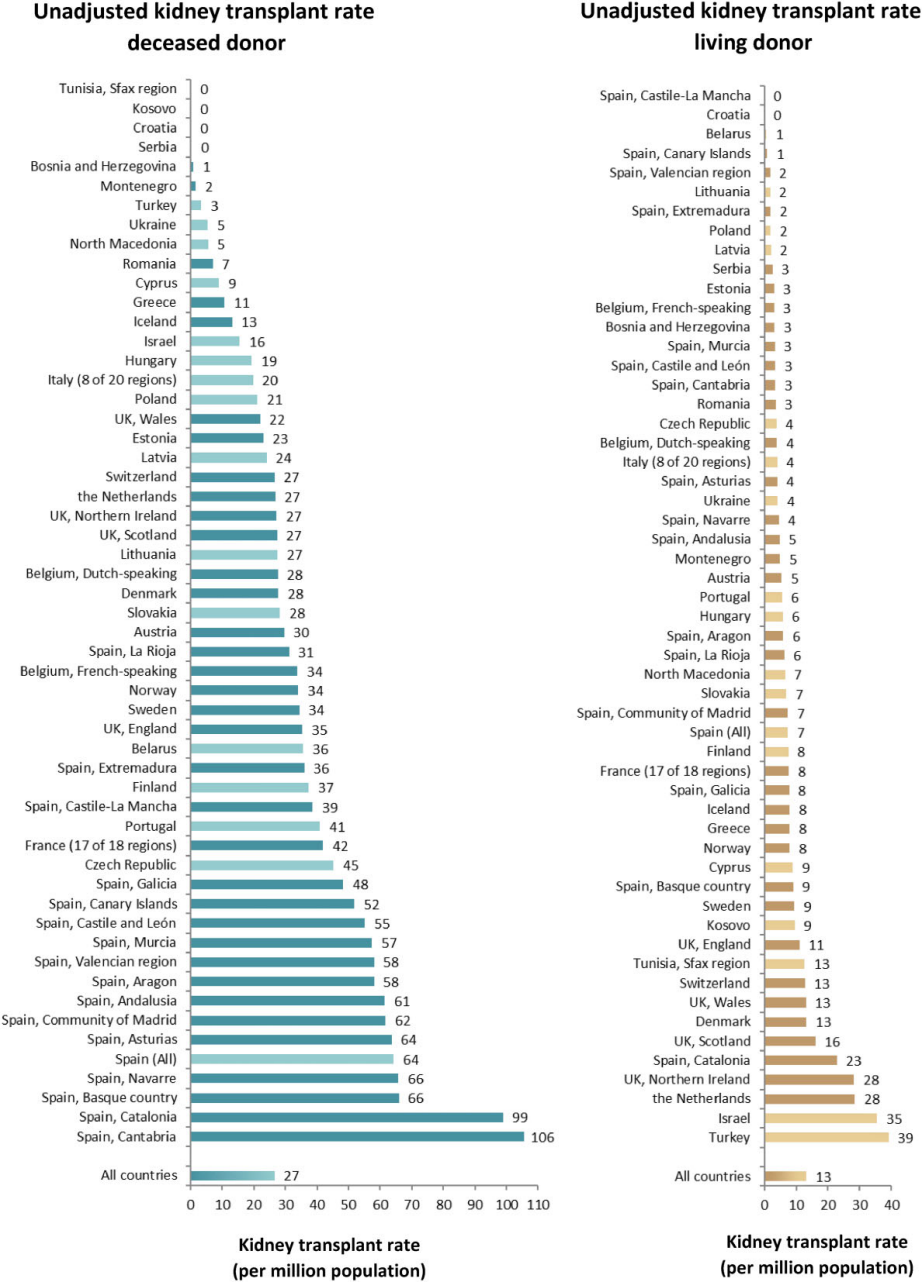
Kidney transplantations performed in 2022 per million population by donor type and by country or region, unadjusted. Registries providing individual patient data are shown as dark bars and registries providing aggregated data as light bars.

**Figure 12:**
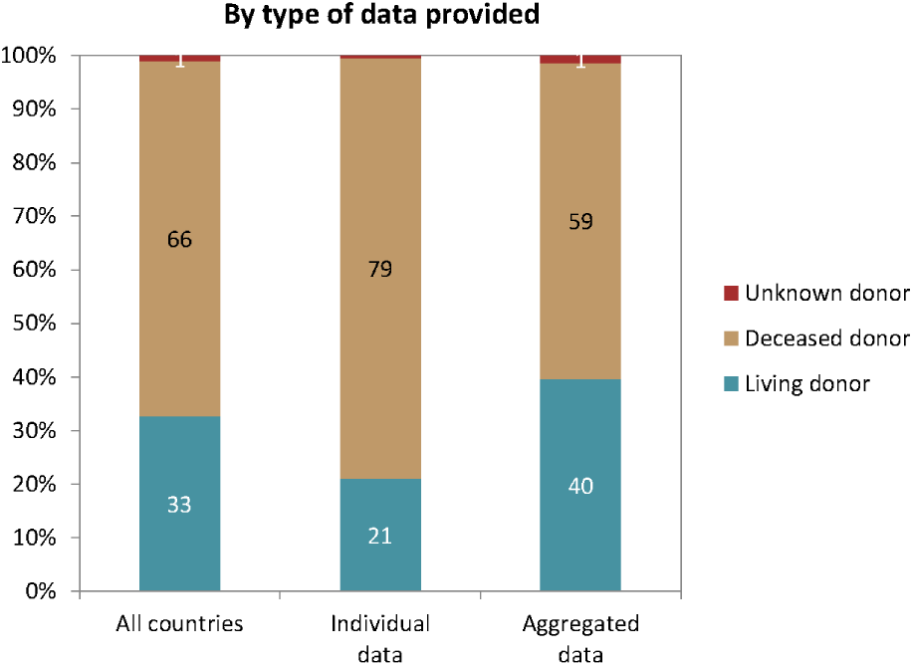
Donor type distribution for kidney transplantations performed in 2022 by type of data provided, unadjusted. See Appendix 1 for a list of countries and regions providing individual patient or aggregated data.

In 2022, 21 261 kidney transplantations were performed, of which 66% were from deceased donors (DD), 33% from living donors (LD), and for 1% the donor type was unknown (Fig. 10). The unadjusted kidney transplantation rate was 40 pmp or 1 in 25 000 inhabitants, ranging from 3 pmp in Serbia (1 in 330 000 inhabitants) to 122 pmp in Catalonia, Spain (1 in 8200 inhabitants, Fig. 10). In the Spanish region Castile-La Mancha, all kidney transplants came from DD while in Kosovo and the Sfax region in Tunisia all kidney transplants came from LD (Fig. 10). The overall unadjusted DD kidney transplantation rate was twice as high as the LD rate (DD: 27 pmp or 1 in 37 000 inhabitants versus LD: 13 pmp or 1 in 76 900 inhabitants, Fig. 11). Cantabria in Spain had the highest rate of DD kidney transplantations (106 pmp or 1 in 9400 inhabitants), while Turkey had the highest rate of LD kidney transplantations (39 pmp or 1 in 25 600 inhabitants, Fig. 11). In countries providing individual patient data, 79% of kidney transplants came from DD compared to 59% in countries providing aggregated data (Fig. 12).

### Survival probability of patients receiving KRT

**Figure 13:**
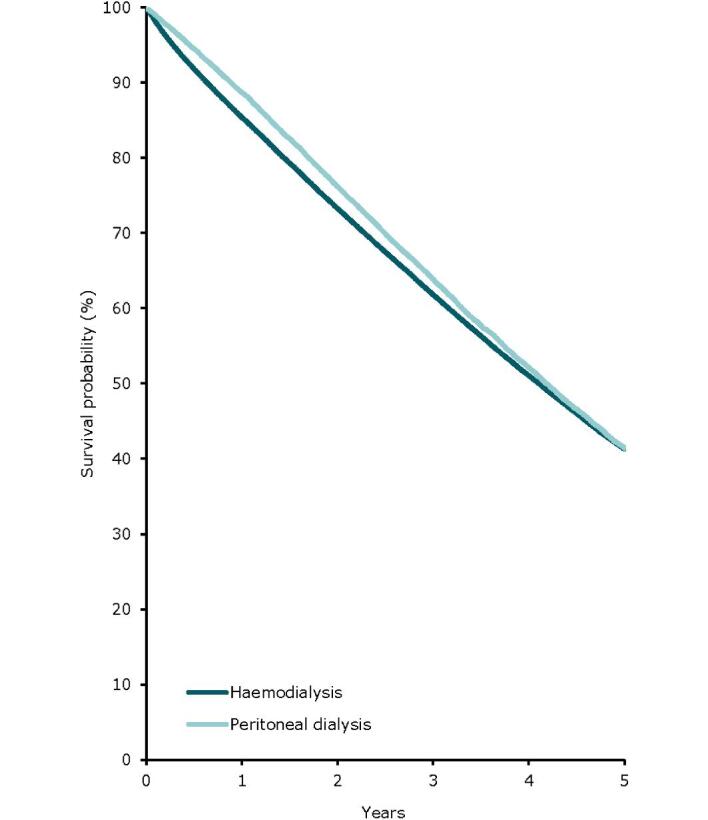
Patient survival by modality (haemodialysis or peritoneal dialysis) for incident dialysis patients accepted for KRT in 2022 on day 91 (cohort 2013–2017), unadjusted. See Appendix 2 for a list of countries and regions providing individual patient data included in the survival analyses.

**Figure 14:**
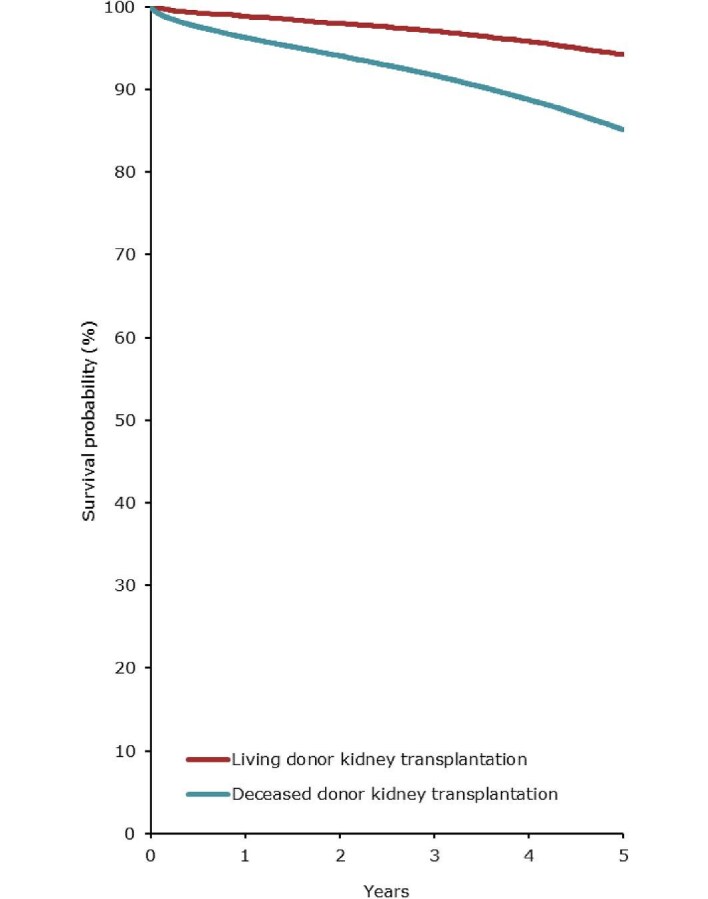
Patient survival in first-time kidney transplant recipients by donor type (deceased or living) from day of transplant (cohort 2013–2017), unadjusted. See Appendix 2 for a list of countries and regions providing individual patient data included in the survival analyses.

For patients initiating KRT between 2013 and 2017, the unadjusted 5-year patient survival probability was 51.5% (95% confidence interval (95% CI) 51.2–51.7, Table 3). In patients initiating dialysis, the unadjusted 5-year survival probability was 41.3% (95% CI 41.0–41.5), while in patients receiving a first kidney transplant, 5-year survival was 85.1% (95% CI 84.7–85.4) for DD and 94.2% (95% CI 93.8–94.6) for LD (Table 3, Figs. 13 and 14). The unadjusted 5-year graft survival probability was 76.2% (95% CI 75.7–76.6) after DD kidney transplantation and 88.1% (95% CI 87.6–88.7) after LD kidney transplantation (Table 3). Similar trends were observed when analyses were adjusted using fixed values for age, sex, and PRD (Table 3).

**Table 3:** One-, two-, and five-year survival probabilities by treatment modality and cohort from day 1 of the start of KRT, dialysis, or from the day of kidney transplantation.

	Survival probabilities as percentages (%) (95% confidence intervals)					
	Cohort: 2013–2017				Cohort: 2016–2020	
Survival type	1 year	2 year	5 year		1 year	2 year
Patient survival on KRT						
Unadjusted	85.3 (85.1–85.5)	75.3 (75.1–75.5)	51.5 (51.2–51.7)		85.7 (85.6–85.9)	75.7 (75.5–75.9)
Adjusted^a^	88.2 (88.0–88.3)	79.3 (79.1–79.5)	54.2 (53.9–54.4)		88.3 (88.2–88.5)	79.2 (79.0–79.4)
Patient survival on dialysis						
Unadjusted	84.2 (84.0–84.4)	72.6 (72.4–72.8)	41.3 (41.0–41.5)		84.7 (84.5–84.9)	73.1 (72.9–73.3)
Adjusted^a^	86.5 (86.4–86.7)	76.3 (76.0–76.5)	46.7 (46.4–47.0)		87.1 (87.0–87.3)	76.9 (76.7–77.1)
Patient survival after a first kidney transplantation (deceased donor)						
Unadjusted	96.3 (96.1–96.5)	94.1 (93.8–94.3)	85.1 (84.7–85.4)		96.1 (95.9–96.3)	93.3 (93.1–93.5)
Adjusted^b^	98.1 (98.0–98.2)	97.0 (96.8–97.1)	91.9 (91.6–92.2)		98.1 (98.0–98.3)	96.8 (96.6–96.9)
Graft survival after a first kidney transplantation (deceased donor)						
Unadjusted	91.0 (90.8–91.3)	87.8 (87.4–88.1)	76.2 (75.7–76.6)		91.1 (90.8–91.3)	87.3 (87.0–87.7)
Adjusted^b^	93.1 (92.9–93.4)	90.5 (90.2–90.8)	80.9 (80.5–81.3)		93.4 (93.2–93.7)	90.6 (90.3–90.9)
Patient survival after a first kidney transplantation (living donor)						
Unadjusted	98.8 (98.6–99.0)	98.0 (97.7–98.2)	94.2 (93.8–94.6)		98.8 (98.6–99.0)	97.9 (97.6–98.1)
Adjusted^b^	99.1 (98.9–99.2)	98.4 (98.2–98.7)	95.3 (95.0–95.7)		99.1 (99.0–99.3)	98.4 (98.2–98.6)
Graft survival after a first kidney transplantation (living donor)						
Unadjusted	96.5 (96.2–96.8)	95.0 (94.6–95.3)	88.1 (87.6–88.7)		96.7 (96.4–97.0)	95.1 (94.7–95.4)
Adjusted^b^	96.4 (96.1–96.8)	94.8 (94.4–95.2)	87.7 (87.1–88.3)		96.6 (96.2–96.9)	94.9 (94.5–95.3)

a Analyses were adjusted using fixed values: age (67 years), sex (63% male), and PRD (24% DM, 19% hypertension/renal vascular disease, 11% glomerulonephritis, and 46% other causes)

b Analyses were adjusted using fixed values: age (50 years), sex (63% male), and PRD (14% DM, 10% hypertension/renal vascular disease, 23% glomerulonephritis, and 53% other causes)

See Appendix 2 for a list of countries and regions providing individual patient data that were included in the survival analyses.

### Expected remaining lifetime

**Figure 15:**
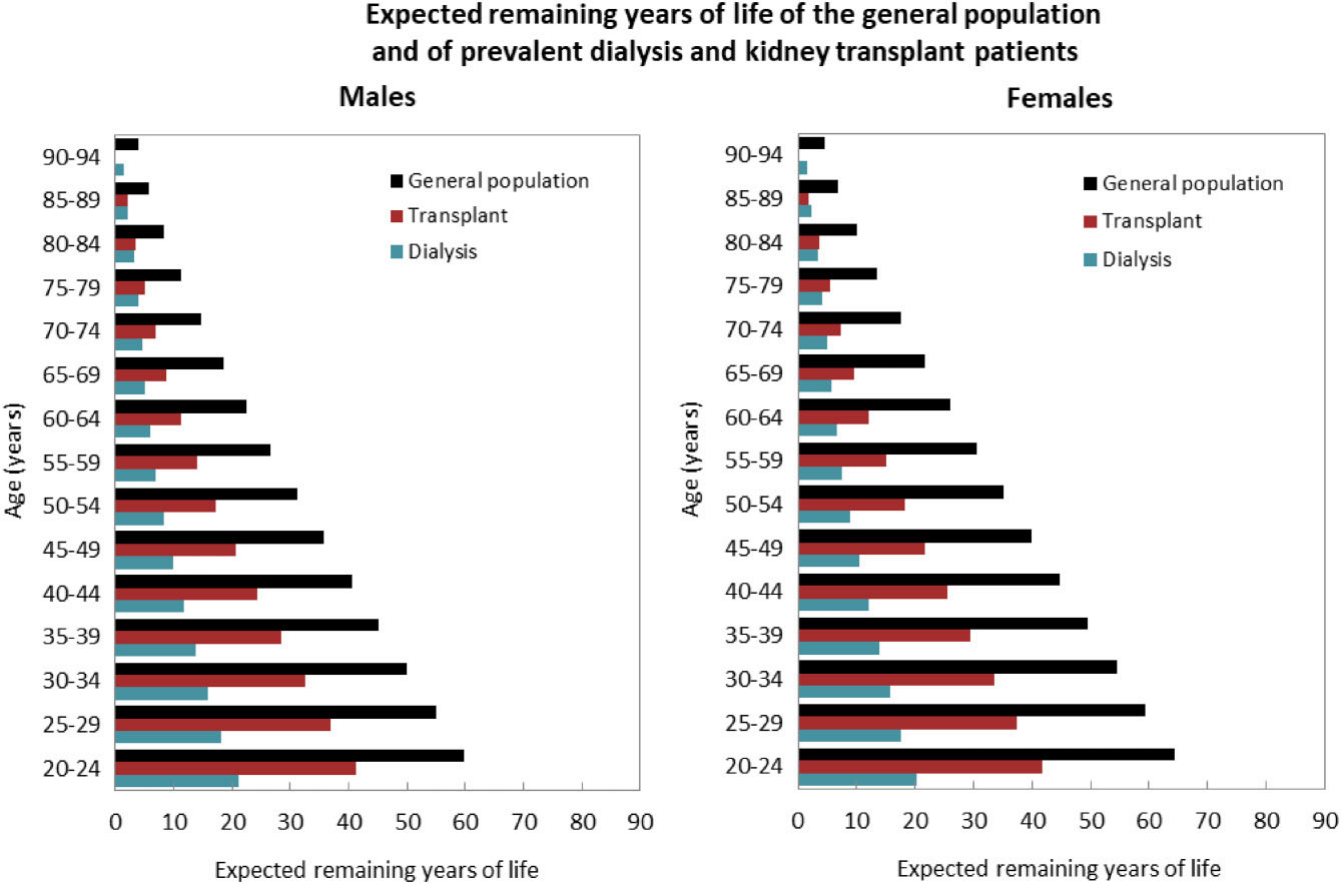
Expected remaining years of life in the general population and for prevalent dialysis and kidney transplant patients (cohort 2018–2022), by sex. See Appendix 2 for a list of countries and regions providing individual patient data included in the expected remaining years of life analyses.

When compared to the adult general population, the expected remaining lifetimes were on average 66% and 68% shorter for males and females on dialysis, and 46% and 49% shorter for males and females living with a functioning graft in the period from 2018 to 2022 (Fig. 15). In males and females aged 20 to 24 years receiving dialysis, the expected remaining lifetimes were 21 and 20 years, respectively, 39 and 44 years shorter than for the general population. For male and female kidney transplant recipients of the same age, expected remaining lifetimes were 41 and 42 years, which were 18 and 23 years shorter than that of the general population. In males and females aged 65 to 69 years receiving dialysis, the expected remaining lifetimes were 5 and 6 years, respectively, 13 and 16 years shorter than in the general population. In male and female kidney transplant recipients of the same age, the expected remaining lifetimes were 9 and 10 years, which were 10 and 12 years shorter than that of the general population.

### Comparisons by sex

**Figure 16:**
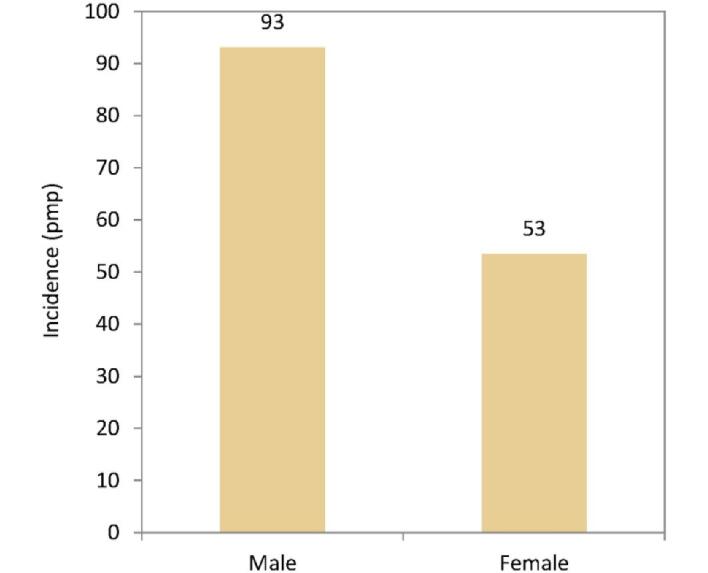
Incidence of KRT per million population (pmp) in 2022 on day 1 by sex, unadjusted.

**Figure 17:**
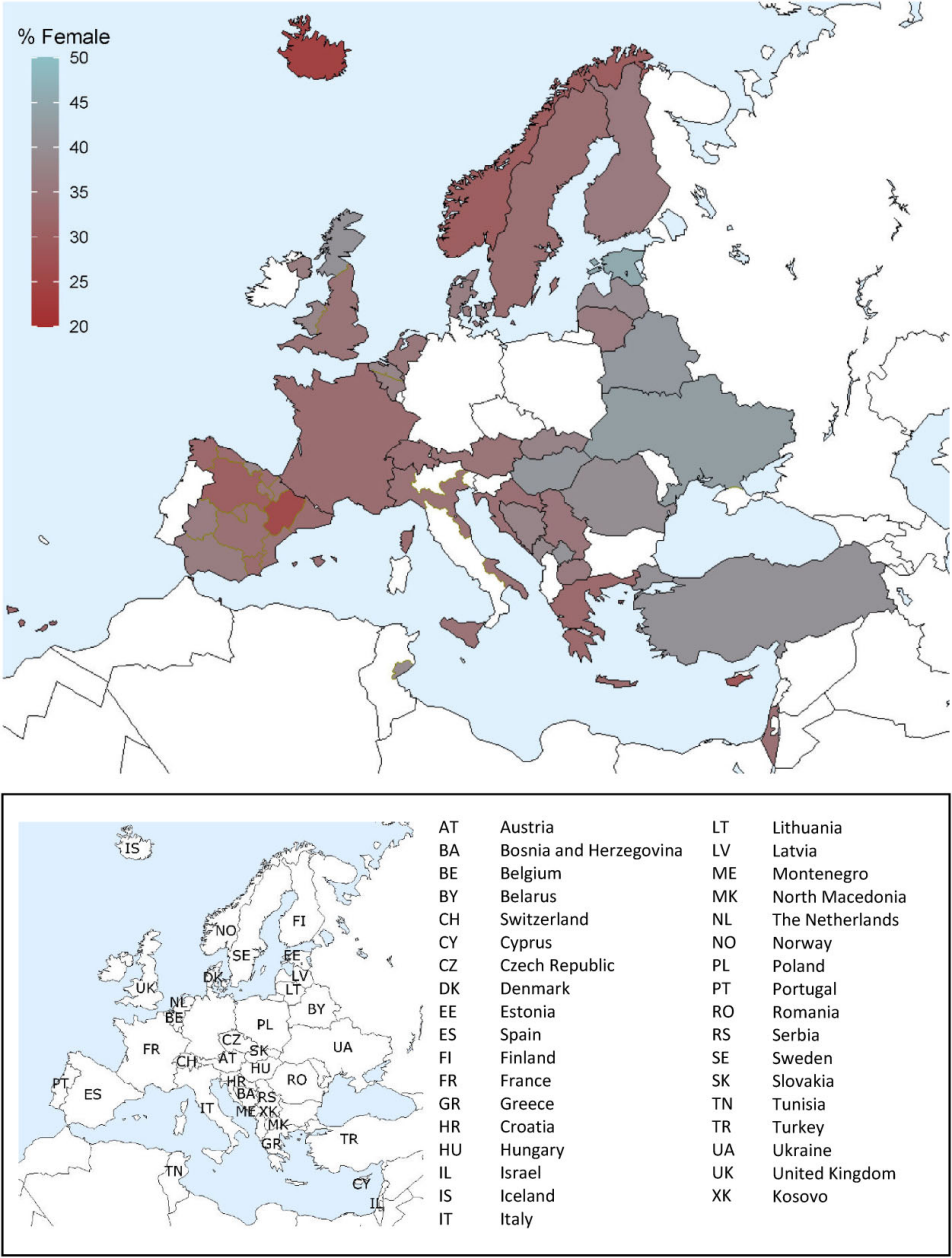
Percentage of female incident patients accepted for KRT in 2022 on day 1 by country or region, unadjusted.

**Figure 18:**
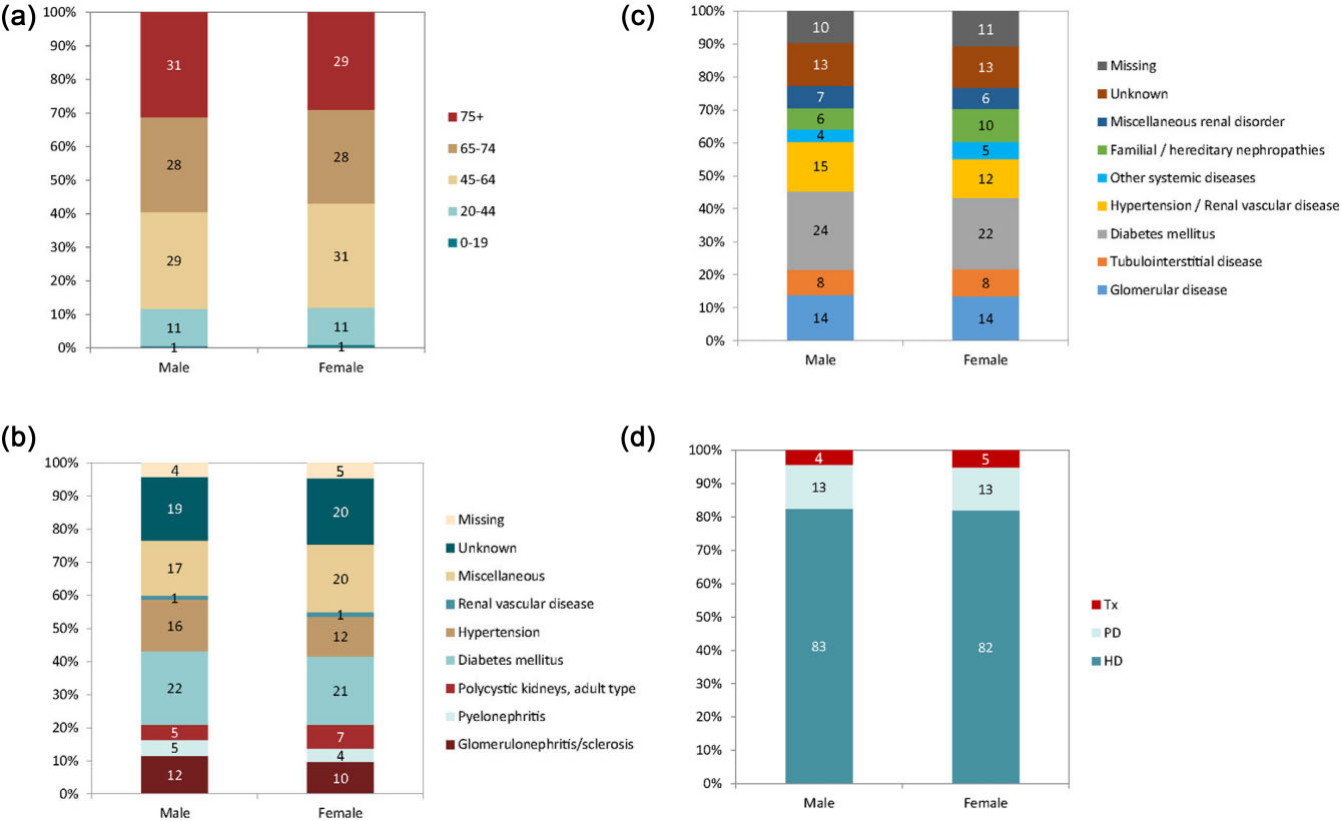
Distribution of (**a**) age, (**b**) PRD (1995 ERA codes), and (**c**) PRD (2012/2018 ERA codes), and (**d**) treatment modality by sex for incident patients accepted for KRT in 2022 on day 1, unadjusted. This figure is only based on data from registries providing individual patient data (see Appendix 1). Bars may not add up to 100% due to rounding. Abbreviation: Tx: transplantation.

In this year's annual report, additional comparisons by sex are presented. In 2022, the KRT incidence was higher in males (93 pmp or 1 in 10 800 inhabitants) compared to females (53 pmp or 1 in 18 900 inhabitants, Fig. 16). The proportion of females among incident KRT patients ranged from 24% in Iceland to 46% in Estonia (Fig. 17). In countries providing individual patient data, the distribution of age and initial treatment modality was similar for males and females (Fig. 18). Although there were no major differences in the PRD distribution among males and females starting KRT, according to the 1995 ERA PRD codes, a higher proportion of males (16%) had hypertension as PRD compared to females (12%, Fig. 18). Findings from the updated ERA PRD codes from 2012 / 2018 showed that females (10%) had a higher proportion of familiar/hereditary nephropathies compared to males (6%, Fig. 18).

**Figure 19:**
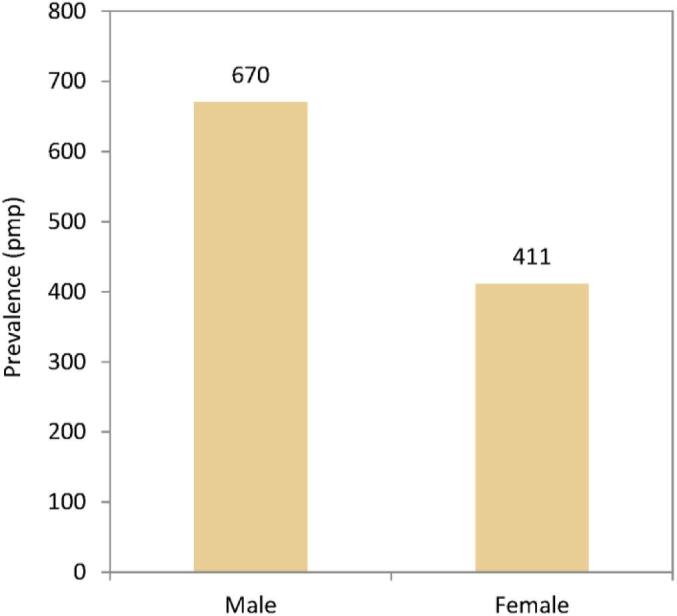
Prevalence of KRT per million population (pmp) on 31 December 2022 by sex, unadjusted.

**Figure 20:**
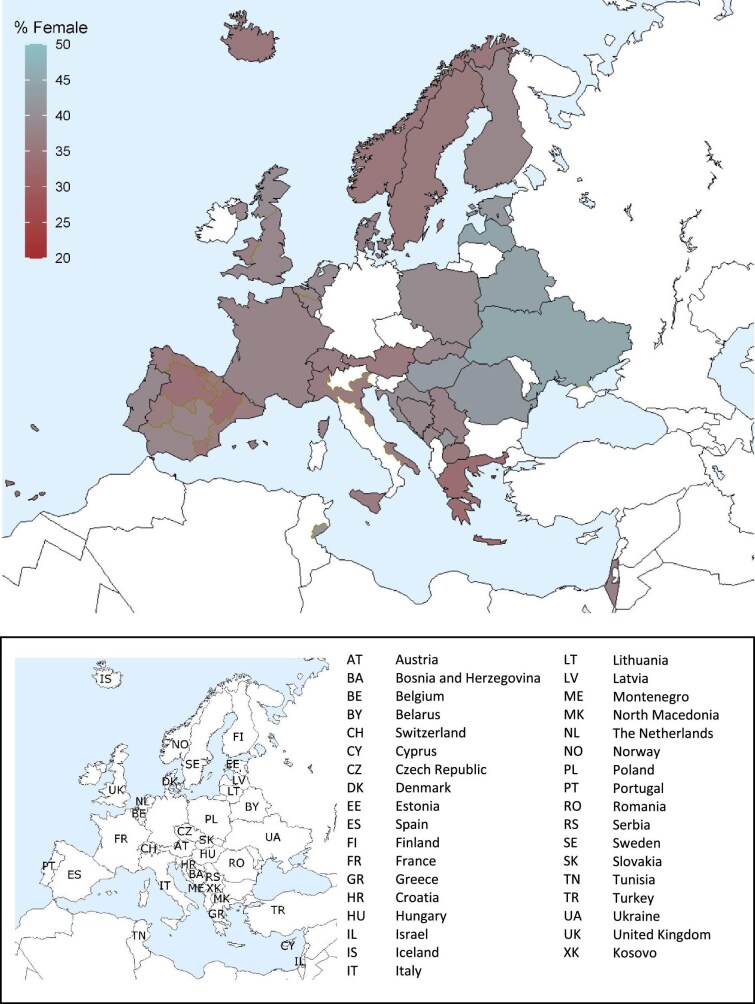
Percentage of female prevalent patients on KRT on 31 December 2022, unadjusted.

**Figure 21:**
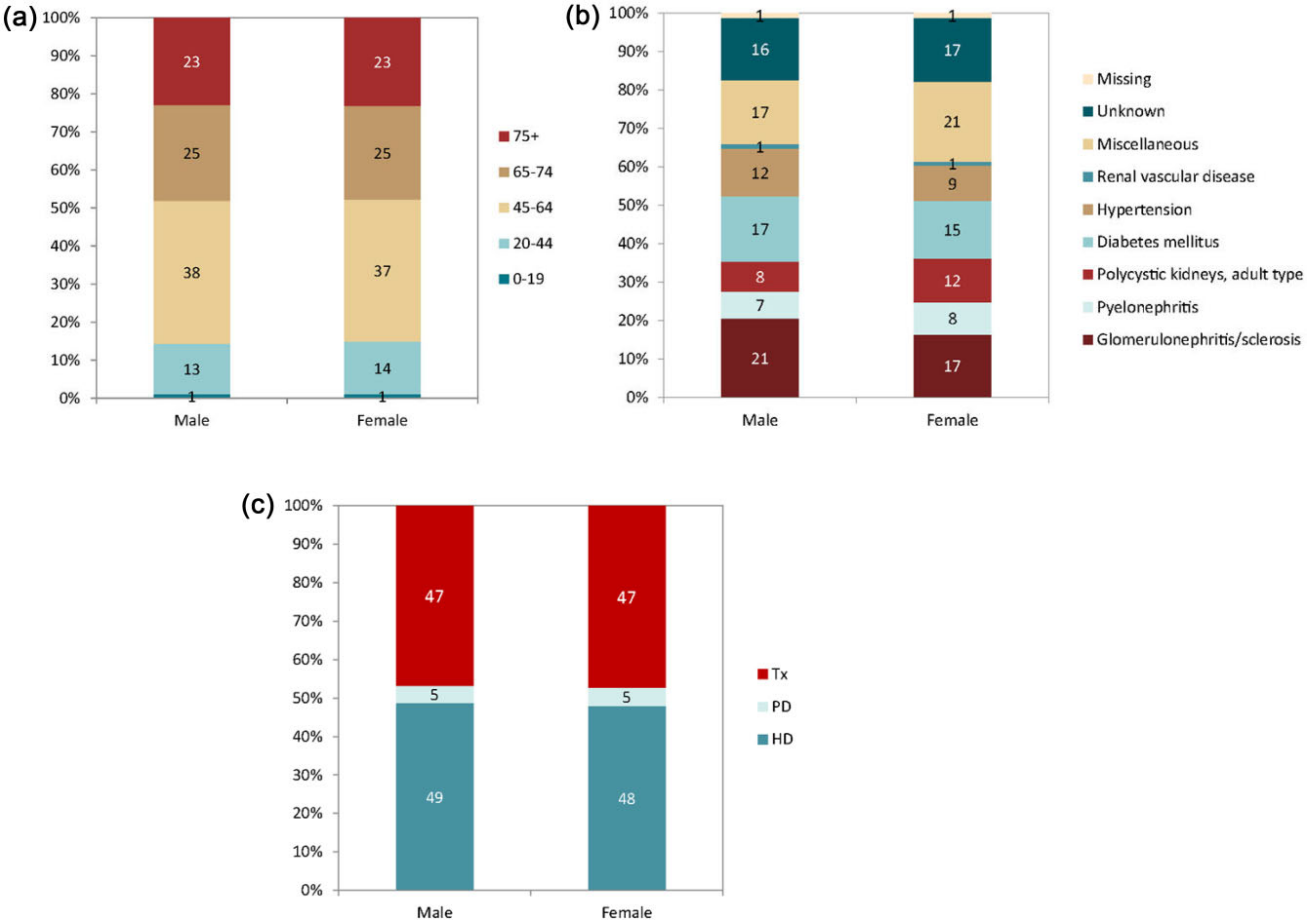
Distribution of (**a**) age, (**b**) PRD (1995 ERA codes), and (**c**) treatment modality by sex for prevalent patients on KRT on 31 December 2022, unadjusted. This figure is only based on data from registries providing individual patient data (see Appendix 1). Bars may not add up to 100% due to rounding. Abbreviation: Tx: transplantation.

On 31 December 2022, the prevalence of KRT was higher in males (670 pmp or 1 in 1500 inhabitants) than in females (411 pmp or 1 in 2400 inhabitants, Fig. 19). The proportion of females in prevalent KRT patients ranged from 32% in Cantabria, Spain to 45% in Ukraine (Fig. 20). The distribution of age and treatment modality was similar across males and females in countries providing individual patient data (Fig. 21). There were no major differences in the distribution of PRDs among prevalent males and females on KRT. Nevertheless, according to the 1995 ERA PRD codes, a higher percentage of males (21%) had glomerulonephritis/sclerosis as PRD than females (17%), while a higher percentage of females had polycystic kidney disease as PRD (12% in females vs 8% in males) or were grouped under miscellaneous (21% in females vs 17% in males, Fig. 21).

**Figure 22:**
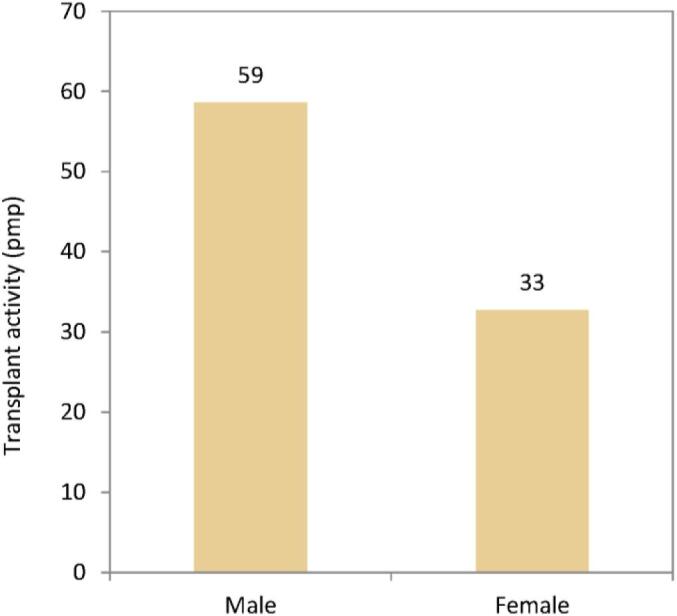
Kidney transplants per million population (pmp) by sex, unadjusted.

**Figure 23:**
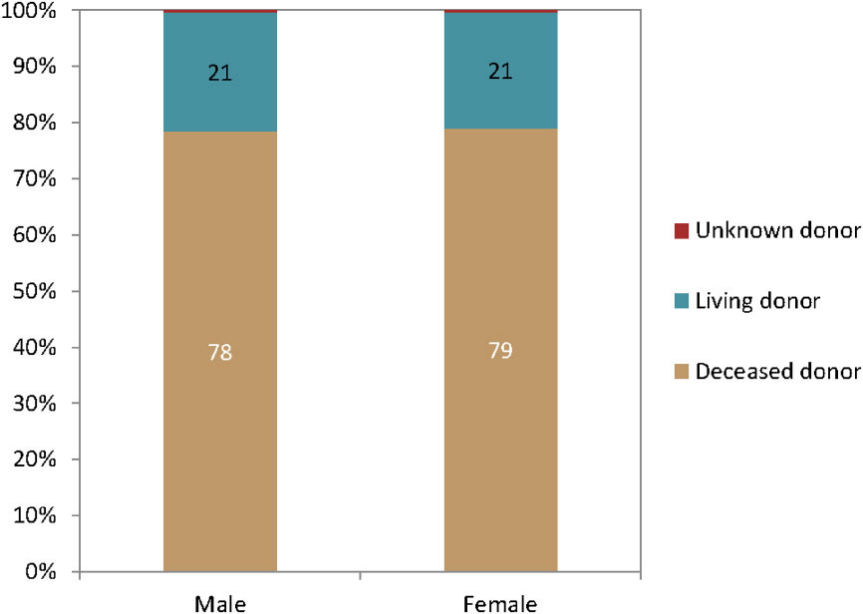
Donor type distribution by sex in kidney transplant recipients. Bars may not add up to 100% due to rounding.

**Figure 24:**
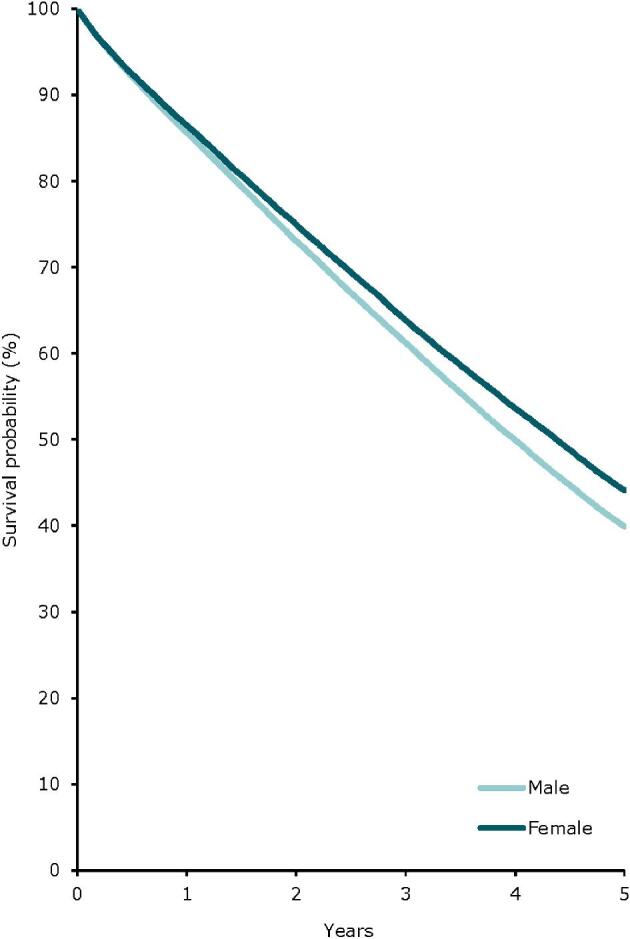
Patient survival in incident dialysis patients by sex from day 91 (cohort 2013–2017), unadjusted. See Appendix 2 for a list of countries and regions providing individual patient data included in the survival analyses.

**Figure 25:**
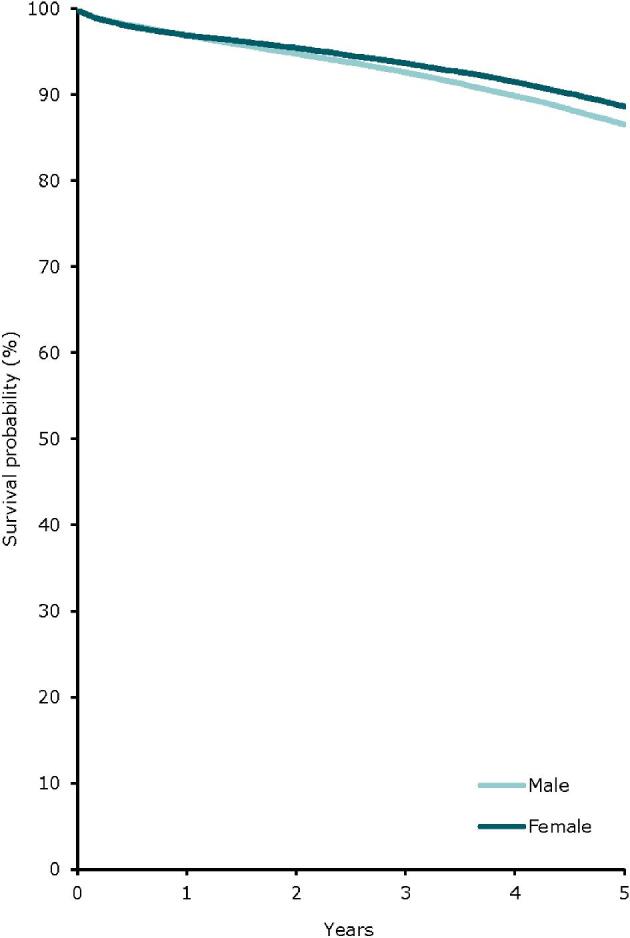
Patient survival in first-time kidney transplant recipients by sex from day of transplant (cohort 2013–2017), unadjusted. See Appendix 2 for a list of countries and regions providing individual patient data included in the survival analyses.

In 2022, the kidney transplantation rate was higher in males (59 pmp or 1 in 16 900 inhabitants) compared to females (33 pmp or 1 in 30 300 inhabitants, Fig. 22). However, the donor type distribution was similar across the sexes, with 21% of recipients receiving a kidney transplant from a LD (Fig. 23). Using data of patients on dialysis at day 91 during the period 2013–2017, the 5-year unadjusted patient survival was higher in females (44.1%) compared to males (39.9%, Fig. 24). Similarly, in patients receiving a first kidney transplant between 2013–2017, the unadjusted patient survival was slightly higher in female (88.6%) compared to male recipients (86.5%, Fig. 25).

## AFFILIATED REGISTRIES (CO-AUTHORS WILL BE REMOVED FROM THE ACKNOWLEDGEMENTS)

We would like to thank the patients and the staff of the dialysis and transplant units for contributing the data via their national and regional renal registries. Furthermore, we gratefully acknowledge the following registries and persons for their contribution of the data: Austrian Dialysis and Transplant Registry (OEDTR) (G. Mayer, J. Kerschbaum, and D. Kaiser-Feistmantl); Belarus Renal Registry (K. Kamisarau and A. Kalachyk); Dutch-speaking Belgian Society of Nephrology (NBVN) (L. Heylen, V. De Meyer, and J. De Meester); French-speaking Belgian Society of Nephrology (GNFB) (JM. des Grottes and F. Collart); Renal Registry Bosnia and Herzegovina (H. Resic, D. Rebic, N. Petkovic, and M. Tomic); Croatian Renal Registry (D. Katicic and K. Altabas); Cyprus Renal Registry (V. Scoutellas and M. Athanasiadou); Czech Republic: Registry of Dialysis Patients (RDP) (L. Francová); Danish Nephrology Registry (DNS); Estonian Society of Nephrology (Ü. Pechter and K. Lilienthal); Finnish Registry for Kidney Diseases (P. Finne); France: The Epidemiology and Information Network in Nephrology (REIN) (C. Couchoud); Hellenic Renal Registry (G. Moustakas); Hungarian Renal Registry (L. Wagner and E. Ladanyi); Icelandic End-Stage Renal Disease Registry (R. Palsson); Montenegro Renal Registry (F. Tomović); Israel National Registry of Renal Replacement Therapy (L. Keinan-Boker and R. Dichtiar); Italian Registry of Dialysis and Transplantation (RIDT) (M. Nordio and P.M. Ferraro); Kosovo Renal Registry (M. Tolaj Avdiu, V. Godanci Kelmendi, and F. Memeti Smaili); Latvian Renal Registry (I. Ziedina, K. Racenis, and A. Petersons); Lithuanian Renal Registry (I. Nedzelskiene and R. Gaidelyte); North Macedonian Renal Registry (I. Rambabova Bushljetikj, V. Tomanoski, and V. Krecova); Norwegian Renal Registry (A.V. Reisæter); Renal Registry of Poland (P. Jagodzinski and R. Gellert); Portuguese Renal Registry (E. Almeida); Romanian Renal Registry (RRR) (G. Mircescu, L. Garneata, and E. Podgoreanu); Renal Registry in Serbia (M. Lausevic and all dialysis units in Serbia); Slovakian Renal Registry (I. Lajdová and J. Rosenberger); Spain Renal Registry (B. Mahillo Durán); Swedish Renal Registry (SRR) (K.G. Prütz, M. Stendahl, M. Evans, T. Lundgren, H. Rydell, and M. Segelmark); Swiss Dialysis Registry (P. Ambühl); Dutch Renal Registry (Nefrodata) (P. Verschoor and L. Heuveling); Sfax Renal Registry (D. Zalila, F. Jarraya, and K. Kammoun); Registry of the Nephrology, Dialysis and Transplantation in Turkey (TSNNR) (I. Koçyigit and K. Ateş); Ukrainian Renal Data System (URDS) (M. Kolesnyk, O. Razvazhaieva, and N. Kozliuk); UK Renal Registry (All the staff of the UK Renal Registry and of the renal units submitting data); Scottish Renal Registry (SRR) (All of the Scottish renal units); and the regional registries of Andalusia (SICATA) [P. Castro de la Nuez (on behalf of all users of SICATA)], Aragon (F. Arribas Monzón), Asturias (M.R. Camblor, J.R. Quirós, and RERCA working group), Basque country (UNIPAR) (Á. Magaz, J. Aranzabal, M. Rodrigo, and I. Moina), Canary Islands (C. García Cantón and D. Marrero Miranda), Cantabria (J.C. Ruiz San Millán), Castile and León (P. Ucio Mingo and M. Prieto Velasco), Castile-La Mancha (G. Gutiérrez Ávila and I. Moreno Alía), Catalonia (RMRC) (J. Tort and M. Vázquez), Community of Madrid (A. Escribá Bárcenas), Extremadura [all the renal units (Nephrology and Dialysis) from Extremadura], Galicia (E. Bouzas-Caamaño), La Rioja (E. Huarte Loza and H. Hernández Vargas), Murcia (C. Santiuste de Pablos), Navarre (J. Manrique Escola), and Valencian region (O.L. Rodríguez-Arévalo).

## ERA REGISTRY COMMITTEE MEMBERS

R. Torra, Spain (ERA President); A. Ortiz, Spain (Chair); M. Arnol, Slovenia; A. Åsberg, Norway; S. Bakkaloglu, Turkey; P.M. Ferraro, Italy; J. Helve, Finland; J. Hogan, France; V. Kuzema, Latvia; B. Ponte, Switzerland; J.E. Sánchez-Álvarez, Spain; and M. Segelmark, Sweden.

## ERA REGISTRY OFFICE STAFF

V.S. Stel (Managing Director), M.E. Astley, R. Boenink, B.A. Boerstra, M. Bonthuis, N.C. Chesnaye, R. Cornet, Ö. Gök Pasayigit, K.J. Jager (former staff), A. Kramer, I.R. Montez de Sousa (ESPN/ERA Registry staff), and A.J. Weerstra.

## Supplementary Material

sfae405_Supplemental_File

## Data Availability

The data underlying this article have been published in the ERA Registry Annual Report 2022 (Supplementary Data).

## Appendix 1

### Countries or regions providing individual patient data to the ERA Registry

Austria, Belgium (Dutch-speaking), Belgium (French-speaking), Bosnia and Herzegovina, Denmark, Estonia, France, Greece, Iceland, Montenegro, Norway, Romania, Serbia, Spain (Andalusia), Spain (Aragon), Spain (Asturias), Spain (Basque country), Spain (Canary Islands), Spain (Cantabria), Spain (Castile and León), Spain (Castile-La Mancha), Spain (Catalonia), Spain (Community of Madrid), Spain (Extremadura), Spain (Galicia), Spain (La Rioja), Spain (Murcia), Spain (Navarre), Spain (Valencian region), Sweden, Switzerland, the Netherlands, UK (England/Northern Ireland/Wales), and UK (Scotland).

### Countries or regions providing aggregated data to the ERA Registry

Belarus, Croatia, Cyprus, Czech Republic, Finland, Hungary, Israel, Italy, Kosovo, Latvia, Lithuania, North Macedonia, Poland, Portugal, Slovakia, Spain, Tunisia (Sfax region), Turkey, and Ukraine.

### Countries part of the European Union (EU27) population as of 1 February 2020 (used as a reference population)

Austria, Belgium, Bulgaria, Croatia, Cyprus, Czech Republic, Denmark, Estonia, Finland, France, Germany, Greece, Hungary, Ireland, Italy, Latvia, Lithuania, Luxembourg, Malta, the Netherlands, Poland, Portugal, Romania, Slovakia, Slovenia, Spain, and Sweden.

## Appendix 2

### Countries or regions included in the survival/expected remaining years of life analyses

Austria, Belgium (Dutch-speaking), Belgium (French-speaking), Bosnia and Herzegovina, Denmark, Estonia, France, Greece, Iceland, Norway, Spain (Andalusia), Spain (Aragon), Spain (Asturias), Spain (Basque country), Spain (Canary Islands), Spain (Cantabria), Spain (Castille and León), Spain (Castille-La Mancha), Spain (Catalonia), Spain (Community of Madrid), Spain (Extremadura), Spain (Galicia), Spain (Murcia), Spain (Navarre), Spain (Valencian Region), Sweden, the Netherlands, UK (England/Northern Ireland/Wales), and UK (Scotland).
