# Correction to: The ERA Registry Annual Report 2022: Epidemiology of Kidney Replacement Therapy in Europe, with a focus on sex comparison

**DOI:** 10.1093/ckj/sfaf107

**Published:** 2025-05-05

This is a correction to: Rianne Boenink (*et al*), The ERA Registry Annual Report 2022: Epidemiology of Kidney Replacement Therapy in Europe, with a focus on sex comparisons, *Clinical Kidney Journal*, Volume 18, Issue 2, February 2025, sfae405, https://doi.org/10.1093/ckj/sfae405

The following changes have been made to the originally published manuscript. A section heading is emended in the paper to read: ‘AFFILIATED REGISTRIES’ instead of ‘AFFILIATED REGISTRIES (CO-AUTHORS WILL BE REMOVED FROM THE ACKNOWLEDGEMENTS)’.

