# Supplementary material for: The ERA Registry Annual Report 2022: Epidemiology of Kidney Replacement Therapy in Europe, with a focus on sex comparisons
Source: Clin Kidney J. 2024 Dec 12;18(2):sfae405. doi: 10.1093/ckj/sfae405 (PMC11852260; doi:10.1093/ckj/sfae405)
Supplement: sfae405_Supplemental_File [file sfae405_Supplemental_File.pdf]

ERA Registry Annual Report

2022

Suggested Citation Once the ERA Registry annual report 2022 is published as a supplement in the article "The ERA Registry Annual Report 2022: summary and sex comparisons." in Clinical Kidney Journal (CKJ), the suggested citation will be the reference to this article in CKJ. Until then the suggested citation is:

ERA Registry: ERA Registry Annual Report 2022. Amsterdam UMC, location AMC, Department of Medical Informatics, Amsterdam, the Netherlands, 2024.

Disclaimer ERA, its Registry Committee, Amsterdam UMC; location AMC, and its department of Medical Informatics disclaim any express or implied warranty of fitness for the use of ERA Registry products and shall not be liable for any direct, indirect or consequential loss; personal injury; special or punitive damages; loss of profits, loss of savings and loss of revenue; loss of business, loss of reputation and loss of goodwill; and loss of data arising from the use of ERA Registry products.

ERA Registry
Annual Report 2022

ERA Registry

Amsterdam UMC, location AMC
Department of Medical Informatics
PO Box 22700
1100 DE Amsterdam
the Netherlands

E-mail erareg@amsterdamumc.nl

Website www.era-online.org/research-education/era-registry

ERA Registry Committee

R Torra, Spain (ERA President)

A Ortiz, Spain (Chair)

M Arnol, Slovenia
A Åsberg, Norway
S Bakkaloglu, Turkey
PM Ferraro, Italy
J Helve, Finland
V Kuzema, Latvia
B Ponte, Switzerland
JE Sánchez-Álvarez, Spain
M Segelmark, Sweden
E Vidal, Italy

ERA Registry Office Staff

VS Stel, senior epidemiologist (Director)

ME Astley, PhD student
R Boenink, epidemiologist
BA Boerstra, PhD student
M Bonthuis, epidemiologist
NC Chesnaye, epidemiologist
R Cornet, senior medical informatician (advisor)
Ö Gök Pasayigit, management assistant
KJ Jager, senior epidemiologist
A Kramer, senior medical informatician
IR Montez de Sousa, PhD student (for paediatric section)
AJ Weerstra, software engineer

Acknowledgements

The ERA Registry would like to thank the patients and staff of all the dialysis and transplant units who have contributed data via their national and regional renal registries. In addition, we would like to thank the following persons and organizations for their contribution to the work of the ERA Registry.

For the provision of individual patient data

Registry	Contributors
Austria	G Mayer, J Kerschbaum, L Buchwinkler, and D Kaiser-Feistmantl
Belgium, Dutch-speaking	L Heylen, V De Meyer, and J De Meester
Belgium, French-speaking	JM des Grottes and F Collart
Bosnia and Herzegovina	H Resic, D Rebic, N Petkovic, and M Tomic
Denmark	K Hommel
Estonia	Ü Pechter, M Ots-Rosenberg, and K Lilienthal
France (17 of 18 regions)	M Lassalle and C Couchoud
Greece	G Moustakas
Iceland	OS Indridason and R Palsson
Montenegro	F Tomović and D Radunović
Norway	AV Reisæter and A Åsberg
Romania	G Mircescu, L Garneata, and E Podgoreanu
Serbia	M Lausevic, M Kravljaca, and all dialysis units in Serbia
Spain, Andalusia	P Castro de la Nuez (on behalf of all users of SICATA)
Spain, Aragon	F Arribas Monzón
Spain, Asturias	MR Camblor, JR Quirós, and RERCA working group
Spain, Basque country	Á Magaz, J Aranzabal, M Rodrigo, and I Moina
Spain, Canary Islands	S Trujillo Alemán, C García Cantón, and D Marrero Miranda
Spain, Cantabria	JC Ruiz San Millán
Spain, Castile and León	P Ucio Mingo, M Prieto Velasco, and H García López
Spain, Castile-La Mancha	G Gutiérrez Ávila and I Moreno Alía
Spain, Catalonia	J Tort, J Comas, and M Vázquez
Spain, Community of Madrid	A Escribá Bárcenas and M Marqués Vidas
Spain, Extremadura	All the renal units (Nephrology and Dialysis) from Extremadura
Spain, Galicia	E Bouzas-Caamaño
Spain, La Rioja	E Huarte Loza, M Artamendi Larrañaga, and H Hernández Vargas
Spain, Murcia	I Marín Sánchez and C Santiuste de Pablos
Spain, Navarre	MF Slon Roblero and J Manrique Escola
Spain, Valencian region	OL Rodríguez-Arévalo and A Sarrión
Sweden	KG Prütz, M Stendahl, M Evans, T Lundgren, H Rydell, and M Segelmark
Switzerland	P Ambühl and R Guidotti
the Netherlands	P Verschoor, L Heuveling, and M ten Dam
United Kingdom, England/Northern Ireland/Wales	All the staff of the UK Renal Registry and of the renal units submitting data
United Kingdom, Scotland	All of the Scottish renal units

For the provision of aggregated data

Registry	Contributors
Belarus	K Komissarov, K Kamisarau, and A Kalachyk
Croatia	D Katicic and K Altabas
Cyprus	N Mitsides, V Scoutellas, and M Athanasiadou
Czech Republic	I Rychlík, L Francová, and F Lopot
Finland	J Helve and P Finne
Hungary	C Ambrus, L Wagner, and E Ladanyi
Israel	L Keinan-Boker, R Dichtiar, and P Beckerman
Italy (8 of 20 regions)	M Nordio and PM Ferraro
Kosovo	M Tolaj Avdiu, V Godanci Kelmendi, and F Memeti Smaili
Latvia	V Kuzema
Lithuania	I Nedzelskiene, R Gaidelyte, and E Ziginskiene
North Macedonia	I Rambabova-Bushljetik, V Tomanoski, V Krecova, and N.Gjorgjievski
Poland	A Debska-Slizien, P Jagodzinski, and R Gellert
Portugal	A Galvão and E Almeida
Slovakia	I Lajdová, A Okša, and J Rosenberger
Spain	B Mahillo Durán and Spanish Regional Registries
Tunisia, Sfax region	D Zalila, F Jarraya, and K Kammoun
Turkey	N Seyahi, I Koçyigit, and K Ates
Ukraine	M Kolesnyk, O Razvazhaieva, and N Kozliuk

The ERA Registry is funded by the European Renal Association (ERA).

List of abbreviations

Abbreviation	Term
APD	Automated peritoneal dialysis
CAKUT	Congenital anomalies of the kidney and urinary tract
CAPD	Continuous ambulatory peritoneal dialysis
CI	Confidence interval
DM	Diabetes mellitus
ERA	European Renal Association
ESPN	European Society for Paediatric Nephrology
ESRD	End-stage renal disease
GN	Glomerulonephritis / sclerosis
HD	Haemodialysis
HDF	Haemodiafiltration
HF	Haemofiltration
HT	Hypertension
HUS	Haemolytic uraemic syndrome
KRT	Kidney replacement therapy
Misc	Miscellaneous
N	Number
P25	Refers to the 25th percentile
P75	Refers to the 75th percentile
PD	Peritoneal dialysis
PKD	Polycystic kidneys, adult type
Pmarp	Per million age-related population
Pmp	Per million population
PN	Pyelonephritis
PRD	Primary renal disease
RVD	Renal vascular disease
SD	Standard deviation
Tx	Transplant
Un	Unknown
Unkn	Unknown

Glossary

EU27: refers to the 27 countries of which on 1 February 2020 the European Union was comprised of. These countries are: Austria, Belgium, Bulgaria, Croatia, Cyprus, Czech Republic, Denmark, Estonia, Finland, France, Germany, Greece, Hungary, Ireland, Italy, Latvia, Lithuania, Luxembourg, Malta, the Netherlands, Poland, Portugal, Romania, Slovakia, Slovenia, Spain, and Sweden.

Eurostat: the statistical office of the European Union whose task is to provide the European Union with statistics at the European level that enable comparisons between countries and regions.

Expected remaining lifetime: is defined as the average number of years of life remaining for those who have reached a given age.

Incidence: the number of new cases during a specific time period. In this annual report this equates to the number of patients commencing kidney replacement therapy for end-stage renal disease during the calendar year on which this annual report is based.

Kidney replacement therapy (KRT): therapy used to replace the normal blood-filtering function of the kidneys. In this annual report kidney replacement therapy refers to the various dialysis modalities and to kidney transplantation (see Modality).

Modality: the method of kidney replacement therapy. Kidney replacement therapy is comprised of the following modalities: haemodialysis (HD), haemofiltration (HF), haemodiafiltration (HDF), automated peritoneal dialysis (APD), continuous ambulatory peritoneal dialysis (CAPD), and kidney transplantation (Tx). Kidney transplantation can be performed with a kidney from a living donor (LD) or from a deceased donor (DD).

Per million age-related population (pmarp): the incidence or prevalence pmarp is the observed incident or prevalent count for a specific age group divided by the general population of that age group and multiplied by one million.

Per million population (pmp): the incidence or prevalence pmp is the observed incident or prevalent count divided by the general population in that year and multiplied by one million.

Percentage coverage: coverage of the general population in a country or region by the registry of that country or region.

Prevalence: the number of people in a given population with a particular disease at a given time. In this annual report this equates to the number of patients receiving kidney replacement therapy for end-stage renal disease on 31st December 2022.

Survival probability: the probability that a person or a kidney transplant has survived up to a specified time point.

Brief content

Introduction 11

Section A: Summary data and comparisons by sex distribution 14

A1 INCIDENT PATIENTS ACCEPTED FOR KRT IN 2022, AT DAY 1 15
A2 INCIDENT PATIENTS ACCEPTED FOR KRT IN 2022, AT DAY 91 21
A3 PREVALENT PATIENTS ON KRT IN 2022 22
A4 KIDNEY TRANSPLANTS IN 2022 27
A5 SURVIVAL PROBABILITY 30
A6 EXPECTED REMAINING LIFETIMES 32
A7 COMPARISONS BY SEX DISTRIBUTION FOR 2022 33

Section B: Individual patient data reference tables 42

B1 AFFILIATED REGISTRY INFORMATION FOR 2022 43
B2 INCIDENT PATIENTS ACCEPTED FOR KRT IN 2022, AT DAY 1 44
B3 INCIDENT PATIENTS ACCEPTED FOR KRT IN 2022, AT DAY 91 57
B4 PREVALENT PATIENTS ON KRT IN 2022 70
B5 KIDNEY TRANSPLANTS IN 2022 81
B6 SURVIVAL PROBABILITY 84
B7 EXPECTED REMAINING LIFETIMES 100

Section C: Aggregated data reference tables 101

C1 AFFILIATED REGISTRY INFORMATION FOR 2022 102
C2 INCIDENT PATIENTS ACCEPTED FOR KRT IN 2022, AT DAY 1 103
C3 INCIDENT PATIENTS ACCEPTED FOR KRT IN 2022, AT DAY 91 111
C4 PREVALENT PATIENTS ON KRT IN 2022 119
C5 KIDNEY TRANSPLANTS IN 2022 127

Section D: Paediatric data reference tables 129

D1 GENERAL POPULATION AGE DISTRIBUTION 130
D2 INCIDENT PATIENTS ACCEPTED FOR KRT, AT DAY 1 131
D3 PREVALENT PATIENTS ON KRT 133

Methods 135

Appendices 144

Table of Contents

Introduction 11

Section overview 13
New to this edition 13
ERA Registry website 13
ESPN/ERA Registry website 13

Section A: Summary data and comparisons by sex distribution 14

A1 INCIDENT PATIENTS ACCEPTED FOR KRT IN 2022, AT DAY 1

Table A.1.1 Summary data on the incidence of KRT in 2022, unadjusted 15
Figure A.1.1 Incidence per million population by country / region, unadjusted 16
Figure A.1.2 Incidence per million population by country / region, unadjusted and adjusted 17
Figure A.1.3 Age, sex, and primary renal disease distribution by type of data provided, unadjusted 18
Figure A.1.4 Treatment modality distribution by type of data provided, age, sex and primary renal disease, unadjusted 20

A2 INCIDENT PATIENTS ACCEPTED FOR KRT IN 2022, AT DAY 91

Figure A.2.1 Treatment modality distribution by type of data provided, age, sex and primary renal disease, unadjusted 21

A3 PREVALENT PATIENTS ON KRT IN 2022

Table A.3.1 Summary data on the prevalence of KRT on 31 December 2022, unadjusted 22
Figure A.3.1 Prevalence per million population by country / region, unadjusted 23
Figure A.3.2 Prevalence per million population by country / region, unadjusted and adjusted 24
Figure A.3.3 Age, sex, and primary renal disease distribution by type of data provided, unadjusted 25
Figure A.3.4 Treatment modality distribution by type of data provided, age, sex and primary renal disease, unadjusted 26

A4 KIDNEY TRANSPLANTS IN 2022

Figure A.4.1 Kidney transplant counts and per million population by country / region, unadjusted 27
Figure A.4.2 Kidney transplants per million population by donor type and by country / region, unadjusted 28
Figure A.4.3 Donor type distribution by type of data provided, unadjusted 29

A5 SURVIVAL PROBABILITY

Table A.5.1 One-, two- and five-year survival probabilities by treatment modality and cohort 30
Figure A.5.1 Patient survival by modality: Incident dialysis patients (cohort 2013-2017) 31
Figure A.5.2 Patient survival by donor type: Patients receiving a first kidney transplant (cohort 2013-2017) 31

A6 EXPECTED REMAINING LIFETIMES

Figure A.6.1 Expected remaining years of life in the general population and in prevalent dialysis and kidney transplant patients (cohort 2018-2022) 32

A7 COMPARISONS BY SEX DISTRIBUTION FOR 2022

Figure A.7.1 Sex distribution by country / region, unadjusted 33
Figure A.7.2 Incidence per million population by sex, unadjusted 34
Figure A.7.3 Age, primary renal disease and treatment modality distribution by sex in incident patients, unadjusted 35
Figure A.7.4 Sex distribution by country / region, unadjusted 37
Figure A.7.5 Prevalence per million population by sex, unadjusted 38
Figure A.7.6 Age, primary renal disease and treatment modality distribution by sex in prevalent patients, unadjusted 39
Figure A.7.7 Kidney transplants per million population by sex 40
Figure A.7.8 Donor type distribution by sex in kidney transplant recipients, unadjusted 40
Figure A.7.9 Patient survival probability by sex: Incident dialysis patients (cohort 2013-2017) 41
Figure A.7.10 Patient survival probability by sex: Patients receiving a first kidney transplant (cohort 2013-2017) 41

Section B: Individual patient data reference tables 42

B1 AFFILIATED REGISTRY INFORMATION FOR 2022

Table B.1.1 General population data and number of renal centres 43

B2 INCIDENT PATIENTS ACCEPTED FOR KRT IN 2022, AT DAY 1

Table B.2.1 Incident counts and percentages by age and sex 44
Table B.2.2 Incidence per million (age-related) population by age and sex, unadjusted 45
Table B.2.3 Sex, mean age, and median age of incident patients 46
Table B.2.4 Incidence per million population, adjusted 47
Table B.2.5.A Incidence per million population and percentages by primary renal disease (1995 PRD codes), unadjusted 48
Table B.2.5.B Incidence per million population and percentages by primary renal disease (2012/2018 PRD codes), unadjusted 49
Table B.2.6.A Incidence per million population by primary renal disease (1995 PRD codes), adjusted 50
Table B.2.6.B Incidence per million population by primary renal disease (2012/2018 PRD codes), adjusted 51
Table B.2.7 Incident counts by treatment modality 52
Table B.2.8 Incidence per million population by treatment modality, unadjusted 53
Table B.2.9 Incidence per million population by treatment modality, adjusted 54
Table B.2.10 Treatment modality distribution, unadjusted 55
Table B.2.11 Treatment modality distribution by age, sex, and primary renal disease, unadjusted 56

B3 INCIDENT PATIENTS ACCEPTED FOR KRT IN 2022, AT DAY 91

Table B.3.1 Incident counts and percentages by age and sex 57
Table B.3.2 Incidence per million (age-related) population by age and sex, unadjusted 58
Table B.3.3 Sex, mean age, and median age of incident patients 59
Table B.3.4 Incidence per million population, adjusted 60
Table B.3.5.A Incidence per million population and percentages by primary renal disease (1995 PRD codes), unadjusted 61
Table B.3.5.B Incidence per million population and percentages by primary renal disease (2012/2018 PRD codes), unadjusted 62
Table B.3.6.A Incidence per million population by primary renal disease (1995 PRD codes), adjusted 63
Table B.3.6.B Incidence per million population by primary renal disease (2012/2018 PRD codes), adjusted 64
Table B.3.7 Incident counts by treatment modality 65
Table B.3.8 Incidence per million population by treatment modality, unadjusted 66
Table B.3.9 Incidence per million population by treatment modality, adjusted 67
Table B.3.10 Treatment modality distribution, unadjusted 68
Table B.3.11 Treatment modality distribution by age, sex, and primary renal disease, unadjusted 69

B4 PREVALENT PATIENTS ON KRT IN 2022

Table B.4.1 Prevalent counts and percentages by age and sex 70
Table B.4.2 Prevalence per million (age-related) population by age and sex, unadjusted 71
Table B.4.3 Sex, mean age, and median age 72
Table B.4.4 Prevalence per million population, adjusted 73
Table B.4.5 Prevalence per million population and percentages by primary renal disease (1995 PRD codes), unadjusted 74
Table B.4.6 Prevalence per million population by primary renal disease (1995 PRD codes), adjusted 75
Table B.4.7 Prevalent counts by treatment modality 76
Table B.4.8 Prevalence per million population by treatment modality, unadjusted 77
Table B.4.9 Prevalence per million population by treatment modality, adjusted 78
Table B.4.10 Treatment modality distribution, unadjusted 79
Table B.4.11 Treatment modality distribution by age, sex, and primary renal disease, unadjusted 80

B5 KIDNEY TRANSPLANTS IN 2022

Table B.5.1 Kidney transplant counts and percentages by donor type 81
Table B.5.2 Kidney transplants per million population by donor type, unadjusted 82
Table B.5.3 Sex, mean age, and median age of kidney transplant recipients 83

B6 SURVIVAL PROBABILITY

Patient Survival on KRT (cohort 2013-2017 and cohort 2016-2020)

Table B.6.1 Incident KRT patients, from day 1, unadjusted 84
Table B.6.2 Incident KRT patients, from day 1, adjusted 85
Table B.6.3 Incident KRT patients, from day 91, unadjusted 86
Table B.6.4 Incident KRT patients, from day 91, adjusted 87

Patient Survival on Dialysis (cohort 2013-2017 and cohort 2016-2020)

Table B.6.5 Incident dialysis patients, from day 1, unadjusted 88
Table B.6.6 Incident dialysis patients, from day 1, adjusted 89
Table B.6.7 Incident dialysis patients, from day 91, unadjusted 90
Table B.6.8 Incident dialysis patients, from day 91, adjusted 91

Patient Survival after First Kidney Transplant (cohort 2013-2017 and cohort 2016-2020)

Table B.6.9 First transplant patients (deceased donor), from day of transplant, unadjusted 92
Table B.6.10 First transplant patients (deceased donor), from day of transplant, adjusted 93
Table B.6.11 First transplant patients (living donor), from day of transplant, unadjusted 94
Table B.6.12 First transplant patients (living donor), from day of transplant, adjusted 95

Graft Survival after First Kidney Transplant (cohort 2013-2017 and cohort 2016-2020)

Table B.6.13 First graft (deceased donor), from day of transplant, unadjusted 96
Table B.6.14 First graft (deceased donor), from day of transplant, adjusted 97
Table B.6.15 First graft (living donor), from day of transplant, unadjusted 98
Table B.6.16 First graft (living donor), from day of transplant, adjusted 99

B7 EXPECTED REMAINING LIFETIMES

Table B.7.1 Expected remaining years of life in the general population and in prevalent dialysis and kidney transplant patients (cohort 2018-2022) 100

Section C: Aggregated data reference tables 101

C1 AFFILIATED REGISTRY INFORMATION FOR 2022

Table C.1.1 General population data and number of renal centres 102

C2 INCIDENT PATIENTS ACCEPTED FOR KRT IN 2022, AT DAY 1

Table C.2.1 Incident counts and percentages by age and sex 103
Table C.2.2 Incidence per million (age-related) population by age and sex, unadjusted 104
Table C.2.3 Sex, mean age, and median age of incident patients 105
Table C.2.4 Incidence per million population, adjusted 106
Table C.2.5 Incidence per million population and percentages by primary renal disease (1995 PRD codes), unadjusted 107
Table C.2.6 Incident counts by treatment modality 108
Table C.2.7 Incidence per million population by treatment modality, unadjusted 109
Table C.2.8 Treatment modality distribution, unadjusted 110

C3 INCIDENT PATIENTS ACCEPTED FOR KRT IN 2022, AT DAY 91

Table C.3.1 Incident counts and percentages by age and sex 111
Table C.3.2 Incidence per million (age-related) population by age and sex, unadjusted 112
Table C.3.3 Sex, mean age, and median age of incident patients 113
Table C.3.4 Incidence per million population, adjusted 114
Table C.3.5 Incidence per million population and percentages by primary renal disease (1995 PRD codes), unadjusted 115
Table C.3.6 Incident counts by treatment modality 116
Table C.3.7 Incidence per million population by treatment modality, unadjusted 117
Table C.3.8 Treatment modality distribution, unadjusted 118

C4 PREVALENT PATIENTS ON KRT IN 2022

Table C.4.1 Prevalent counts and percentages by age and sex 119
Table C.4.2 Prevalence per million (age-related) population by age and sex, unadjusted 120
Table C.4.3 Sex, mean age, and median age 121
Table C.4.4 Prevalence per million population, adjusted 122
Table C.4.5 Prevalence per million population and percentages by primary renal disease (1995 PRD codes), unadjusted 123
Table C.4.6 Prevalent counts by treatment modality 124
Table C.4.7 Prevalence per million population by treatment modality, unadjusted 125
Table C.4.8 Treatment modality distribution, unadjusted 126

C5 KIDNEY TRANSPLANTS IN 2022

Table C.5.1 Kidney transplant counts and percentages by donor type 127
Table C.5.2 Kidney transplants per million population by donor type, unadjusted 128

Section D: Paediatric data reference tables 129

D1 GENERAL POPULATION AGE DISTRIBUTION

Table D.1.1 Population covered (in thousands) 130

D2 INCIDENT PATIENTS ACCEPTED FOR KRT, AT DAY 1

Table D.2.1 Incident counts by age and cohort 131
Table D.2.2 Incident counts by age, treatment modality, and cohort 131
Table D.2.3 Incident counts by age and primary renal disease 131
Table D.2.4 Incidence per million age-related population by age and cohort 132
Table D.2.5 Incidence per million age-related population by age, treatment modality, and cohort 132
Table D.2.6 Incidence per million age-related population by age and primary renal disease 132

D3 PREVALENT PATIENTS ON KRT

Table D.3.1 Prevalent counts by age and cohort 133
Table D.3.2 Prevalent counts by age, treatment modality, and cohort 133
Table D.3.3 Prevalent counts by age and primary renal disease 133
Table D.3.4 Prevalence per million age-related population by age and cohort 134
Table D.3.5 Prevalence per million age-related population by age, treatment modality, and cohort 134
Table D.3.6 Prevalence per million age-related population by age and primary renal disease 134

Methods 135

Data collection and preparation 136
Coding systems 139
Statistical analyses 139
Bibliography 143

Appendices 144

Appendix 1 - Grouping of primary renal disease codes 1995 145
Appendix 2 - Grouping of primary renal disease codes 2018 146
Appendix 3 - Grouping of causes of death 151
Appendix 4 - Event type codes 152
Appendix 5 - Renal registries contributing data for the different types of analyses 153

Introduction

Figure 1: National and regional renal registries that contributed data to this annual report

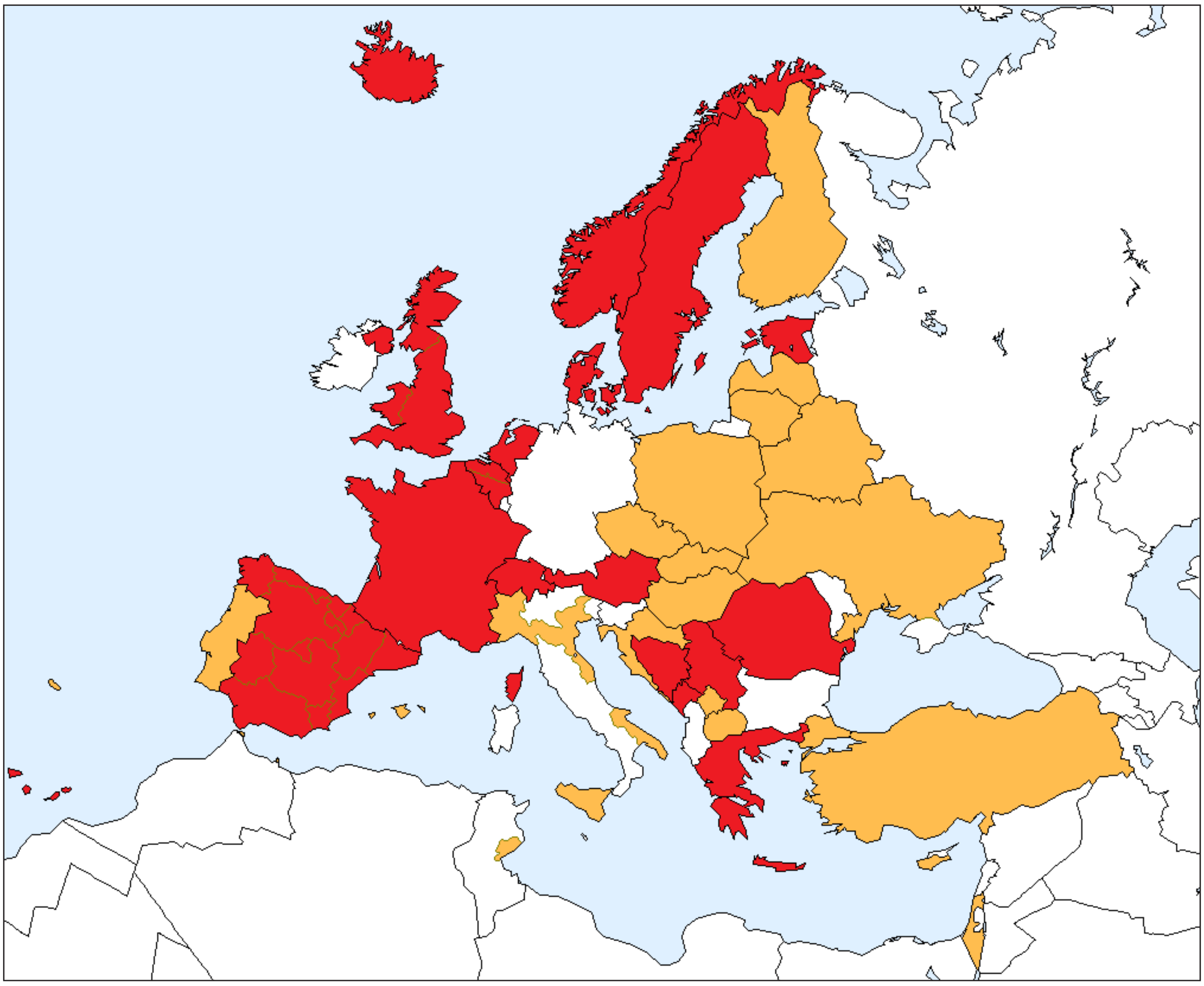

registries contributing individual patient data

registries contributing aggregated data

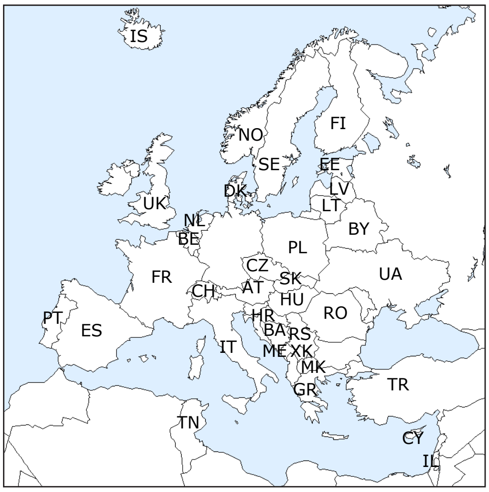

AT	Austria	LT	Lithuania
BA	Bosnia and Herzegovina	LV	Latvia
BE	Belgium	ME	Montenegro
BY	Belarus	MK	North Macedonia
CH	Switzerland	NL	the Netherlands
CY	Cyprus	NO	Norway
CZ	Czech Republic	PL	Poland
DK	Denmark	PT	Portugal
EE	Estonia	RO	Romania
ES	Spain	RS	Serbia
FI	Finland	SE	Sweden
FR	France	SK	Slovakia
GR	Greece	TN	Tunisia
HR	Croatia	TR	Turkey
HU	Hungary	UA	Ukraine
IL	Israel	UK	United Kingdom
IS	Iceland	XK	Kosovo
IT	Italy		

I Introduction

The ERA Registry collects data on kidney replacement therapy (KRT) via the national and regional renal registries in Europe and countries bordering the Mediterranean Sea. For this 2022 Annual Report data sets from 53 national or regional renal registries in 35 countries were used. The reference tables are presented in two parts: Section B relates to individual patient data from 34 national and regional registries from 17 countries (shown in red in Figure 1), whereas Section C relates to 19 national and regional registries from 19 countries providing aggregated data (shown in orange in Figure 1).

Section overview

In Section A, figures summarizing the data from all participating registries are presented. In addition, in Section A of this 2022 Annual Report, figures on comparisons by sex are included. Section B contains data on the incidence and prevalence of KRT and on the kidney transplant activity, the survival and expected remaining lifetimes from registries providing individual patient data. Section C covers data on the incidence and prevalence and kidney transplant activity from the countries and regions providing aggregated data. The incidence and prevalence of KRT in paediatric patients are described in Section D, and are based on data from registries providing individual paediatric patient data. The Methods section describes the procedures used, including data collection and preparation, definitions, coding systems, and statistical methods.

New to this edition

For the first time, figures and tables are included showing the distribution of primary renal diseases (PRD) based on the 2018 ERA PRD categorization (in addition to the 1995 ERA PRD categorization), for incident patients accepted for KRT in 2022.

ERA Registry website

The ERA Registry annual reports, the reference tables and the slides based on the information of these annual reports can be downloaded from the ERA website: www.era-online.org/research-education/era-registry. In addition, the website includes a repository of educational publications and a link to the 2018 version of the ERA Registry Primary Renal Disease Codes.

ESPN/ERA Registry website

Information on the activities of the European Society for Paediatric Nephrology (ESPN)/ERA Registry and on paediatric registries in general can be found on the ESPN/ERA Registry website: www.espn-reg.org. In addition, the ESPN/ERA Registry annual reports can be downloaded from this website.

Section A: Summary data and comparisons by sex distribution

Table A.1.1
Summary data on the incidence of KRT in 2022, unadjusted

	General population covered by the registry in thousands	Incidence in 2022, at day 1					
		All N	All Pmp	Mean Age	Median Age	DM N	DM Pmp
Austria §	8799	1073	122	65.2	67.8	251	29
Belarus ‖	8490	888	105			160	19
Belgium, Dutch-speaking *	6749	1177	174	70.4	73.5	242	36
Belgium, French-speaking *	4931	978	198	68.4	71.4	189	38
Bosnia and Herzegovina	3531	424	120	63.5	65.9	125	35
Croatia †	3162	472	149	71.0	72.0	142	45
Cyprus	905	277	306	69.6	71.0	107	118
Czech Republic †	10611	2502	236				
Denmark	5903	707	120	63.0	65.7	195	33
Estonia	1349	112	83	61.2	62.4	20	15
Finland	5564	465	84	62.1	65.1	143	26
France (17 of 18 regions)	67614	10989	163	66.9	70.4	2448	36
Greece	10437	2907	279	72.0	74.7	660	63
Hungary	9689	2186	226	65.0	68.0	1070	110
Iceland	382	33	86	55.9	61.8	7	18
Israel	9557	1703	178	65.8	69.4	750	78
Italy (8 of 20 regions)	27261	4362	160	68.7	71.4	577	21
Kosovo ‖	1688	254	151	62.4	66.0	97	57
Latvia	1670	114	68	59.8	62.0	24	14
Lithuania	2806	227	81	62.6	64.3	37	13
Montenegro *	617	49	79	63.4	67.4	14	23
North Macedonia	1830	385	210	63.6	66.0	85	46
Norway	5457	553	101	64.2	67.5	93	17
Poland	37827	6068	160			1523	40
Portugal ¶	10467	2515	240			731	70
Romania	19049	3654	192	63.0	65.6	400	21
Serbia	6383	500	78	61.9	65.4	97	15
Slovakia †	4362	710	163	62.9	65.0	215	49
Spain (All)	47475	7136	150	63.8	68.3	1574	33
Spain, Andalusia	8542	1281	150	64.8	68.2	321	38
Spain, Aragon	1343	191	142	64.6	68.2	42	31
Spain, Asturias	1006	193	192	68.6	71.6	47	47
Spain, Basque country	2213	300	136	64.2	66.7	62	28
Spain, Canary Islands	2199	392	178	63.9	66.6	123	56
Spain, Cantabria *	587	92	157	66.6	70.4	15	26
Spain, Castile and León *	2373	318	134	68.6	71.4	76	32
Spain, Castile-La Mancha *	2069	241	116	66.7	67.6	56	27
Spain, Catalonia	7793	1422	182	66.4	69.7	270	35
Spain, Community of Madrid	6413	702	109	64.1	66.5	139	22
Spain, Extremadura	1055	167	158	65.5	69.0	31	29
Spain, Galicia	2696	409	152	66.3	67.9	93	34
Spain, La Rioja	321	40	125	65.9	66.5	5	16
Spain, Murcia	1532	252	165	65.6	69.6	64	42
Spain, Navarre *	668	86	129	62.8	64.8	24	36
Spain, Valencian region	5098	768	151	66.4	69.0	139	27
Sweden	10487	1115	106	64.6	68.8	270	26
Switzerland	8689	889	102	65.7	69.8	185	21
the Netherlands	16285	1902	117	62.5	66.0	370	23
Tunisia, Sfax region †	1023	184	179	61.9	65.0	56	55
Turkey ‡	85280	13725	161			2502	59
Ukraine ‖	20647	1257	61	54.5	56.0	295	14
United Kingdom, England	52823	6647	126	60.4	63.3	1663	31
United Kingdom, Northern Ireland	1911	203	106	60.3	63.2	35	18
United Kingdom, Scotland	5448	624	115	59.3	61.7	149	27
United Kingdom, Wales	3132	423	135	60.8	63.3	125	40
All countries	530138	80389	152	64.8	68.0	17626	37

DM = diabetes mellitus as primary renal disease
When cells are left empty, the data are unavailable and could not be used for the calculation of the summary data
§ The incidence is underestimated by approximately 2% due to one haemodialysis centre not submitting data
‖ Patients younger than 18 years of age are not reported
** Patients younger than 20 years of age are not reported*
† Data include dialysis patients only
¶ Data on primary renal disease are available for dialysis patients only (N=2492, 99.1% of total)
‡ Data on DM are extrapolated from data of 6821 patients (49.7% of total)

Figure A.1.1

Incidence per million population by country / region, unadjusted

at day 1

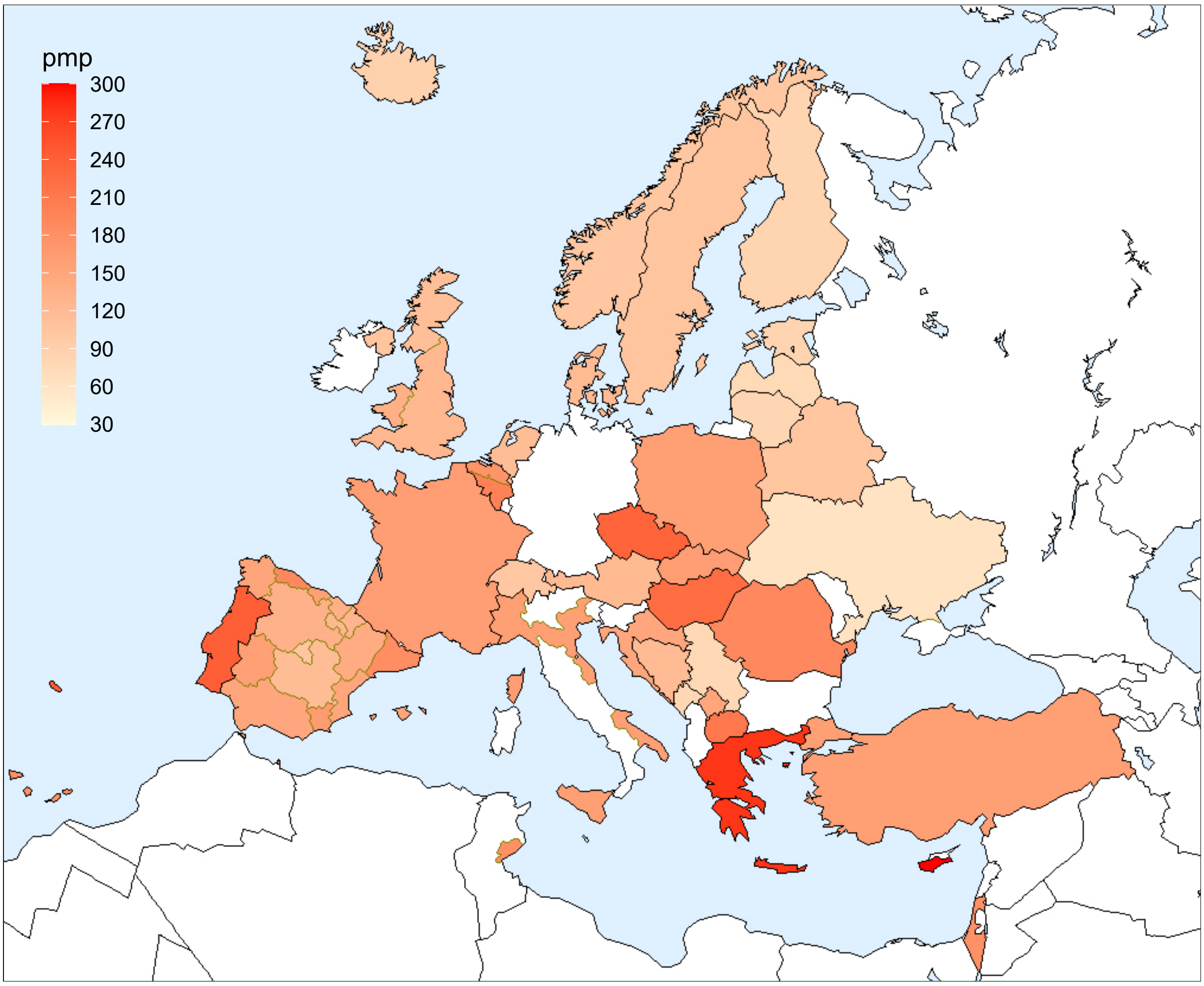

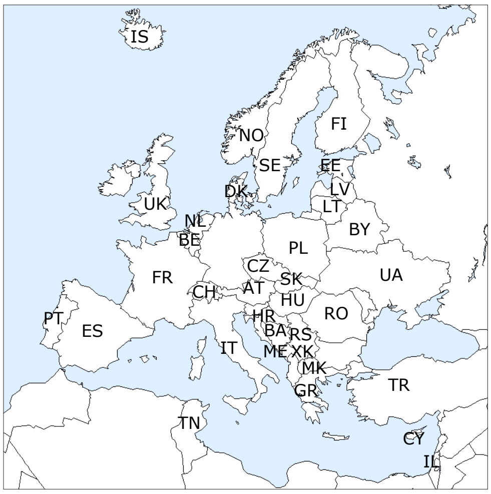

AT	Austria	LT	Lithuania
BA	Bosnia and Herzegovina	LV	Latvia
BE	Belgium	ME	Montenegro
BY	Belarus	MK	North Macedonia
CH	Switzerland	NL	the Netherlands
CY	Cyprus	NO	Norway
CZ	Czech Republic	PL	Poland
DK	Denmark	PT	Portugal
EE	Estonia	RO	Romania
ES	Spain	RS	Serbia
FI	Finland	SE	Sweden
FR	France	SK	Slovakia
GR	Greece	TN	Tunisia
HR	Croatia	TR	Turkey
HU	Hungary	UA	Ukraine
IL	Israel	UK	United Kingdom
IS	Iceland	XK	Kosovo
IT	Italy		

Detailed information on the contents of this figure can be found in table B.2.2 for registries providing individual patient data, and in table C.2.2 for registries providing aggregated data.

Figure A.1.2
Incidence per million population by country / region, unadjusted and adjusted
at day 1

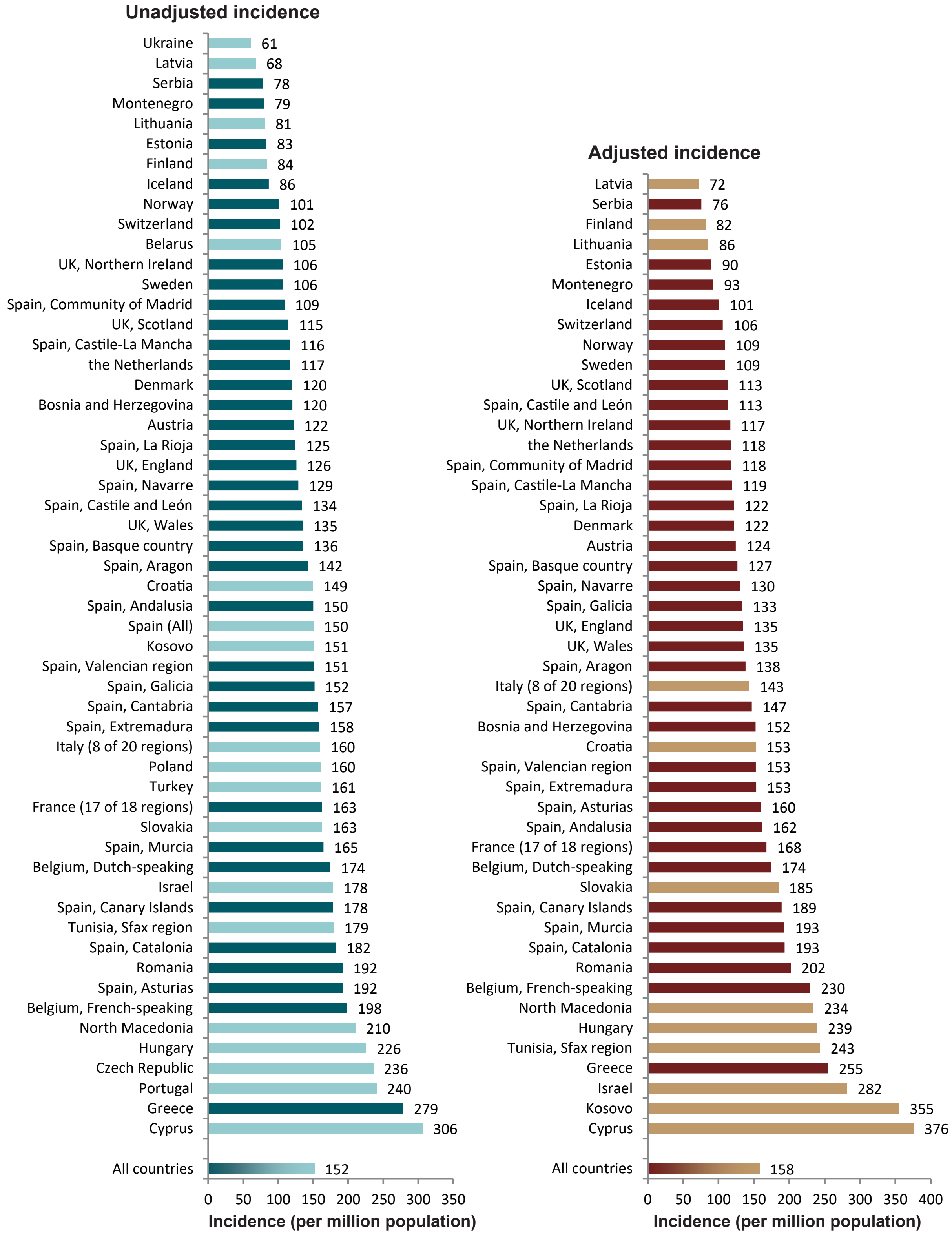

Registries providing individual patient data are shown as dark bars, and registries providing aggregated data as light bars.

Detailed information on the contents of this figure can be found in tables B.2.2 and B.2.4 for registries providing individual patient data, and in tables C.2.2 and C.2.4 for registries providing aggregated data.

Adjustment of the incidence was performed by standardising the incidence to the age and sex distribution of the EU27 population.

Figure A.1.3
Age, sex, and primary renal disease distribution by type of data provided, unadjusted
at day 1

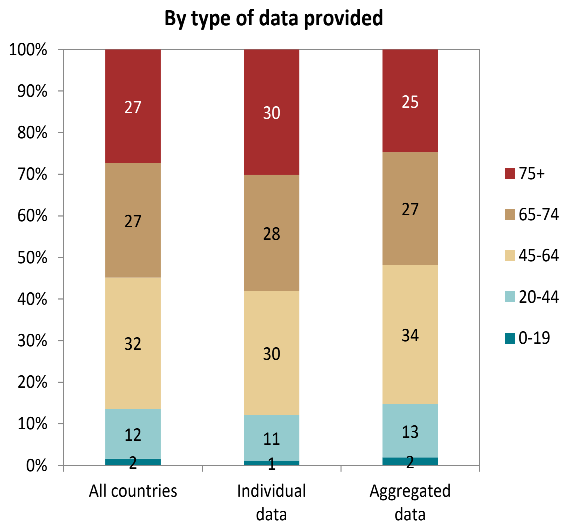

This figure summarizes the data presented in tables B.2.1 and C.2.1.

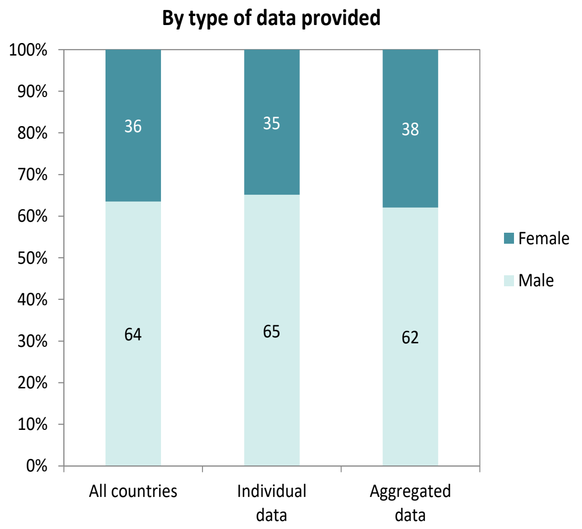

This figure summarizes the data presented in tables B.2.3 and C.2.3.

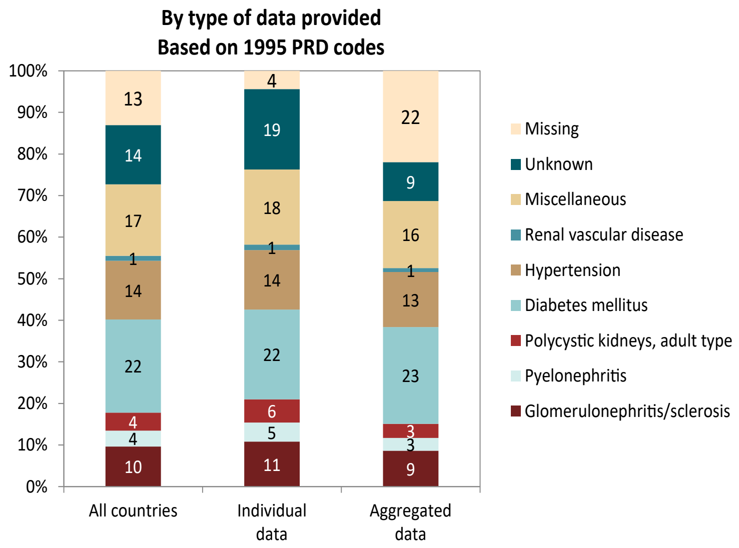

This figure summarizes the data presented in tables B.2.5.A and C.2.5.

Figure A.1.3 (continued)

Age, sex, and primary renal disease distribution by type of data provided, unadjusted

at day 1

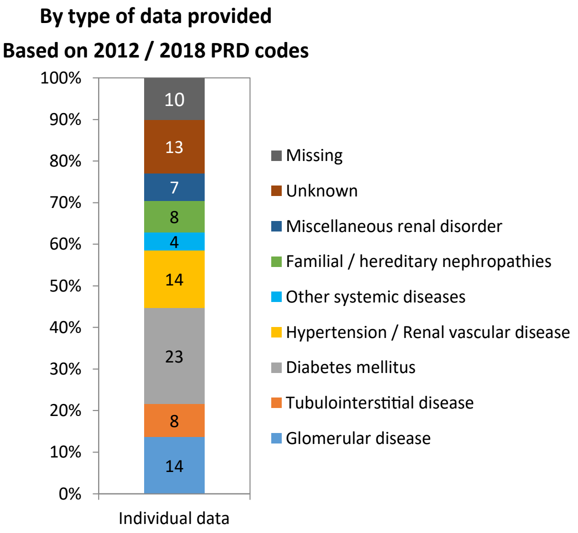

This figure summarizes the data presented in table B.2.5.B.

Figure A.1.4

Treatment modality distribution by type of data provided, age, sex and primary renal disease, unadjusted

at day 1

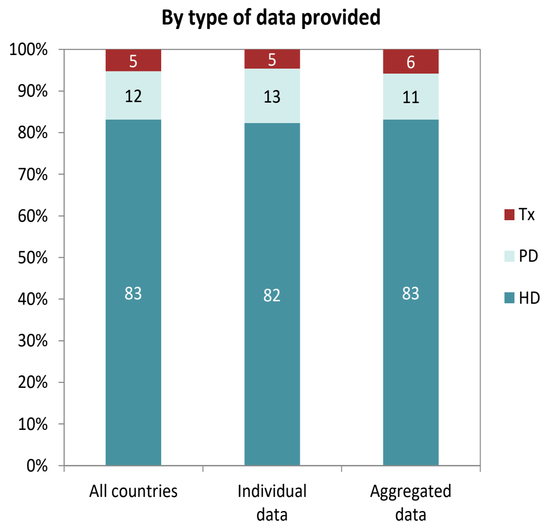

This figure summarizes the data presented in tables B.2.10 and C.2.8.

The figures below are only based on data from registries providing individual patient data.

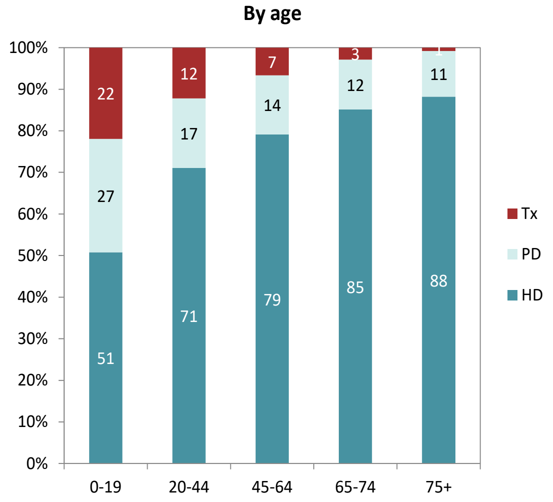

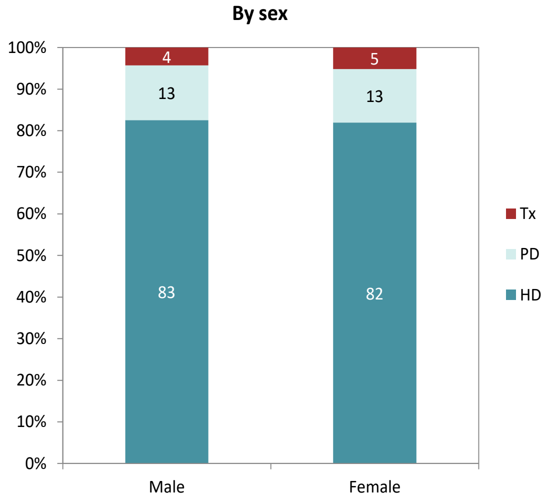

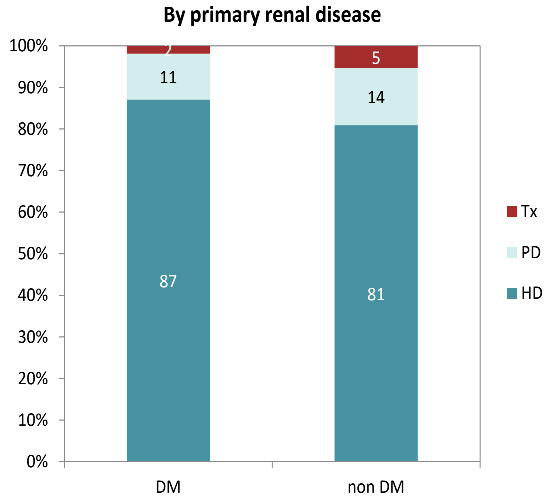

These figures summarize the data presented in table B.2.11.

Abbreviations used: HD: haemodialysis; PD: peritoneal dialysis; Tx: transplant; DM: diabetes mellitus

Figure A.2.1
Treatment modality distribution by type of data provided, age, sex and primary renal disease, unadjusted
at day 91

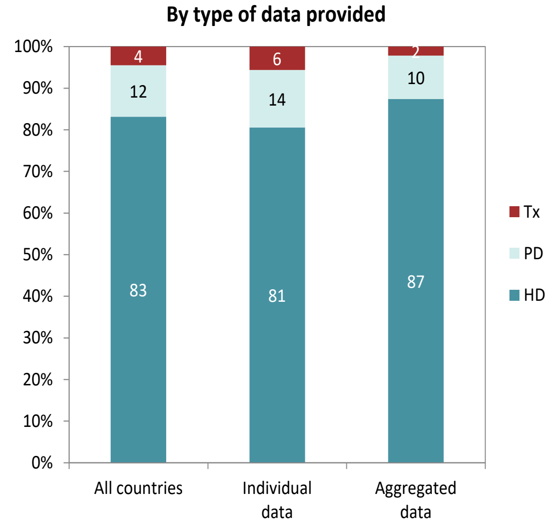

This figure summarizes the data presented in tables B.3.10 and C.3.8.

The figures below are only based on data from registries providing individual patient data.

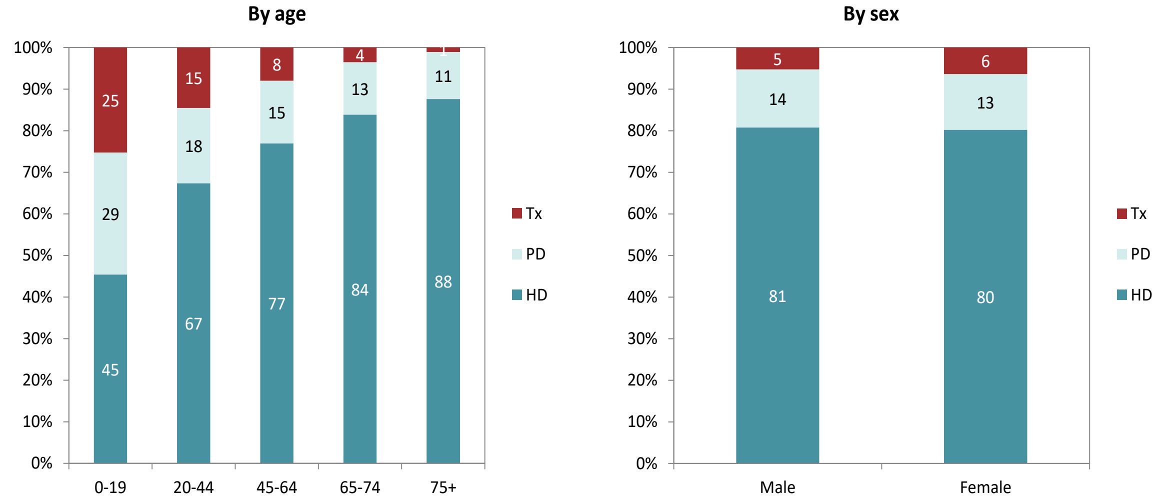

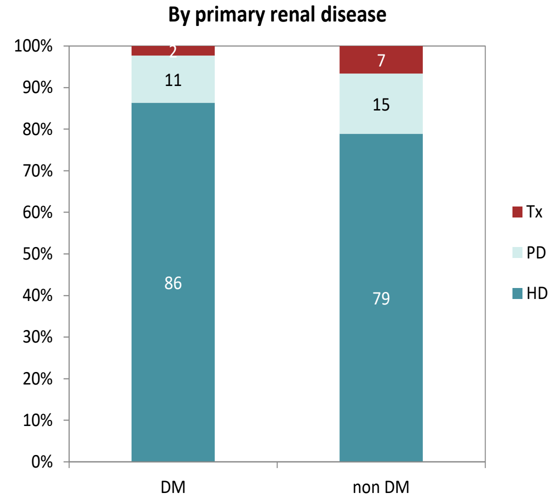

These figures summarize the data presented in table B.3.11.

Abbreviations used: HD: haemodialysis; PD: peritoneal dialysis; Tx: transplant; DM: diabetes mellitus

Table A.3.1
Summary data on the prevalence of KRT on 31 December 2022, unadjusted

	General population covered by the registry in thousands	Prevalent patients on KRT in 2022					
		All N	All Pmp	Mean Age	Median Age	DM N	DM Pmp
Austria $	8799	8950	1017	62.4	63.9	1655	188
Belarus ||	8490	4108	484			461	54
Belgium, Dutch-speaking *	6749	8702	1289	66.6	68.5	1413	209
Belgium, French-speaking *	4931	7083	1436	66.2	68.0	1233	250
Bosnia and Herzegovina	3531	2441	691	60.3	62.0	451	128
Croatia &	3162	2018	638	66.8	70.0	513	162
Czech Republic	10611	11157	1051				
Denmark	5903	5848	991	59.6	60.9	991	168
Estonia	1349	1136	842	60.3	61.6	205	152
Finland	5564	5240	942	60.1	62.6	1256	226
France (17 of 18 regions)	67614	93486	1383	63.5	65.7	15364	227
Greece	10437	15440	1479	66.4	68.4	2729	261
Hungary	9689	9485	979	59.4	61.0	1964	203
Iceland	382	312	817	57.5	58.6	41	107
Israel &	9557	6963	729	68.0	70.4	3213	336
Italy (8 of 20 regions)	27261	31758	1165	63.6	65.6	3329	122
Kosovo ||	1688	1044	619	59.3	62.0	284	168
Latvia	1670	1046	626	56.1	58.0	123	74
Lithuania	2806	2318	826				
Montenegro *	617	305	494	60.7	63.5	57	92
North Macedonia	1830	1731	946	59.8	62.0	309	169
Norway	5457	5442	997	60.4	62.3	708	130
Poland &	37827	20198	534			4434	117
Portugal ¤	10467	21198	2025	67.6		3754	552
Romania	19049	24054	1263	64.4	66.4	2189	115
Serbia	6383	6000	940	62.1	64.4	1056	165
Slovakia &	4362	3154	723	63.9	66.0	853	196
Spain (All)	47475	66856	1408	60.1	63.5	11102	234
Spain, Andalusia	8542	11412	1336	62.0	63.4	1936	227
Spain, Aragon	1343	2014	1500	66.0	67.8	357	266
Spain, Asturias	1006	1492	1483	65.1	67.0	267	265
Spain, Basque country	2213	2887	1304	62.5	64.6	399	180
Spain, Canary Islands	2199	3497	1590	63.1	64.1	905	412
Spain, Cantabria *	587	707	1205	64.0	65.1	113	193
Spain, Castile and León *	2373	3165	1334	66.2	67.2	518	218
Spain, Castile-La Mancha *	2069	2551	1233	64.3	65.0	443	214
Spain, Catalonia	7793	12027	1543	63.5	65.1	1833	235
Spain, Community of Madrid	6413	8194	1278	62.8	64.2	1392	217
Spain, Extremadura	1055	1512	1433	64.5	65.3	253	240
Spain, Galicia	2696	4017	1490	64.1	65.5	677	251
Spain, La Rioja	321	397	1237	62.9	63.6	52	162
Spain, Murcia	1532	2230	1456	63.2	64.3	369	241
Spain, Navarre *	668	933	1397	63.6	65.5	157	235
Spain, Valencian region	5098	7582	1487	64.2	66.0	1112	218
Sweden	10487	10573	1008	60.6	62.6	1774	169
Switzerland	8689	8885	1023	63.2	65.3	1278	147
the Netherlands	16993	18096	1065	61.3	63.3	2381	140
Tunisia, Sfax region &	1023	1043	1016	58.0	60.0	220	214
Turkey ‡	85280	86665	1016			6188	359
Ukraine ||	20647	7625	369	53.0	54.0	1283	62
United Kingdom, England	52309	55950	1070	58.3	59.8	10020	192
United Kingdom, Northern Ireland	1911	2099	1099	58.9	60.1	291	152
United Kingdom, Scotland	5448	5671	1041	57.7	59.5	935	172
United Kingdom, Wales	3132	3360	1073	58.4	59.5	626	200
All countries	529427	567440	1074	62.0	63.9	84683	191

DM = diabetes mellitus as primary renal disease
When cells are left empty, the data are unavailable and could not be used for the calculation of the summary data
$ The prevalence is underestimated by approximately 2% due to one haemodialysis centre not submitting data
|| Patients younger than 18 years of age are not reported
** Patients younger than 20 years of age are not reported*
& Data on prevalence include dialysis patients only
¤ Data on DM are extrapolated from data of 13759 patients (65.0% of total)
‡ Data on DM are extrapolated from data of 17506 patients (20.2% of total)

Figure A.3.1
Prevalence per million population by country / region, unadjusted
prevalent patients on December 31

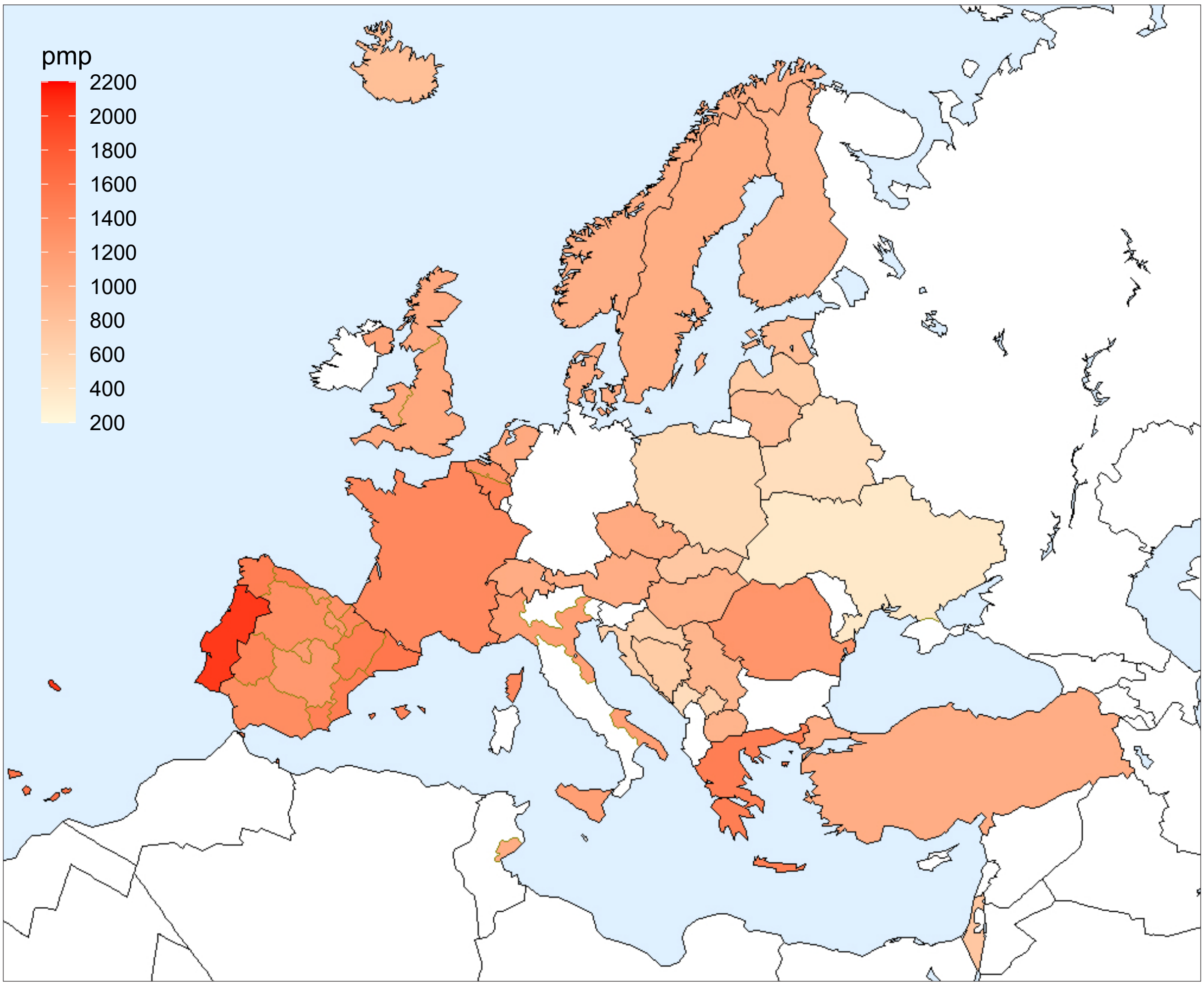

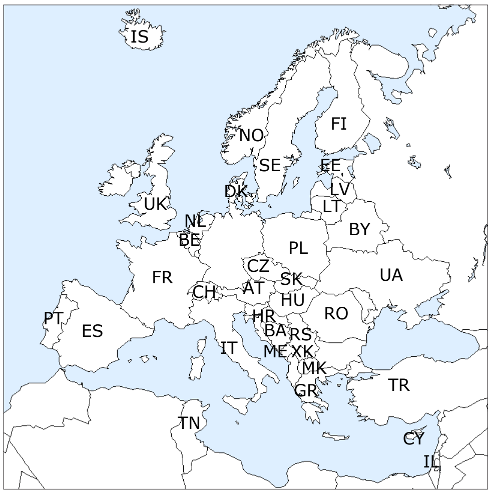

AT	Austria	LT	Lithuania
BA	Bosnia and Herzegovina	LV	Latvia
BE	Belgium	ME	Montenegro
BY	Belarus	MK	North Macedonia
CH	Switzerland	NL	the Netherlands
CY	Cyprus	NO	Norway
CZ	Czech Republic	PL	Poland
DK	Denmark	PT	Portugal
EE	Estonia	RO	Romania
ES	Spain	RS	Serbia
FI	Finland	SE	Sweden
FR	France	SK	Slovakia
GR	Greece	TN	Tunisia
HR	Croatia	TR	Turkey
HU	Hungary	UA	Ukraine
IL	Israel	UK	United Kingdom
IS	Iceland	XK	Kosovo
IT	Italy		

Detailed information on the contents of this figure can be found in table B.4.2 for registries providing individual patient data, and in table C.4.2 for registries providing aggregated data.

Figure A.3.2

Prevalence per million population by country / region, unadjusted and adjusted

prevalent patients on December 31

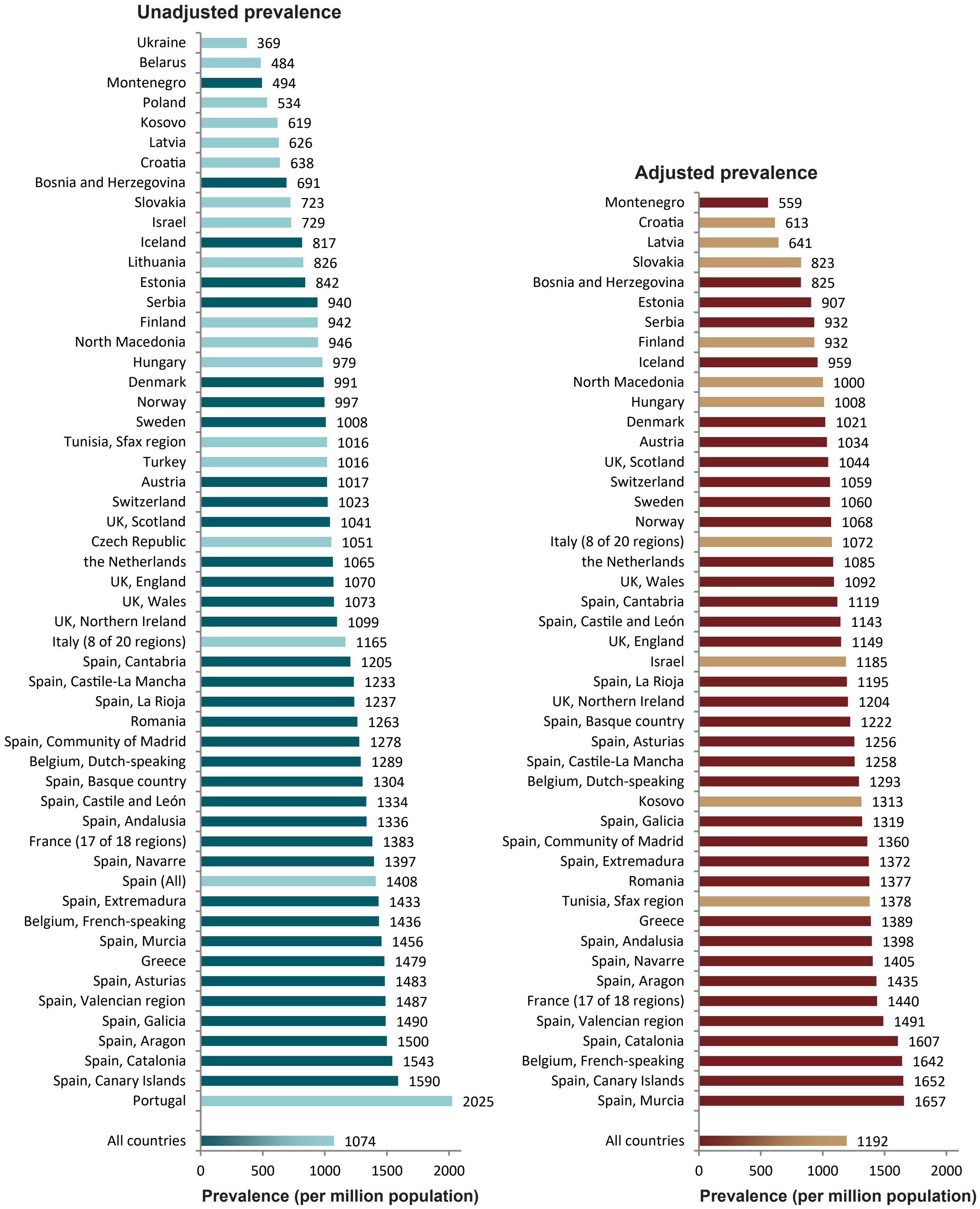

Registries providing individual patient data are shown as dark bars, and registries providing aggregated data as light bars.

Detailed information on the contents of this figure can be found in tables B.4.2 and B.4.4 for registries providing individual patient data, and in tables C.4.2 and C.4.4 for registries providing aggregated data.

Adjustment of the prevalence was performed by standardising the prevalence to the age and sex distribution of the EU27 population.

Figure A.3.3
Age, sex, and primary renal disease distribution by type of data provided, unadjusted
prevalent patients on December 31

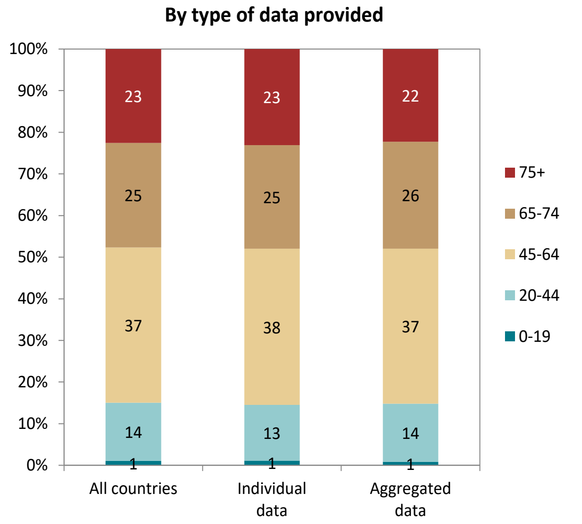

This figure summarizes the data presented in tables B.4.1 and C.4.1.

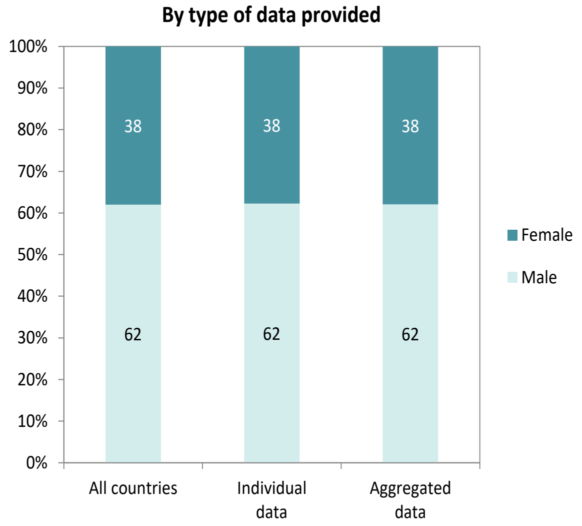

This figure summarizes the data presented in tables B.4.3 and C.4.3.

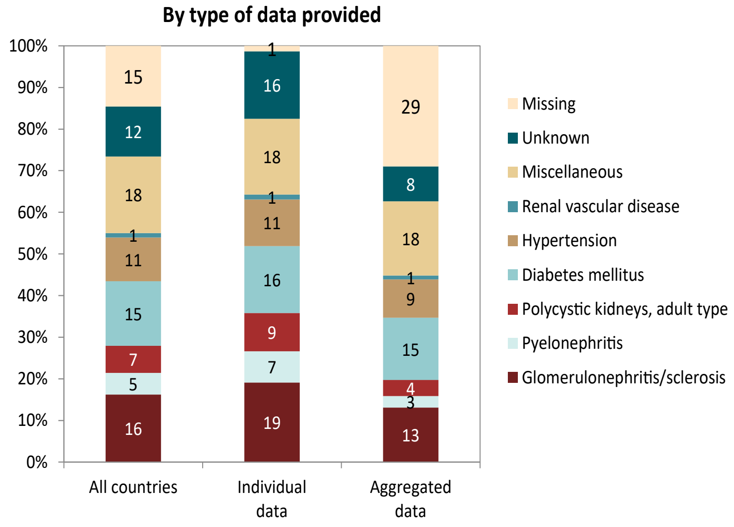

This figure summarizes the data presented in tables B.4.5 and C.4.5.

Figure A.3.4

Treatment modality distribution by type of data provided, age, sex and primary renal disease, unadjusted

prevalent patients on December 31

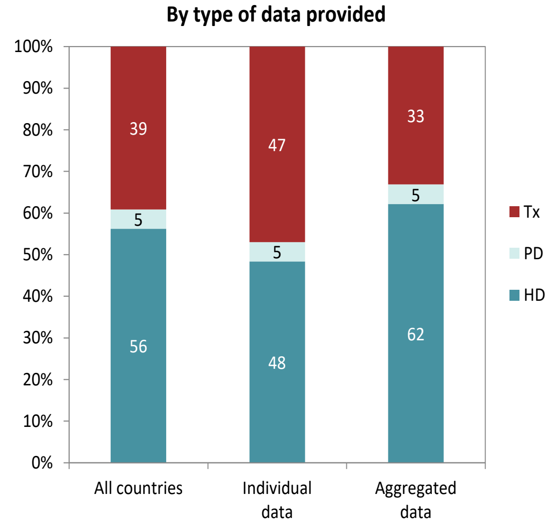

This figure summarizes the data presented in tables B.4.10 and C.4.8.

The figures below are only based on data from registries providing individual patient data.

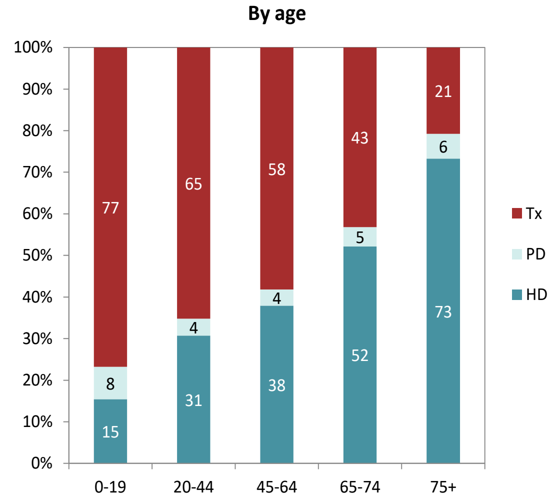

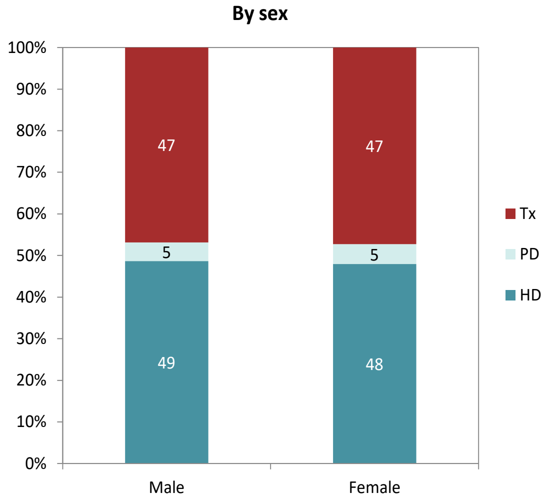

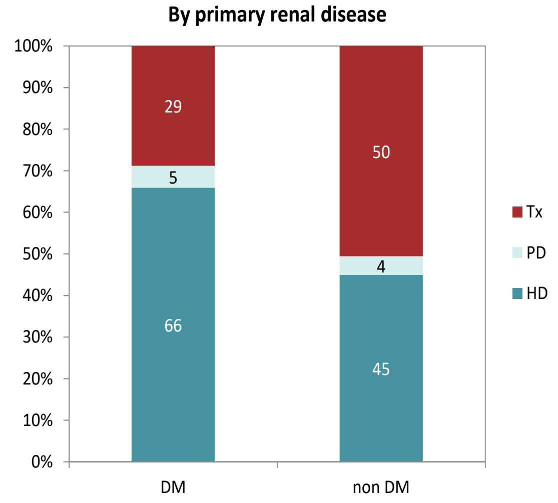

These figures summarize the data presented in table B.4.11.

Abbreviations used: HD: haemodialysis; PD: peritoneal dialysis; Tx: transplant; DM: diabetes mellitus

Figure A.4.1
Kidney transplant counts and per million population by country / region, unadjusted

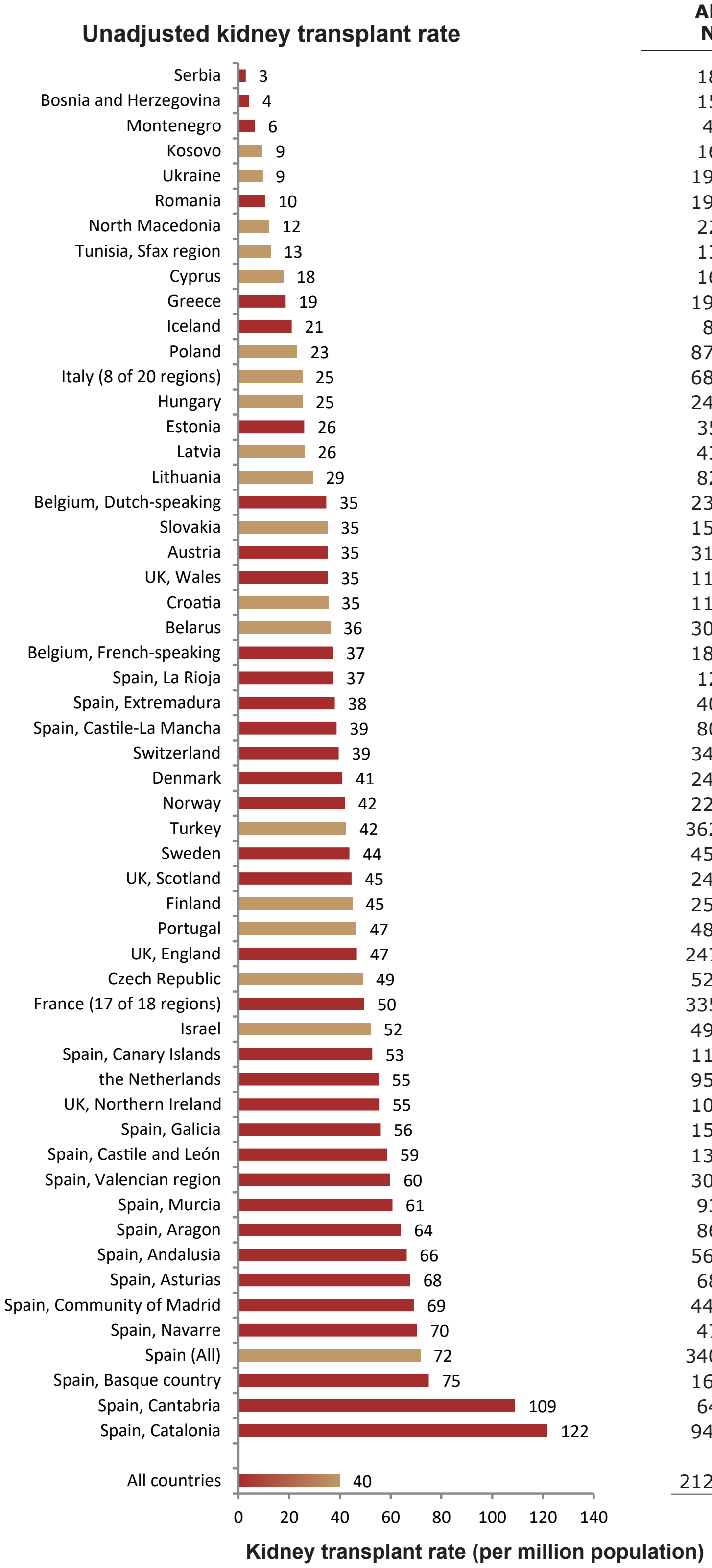

Country / region	All N	% Deceased donor	% Living donor	% Unknown donor type
Serbia	18	11	89	0
Bosnia and Herzegovina	15	20	73	7
Montenegro	4	25	75	0
Kosovo	16	0	100	0
Ukraine	196	57	43	0
Romania	199	67	33	0
North Macedonia	22	45	55	0
Tunisia, Sfax region	13	0	100	0
Cyprus	16	50	50	0
Greece	194	58	42	0
Iceland	8	63	38	0
Poland	874	92	8	0
Italy (8 of 20 regions)	685	79	16	6
Hungary	244	77	23	0
Estonia	35	89	11	0
Latvia	43	93	7	0
Lithuania	82	94	6	0
Belgium, Dutch-speaking	234	79	11	9
Slovakia	153	80	20	0
Austria	310	85	15	0
UK, Wales	110	63	37	0
Croatia	112			100
Belarus	307	98	2	0
Belgium, French-speaking	184	91	8	1
Spain, La Rioja	12	83	17	0
Spain, Extremadura	40	95	5	0
Spain, Castile-La Mancha	80	100	0	0
Switzerland	343	67	32	0
Denmark	242	68	32	0
Norway	229	81	19	0
Turkey	3621	8	92	0
Sweden	459	79	21	0
UK, Scotland	243	61	36	2
Finland	250	83	17	0
Portugal	487	88	12	0
UK, England	2474	76	24	1
Czech Republic	520	92	8	0
France (17 of 18 regions)	3352	85	15	0
Israel	498	30	68	2
Spain, Canary Islands	116	98	2	0
the Netherlands	959	48	52	0
UK, Northern Ireland	106	49	51	0
Spain, Galicia	151	86	14	0
Spain, Castile and León	139	94	6	0
Spain, Valencian region	305	97	3	0
Spain, Murcia	93	95	5	0
Spain, Aragon	86	91	9	0
Spain, Andalusia	566	93	7	0
Spain, Asturias	68	94	6	0
Spain, Community of Madrid	443	89	11	0
Spain, Navarre	47	94	6	0
Spain (All)	3404	90	10	0
Spain, Basque country	166	88	12	0
Spain, Cantabria	64	97	3	0
Spain, Catalonia	949	81	19	0
All countries	21261	66	33	1

Registries providing individual patient data are shown as red bars, and registries providing aggregated data as orange bars.

Detailed information on the contents of this figure can be found in tables B.5.1 and B.5.2 for registries providing individual patient data, and in tables C.5.1 and C.5.2 for registries providing aggregated data.

Figure A.4.2
Kidney transplants per million population by donor type and by country / region, unadjusted

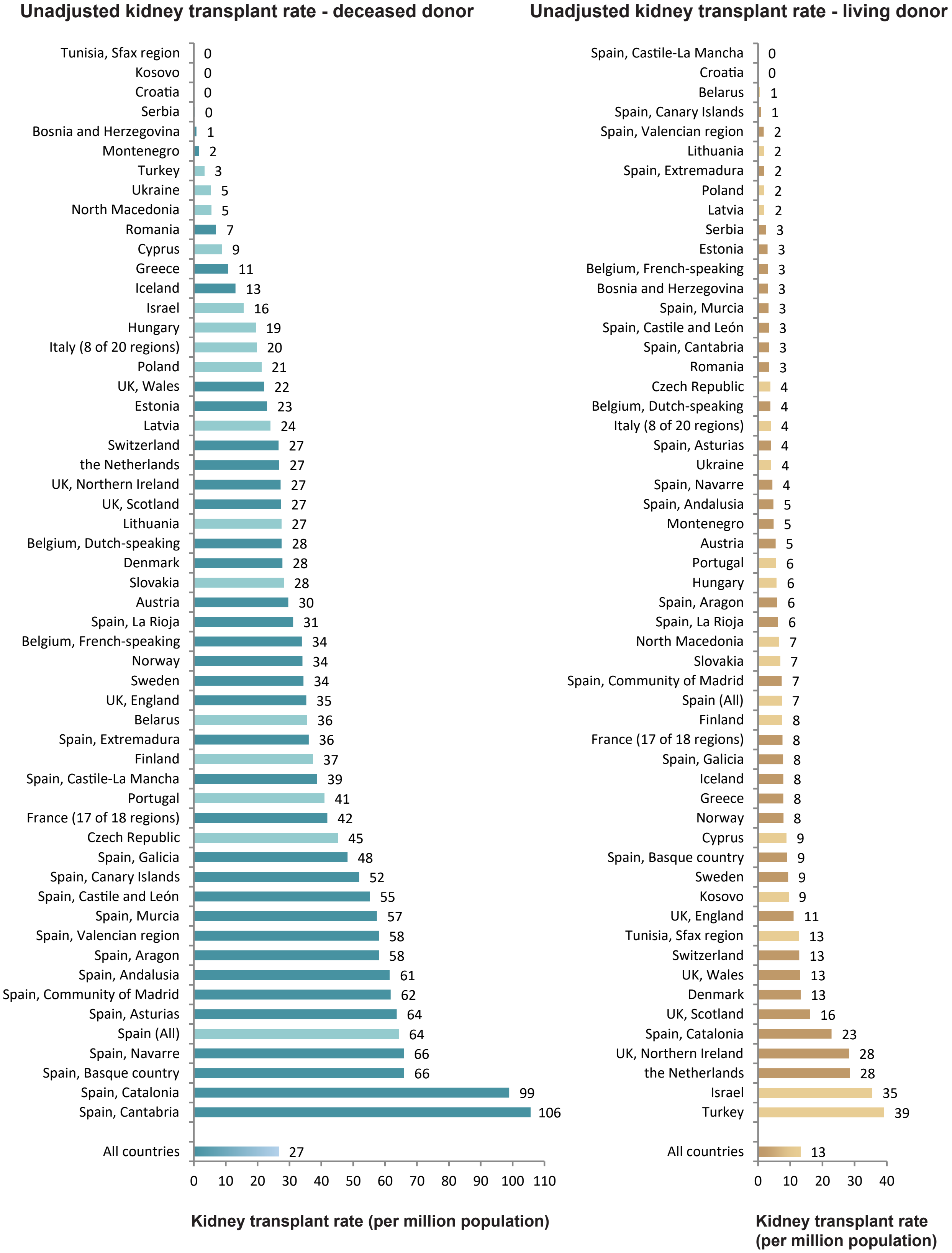

Registries providing individual patient data are shown as dark bars, and registries providing aggregated data as light bars.

Detailed information on the contents of this figure can be found in table B.5.2 for registries providing individual patient data, and in table C.5.2 for registries providing aggregated data.

Figure A.4.3
Donor type distribution by type of data provided, unadjusted

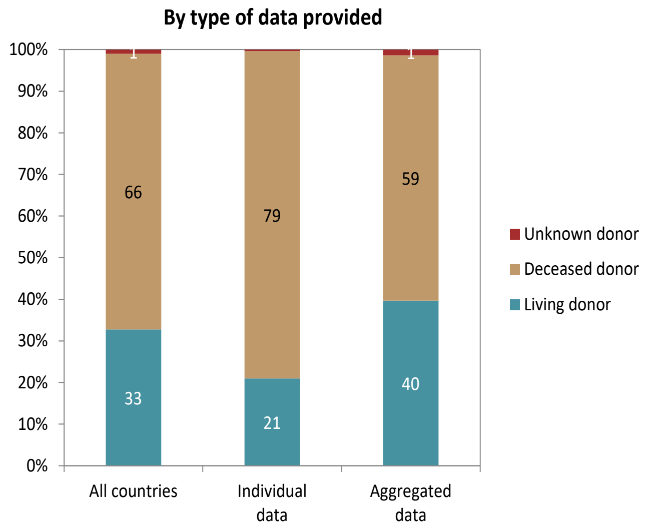

This figure summarizes the data presented in table B.5.1 for registries providing individual patient data, and in table C.5.1 for registries providing aggregated data.

Table A.5.1

One-, two- and five-year survival probabilities by treatment modality and cohort

from day 1 of KRT / dialysis, or from day of transplant

	Survival probabilities as % (95% CI)				
	cohort 2013 - 2017			cohort 2016 - 2020	
	1 year	2 year	5 year	1 year	2 year
**Patient survival on KRT**					
Unadjusted	85.3 (85.1-85.5)	75.3 (75.1-75.5)	51.5 (51.2-51.7)	85.7 (85.6-85.9)	75.7 (75.5-75.9)
Adjusted †	88.2 (88.0-88.3)	79.3 (79.1-79.5)	54.2 (53.9-54.4)	88.3 (88.2-88.5)	79.2 (79.0-79.4)
**Patient survival on dialysis**					
Unadjusted	84.2 (84.0-84.4)	72.6 (72.4-72.8)	41.3 (41.0-41.5)	84.7 (84.5-84.9)	73.1 (72.9-73.3)
Adjusted †	86.5 (86.4-86.7)	76.3 (76.0-76.5)	46.7 (46.4-47.0)	87.1 (87.0-87.3)	76.9 (76.7-77.1)
**Patient survival after first kidney transplantation (deceased donor)**					
Unadjusted	96.3 (96.1-96.5)	94.1 (93.8-94.3)	85.1 (84.7-85.4)	96.1 (95.9-96.3)	93.3 (93.1-93.5)
Adjusted ††	98.1 (98.0-98.2)	97.0 (96.8-97.1)	91.9 (91.6-92.2)	98.1 (98.0-98.3)	96.8 (96.6-96.9)
**Graft survival after first kidney transplantation (deceased donor)**					
Unadjusted	91.0 (90.8-91.3)	87.8 (87.4-88.1)	76.2 (75.7-76.6)	91.1 (90.8-91.3)	87.3 (87.0-87.7)
Adjusted ††	93.1 (92.9-93.4)	90.5 (90.2-90.8)	80.9 (80.5-81.3)	93.4 (93.2-93.7)	90.6 (90.3-90.9)
**Patient survival after first kidney transplantation (living donor)**					
Unadjusted	98.8 (98.6-99.0)	98.0 (97.7-98.2)	94.2 (93.8-94.6)	98.8 (98.6-99.0)	97.9 (97.6-98.1)
Adjusted ††	99.1 (98.9-99.2)	98.4 (98.2-98.7)	95.3 (95.0-95.7)	99.1 (99.0-99.3)	98.4 (98.2-98.6)
**Graft survival after first kidney transplantation (living donor)**					
Unadjusted	96.5 (96.2-96.8)	95.0 (94.6-95.3)	88.1 (87.6-88.7)	96.7 (96.4-97.0)	95.1 (94.7-95.4)
Adjusted ††	96.4 (96.1-96.8)	94.8 (94.4-95.2)	87.7 (87.1-88.3)	96.6 (96.2-96.9)	94.9 (94.5-95.3)

† Analyses were adjusted using fixed values: age (67 years), sex (63% male) and PRD (24% diabetes mellitus, 19% hypertension/renal vascular disease, 11% glomerulonephritis and 46% other causes)

†† Analyses were adjusted using fixed values: age (50 years), sex (63% male) and PRD (14% diabetes mellitus, 10% hypertension/renal vascular disease, 23% glomerulonephritis and 53% other causes)

This table summarizes the survival data presented in the tables B.6.1 to B.6.16, and is based on data from the following registries providing individual patient data: Austria, Belgium (Dutch-speaking), Belgium (French-speaking), Bosnia and Herzegovina, Denmark, Estonia, France, Greece, Iceland, Norway, Spain (Andalusia), Spain (Aragon), Spain (Asturias), Spain (Basque country), Spain (Canary Islands), Spain (Cantabria), Spain (Castile and León), Spain (Castile-La Mancha), Spain (Catalonia), Spain (Community of Madrid), Spain (Extremadura), Spain (Galicia), Spain (Murcia), Spain (Navarre), Spain (Valencian Region), Sweden, the Netherlands, United Kingdom (England/Northern Ireland/ Wales) and United Kingdom (Scotland).

Figure A.5.1

Patient survival by modality: Incident dialysis patients (cohort 2013-2017)

from day 91, unadjusted

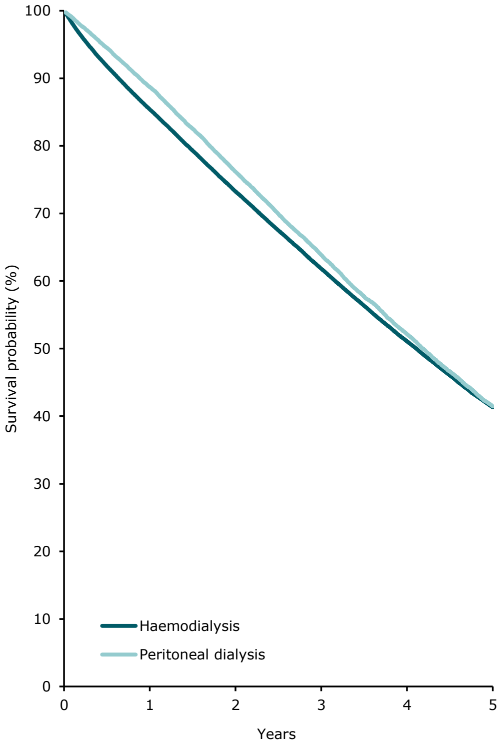

Figure A.5.2

Patient survival by donor type: Patients receiving a first kidney transplant (cohort 2013-2017)

from day of transplant, unadjusted

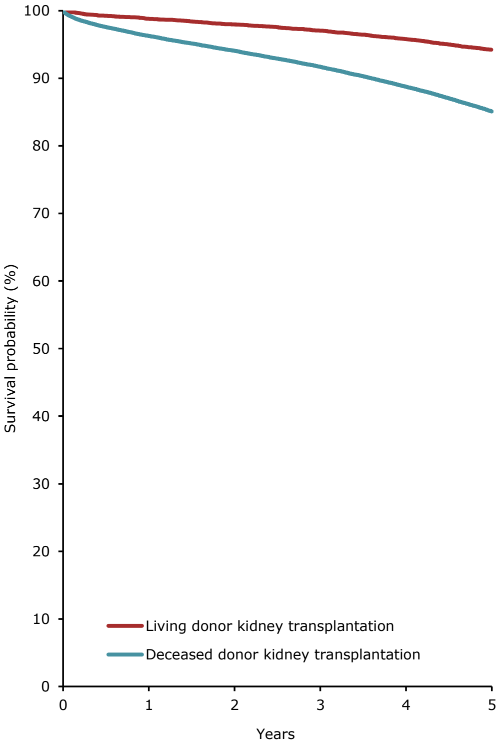

These figures are based on patients starting dialysis or receiving a kidney transplant between 2013 and 2017 from the following registries providing individual patient data: Austria, Belgium (Dutch-speaking), Belgium (French-speaking), Bosnia and Herzegovina, Denmark, Estonia, France, Greece, Iceland, Norway, Spain (Andalusia), Spain (Aragon), Spain (Asturias), Spain (Basque country), Spain (Canary Islands), Spain (Cantabria), Spain (Castile and León), Spain (Castile-La Mancha), Spain (Catalonia), Spain (Community of Madrid), Spain (Extremadura), Spain (Galicia), Spain (Murcia), Spain (Navarre), Spain (Valencian Region), Sweden, the Netherlands, United Kingdom (England/Northern Ireland/Wales) and United Kingdom (Scotland).

Figure A.6.1
Expected remaining years of life in the general population and in prevalent dialysis and kidney transplant patients (cohort 2018-2022)
by age

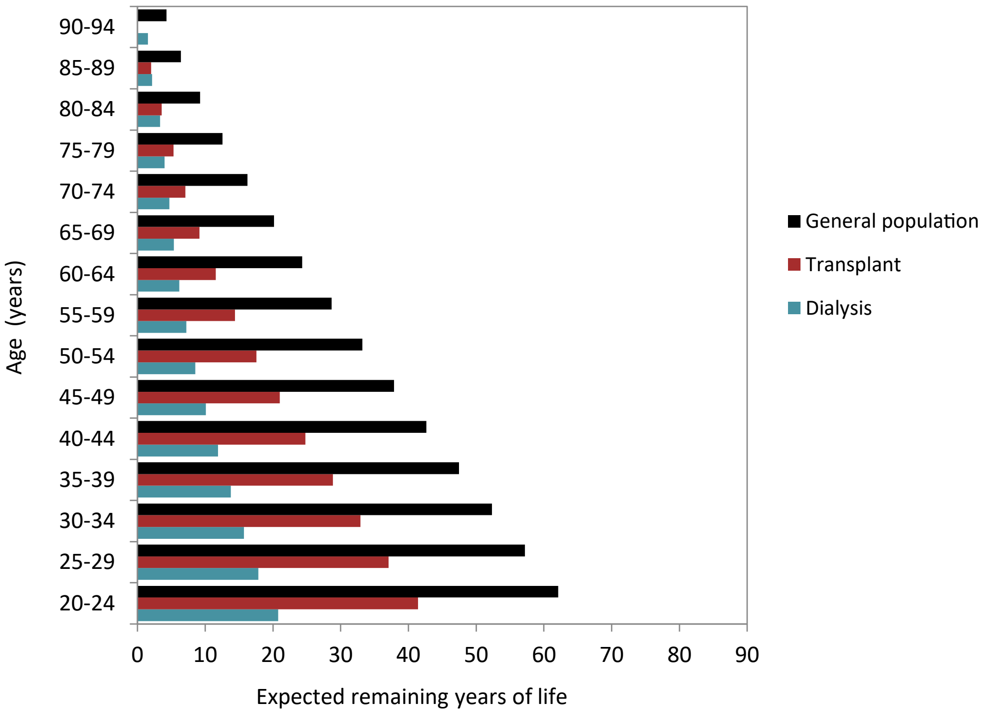

This figure summarizes the data presented in table B.7.1, and is based on data from the following registries providing individual patient data: Austria, Belgium (Dutch-speaking), Belgium (French-speaking), Bosnia and Herzegovina, Denmark, Estonia, France, Greece, Iceland, Norway, Spain (Andalusia), Spain (Aragon), Spain (Asturias), Spain (Basque country), Spain (Canary Islands), Spain (Cantabria), Spain (Castille and León), Spain (Castille-La Mancha), Spain (Catalonia), Spain (Community of Madrid), Spain (Extremadura), Spain (Galicia), Spain (Murcia), Spain (Navarre), Spain (Valencian Region), Sweden, the Netherlands, United Kingdom (England/Northern Ireland/Wales) and United Kingdom (Scotland).

Figure A.7.1

Sex distribution by country / region, unadjusted

incident patients at day 1

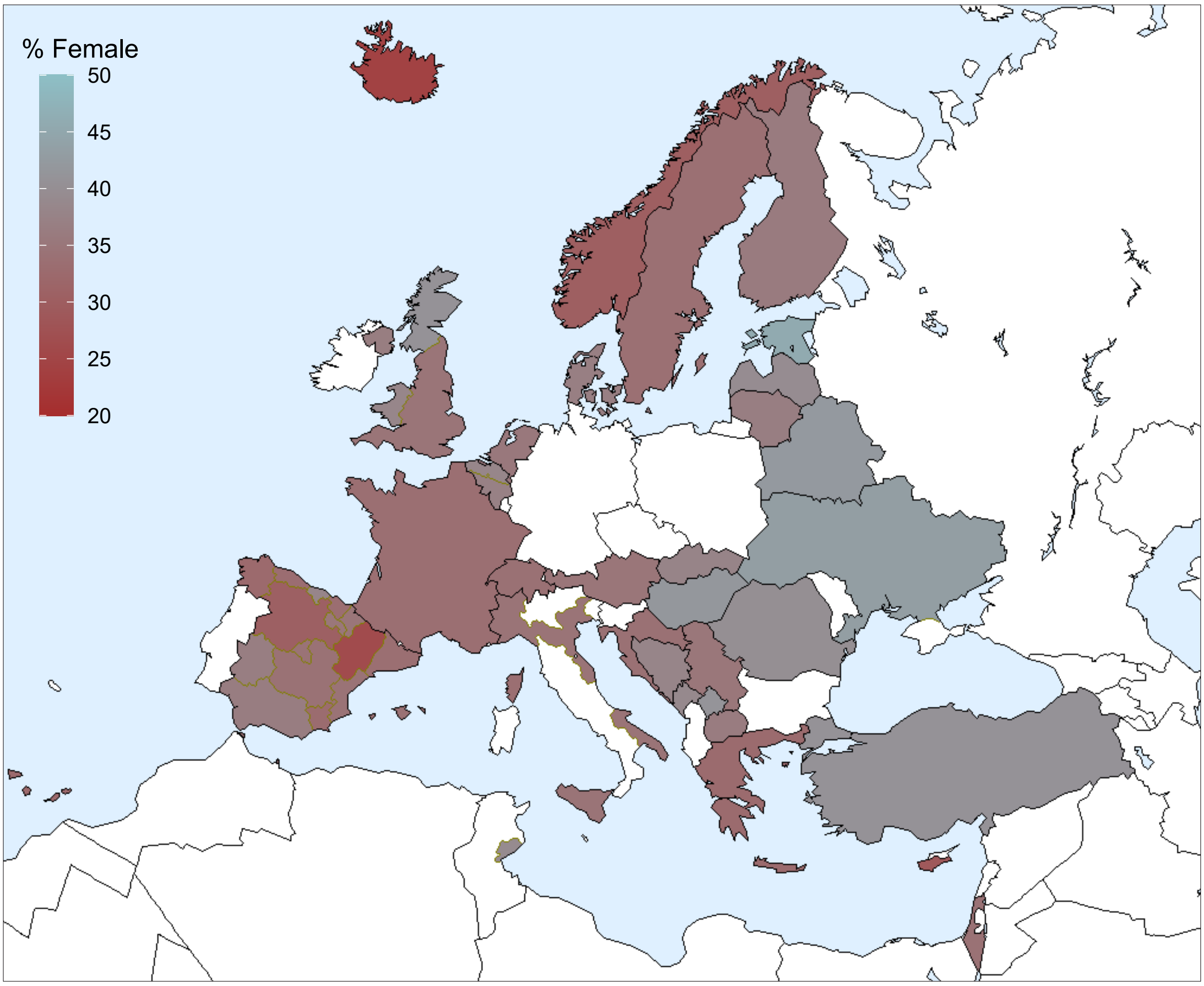

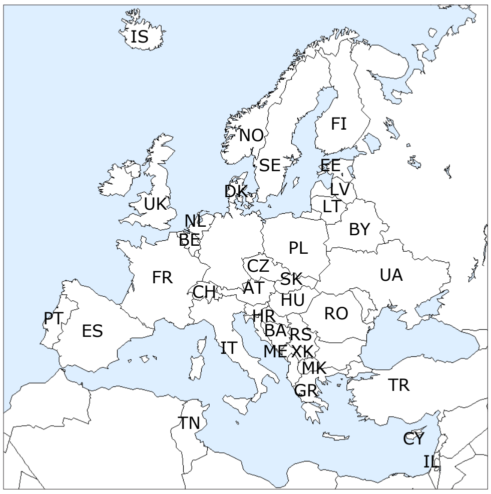

AT	Austria	LT	Lithuania
BA	Bosnia and Herzegovina	LV	Latvia
BE	Belgium	ME	Montenegro
BY	Belarus	MK	North Macedonia
CH	Switzerland	NL	the Netherlands
CY	Cyprus	NO	Norway
CZ	Czech Republic	PL	Poland
DK	Denmark	PT	Portugal
EE	Estonia	RO	Romania
ES	Spain	RS	Serbia
FI	Finland	SE	Sweden
FR	France	SK	Slovakia
GR	Greece	TN	Tunisia
HR	Croatia	TR	Turkey
HU	Hungary	UA	Ukraine
IL	Israel	UK	United Kingdom
IS	Iceland	XK	Kosovo
IT	Italy		

Detailed information on the contents of this figure can be found in table B.2.1 for registries providing individual patient data, and in table C.2.1 for registries providing aggregated data.

Figure A.7.2
Incidence per million population by sex, unadjusted
at day 1

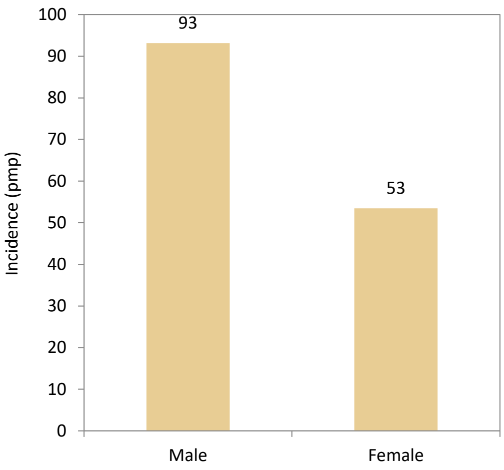

This figure summarizes the data presented in tables B.2.1 and C.2.1

Figure A.7.3

Age, primary renal disease and treatment modality distribution by sex in incident patients, unadjusted

at day 1

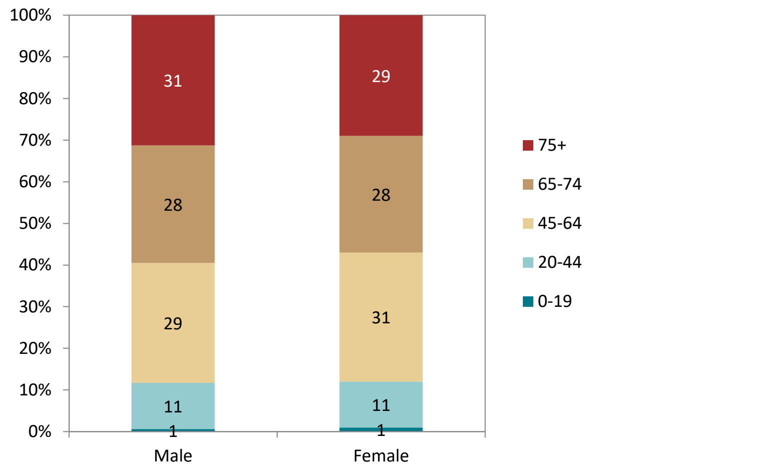

This figure summarizes the data presented in table B.2.1

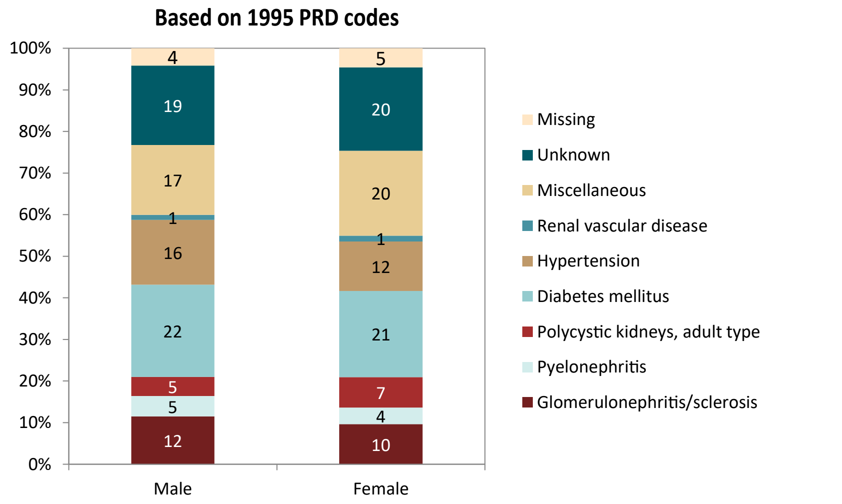

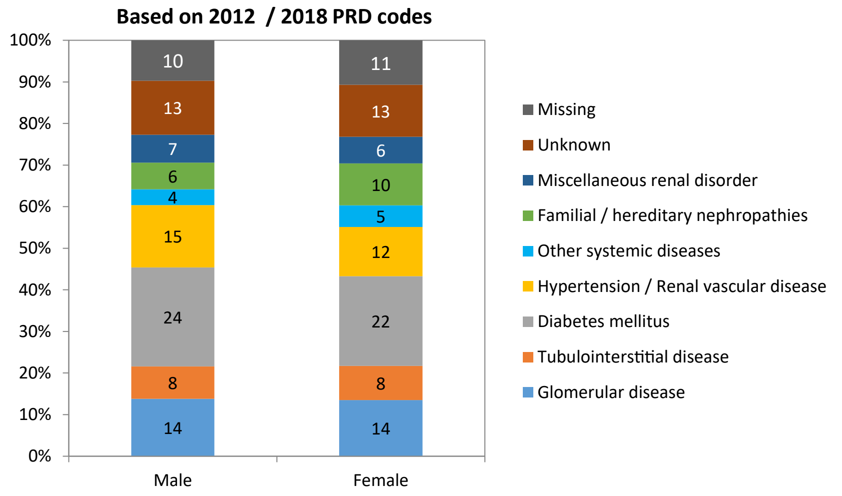

Figure A.7.3 (continued)

Age, primary renal disease and treatment modality distribution by sex in incident patients, unadjusted

at day 1

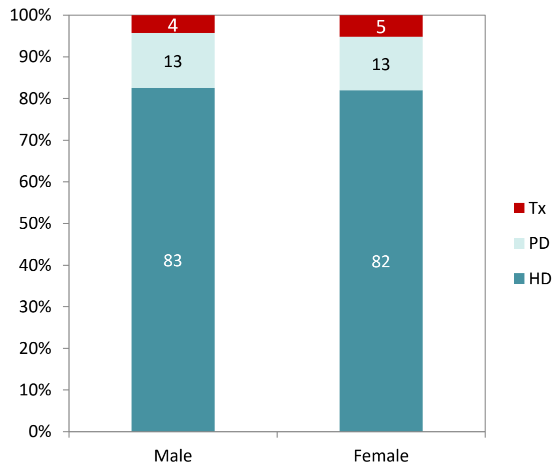

This figure summarizes the data presented in table B.2.11

Abbreviations used: HD: haemodialysis; PD: peritoneal dialysis; Tx: transplant

Figure A.7.4

Sex distribution by country / region, unadjusted

prevalent patients on December 31

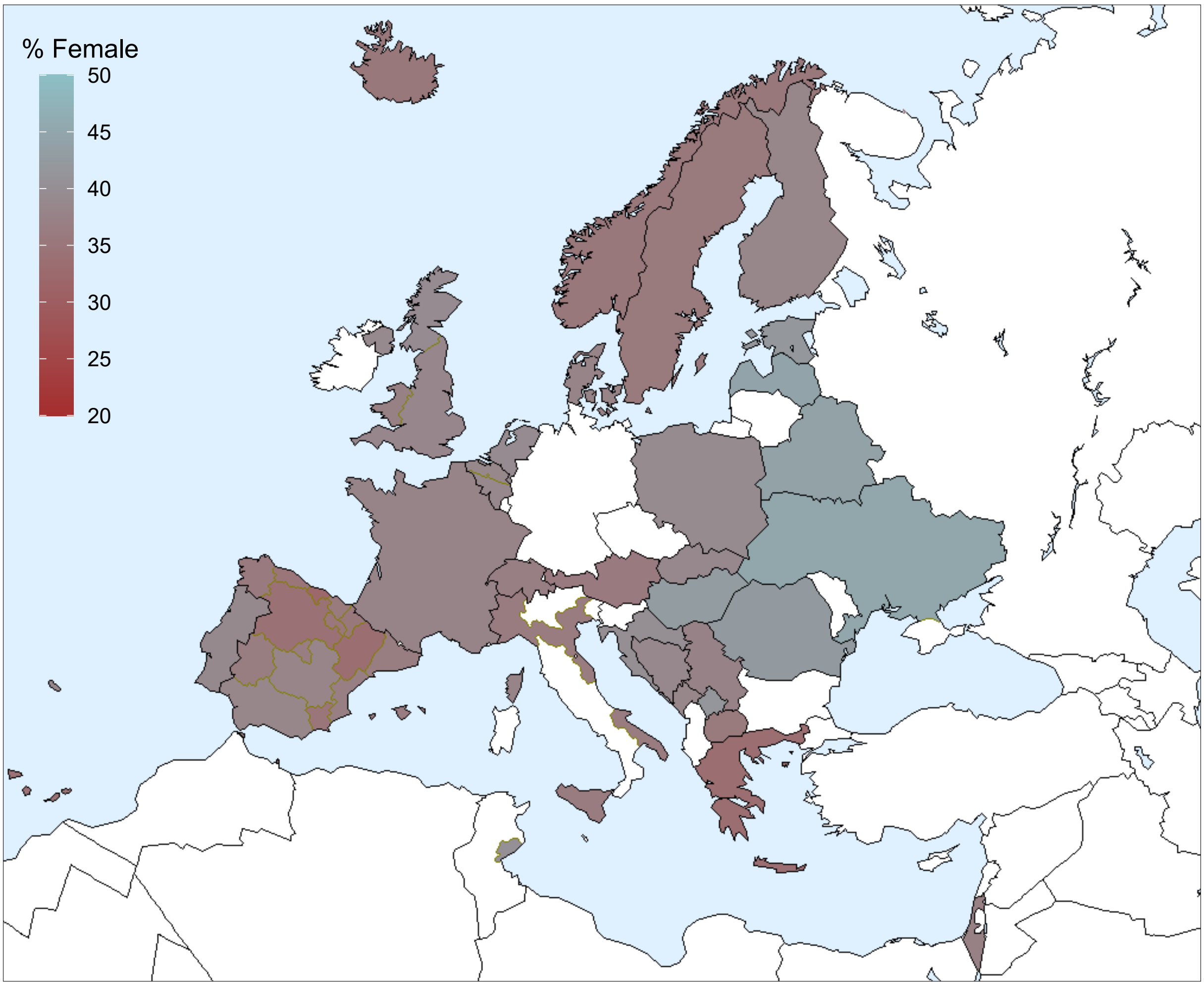

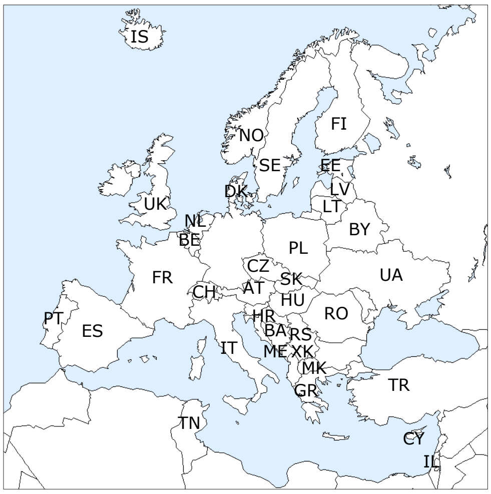

AT	Austria	LT	Lithuania
BA	Bosnia and Herzegovina	LV	Latvia
BE	Belgium	ME	Montenegro
BY	Belarus	MK	North Macedonia
CH	Switzerland	NL	the Netherlands
CY	Cyprus	NO	Norway
CZ	Czech Republic	PL	Poland
DK	Denmark	PT	Portugal
EE	Estonia	RO	Romania
ES	Spain	RS	Serbia
FI	Finland	SE	Sweden
FR	France	SK	Slovakia
GR	Greece	TN	Tunisia
HR	Croatia	TR	Turkey
HU	Hungary	UA	Ukraine
IL	Israel	UK	United Kingdom
IS	Iceland	XK	Kosovo
IT	Italy		

Detailed information on the contents of this figure can be found in table B.4.1 for registries providing individual patient data, and in table C.4.1 for registries providing aggregated data.

Figure A.7.5
Prevalence per million population by sex, unadjusted
prevalent patients on December 31

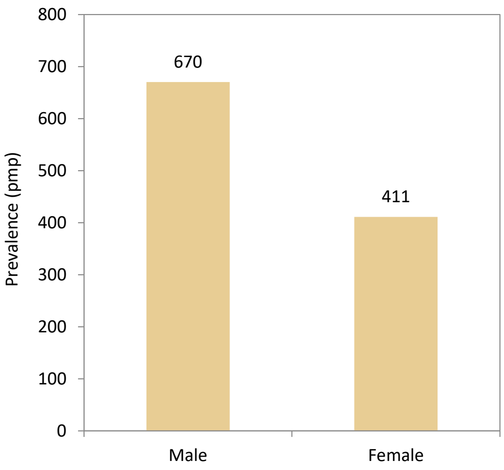

This figure summarizes the data presented in tables B.4.1 and C.4.1

Figure A.7.6

Age, primary renal disease and treatment modality distribution by sex in prevalent patients, unadjusted

prevalent patients on December 31

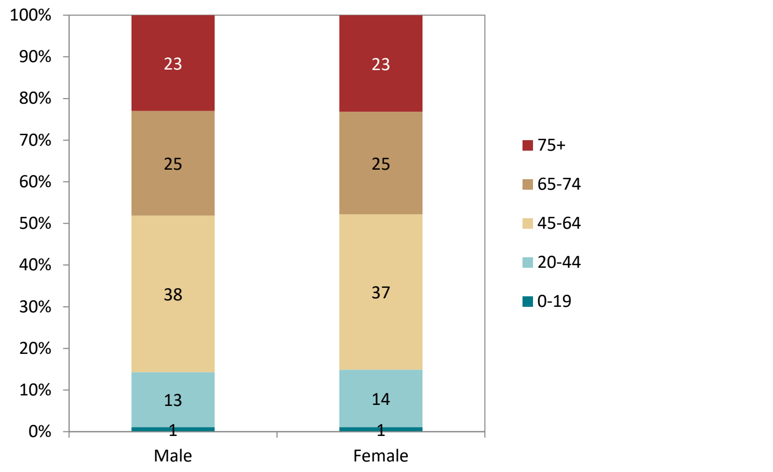

This figure summarizes the data presented in table B.4.1

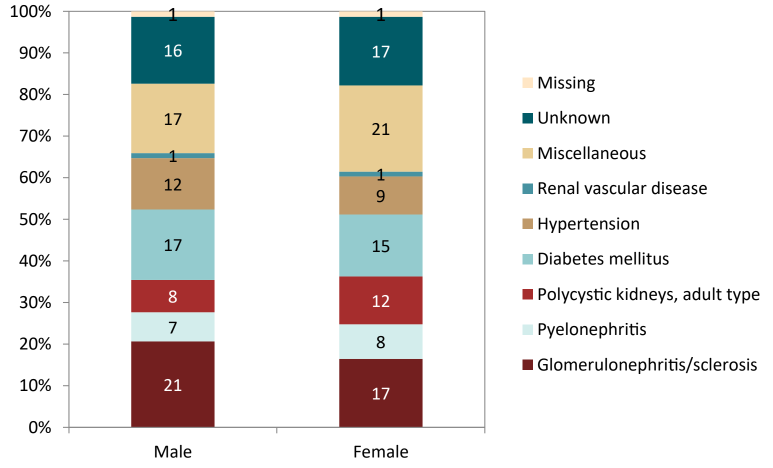

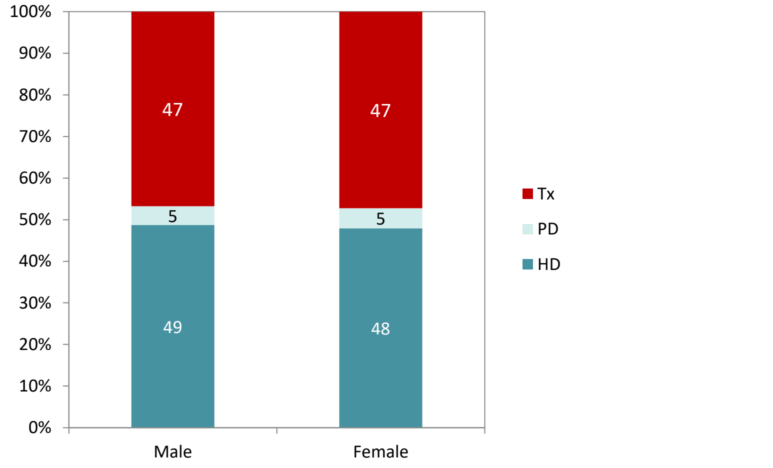

This figure summarizes the data presented in table B.4.11

Abbreviations used: HD: haemodialysis; PD: peritoneal dialysis; Tx: transplant

Figure A.7.7
Kidney transplants per million population by sex

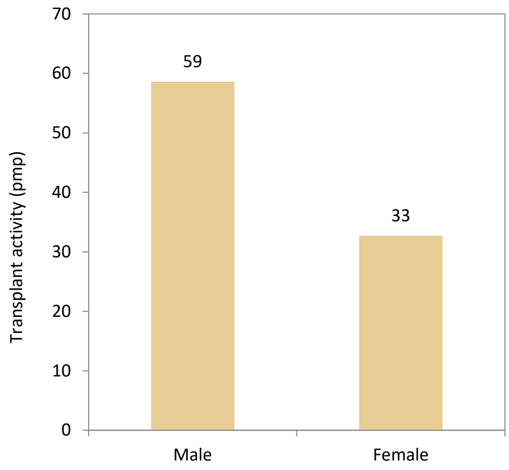

Figure A.7.8
Donor type distribution by sex in kidney transplant recipients, unadjusted

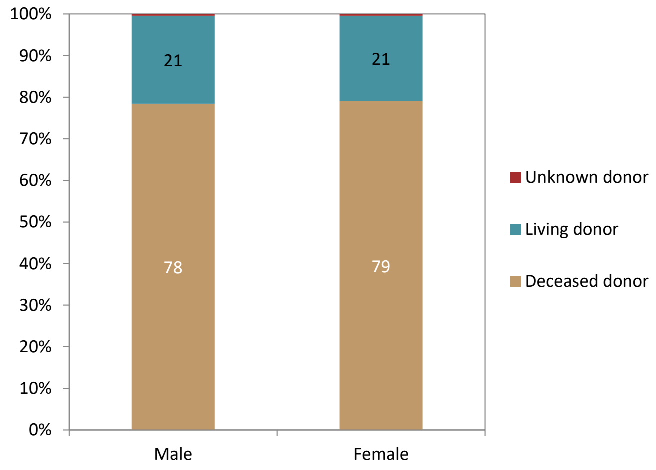

Figure A.7.9
Patient survival probability by sex: Incident dialysis patients (cohort 2013-2017)
from day 91, unadjusted

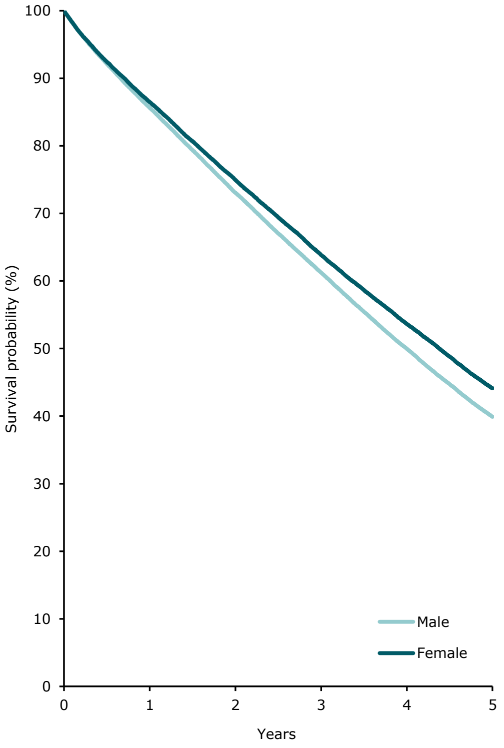

Figure A.7.10
Patient survival probability by sex: Patients receiving a first kidney transplant (cohort 2013-2017)
from day of transplant, unadjusted

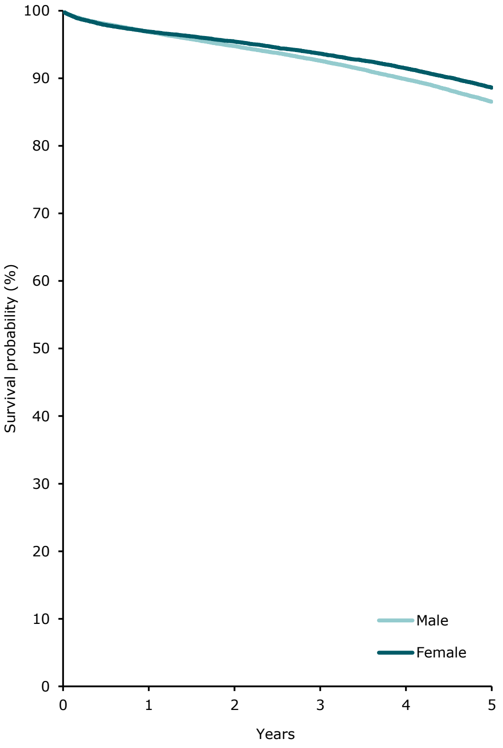

Section B: Individual patient data reference tables

Table B.1.1
General population data and number of renal centres

	General population of country/region in thousands	% Coverage of general population by the registry for individual patient data	Total number of renal centres in country/region	Number of renal centres collaborating with the registry for individual patient data
Austria §	8979	98.0	78	77
Belgium, Dutch-speaking	6749	100	26	26
Belgium, French-speaking	4931	100	26	26
Bosnia and Herzegovina	3531	100	30	30
Denmark	5903	100	15	15
Estonia	1349	100	3	3
France (17 of 18 regions)	67614	100	866	866
Greece	10437	100	213	213
Iceland	382	100	1	1
Montenegro	617	100	12	12
Norway	5457	100	27	27
Romania	19049	100	142	142
Serbia	6719	95.0	63	50
Spain, Andalusia	8542	100	63	63
Spain, Aragon	1343	100	10	10
Spain, Asturias	1006	100	11	11
Spain, Basque country	2213	100	10	10
Spain, Canary Islands $	2199	100	21	15
Spain, Cantabria	587	100	2	2
Spain, Castile and León	2373	100	22	22
Spain, Castile-La Mancha	2069	100	15	15
Spain, Catalonia	7793	100	49	49
Spain, Community of Madrid	6750	95.0	40	38
Spain, Extremadura	1055	100	13	13
Spain, Galicia	2696	100	25	25
Spain, La Rioja	321	100	3	3
Spain, Murcia	1532	100	14	14
Spain, Navarre	668	100	3	3
Spain, Valencian region	5098	100	49	49
Sweden	10487	100	67	67
Switzerland	8777	99.0	103	103
the Netherlands &	17701	92 - 98	57	57
United Kingdom, England ¶	57106	92 - 93	58	57
United Kingdom, Northern Ireland	1911	100	6	6
United Kingdom, Scotland	5448	100	10	10
United Kingdom, Wales	3132	100	6	6

§ No data from one centre treating haemodialysis patients only, it is estimated that most of these patients are covered by other centres
$ No data from 6 centres which treat foreign patients only
& The percentage coverage is different for the incidence (92%), prevalence (96%) and transplant activity (98%) calculations
¶ The percentage coverage is different for the incidence (93%), prevalence (92%) and transplant activity (93%) calculation

Table B.2.1

Incident counts and percentages by age and sex

at day 1

	All			0-19						20-44						45-64						65-74						75+					
	All	Male	Female	All		Male		Female		All		Male		Female		All		Male		Female		All		Male		Female		All		Male		Female	
	N (100%)	N (100%)	N (100%)	N	%	N	%	N	%	N	%	N	%	N	%	N	%	N	%	N	%	N	%	N	%	N	%	N	%	N	%	N	%
Austria §	1073	699	374	10	1	7	1	3	1	120	11	64	9	56	15	330	31	229	33	101	27	289	27	190	27	99	26	324	30	209	30	115	31
Belgium, Dutch-speaking *	1177	728	449							78	7	43	6	35	8	249	21	152	21	97	22	334	28	223	31	111	25	516	44	310	43	206	46
Belgium, French-speaking *	978	614	364							79	8	50	8	29	8	236	24	154	25	82	23	292	30	170	28	122	34	371	38	240	39	131	36
Bosnia and Herzegovina	424	263	161	6	1	4	2	2	1	41	10	30	11	11	7	153	36	100	38	53	33	145	34	89	34	56	35	79	19	40	15	39	24
Denmark	707	447	260	9	1	4	1	5	2	95	13	62	14	33	13	239	34	139	31	100	38	168	24	113	25	55	21	196	28	129	29	67	26
Estonia	112	61	51	2	2	0	0	2	4	13	12	6	10	7	14	46	41	25	41	21	41	35	31	22	36	13	25	16	14	8	13	8	16
France (17 of 18 regions)	10989	7322	3667	131	1	89	1	42	1	1043	9	684	9	359	10	2852	26	1849	25	1003	27	3106	28	2102	29	1004	27	3857	35	2598	35	1259	34
Greece	2907	1956	951	13	0	11	1	2	0	142	5	93	5	49	5	577	20	408	21	169	18	749	26	521	27	228	24	1426	49	923	47	503	53
Iceland	33	25	8	3	9	2	8	1	13	7	21	6	24	1	13	10	30	7	28	3	38	5	15	4	16	1	13	8	24	6	24	2	25
Montenegro *	49	30	19							7	14	6	20	1	5	10	20	6	20	4	21	22	45	11	37	11	58	10	20	7	23	3	16
Norway	553	386	167	5	1	4	1	1	1	61	11	38	10	23	14	171	31	123	32	48	29	160	29	109	28	51	31	156	28	112	29	44	26
Romania	3654	2195	1459	37	1	21	1	16	1	370	10	244	11	126	9	1366	37	868	40	498	34	1222	33	699	32	523	36	659	18	363	17	296	20
Serbia	500	322	175	8	2	5	2	3	2	65	13	45	14	20	11	174	35	106	33	67	38	167	33	114	35	52	30	85	17	51	16	33	19
Spain, Andalusia	1281	816	465	13	1	11	1	2	0	139	11	94	12	45	10	378	30	226	28	152	33	366	29	245	30	121	26	385	30	240	29	145	31
Spain, Aragon	191	141	50	5	3	4	3	1	2	20	10	14	10	6	12	53	28	36	26	17	34	59	31	44	31	15	30	54	28	43	30	11	22
Spain, Asturias	193	129	64	0	0	0	0	0	0	8	4	4	3	4	6	52	27	31	24	21	33	71	37	51	40	20	31	62	32	43	33	19	30
Spain, Basque country	300	200	100	1	0	0	0	1	1	32	11	14	7	18	18	106	35	68	34	38	38	82	27	57	29	25	25	79	26	61	31	18	18
Spain, Canary Islands	392	265	127	1	0	0	0	1	1	46	12	25	9	21	17	132	34	94	35	38	30	117	30	80	30	37	29	96	24	66	25	30	24
Spain, Cantabria *	92	57	35							10	11	7	12	3	9	24	26	12	21	12	34	27	29	17	30	10	29	31	34	21	37	10	29
Spain, Castile and León *	318	221	97							22	7	14	6	8	8	93	29	65	29	28	29	78	25	59	27	19	20	125	39	83	38	42	43
Spain, Castile-La Mancha *	241	159	82							13	5	8	5	5	6	92	38	53	33	39	48	56	23	41	26	15	18	80	33	57	36	23	28
Spain, Catalonia	1422	946	476	15	1	9	1	6	1	125	9	76	8	49	10	407	29	255	27	152	32	402	28	287	30	115	24	473	33	319	34	154	32
Spain, Community of Madrid	702	462	240	5	1	4	1	1	0	99	14	59	13	40	17	213	30	132	29	81	34	177	25	131	28	46	19	208	30	136	29	72	30
Spain, Extremadura	167	106	61	1	1	1	1	0	0	18	11	9	8	9	15	53	32	30	28	23	38	43	26	30	28	13	21	52	31	36	34	16	26
Spain, Galicia	409	276	133	3	1	2	1	1	1	25	6	13	5	12	9	142	35	92	33	50	38	115	28	76	28	39	29	124	30	93	34	31	23
Spain, La Rioja	40	26	14	0	0	0	0	0	0	3	8	2	8	1	7	13	33	10	38	3	21	12	30	6	23	6	43	12	30	8	31	4	29
Spain, Murcia	252	169	83	1	0	0	0	1	1	23	9	11	7	12	14	76	30	51	30	25	30	72	29	52	31	20	24	80	32	55	33	25	30
Spain, Navarre *	86	56	30							13	15	9	16	4	13	30	35	18	32	12	40	23	27	13	23	10	33	20	23	16	29	4	13
Spain, Valencian region	768	505	263	7	1	5	1	2	1	62	8	41	8	21	8	226	29	145	29	81	31	236	31	154	30	82	31	237	31	160	32	77	29
Sweden	1115	740	375	22	2	13	2	9	2	127	11	88	12	39	10	304	27	198	27	106	28	320	29	203	27	117	31	342	31	238	32	104	28
Switzerland	889	588	301	15	2	12	2	3	1	91	10	51	9	40	13	251	28	168	29	83	28	215	24	134	23	81	27	317	36	223	38	94	31
the Netherlands &	1902	1229	673	22	1	13	1	9	1	261	14	171	14	90	13	613	32	387	31	226	34	567	30	357	29	210	31	439	23	301	24	138	21
United Kingdom, England ¶ #	6647	4346	2301	153	2	84	2	69	3	1075	16	683	16	392	17	2371	36	1505	35	866	38	1616	24	1064	24	552	24	1432	22	1010	23	422	18
United Kingdom, Northern Ireland #	203	129	74							33	16	24	19	9	12	71	35	45	35	26	35	46	23	27	21	19	26	48	24	31	24	17	23
United Kingdom, Scotland	624	355	254	8	1	6	2	2	1	101	16	64	18	37	15	245	39	148	42	97	38	163	26	82	23	81	32	92	15	55	15	37	15
United Kingdom, Wales #	423	262	161					0	0	82	19	47	18	35	22	136	32	73	28	63	39	96	23	67	26	29	18	104	25	70	27	34	21

Categories may not add up because of missing values or rounding
§ The incidence is underestimated by approximately 2% due to one haemodialysis centre not submitting data
** Patients younger than 20 years of age are not reported*
& The incidence is underestimated by approximately 8%
¶ The incidence is underestimated by approximately 7.5% due to one centre not providing data and patients opting out of data sharing for research purposes
# Values based on 1 to 5 patients are suppressed

Table B.2.2
Incidence per million (age-related) population by age and sex, unadjusted
at day 1

	All			0-19			20-44			45-64			65-74			75+		
	All	Male	Female	All	Male	Female	All	Male	Female	All	Male	Female	All	Male	Female	All	Male	Female
	Pmp	Pmp	Pmp	Pmarp	Pmarp	Pmarp	Pmarp	Pmarp	Pmarp	Pmarp	Pmarp	Pmarp	Pmarp	Pmarp	Pmarp	Pmarp	Pmarp	Pmarp
Austria	121.9	161.2	83.8	5.9	8.0	3.6	42.1	44.0	40.1	129.9	181.5	79.0	329.1	463.1	211.6	389.1	620.9	231.8
Belgium, Dutch-speaking *	174.4	217.7	131.9				37.7	41.2	34.1	137.7	166.7	108.2	456.6	624.9	296.4	753.0	1079.1	517.6
Belgium, French-speaking *	198.3	253.6	145.0				48.1	60.6	35.5	185.6	242.5	128.8	607.2	760.2	474.2	945.1	1589.9	542.2
Bosnia and Herzegovina	120.1	151.8	89.5	7.6	9.9	5.2	33.5	48.2	18.3	150.1	201.3	101.4	493.2	690.4	339.2	379.9	498.6	305.3
Denmark	119.8	152.3	87.6	7.0	6.0	7.9	51.2	65.7	36.1	154.2	179.5	129.0	268.1	372.8	170.0	339.3	510.7	206.1
Estonia	83.0	95.3	72.0	6.9	0	14.1	30.1	26.8	33.8	130.4	146.1	115.6	235.6	368.2	146.4	127.2	218.7	89.7
France (17 of 18 regions)	162.5	223.7	105.1	8.2	10.9	5.4	51.8	68.7	35.3	165.1	219.0	113.5	410.3	598.2	247.6	577.1	979.7	312.3
Greece	278.5	383.3	178.3	6.7	11.0	2.1	46.3	59.7	32.6	189.2	274.1	108.3	635.3	941.5	364.4	1185.5	1840.9	717.1
Iceland	86.4	127.2	43.1	32.1	41.6	22.1	49.6	80.2	15.1	111.1	152.4	68.0	150.3	240.8	60.0	332.4	543.6	153.5
Montenegro *	79.4	98.4	60.8				33.6	56.4	9.8	62.1	76.7	48.3	336.4	369.4	308.8	289.5	517.0	142.8
Norway	101.3	140.3	61.7	4.0	6.3	1.7	33.7	40.9	26.1	121.6	171.5	69.6	294.9	406.0	186.1	340.7	557.3	171.2
Romania	191.8	237.2	148.9	8.9	9.9	8.0	63.6	81.5	44.6	254.9	328.5	183.3	538.5	719.6	403.0	451.0	701.6	313.6
Serbia	78.3	103.7	53.4	6.4	7.8	5.0	32.6	44.4	20.5	98.8	123.8	74.1	190.6	290.3	107.6	166.8	253.3	107.0
Spain, Andalusia	150.0	194.0	107.3	7.5	12.3	2.4	51.7	69.0	34.0	146.9	177.5	117.0	447.4	633.7	280.5	529.1	826.4	331.6
Spain, Aragon	142.2	212.5	73.6	20.2	31.4	8.3	50.8	69.5	31.2	130.4	175.3	84.6	415.4	651.4	201.3	352.0	697.2	119.9
Spain, Asturias	191.9	268.8	121.7	0	0	0	31.2	31.1	31.3	157.6	192.4	124.4	515.3	800.5	270.0	451.8	824.3	223.4
Spain, Basque country	135.5	185.9	87.9	2.5	0	5.2	52.2	45.1	59.5	154.0	199.4	109.5	321.7	478.4	184.2	302.0	601.4	112.4
Spain, Canary Islands	178.3	244.0	114.1	2.6	0	5.4	63.4	69.0	57.8	185.2	263.2	106.9	559.5	798.3	339.7	559.2	929.2	298.1
Spain, Cantabria *	156.8	200.3	115.8				61.4	85.4	37.1	128.6	130.3	126.8	382.6	510.0	268.6	470.3	825.7	247.1
Spain, Castile and León *	134.0	189.2	80.5				35.0	43.5	26.1	124.5	173.0	75.4	271.6	415.2	131.0	373.2	611.9	210.7
Spain, Castile-La Mancha *	116.5	153.3	79.5				20.4	24.3	16.3	147.2	166.4	127.2	288.1	431.8	150.9	389.9	675.6	190.4
Spain, Catalonia	182.5	246.8	120.2	9.7	11.3	8.0	50.5	60.4	40.2	179.0	224.4	133.7	531.6	819.1	283.4	634.4	1080.3	342.0
Spain, Community of Madrid	109.5	150.6	71.8	4.0	6.2	1.6	47.6	57.6	37.8	112.1	144.6	82.1	298.7	496.8	139.9	357.5	609.6	200.7
Spain, Extremadura	158.3	203.3	114.4	5.3	10.3	0	57.5	56.4	58.6	162.7	182.8	142.3	393.3	559.0	233.5	443.0	767.8	226.9
Spain, Galicia	151.7	212.7	95.1	7.2	9.3	4.9	34.0	35.2	32.9	169.9	225.1	117.1	350.4	493.1	224.0	327.7	623.0	135.3
Spain, La Rioja	124.6	164.1	86.1	0	0	0	32.4	42.9	21.7	132.9	202.9	61.8	352.7	360.9	344.9	337.7	553.3	189.8
Spain, Murcia	164.5	220.2	108.6	2.9	0	6.1	45.7	42.5	49.2	172.4	228.7	114.7	560.9	856.0	295.8	675.3	1141.3	355.7
Spain, Navarre *	128.7	169.4	88.9				65.6	89.7	40.9	151.0	179.1	122.3	340.8	398.9	286.6	291.7	575.9	98.1
Spain, Valencian region	150.6	201.2	101.6	7.2	9.9	4.2	39.9	52.1	27.4	146.0	187.8	104.4	446.0	621.0	291.7	484.5	804.4	265.3
Sweden	106.3	140.2	72.0	9.0	10.4	7.6	37.8	50.7	24.0	118.7	152.8	83.7	298.6	385.7	214.5	322.3	505.3	176.2
Switzerland	102.3	136.3	68.8	8.7	13.5	3.6	31.6	34.9	28.3	103.6	138.2	68.8	255.9	332.8	185.2	386.8	646.7	198.0
the Netherlands	116.8	151.8	82.2	6.4	7.4	5.4	50.6	65.6	35.3	139.1	176.0	102.4	314.7	403.7	228.9	297.3	468.8	165.4
United Kingdom, England #	125.8	167.9	85.4	12.5	13.4	11.6	62.2	80.5	44.5	175.8	226.9	126.3	318.6	436.3	209.6	300.8	488.0	156.8
United Kingdom, Northern Ireland #	106.3	137.2	76.2				55.1	81.1	29.7	143.5	185.6	103.1	256.6	306.9	208.1	307.3	462.9	190.6
United Kingdom, Scotland	114.5	134.1	90.7	7.1	10.4	3.6	58.7	76.0	42.1	163.7	203.9	125.8	271.9	285.4	259.5	184.8	259.9	129.2
United Kingdom, Wales #	135.1	170.7	100.8			0	86.7	100.5	73.1	165.8	182.5	149.8	271.8	391.4	159.3	324.5	497.3	189.2

** Patients younger than 20 years of age are not reported*
# Values based on 1 to 5 patients are suppressed

Table B.2.3

Sex, mean age, and median age of incident patients

at day 1

	All						Male						Female					
	%	Mean (years)	SD	Median (years)	P25	P75	%	Mean (years)	SD	Median (years)	P25	P75	%	Mean (years)	SD	Median (years)	P25	P75
Austria	100	65.2	15.6	67.8	57.2	76.9	65.1	65.4	15.2	67.6	57.7	76.7	34.9	64.7	16.4	68.2	55.1	77.6
Belgium, Dutch-speaking *	100	70.4	14.3	73.5	63.5	80.6	61.9	70.5	13.6	73.5	64.1	80.3	38.1	70.1	15.2	73.5	62.0	81.1
Belgium, French-speaking *	100	68.4	14.1	71.4	60.6	78.1	62.8	68.4	14.0	71.9	60.3	78.2	37.2	68.3	14.4	70.8	61.8	77.8
Bosnia and Herzegovina	100	63.5	14.3	65.9	56.9	72.8	62.0	62.6	14.6	64.6	56.5	71.6	38.0	65.0	13.8	68.7	57.5	74.5
Denmark	100	63.0	16.3	65.7	52.8	76.1	63.2	63.7	16.3	67.3	53.8	76.3	36.8	61.7	16.4	63.1	51.3	75.2
Estonia	100	61.2	15.8	62.4	53.9	72.0	54.5	62.9	13.6	64.9	56.1	72.4	45.5	59.1	18.1	58.6	51.7	71.7
France (17 of 18 regions)	100	66.9	16.0	70.4	58.5	78.1	66.6	67.0	15.9	70.7	59.0	78.2	33.4	66.5	16.1	69.8	57.7	78.1
Greece	100	72.0	13.9	74.7	64.9	82.5	67.3	71.4	13.9	74.0	64.5	82.0	32.7	73.3	13.7	75.9	66.2	83.6
Iceland	100	55.9	22.0	61.8	43.5	72.4	75.8	55.9	21.2	61.9	42.9	72.4	24.2	55.8	26.1	59.3	44.7	74.8
Montenegro *	100	63.4	15.2	67.4	51.8	73.2	61.2	61.5	16.5	66.7	47.4	73.6	38.8	66.3	12.6	69.9	54.3	73.2
Norway	100	64.2	15.4	67.5	54.4	76.3	69.8	64.5	15.5	68.2	54.3	76.4	30.2	63.5	15.2	66.7	54.4	76.1
Romania	100	63.0	14.2	65.6	54.7	72.7	60.1	62.2	14.2	64.4	53.9	72.1	39.9	64.3	14.1	66.9	56.6	73.6
Serbia	100	61.9	15.1	65.4	54.2	72.1	64.4	61.8	15.1	66.3	53.5	72.1	35.0	61.9	15.2	64.6	55.0	72.2
Spain, Andalusia	100	64.8	15.6	68.2	55.7	76.7	63.7	64.5	16.1	68.4	55.1	76.7	36.3	65.3	14.7	67.8	56.9	76.8
Spain, Aragon	100	64.6	16.4	68.2	58.6	76.4	73.8	64.8	16.9	69.6	54.7	76.5	26.2	64.0	15.3	65.9	60.1	73.2
Spain, Asturias	100	68.6	11.8	71.6	61.8	77.0	66.8	69.6	11.2	72.1	64.7	77.0	33.2	66.6	12.6	69.7	58.0	76.8
Spain, Basque country	100	64.2	14.7	66.7	55.5	75.3	66.7	66.4	13.6	68.7	59.1	76.5	33.3	59.8	16.0	60.3	50.1	72.7
Spain, Canary Islands	100	63.9	14.6	66.6	55.3	74.8	67.6	65.0	13.6	67.3	56.5	75.0	32.4	61.5	16.4	66.4	51.5	73.9
Spain, Cantabria *	100	66.6	14.6	70.4	57.7	77.5	62.0	66.9	15.4	70.8	58.8	77.7	38.0	66.0	13.4	68.4	57.7	75.7
Spain, Castile and León *	100	68.6	13.6	71.4	60.6	78.9	69.5	68.8	13.2	71.0	60.6	78.7	30.5	68.2	14.4	73.1	59.6	79.3
Spain, Castile-La Mancha *	100	66.7	13.8	67.6	58.2	78.0	66.0	68.1	13.5	69.7	60.3	78.0	34.0	64.1	14.0	64.0	52.9	77.3
Spain, Catalonia	100	66.4	15.2	69.7	57.6	77.7	66.5	67.0	14.7	70.2	59.1	77.8	33.5	65.2	16.1	68.3	55.5	77.5
Spain, Community of Madrid	100	64.1	16.1	66.5	54.7	76.4	65.8	64.5	16.1	68.0	55.8	76.2	34.2	63.1	16.2	64.6	51.9	77.1
Spain, Extremadura	100	65.5	15.1	69.0	57.7	77.2	63.5	66.7	15.1	71.2	59.2	77.8	36.5	63.5	15.1	61.9	52.8	76.2
Spain, Galicia	100	66.3	13.8	67.9	57.3	76.7	67.5	67.2	13.4	69.1	58.9	77.5	32.5	64.2	14.5	66.3	54.0	74.9
Spain, La Rioja	100	65.9	13.5	66.5	57.1	77.3	65.0	65.3	12.9	66.0	56.6	77.0	35.0	67.0	14.9	68.2	61.1	81.1
Spain, Murcia	100	65.6	14.3	69.6	56.5	77.2	67.1	67.2	12.2	69.7	59.4	77.6	32.9	62.5	17.6	68.8	52.0	76.7
Spain, Navarre *	100	62.8	15.6	64.8	52.7	73.8	65.1	64.0	15.9	66.4	52.7	77.1	34.9	60.6	15.0	64.3	51.8	72.2
Spain, Valencian region	100	66.4	14.5	69.0	58.1	76.9	65.8	66.6	14.7	69.5	57.9	77.2	34.2	65.9	14.0	68.2	58.4	76.4
Sweden	100	64.6	16.7	68.8	56.2	76.8	66.4	64.8	16.7	69.4	55.8	77.1	33.6	64.3	16.6	68.0	57.1	76.0
Switzerland	100	65.7	17.0	69.8	56.5	78.2	66.1	66.2	17.1	70.6	57.1	78.7	33.9	64.7	16.7	68.5	55.1	77.4
the Netherlands	100	62.5	15.9	66.0	54.0	74.4	64.6	62.8	15.9	66.3	54.3	74.9	35.4	62.0	15.9	65.5	53.8	73.2
United Kingdom, England	100	60.4	17.1	63.3	50.1	73.6	65.4	61.2	17.0	64.0	51.0	74.4	34.6	59.0	17.3	61.5	48.5	72.2
United Kingdom, Northern Ireland	100	60.3	17.4	63.2	49.0	74.7	63.5	60.0	17.2	61.5	49.0	74.7	36.5	60.9	17.8	64.1	49.4	74.6
United Kingdom, Scotland	100	59.3	15.7	61.7	50.6	70.9	56.9	58.8	16.2	60.4	49.1	71.4	40.7	60.2	15.1	63.7	51.3	70.4
United Kingdom, Wales	100	60.8	16.9	63.3	47.7	74.9	61.9	61.7	17.4	66.0	49.5	75.4	38.1	59.3	16.1	60.1	46.8	73.7

Categories may not add up because of missing values or rounding

** Patients younger than 20 years of age are not reported*

Table B.2.4
Incidence per million population, adjusted
at day 1, adjusted for age and sex

	All	Male	Female
	Pmp	Pmp	Pmp
Austria	124.4	165.0	85.6
Belgium, Dutch-speaking *	174.2	214.3	135.8
Belgium, French-speaking *	229.6	296.4	165.8
Bosnia and Herzegovina	152.4	201.2	105.7
Denmark	122.0	153.7	91.7
Estonia	90.0	107.7	73.0
France (17 of 18 regions)	167.9	228.8	109.6
Greece	254.9	347.0	166.9
Iceland	100.8	148.9	54.9
Montenegro *	92.7	117.5	69.0
Norway	109.0	150.5	69.3
Romania	202.0	256.6	149.9
Serbia	75.9	101.3	51.7
Spain, Andalusia	161.7	209.8	115.7
Spain, Aragon	138.1	205.1	74.1
Spain, Asturias	159.6	220.4	101.5
Spain, Basque country	126.8	171.0	84.5
Spain, Canary Islands	189.3	258.5	123.2
Spain, Cantabria *	146.9	188.7	106.9
Spain, Castile and León *	113.1	156.0	72.0
Spain, Castile-La Mancha *	119.1	157.3	82.6
Spain, Catalonia	193.4	263.1	126.7
Spain, Community of Madrid	119.4	164.7	76.1
Spain, Extremadura	153.4	195.9	112.8
Spain, Galicia	133.4	182.2	86.7
Spain, La Rioja	121.8	152.5	92.4
Spain, Murcia	193.2	265.7	124.0
Spain, Navarre *	130.3	170.3	92.0
Spain, Valencian region	152.7	204.4	103.4
Sweden	109.3	142.7	77.5
Switzerland	106.0	140.4	73.0
the Netherlands	117.6	151.2	85.4
United Kingdom, England	134.9	178.4	93.3
United Kingdom, Northern Ireland	116.7	148.4	86.3
United Kingdom, Scotland	112.8	134.3	92.4
United Kingdom, Wales	135.4	168.4	103.9

** Patients younger than 20 years of age are not reported*

Table B.2.5.A
Incidence per million population and percentages by primary renal disease (1995 PRD codes), unadjusted
at day 1

	All		GN		PN		PKD		DM						HT		RVD		Misc		Unkn		Missing	
									Type 1		Type 2		Both											
	Pmp	%	Pmp	%	Pmp	%	Pmp	%	Pmp	%	Pmp	%	Pmp	%	Pmp	%	Pmp	%	Pmp	%	Pmp	%	Pmp	%
Austria	121.9	100	12.4	10.2	3.2	2.6	6.4	5.2	3.9	3.2	24.7	20.2	28.5	23.4	17.3	14.2	1.3	1.0	29.0	23.8	23.6	19.4	0.3	0.3
Belgium, Dutch-speaking *	174.4	100	15.1	8.7	5.2	3.0	7.3	4.2	3.6	2.0	32.3	18.5	35.9	20.6	16.6	9.5	6.8	3.9	62.8	36.0	24.7	14.2	0	0
Belgium, French-speaking *	198.3	100	17.2	8.7	6.1	3.1	11.0	5.5	3.2	1.6	35.1	17.7	38.3	19.3	35.5	17.9	1.4	0.7	57.2	28.8	31.6	16.0	0	0
Bosnia and Herzegovina	120.1	100	10.2	8.5	7.1	5.9	4.5	3.8	8.5	7.1	26.9	22.4	35.4	29.5	15.9	13.2	1.7	1.4	17.8	14.9	27.5	22.9	0	0
Denmark	119.8	100	15.4	12.9	7.1	5.9	8.8	7.4	7.6	6.4	25.4	21.2	33.0	27.6	14.1	11.7	2.7	2.3	22.5	18.8	16.1	13.4	0	0
Estonia	83.0	100	13.3	16.1	4.4	5.4	9.6	11.6					14.8	17.9	25.9	31.3	0.7	0.9	14.1	17.0	0	0	0	0
France (17 of 18 regions)	162.5	100	17.7	10.9	8.1	5.0	10.2	6.3					36.2	22.3	38.6	23.8	1.7	1.0	24.2	14.9	25.8	15.9	0	0
Greece	278.5	100	17.7	6.4	14.7	5.3	9.7	3.5	2.5	0.9	60.7	21.8	63.2	22.7	27.0	9.7	6.0	2.2	33.4	12.0	106.7	38.3	0	0
Iceland	86.4	100	5.2	6.1	7.9	9.1	5.2	6.1	2.6	3.0	15.7	18.2	18.3	21.2	26.2	30.3	0	0	20.9	24.2	2.6	3.0	0	0
Montenegro *	79.4	100	0	0	1.6	2.0	0	0	1.6	2.0	21.1	26.5	22.7	28.6	27.5	34.7	0	0	17.8	22.4	9.7	12.2	0	0
Norway	101.3	100	14.1	13.9	4.4	4.3	9.7	9.6	5.1	5.1	11.9	11.8	17.0	16.8	29.3	28.9	0.7	0.7	19.8	19.5	4.2	4.2	2.0	2.0
Romania	191.8	100	25.2	13.1	7.5	3.9	4.1	2.2					21.0	10.9	11.2	5.8	1.2	0.6	30.8	16.1	90.8	47.3	0	0
Serbia	78.3	100	6.4	8.2	6.1	7.8	2.8	3.6					15.2	19.4	25.5	32.6	0.8	1.0	14.4	18.4	2.2	2.8	4.9	6.2
Spain, Andalusia	150.0	100	17.4	11.6	7.6	5.1	9.7	6.5					37.6	25.1	16.6	11.1	0.5	0.3	27.5	18.3	33.0	22.0	0	0
Spain, Aragon	142.2	100	22.3	15.7	8.2	5.8	10.4	7.3	5.2	3.7	26.1	18.3	31.3	22.0	31.3	22.0	0	0	23.1	16.2	15.6	11.0	0	0
Spain, Asturias	191.9	100	25.8	13.5	8.9	4.7	14.9	7.8	3.0	1.6	43.7	22.8	46.7	24.4	37.8	19.7	2.0	1.0	27.8	14.5	27.8	14.5	0	0
Spain, Basque country	135.5	100	13.6	10.0	8.1	6.0	12.2	9.0	0.5	0.3	27.6	20.3	28.0	20.7	19.4	14.3	0	0	26.2	19.3	26.2	19.3	1.8	1.3
Spain, Canary Islands	178.3	100	15.9	8.9	5.0	2.8	15.0	8.4	10.0	5.6	45.9	25.8	55.9	31.4	11.8	6.6	0.5	0.3	35.0	19.6	39.1	21.9	0	0
Spain, Cantabria *	156.8	100	39.2	25.0	10.2	6.5	22.2	14.1	3.4	2.2	22.2	14.1	25.6	16.3	10.2	6.5	3.4	2.2	11.9	7.6	10.2	6.5	23.9	15.2
Spain, Castile and León * ‡	134.0	100	16.4	12.3	8.9	6.6	8.0	6.0	3.0	2.2	29.1	21.7	32.0	23.9	21.1	15.7	0.4	0.3	29.1	21.7	18.1	13.5	0	0
Spain, Castile-La Mancha *	116.5	100	8.7	7.5	6.8	5.8	8.2	7.1					27.1	23.2	10.2	8.7	1.9	1.7	24.2	20.7	29.5	25.3	0	0
Spain, Catalonia	182.5	100	17.6	9.6	8.7	4.8	12.2	6.7	3.0	1.6	31.1	17.0	34.6	19.0	10.8	5.9	3.5	1.9	16.8	9.2	37.7	20.7	40.6	22.2
Spain, Community of Madrid	109.5	100	10.3	9.4	2.5	2.3	6.7	6.1	1.6	1.4	20.1	18.4	21.7	19.8	7.6	7.0	1.1	1.0	13.4	12.3	15.7	14.4	30.4	27.8
Spain, Extremadura	158.3	100	22.8	14.4	14.2	9.0	5.7	3.6					29.4	18.6	17.1	10.8	5.7	3.6	27.5	17.4	23.7	15.0	12.3	7.8
Spain, Galicia	151.7	100	20.8	13.7	5.2	3.4	15.2	10.0	7.0	4.6	27.4	18.1	34.5	22.7	16.3	10.8	0	0	24.1	15.9	35.6	23.5	0	0
Spain, La Rioja	124.6	100	9.3	7.5	0	0	12.5	10.0	3.1	2.5	12.5	10.0	15.6	12.5	12.5	10.0	0	0	53.0	42.5	21.8	17.5	0	0
Spain, Murcia	164.5	100	24.8	15.1	5.2	3.2	11.8	7.1	3.9	2.4	37.9	23.0	41.8	25.4	19.6	11.9	0	0	35.3	21.4	26.1	15.9	0	0
Spain, Navarre *	128.7	100	9.0	7.0	1.5	1.2	18.0	14.0	12.0	9.3	23.9	18.6	35.9	27.9	21.0	16.3	0	0	26.9	20.9	16.5	12.8	0	0
Spain, Valencian region	150.6	100	16.9	11.2	6.9	4.6	7.8	5.2					27.3	18.1	24.1	16.0	1.2	0.8	36.3	24.1	30.2	20.1	0	0
Sweden ‡	106.3	100	14.3	13.5	3.6	3.4	7.6	7.2	5.5	5.2	20.2	19.0	25.7	24.2	19.1	17.9	1.0	0.9	25.3	23.8	9.7	9.1	0	0
Switzerland	102.3	100	14.8	14.5	2.3	2.2	6.2	6.1	2.5	2.5	18.8	18.3	21.3	20.8	21.2	20.7	3.2	3.1	25.4	24.9	7.8	7.6	0	0
the Netherlands	116.8	100	12.6	10.8	4.4	3.8	6.5	5.6	2.7	2.3	20.0	17.1	22.7	19.5	18.3	15.7	4.6	3.9	29.9	25.6	16.5	14.1	1.2	1.1
United Kingdom, England #	125.8	100	13.2	10.5	5.9	4.7	6.5	5.1					31.5	25.0	8.3	6.6	1.1	0.9	22.7	18.1	16.2	12.9	20.4	16.2
United Kingdom, Northern Ireland #	106.3	100	14.1	13.3	9.9	9.4	6.8	6.4					18.3	17.2					20.4	19.2	9.9	9.4	23.0	21.7
United Kingdom, Scotland	114.5	100	13.4	11.7	6.4	5.6	7.3	6.4					27.4	23.9	5.0	4.3	1.8	1.6	25.3	22.1	13.2	11.5	14.7	12.8
United Kingdom, Wales #	135.1	100	18.5	13.7	8.0	5.9	7.3	5.4					39.9	29.6	5.7	4.3	0	0	31.9	23.6	16.9	12.5	6.7	5.0

Abbreviations used: GN: glomerulonephritis/sclerosis; PN: pyelonephritis; PKD: polycystic kidneys, adult type; DM: diabetes mellitus; HT: hypertension; RVD: renal vascular disease; Misc: miscellaneous; Unkn: unknown
Categories may not add up because of rounding or a limited number of cases (<10%) with diabetes mellitus type unknown; When cells are left empty, (complete) data are unavailable
** Patients younger than 20 years of age are not reported*
‡ Mapping the 2018 PRD codes to the old PRD codes results in a different distribution of PRD groups
# Values based on 1 to 5 patients are suppressed

Table B.2.5.B

Incidence per million population and percentages by primary renal disease (2012/2018 PRD codes), unadjusted

at day 1

	All		Glomerular disease		Tubulo-interstitial disease		DM		HT / RVD		Other systemic diseases		Familial/ hereditary nephropathies		Misc		Unkn		Missing	
	**Pmp**	**%**	**Pmp**	**%**	**Pmp**	**%**	**Pmp**	**%**	**Pmp**	**%**	**Pmp**	**%**	**Pmp**	**%**	**Pmp**	**%**	**Pmp**	**%**	**Pmp**	**%**
Austria	121.9	100	17.2	14.1	7.0	5.8	28.5	23.4	21.6	17.7	7.0	5.8	9.2	7.5	12.7	10.4	18.3	15.0	0.3	0.3
Belgium, Dutch-speaking *	174.4	100	19.3	11.0	10.5	6.0	35.9	20.6	37.8	21.7	25.0	14.4	10.5	6.0	23.9	13.7	11.6	6.6	0	0
Belgium, French-speaking *	198.3	100	20.1	10.1	18.9	9.5	38.3	19.3	38.5	19.4	16.2	8.2	15.2	7.7	31.4	15.8	19.7	9.9	0	0
Bosnia and Herzegovina	120.1	100	11.9	9.9	10.5	8.7	34.8	29.0	17.0	14.2	2.8	2.4	8.8	7.3	4.5	3.8	29.7	24.8	0	0
Denmark	119.8	100	20.8	17.4	12.4	10.3	33.0	27.6	18.8	15.7	1.9	1.6	10.8	9.1	6.3	5.2	15.8	13.2	0	0
Iceland	86.4	100	13.1	15.2	15.7	18.2	18.3	21.2	26.2	30.3	0	0	10.5	12.1	0	0	2.6	3.0	0	0
Montenegro *	79.4	100	1.6	2.0	1.6	2.0	22.7	28.6	27.5	34.7	4.9	6.1	6.5	8.2	11.3	14.3	3.2	4.1	0	0
Norway	101.3	100	17.0	16.8	8.6	8.5	17.0	16.8	31.3	30.9	5.9	5.8	12.1	11.9	4.2	4.2	3.1	3.1	2.0	2.0
Spain, Andalusia	150.0	100	21.2	14.1	15.6	10.4	37.6	25.1	19.8	13.2	6.0	4.0	13.2	8.8	6.3	4.2	30.3	20.2	0	0
Spain, Aragon	142.2	100	23.8	16.8	13.4	9.4	31.3	22.0	32.8	23.0	6.7	4.7	11.9	8.4	10.4	7.3	11.9	8.4	0	0
Spain, Asturias	191.9	100	30.8	16.1	16.9	8.8	46.7	24.4	41.8	21.8	4.0	2.1	18.9	9.8	5.0	2.6	27.8	14.5	0	0
Spain, Basque country	135.5	100	15.8	11.7	16.3	12.0	28.0	20.7	22.1	16.3	3.2	2.3	15.4	11.3	7.7	5.7	25.3	18.7	1.8	1.3
Spain, Canary Islands	178.3	100	19.1	10.7	15.0	8.4	55.9	31.4	19.6	11.0	7.3	4.1	16.8	9.4	5.5	3.1	39.1	21.9	0	0
Spain, Cantabria *	156.8	100	22.2	14.1	11.9	7.6	25.6	16.3	13.6	8.7	0	0	22.2	14.1	6.8	4.3	27.3	17.4	27.3	17.4
Spain, Castile and León *	134.0	100	23.6	17.6	14.8	11.0	32.0	23.9	23.6	17.6	7.2	5.3	11.0	8.2	6.3	4.7	15.6	11.6	0	0
Spain, Catalonia	182.5	100	21.0	11.5	10.5	5.8	33.7	18.5	14.4	7.9	3.7	2.0	15.1	8.3	6.2	3.4	37.2	20.4	40.6	22.2
Spain, Community of Madrid	109.5	100	11.7	10.7	7.5	6.8	21.7	19.8	9.2	8.4	2.2	2.0	7.6	7.0	5.0	4.6	14.2	13.0	30.4	27.8
Spain, Galicia	151.7	100	26.0	17.1	11.5	7.6	34.5	22.7	18.2	12.0	4.1	2.7	16.3	10.8	11.9	7.8	29.3	19.3	0	0
Spain, La Rioja	124.6	100	12.5	10.0	18.7	15.0	15.6	12.5	24.9	20.0	3.1	2.5	18.7	15.0	9.3	7.5	21.8	17.5	0	0
Spain, Murcia	164.5	100	29.4	17.9	11.1	6.7	41.8	25.4	35.3	21.4	3.9	2.4	12.4	7.5	5.2	3.2	25.5	15.5	0	0
Spain, Navarre *	128.7	100	16.5	12.8	4.5	3.5	35.9	27.9	21.0	16.3	7.5	5.8	18.0	14.0	9.0	7.0	16.5	12.8	0	0
Spain, Valencian region	150.6	100	21.4	14.2	15.5	10.3	27.3	18.1	29.0	19.3	11.0	7.3	11.4	7.6	7.1	4.7	28.1	18.6	0	0
Sweden	106.3	100	18.6	17.5	9.7	9.1	25.7	24.2	23.6	22.2	3.8	3.6	10.7	10.0	7.9	7.4	6.3	5.9	0	0
United Kingdom, England #	125.8	100	17.1	13.6	9.1	7.3	30.4	24.2	11.2	8.9	3.9	3.1	7.6	6.0	7.3	5.8	13.8	11.0	25.3	20.1
United Kingdom, Northern Ireland #	106.3	100	12.6	11.8	12.6	11.8	18.3	17.2	7.3	6.9			7.9	7.4	7.9	7.4	6.8	6.4	30.4	28.6
United Kingdom, Scotland	114.5	100	16.5	14.4	9.7	8.5	27.4	23.9	12.8	11.2	2.8	2.4	9.7	8.5	4.8	4.2	12.3	10.7	18.5	16.2
United Kingdom, Wales #	135.1	100	22.4	16.5	10.5	7.8	40.2	29.8	17.9	13.2	3.5	2.6	9.3	6.9	11.5	8.5	12.8	9.5	7.0	5.2

Abbreviations used: DM: diabetes mellitus; HT / RVD: hypertension / renal vascular disease; Misc: miscellaneous renal disorders; Unkn: unknown
** Patients younger than 20 years of age are not reported*
# Values based on 1 to 5 patients are suppressed

Table B.2.6.A

Incidence per million population by primary renal disease (1995 PRD codes), adjusted

at day 1, adjusted for age and sex

	All	GN	PN	PKD	DM			HT	RVD	Misc	Unkn	Missing
					Type 1	Type 2	Both					
	Pmp	Pmp	Pmp	Pmp	Pmp	Pmp	Pmp	Pmp	Pmp	Pmp	Pmp	Pmp
Austria	124.4	12.4	3.4	6.5	3.8	25.4	29.3	17.5	1.3	29.5	24.1	0.4
Belgium, Dutch-speaking *	174.2	15.3	5.2	7.6	3.6	32.3	35.9	16.6	6.7	62.1	24.8	0
Belgium, French-speaking *	229.6	19.4	7.1	12.2	3.4	41.1	44.5	42.6	1.6	65.0	37.2	0
Bosnia and Herzegovina	152.4	11.1	9.4	5.6	9.8	33.9	43.8	21.7	2.4	21.9	36.6	0
Denmark	122.0	16.1	6.9	9.3	8.0	26.1	34.0	13.7	2.9	22.9	16.1	0
Estonia	90.0	14.2	4.8	10.4			15.6	29.5	0.8	14.7	0	0
France (17 of 18 regions)	167.9	18.5	8.4	10.9			37.5	39.5	1.7	24.9	26.5	0
Greece	254.9	17.1	13.3	9.3	2.4	56.1	58.5	24.4	5.5	30.9	96.0	0
Iceland	100.8	4.9	9.8	6.4	2.5	19.1	21.6	33.6	0	22.7	1.8	0
Montenegro *	92.7	0	1.8	0	1.4	23.4	24.9	34.0	0	19.4	12.7	0
Norway	109.0	15.1	4.6	10.7	5.3	13.0	18.3	31.8	0.9	21.2	4.5	2.1
Romania	202.0	26.6	8.2	4.1			21.9	11.9	1.3	32.5	95.5	0
Serbia	75.9	6.2	5.9	2.6			14.3	24.7	0.7	14.1	2.2	5.1
Spain, Andalusia	161.7	19.0	8.0	10.2			40.3	17.8	0.6	29.8	36.0	0
Spain, Aragon	138.1	22.0	8.1	9.7	5.2	25.5	30.6	29.7	0	22.5	15.4	0
Spain, Asturias	159.6	22.4	7.5	12.6	3.2	36.4	39.6	30.3	1.5	23.0	22.8	0
Spain, Basque country	126.8	13.2	7.4	11.0	0.4	25.3	25.7	18.4	0	24.9	24.6	1.6
Spain, Canary Islands	189.3	16.2	5.7	14.2	9.2	50.4	59.6	13.1	0.5	38.6	41.5	0
Spain, Cantabria *	146.9	36.9	9.0	20.6	3.1	21.0	24.1	9.5	3.2	12.3	8.6	22.7
Spain, Castile and León * ‡	113.1	14.7	7.1	7.3	2.6	24.1	26.7	17.4	0.4	24.5	15.0	0
Spain, Castile-La Mancha *	119.1	8.6	7.0	8.4			27.7	9.6	2.0	25.1	30.7	0
Spain, Catalonia	193.4	18.4	9.3	12.1	2.9	33.9	37.5	11.5	3.8	17.5	40.4	43.0
Spain, Community of Madrid	119.4	10.5	2.6	6.7	1.8	22.8	24.5	8.4	1.2	14.7	17.4	33.3
Spain, Extremadura	153.4	21.5	14.1	5.3			28.6	16.6	4.9	27.3	23.4	11.8
Spain, Galicia	133.4	19.3	4.3	13.4	6.9	24.3	31.2	13.9	0	21.0	30.2	0
Spain, La Rioja	121.8	9.5	0	11.9	2.9	12.6	15.5	11.8	0	51.7	21.5	0
Spain, Murcia	193.2	26.9	6.0	11.4	3.8	47.5	51.3	25.2	0	41.7	30.7	0
Spain, Navarre *	130.3	9.1	1.6	18.1	12.0	23.9	35.9	21.1	0	27.6	16.9	0
Spain, Valencian region	152.7	16.6	6.9	7.6			27.5	24.9	1.2	36.9	31.1	0
Sweden ‡	109.3	14.5	3.6	8.2	5.9	20.9	26.8	19.3	1.0	26.0	10.0	0
Switzerland	106.0	15.0	2.4	6.4	2.5	19.7	22.2	21.9	3.3	26.4	8.3	0
the Netherlands	117.6	12.6	4.4	6.8	2.8	19.8	22.5	18.6	4.7	30.0	16.7	1.2
United Kingdom, England #	134.9	13.9	6.2	7.1			34.1	9.0	1.2	24.4	17.4	21.7
United Kingdom, Northern Ireland #	116.7	15.0	11.1	7.2			20.2			22.6	11.2	25.2
United Kingdom, Scotland	112.8	13.1	6.3	7.4			27.4	5.2	1.9	25.5	13.0	13.0
United Kingdom, Wales #	135.4	19.1	8.2	8.0			39.4	5.8	0	31.2	16.9	6.8

Abbreviations used: GN: glomerulonephritis/sclerosis; PN: pyelonephritis; PKD: polycystic kidneys, adult type; DM: diabetes mellitus; HT: hypertension; RVD: renal vascular disease; Misc: miscellaneous; Unkn: unknown
Categories may not add up because of rounding or a limited number of cases (<10%) with diabetes mellitus type unknown; When cells are left empty, (complete) data are unavailable
** Patients younger than 20 years of age are not reported*
‡ Mapping the 2018 PRD codes to the old PRD codes results in a different distribution of PRD groups
# Values based on 1 to 5 patients are suppressed

Table B.2.6.B

Incidence per million population by primary renal disease (2012/2018 PRD codes), adjusted

at day 1, adjusted for age and sex

	All	Glomerular disease	Tubulo-interstitial disease	DM	HT / RVD	Other systemic diseases	Familial/ hereditary nephropathies	Misc	Unkn	Missing
	Pmp	Pmp	Pmp	Pmp	Pmp	Pmp	Pmp	Pmp	Pmp	Pmp
Austria	124.4	17.2	7.3	29.3	22.2	7.2	9.4	13.0	18.7	0.4
Belgium, Dutch-speaking *	174.2	19.5	10.5	35.9	37.4	24.7	10.9	23.7	11.6	0
Belgium, French-speaking *	229.6	22.5	21.7	44.5	46.2	19.1	16.6	36.0	23.1	0
Bosnia and Herzegovina	152.4	13.0	14.2	42.9	23.5	3.6	10.1	5.4	39.6	0
Denmark	122.0	21.7	12.3	34.0	18.6	1.9	11.3	6.4	15.8	0
Iceland	100.8	14.5	18.1	21.6	33.6	0	11.3	0	1.8	0
Montenegro *	92.7	1.8	1.8	24.9	34.0	4.9	7.7	13.2	4.5	0
Norway	109.0	18.3	9.1	18.3	34.1	6.1	13.0	4.6	3.4	2.1
Spain, Andalusia	161.7	23.0	16.6	40.3	21.3	6.5	14.0	7.0	33.0	0
Spain, Aragon	138.1	23.3	13.1	30.6	31.2	6.8	11.3	10.2	11.7	0
Spain, Asturias	159.6	26.4	14.5	39.6	33.4	3.1	16.2	3.7	22.8	0
Spain, Basque country	126.8	15.5	15.1	25.7	21.0	3.0	13.9	7.3	23.7	1.6
Spain, Canary Islands	189.3	19.4	16.6	59.6	22.0	8.3	16.1	5.9	41.5	0
Spain, Cantabria *	146.9	21.6	10.6	24.1	12.6	0	21.1	6.5	24.3	26.1
Spain, Castile and León *	113.1	20.6	12.0	26.7	19.5	6.1	10.1	5.4	12.7	0
Spain, Catalonia	193.4	21.9	11.0	36.6	15.4	3.9	15.0	6.6	40.0	43.0
Spain, Community of Madrid	119.4	12.1	8.1	24.5	10.2	2.4	7.6	5.7	15.5	33.3
Spain, Galicia	133.4	23.7	10.2	31.2	15.5	3.4	14.4	10.5	24.5	0
Spain, La Rioja	121.8	12.2	19.2	15.5	24.7	3.4	17.4	7.9	21.5	0
Spain, Murcia	193.2	31.8	12.6	51.3	44.5	4.7	12.2	6.1	30.1	0
Spain, Navarre *	130.3	16.4	4.7	35.9	21.1	7.8	18.1	9.4	16.9	0
Spain, Valencian region	152.7	21.2	15.7	27.5	30.1	11.3	11.0	7.1	28.8	0
Sweden	109.3	19.0	9.9	26.8	23.9	3.9	11.3	8.0	6.5	0
United Kingdom, England #	134.9	18.0	9.6	33.0	12.1	4.3	8.2	7.9	14.8	27.0
United Kingdom, Northern Ireland #	116.7	13.4	13.3	20.2	8.6		8.3	8.8	7.6	33.5
United Kingdom, Scotland	112.8	16.2	9.8	27.4	13.2	2.7	9.8	4.6	12.1	16.9
United Kingdom, Wales #	135.4	23.0	10.6	39.8	17.4	3.6	9.9	11.2	12.9	7.1

Abbreviations used: DM: diabetes mellitus; HT / RVD: hypertension / renal vascular disease; Misc: miscellaneous renal disorders; Unkn: unknown
** Patients younger than 20 years of age are not reported*
# Values based on 1 to 5 patients are suppressed

Table B.2.7

Incident counts by treatment modality

at day 1

	All	Haemodialysis						Peritoneal dialysis				Preemptive kidney transplant				Unkn	Missing
		HD hospital/ centre	HD home	HD type Unkn	HF	HDF	Total HD/ HF/HDF	APD	CAPD	PD type Unkn	Total PD	Living donor	Deceased donor	Tx type Unkn	Total Tx		
	N	N	N	N	N	N	N	N	N	N	N	N	N	N	N	N	N
Austria §	1073	847	0	0	3	109	959	16	63	3	82	18	14	0	32	0	0
Belgium, Dutch-speaking *	1177	770	1	0	0	272	1043	52	66	0	118	9	7	0	16	0	0
Belgium, French-speaking *	978	514	5	0	53	302	874	45	41	0	86	3	15	0	18	0	0
Bosnia and Herzegovina	424	390	0	0	0	22	412	0	11	0	11	1	0	0	1	0	0
Denmark	707	416	0	0			416	84	152	1	237	26	28	0	54	0	0
Estonia	112	97	0	0	0	0	97	14	0	0	14	1	0	0	1	0	0
France (17 of 18 regions)	10989	7309	5	16	24	2120	9474	206	850	13	1069	201	245	0	446	0	0
Greece	2907	2742	1	0	0	30	2773	25	93	0	118	16	0	0	16	0	0
Iceland	33	25	0	0			25	0	6	0	6	2	0	0	2	0	0
Montenegro *	49	49	0	0	0	0	49	0	0	0	0	0	0	0	0	0	0
Norway	553	349	0	0			349			159	159	17	28	0	45	0	0
Romania ||	3654	3612	0	0	0	6	3618	1	35	0	36	0	0	0	0	0	0
Serbia	500	444	0	1	0	7	452	3	40	0	43	4	1	0	5	0	0
Spain, Andalusia	1281	1026	8	0			1034			202	202	22	23	0	45	0	0
Spain, Aragon	191	126	0	0		27	153	5	24	0	29	3	6	0	9	0	0
Spain, Asturias	193	151	0	0		0	151	3	33	0	36	1	5	0	6	0	0
Spain, Basque country	300	212	7	0			219	1	68	0	69	10	2	0	12	0	0
Spain, Canary Islands	392	278	2	0	0	0	280	0	0	107	107	2	3	0	5	0	0
Spain, Cantabria *	92	69	1	0			70	3	5	0	8	1	13	0	14	0	0
Spain, Castile and León *	318	252	3	0			255	17	38	0	55	4	4	0	8	0	0
Spain, Castile-La Mancha *	241	200	2	0			202	5	34	0	39	0	0	0	0	0	0
Spain, Catalonia	1422	770	6	0		320	1096	31	147	0	178	82	66	0	148	0	0
Spain, Community of Madrid	702	499	4	0		60	563	35	75	0	110	15	14	0	29	0	0
Spain, Extremadura	167	150	4	0			154	7	4	0	11	2	0	0	2	0	0
Spain, Galicia	409	312	3	0	0	0	315	4	72	0	76	14	4	0	18	0	0
Spain, La Rioja	40	28	1	0	0	0	29	3	6	0	9	2	0	0	2	0	0
Spain, Murcia	252	212	0	0	0	1	213	0	34	0	34	3	2	0	5	0	0
Spain, Navarre *	86	60	0	0			60	22	1	0	23	1	2	0	3	0	0
Spain, Valencian region	768	548	17	0			565	15	122	0	137	7	59	0	66	0	0
Sweden	1115	624	2	0			626			401	401	35	53	0	88	0	0
Switzerland	889	723	4	19	3	0	749	33	69	2	104	30	5	0	35	1	0
the Netherlands &	1902	1266	4	0		5	1275	122	231	0	353	240	33	0	273	1	0
United Kingdom, England ¶ #	6647	4179	23		10	753	4967	582	657	32	1271	183	216	10	409	0	0
United Kingdom, Northern Ireland #	203	118	0	0			120	28	7	0	35	32	16	0	48	0	0
United Kingdom, Scotland	624	459	0	0			459	50	46	13	109	30	24	2	56	0	0
United Kingdom, Wales #	423	296		0	0	26	324	19	63		83	8	8	0	16	0	0

Abbreviations used: HD: haemodialysis; Unkn: unknown; HF: haemofiltration; HDF: haemodiafiltration; APD: automated peritoneal dialysis; CAPD: continuous ambulatory peritoneal dialysis; PD: peritoneal dialysis; Tx: transplant
When cells are left empty, (complete) data are unavailable
§ The incidence is underestimated by approximately 2% due to an estimated 2% underestimation of the incidence of haemodialysis
** Patients younger than 20 years of age are not reported*
|| The incidence of preemptive transplantation is underestimated by approximately 30%
& The incidence is underestimated by approximately 8%
¶ The incidence is underestimated by approximately 7.5% due to one centre not providing data and patients opting out of data sharing for research purposes
# Values based on 1 to 5 patients are suppressed

Table B.2.8
Incidence per million population by treatment modality, unadjusted
at day 1

	All	Haemodialysis						Peritoneal dialysis				Preemptive kidney transplant				Unkn	Missing
		HD hospital/ centre	HD home	HD type Unkn	HF	HDF	Total HD/ HF/HDF	APD	CAPD	PD type Unkn	Total PD	Living donor	Deceased donor	Tx type Unkn	Total Tx		
	Pmp	Pmp	Pmp	Pmp	Pmp	Pmp	Pmp	Pmp	Pmp	Pmp	Pmp	Pmp	Pmp	Pmp	Pmp	Pmp	Pmp
Austria	121.9	96.3	0	0	0.3	12.4	109.0	1.8	7.2	0.3	9.3	2.0	1.6	0	3.6	0	0
Belgium, Dutch-speaking *	174.4	114.1	0.1	0	0	40.3	154.5	7.7	9.8	0	17.5	1.3	1.0	0	2.4	0	0
Belgium, French-speaking *	198.3	104.2	1.0	0	10.7	61.2	177.2	9.1	8.3	0	17.4	0.6	3.0	0	3.7	0	0
Bosnia and Herzegovina	120.1	110.4	0	0	0	6.2	116.7	0	3.1	0	3.1	0.3	0	0	0.3	0	0
Denmark	119.8	70.5	0	0			70.5	14.2	25.7	0.2	40.1	4.4	4.7	0	9.1	0	0
Estonia	83.0	71.9	0	0	0	0	71.9	10.4	0	0	10.4	0.7	0	0	0.7	0	0
France (17 of 18 regions)	162.5	108.1	0.1	0.2	0.4	31.4	140.1	3.0	12.6	0.2	15.8	3.0	3.6	0	6.6	0	0
Greece	278.5	262.7	0.1	0	0	2.9	265.7	2.4	8.9	0	11.3	1.5	0	0	1.5	0	0
Iceland	86.4	65.4	0	0			65.4	0	15.7	0	15.7	5.2	0	0	5.2	0	0
Montenegro *	79.4	79.4	0	0	0	0	79.4	0	0	0	0	0	0	0	0	0	0
Norway	101.3	64.0	0	0			64.0			29.1	29.1	3.1	5.1	0	8.2	0	0
Romania ||	191.8	189.6	0	0	0	0.3	189.9	0.1	1.8	0	1.9	0	0	0	0	0	0
Serbia	78.3	69.6	0	0.2	0	1.1	70.8	0.5	6.3	0	6.7	0.6	0.2	0	0.8	0	0
Spain, Andalusia	150.0	120.1	0.9	0			121.0			23.6	23.6	2.6	2.7	0	5.3	0	0
Spain, Aragon	142.2	93.8	0	0		20.1	113.9	3.7	17.9	0	21.6	2.2	4.5	0	6.7	0	0
Spain, Asturias	191.9	150.1	0	0		0	150.1	3.0	32.8	0	35.8	1.0	5.0	0	6.0	0	0
Spain, Basque country	135.5	95.8	3.2	0			98.9	0.5	30.7	0	31.2	4.5	0.9	0	5.4	0	0
Spain, Canary Islands	178.3	126.4	0.9	0	0	0	127.3	0	0	48.7	48.7	0.9	1.4	0	2.3	0	0
Spain, Cantabria *	156.8	117.6	1.7	0			119.3	5.1	8.5	0	13.6	1.7	22.2	0	23.9	0	0
Spain, Castile and León *	134.0	106.2	1.3	0			107.5	7.2	16.0	0	23.2	1.7	1.7	0	3.4	0	0
Spain, Castile-La Mancha *	116.5	96.7	1.0	0			97.6	2.4	16.4	0	18.9	0	0	0	0	0	0
Spain, Catalonia	182.5	98.8	0.8	0		41.1	140.6	4.0	18.9	0	22.8	10.5	8.5	0	19.0	0	0
Spain, Community of Madrid	109.5	77.8	0.6	0		9.4	87.8	5.5	11.7	0	17.2	2.3	2.2	0	4.5	0	0
Spain, Extremadura	158.3	142.2	3.8	0			146.0	6.6	3.8	0	10.4	1.9	0	0	1.9	0	0
Spain, Galicia	151.7	115.7	1.1	0	0	0	116.8	1.5	26.7	0	28.2	5.2	1.5	0	6.7	0	0
Spain, La Rioja	124.6	87.2	3.1	0	0	0	90.3	9.3	18.7	0	28.0	6.2	0	0	6.2	0	0
Spain, Murcia	164.5	138.4	0	0	0	0.7	139.0	0	22.2	0	22.2	2.0	1.3	0	3.3	0	0
Spain, Navarre *	128.7	89.8	0	0			89.8	32.9	1.5	0	34.4	1.5	3.0	0	4.5	0	0
Spain, Valencian region	150.6	107.5	3.3	0			110.8	2.9	23.9	0	26.9	1.4	11.6	0	12.9	0	0
Sweden	106.3	59.5	0.2	0			59.7			38.2	38.2	3.3	5.1	0	8.4	0	0
Switzerland	102.3	83.2	0.5	2.2	0.3	0	86.2	3.8	7.9	0.2	12.0	3.5	0.6	0	4.0	0.1	0
the Netherlands	116.8	77.7	0.2	0		0.3	78.3	7.5	14.2	0	21.7	14.7	2.0	0	16.8	0.1	0
United Kingdom, England #	125.8	79.1	0.4		0.2	14.3	94.0	11.0	12.4	0.6	24.1	3.5	4.1	0.2	7.7	0	0
United Kingdom, Northern Ireland #	106.3	61.8	0	0			62.8	14.7	3.7	0	18.3	16.7	8.4	0	25.1	0	0
United Kingdom, Scotland	114.5	84.3	0	0			84.3	9.2	8.4	2.4	20.0	5.5	4.4	0.4	10.3	0	0
United Kingdom, Wales #	135.1	94.5		0	0	8.3	103.5	6.1	20.1		26.5	2.6	2.6	0	5.1	0	0

Abbreviations used: HD: haemodialysis; Unkn: unknown; HF: haemofiltration; HDF: haemodiafiltration; APD: automated peritoneal dialysis; CAPD: continuous ambulatory peritoneal dialysis; PD: peritoneal dialysis; Tx: transplant
Categories may not add up because of rounding; When cells are left empty, (complete) data are unavailable
** Patients younger than 20 years of age are not reported*
|| The incidence of preemptive transplantation is underestimated by approximately 30%
# Values based on 1 to 5 patients are suppressed

Table B.2.9

Incidence per million population by treatment modality, adjusted

at day 1, adjusted for age and sex

	All	Haemodialysis						Peritoneal dialysis				Preemptive kidney transplant				Unkn	Missing
		HD hospital/ centre	HD home	HD type Unkn	HF	HDF	Total HD/ HF/HDF	APD	CAPD	PD type Unkn	Total PD	Living donor	Deceased donor	Tx type Unkn	Total Tx		
	Pmp	Pmp	Pmp	Pmp	Pmp	Pmp	Pmp	Pmp	Pmp	Pmp	Pmp	Pmp	Pmp	Pmp	Pmp	Pmp	Pmp
Austria	124.4	98.5	0	0	0.3	12.7	111.5	1.9	7.2	0.3	9.4	2.0	1.6	0	3.5	0	0
Belgium, Dutch-speaking *	174.2	113.9	0.1	0	0	40.1	154.2	7.8	9.9	0	17.6	1.3	1.1	0	2.4	0	0
Belgium, French-speaking *	229.6	121.6	1.1	0	12.0	71.0	205.7	10.5	9.7	0	20.2	0.7	3.1	0	3.8	0	0
Bosnia and Herzegovina	152.4	140.3	0	0	0	8.6	148.9	0	3.3	0	3.3	0.3	0	0	0.3	0	0
Denmark	122.0	72.1	0	0			72.1	14.1	26.0	0.2	40.2	4.7	4.9	0	9.6	0	0
Estonia	90.0	78.3	0	0	0	0	78.3	10.8	0	0	10.8	0.9	0	0	0.9	0	0
France (17 of 18 regions)	167.9	111.4	0.1	0.2	0.4	32.4	144.6	3.1	13.0	0.2	16.3	3.1	3.8	0	6.9	0	0
Greece	254.9	239.9	0.1	0	0	2.7	242.7	2.3	8.4	0	10.7	1.6	0	0	1.6	0	0
Iceland	100.8	80.7	0	0			80.7	0	15.7	0	15.7	4.3	0	0	4.3	0	0
Montenegro *	92.7	92.7	0	0	0	0	92.7	0	0	0	0	0	0	0	0	0	0
Norway	109.0	69.0	0	0			69.0			31.4	31.4	3.1	5.5	0	8.7	0	0
Romania ||	202.0	199.8	0	0	0	0.4	200.1	0	1.9	0	1.9	0	0	0	0	0	0
Serbia	75.9	67.2	0	0.1	0	1.2	68.5	0.5	6.1	0	6.6	0.6	0.1	0	0.8	0	0
Spain, Andalusia	161.7	129.7	1.0	0			130.7			25.5	25.5	2.7	2.8	0	5.5	0	0
Spain, Aragon	138.1	90.9	0	0		19.7	110.6	3.7	17.1	0	20.8	2.2	4.4	0	6.6	0	0
Spain, Asturias	159.6	124.3	0	0		0	124.3	2.5	26.8	0	29.3	0.8	5.2	0	6.0	0	0
Spain, Basque country	126.8	89.7	3.0	0			92.7	0.4	28.6	0	29.0	4.2	0.8	0	5.0	0	0
Spain, Canary Islands	189.3	136.0	1.0	0	0	0	137.0	0	0	50.1	50.1	0.8	1.4	0	2.1	0	0
Spain, Cantabria *	146.9	110.2	1.8	0			112.0	4.5	8.2	0	12.6	2.1	20.2	0	22.2	0	0
Spain, Castile and León *	113.1	88.9	1.3	0			90.2	6.1	13.8	0	19.9	1.5	1.5	0	3.1	0	0
Spain, Castile-La Mancha *	119.1	99.0	0.9	0			99.9	2.5	16.8	0	19.2	0	0	0	0	0	0
Spain, Catalonia	193.4	105.6	0.8	0		43.8	150.2	4.0	19.9	0	23.9	10.5	8.8	0	19.3	0	0
Spain, Community of Madrid	119.4	85.7	0.7	0		10.2	96.5	5.7	12.7	0	18.4	2.2	2.3	0	4.5	0	0
Spain, Extremadura	153.4	137.7	3.7	0			141.4	6.6	3.7	0	10.3	1.7	0	0	1.7	0	0
Spain, Galicia	133.4	101.3	0.9	0	0	0	102.1	1.5	23.6	0	25.1	4.8	1.3	0	6.1	0	0
Spain, La Rioja	121.8	85.7	2.9	0	0	0	88.6	9.0	18.3	0	27.3	5.8	0	0	5.8	0	0
Spain, Murcia	193.2	164.1	0	0	0	0.8	164.9	0	25.1	0	25.1	2.0	1.3	0	3.2	0	0
Spain, Navarre *	130.3	90.9	0	0			90.9	33.1	1.8	0	34.9	1.3	3.1	0	4.5	0	0
Spain, Valencian region	152.7	109.4	3.3	0			112.7	2.8	24.4	0	27.3	1.4	11.4	0	12.7	0	0
Sweden	109.3	61.3	0.2	0			61.5			38.9	38.9	3.5	5.5	0	9.0	0	0
Switzerland	106.0	86.6	0.5	2.2	0.3	0	89.6	3.8	8.2	0.2	12.3	3.4	0.6	0	4.0	0.1	0
the Netherlands	117.6	78.5	0.3	0		0.3	79.1	7.4	14.2	0	21.6	14.8	2.0	0	16.8	0.1	0
United Kingdom, England #	134.9	85.4	0.5		0.2	15.3	101.4	11.6	13.3	0.6	25.5	3.5	4.4	0.2	8.1	0	0
United Kingdom, Northern Ireland #	116.7	69.1	0	0			70.2	15.5	4.2	0	19.6	17.8	9.0	0	26.8	0	0
United Kingdom, Scotland	112.8	83.0	0	0			83.0	9.5	8.1	2.0	19.6	5.6	4.4	0.3	10.3	0	0
United Kingdom, Wales #	135.4	93.5		0	0	8.4	102.5	6.3	20.8		27.4	2.8	2.8	0	5.5	0	0

Abbreviations used: HD: haemodialysis; Unkn: unknown; HF: haemofiltration; HDF: haemodiafiltration; APD: automated peritoneal dialysis; CAPD: continuous ambulatory peritoneal dialysis; PD: peritoneal dialysis; Tx: transplant
Categories may not add up because of rounding; When cells are left empty, (complete) data are unavailable
** Patients younger than 20 years of age are not reported*
|| The incidence of preemptive transplantation is underestimated by approximately 30%
# Values based on 1 to 5 patients are suppressed

Table B.2.10

Treatment modality distribution, unadjusted

at day 1

	All	Haemodialysis						Peritoneal dialysis				Preemptive kidney transplant				Unkn	Missing
		HD hospital/ centre	HD home	HD type Unkn	HF	HDF	Total HD/ HF/HDF	APD	CAPD	PD type Unkn	Total PD	Living donor	Deceased donor	Tx type Unkn	Total Tx		
	%	%	%	%	%	%	%	%	%	%	%	%	%	%	%	%	%
Austria	100	78.9	0	0	0.3	10.2	89.4	1.5	5.9	0.3	7.6	1.7	1.3	0	3.0	0	0
Belgium, Dutch-speaking *	100	65.4	0.1	0	0	23.1	88.6	4.4	5.6	0	10.0	0.8	0.6	0	1.4	0	0
Belgium, French-speaking *	100	52.6	0.5	0	5.4	30.9	89.4	4.6	4.2	0	8.8	0.3	1.5	0	1.8	0	0
Bosnia and Herzegovina	100	92.0	0	0	0	5.2	97.2	0	2.6	0	2.6	0.2	0	0	0.2	0	0
Denmark	100	58.8	0	0			58.8	11.9	21.5	0.1	33.5	3.7	4.0	0	7.6	0	0
Estonia	100	86.6	0	0	0	0	86.6	12.5	0	0	12.5	0.9	0	0	0.9	0	0
France (17 of 18 regions)	100	66.5	0	0.1	0.2	19.3	86.2	1.9	7.7	0.1	9.7	1.8	2.2	0	4.1	0	0
Greece	100	94.3	0	0	0	1.0	95.4	0.9	3.2	0	4.1	0.6	0	0	0.6	0	0
Iceland	100	75.8	0	0			75.8	0	18.2	0	18.2	6.1	0	0	6.1	0	0
Montenegro *	100	100	0	0	0	0	100	0	0	0	0	0	0	0	0	0	0
Norway	100	63.1	0	0			63.1			28.8	28.8	3.1	5.1	0	8.1	0	0
Romania ||	100	98.9	0	0	0	0.2	99.0	0	1.0	0	1.0	0	0	0	0	0	0
Serbia	100	88.8	0	0.2	0	1.4	90.4	0.6	8.0	0	8.6	0.8	0.2	0	1.0	0	0
Spain, Andalusia	100	80.1	0.6	0			80.7			15.8	15.8	1.7	1.8	0	3.5	0	0
Spain, Aragon	100	66.0	0	0		14.1	80.1	2.6	12.6	0	15.2	1.6	3.1	0	4.7	0	0
Spain, Asturias	100	78.2	0	0		0	78.2	1.6	17.1	0	18.7	0.5	2.6	0	3.1	0	0
Spain, Basque country	100	70.7	2.3	0			73.0	0.3	22.7	0	23.0	3.3	0.7	0	4.0	0	0
Spain, Canary Islands	100	70.9	0.5	0	0	0	71.4	0	0	27.3	27.3	0.5	0.8	0	1.3	0	0
Spain, Cantabria *	100	75.0	1.1	0			76.1	3.3	5.4	0	8.7	1.1	14.1	0	15.2	0	0
Spain, Castile and León *	100	79.2	0.9	0			80.2	5.3	11.9	0	17.3	1.3	1.3	0	2.5	0	0
Spain, Castile-La Mancha *	100	83.0	0.8	0			83.8	2.1	14.1	0	16.2	0	0	0	0	0	0
Spain, Catalonia	100	54.1	0.4	0		22.5	77.1	2.2	10.3	0	12.5	5.8	4.6	0	10.4	0	0
Spain, Community of Madrid	100	71.1	0.6	0		8.5	80.2	5.0	10.7	0	15.7	2.1	2.0	0	4.1	0	0
Spain, Extremadura	100	89.8	2.4	0			92.2	4.2	2.4	0	6.6	1.2	0	0	1.2	0	0
Spain, Galicia	100	76.3	0.7	0	0	0	77.0	1.0	17.6	0	18.6	3.4	1.0	0	4.4	0	0
Spain, La Rioja	100	70.0	2.5	0	0	0	72.5	7.5	15.0	0	22.5	5.0	0	0	5.0	0	0
Spain, Murcia	100	84.1	0	0	0	0.4	84.5	0	13.5	0	13.5	1.2	0.8	0	2.0	0	0
Spain, Navarre *	100	69.8	0	0			69.8	25.6	1.2	0	26.7	1.2	2.3	0	3.5	0	0
Spain, Valencian region	100	71.4	2.2	0			73.6	2.0	15.9	0	17.8	0.9	7.7	0	8.6	0	0
Sweden	100	56.0	0.2	0			56.1			36.0	36.0	3.1	4.8	0	7.9	0	0
Switzerland	100	81.3	0.4	2.1	0.3	0	84.3	3.7	7.8	0.2	11.7	3.4	0.6	0	3.9	0.1	0
the Netherlands	100	66.6	0.2	0		0.3	67.0	6.4	12.1	0	18.6	12.6	1.7	0	14.4	0.1	0
United Kingdom, England #	100	62.9	0.3		0.2	11.3	74.7	8.8	9.9	0.5	19.1	2.8	3.2	0.2	6.2	0	0
United Kingdom, Northern Ireland #	100	58.1	0	0			59.1	13.8	3.4	0	17.2	15.8	7.9	0	23.6	0	0
United Kingdom, Scotland	100	73.6	0	0			73.6	8.0	7.4	2.1	17.5	4.8	3.8	0.3	9.0	0	0
United Kingdom, Wales #	100	70.0		0	0	6.1	76.6	4.5	14.9		19.6	1.9	1.9	0	3.8	0	0

Abbreviations used: HD: haemodialysis; Unkn: unknown; HF: haemofiltration; HDF: haemodiafiltration; APD: automated peritoneal dialysis; CAPD: continuous ambulatory peritoneal dialysis; PD: peritoneal dialysis; Tx: transplant
Categories may not add up because of rounding; When cells are left empty, (complete) data are unavailable
** Patients younger than 20 years of age are not reported*
|| The incidence of preemptive transplantation is underestimated by approximately 30%
# Values based on 1 to 5 patients are suppressed

Table B.2.11

Treatment modality distribution by age, sex, and primary renal disease, unadjusted

at day 1

	All				0-19				20-44				45-64				65-74				75+				Male				Female				DM				Non-DM			
	HD	PD	Tx	Un	HD	PD	Tx	Un	HD	PD	Tx	Un	HD	PD	Tx	Un	HD	PD	Tx	Un	HD	PD	Tx	Un	HD	PD	Tx	Un	HD	PD	Tx	Un	HD	PD	Tx	Un	HD	PD	Tx	Un
	%	%	%	%	%	%	%	%	%	%	%	%	%	%	%	%	%	%	%	%	%	%	%	%	%	%	%	%	%	%	%	%	%	%	%	%	%	%	%	%
Austria	89	8	3	0	50	0	50	0	79	10	11	0	85	11	4	0	94	6	0	0	94	6	0	0	89	8	3	0	90	7	3	0	93	4	2	0	88	9	3	0
Belgium, Dutch-speaking *	89	10	1	0					72	22	6	0	83	14	3	0	90	9	0	0	93	7	0	0	88	11	1	0	90	8	2	0	88	11	0	0	89	10	2	0
Belgium, French-speaking *	89	9	2	0					78	11	10	0	86	11	3	0	93	6	1	0	91	9	0	0	90	9	1	0	88	9	3	0	91	8	1	0	89	9	2	0
Bosnia and Herzegovina	97	3	0	0	100	0	0	0	98	0	2	0	94	6	0	0	99	1	0	0	99	1	0	0	97	3	0	0	98	2	1	0	97	3	0	0	97	2	0	0
Denmark	59	34	8	0	22	56	22	0	54	33	14	0	51	36	13	0	67	29	5	0	65	34	1	0	57	36	7	0	61	30	9	0	66	29	5	0	56	35	9	0
Estonia	87	13	1	0	100	0	0	0	77	15	8	0	85	15	0	0	94	6	0	0	81	19	0	0	87	11	2	0	86	14	0	0	100	0	0	0	84	15	1	0
France (17 of 18 regions)	86	10	4	0	57	19	24	0	74	12	14	0	84	10	7	0	90	8	2	0	90	10	1	0	87	9	4	0	85	10	4	0	92	7	1	0	85	11	5	0
Greece	95	4	1	0	77	15	8	0	84	8	8	0	94	6	0	0	95	5	0	0	97	3	0	0	95	4	1	0	96	3	1	0	97	3	0	0	95	4	1	0
Iceland	76	18	6	0	33	0	67	0	57	43	0	0	80	20	0	0	80	20	0	0	100	0	0	0	72	24	4	0	88	0	13	0	86	14	0	0	73	19	8	0
Montenegro *	100	0	0	0					100	0	0	0	100	0	0	0	100	0	0	0	100	0	0	0	100	0	0	0	100	0	0	0	100	0	0	0	100	0	0	0
Norway	63	29	8	0	40	20	40	0	54	23	23	0	64	24	12	0	64	31	5	0	65	35	0	0	63	31	7	0	64	25	11	0	65	32	3	0	63	28	9	0
Romania ||	99	1	0	0	81	19	0	0	99	1	0	0	99	1	0	0	99	1	0	0	100	0	0	0	99	1	0	0	99	1	0	0	99	1	0	0	99	1	0	0
Serbia	90	9	1	0	63	38	0	0	89	6	5	0	86	13	1	0	95	5	0	0	95	5	0	0	92	7	1	0	88	11	1	0	90	10	0	0	91	8	1	0
Spain, Andalusia	81	16	4	0	62	15	23	0	81	17	3	0	79	16	5	0	81	16	3	0	83	15	2	0	80	16	3	0	82	15	4	0	88	11	1	0	78	17	4	0
Spain, Aragon	80	15	5	0	60	20	20	0	65	10	25	0	77	21	2	0	78	19	3	0	93	7	0	0	79	16	4	0	82	12	6	0	93	5	2	0	77	18	5	0
Spain, Asturias	78	19	3	0	0	0	0	0	63	13	25	0	75	19	6	0	77	21	1	0	84	16	0	0	79	19	2	0	77	17	6	0	70	28	2	0	81	16	3	0
Spain, Basque country	73	23	4	0	100	0	0	0	78	16	6	0	65	29	6	0	76	22	2	0	78	19	3	0	74	23	4	0	72	24	4	0	84	16	0	0	70	25	5	0
Spain, Canary Islands	71	27	1	0	100	0	0	0	63	35	2	0	69	29	2	0	68	31	1	0	82	18	0	0	73	26	1	0	69	29	2	0	82	17	1	0	67	32	1	0
Spain, Cantabria *	76	9	15	0					70	0	30	0	75	17	8	0	63	11	26	0	90	3	6	0	84	0	16	0	63	23	14	0	80	0	20	0	75	10	14	0
Spain, Castile and León *	80	17	3	0					82	9	9	0	65	30	5	0	81	18	1	0	91	9	0	0	80	17	3	0	80	18	2	0	88	8	4	0	78	20	2	0
Spain, Castile-La Mancha *	84	16	0	0					69	31	0	0	77	23	0	0	79	21	0	0	98	3	0	0	83	17	0	0	85	15	0	0	91	9	0	0	82	18	0	0
Spain, Catalonia	77	13	10	0	40	7	53	0	70	11	19	0	67	17	16	0	77	14	9	0	89	8	3	0	77	12	10	0	76	13	11	0	86	11	3	0	75	13	12	0
Spain, Community of Madrid	80	16	4	0	40	20	40	0	74	17	9	0	73	22	5	0	80	16	3	0	91	8	0	0	81	16	3	0	79	15	6	0	90	7	3	0	78	18	4	0
Spain, Extremadura	92	7	1	0	100	0	0	0	83	17	0	0	92	4	4	0	93	7	0	0	94	6	0	0	91	8	1	0	95	3	2	0	94	6	0	0	92	7	1	0
Spain, Galicia	77	19	4	0	67	33	0	0	72	20	8	0	70	21	8	0	78	19	3	0	85	15	1	0	78	18	3	0	74	19	7	0	85	15	0	0	75	20	6	0
Spain, La Rioja	73	23	5	0	0	0	0	0	67	33	0	0	54	31	15	0	75	25	0	0	92	8	0	0	65	27	8	0	86	14	0	0	60	40	0	0	74	20	6	0
Spain, Murcia	85	13	2	0	100	0	0	0	70	30	0	0	78	16	7	0	86	14	0	0	94	6	0	0	86	12	2	0	82	17	1	0	86	14	0	0	84	13	3	0
Spain, Navarre *	70	27	3	0					69	15	15	0	70	27	3	0	70	30	0	0	70	30	0	0	71	25	4	0	67	30	3	0	83	17	0	0	65	31	5	0
Spain, Valencian region	74	18	9	0	29	29	43	0	65	15	21	0	67	19	13	0	69	24	7	0	88	11	1	0	75	18	7	0	71	17	12	0	83	12	5	0	72	19	9	0
Sweden	56	36	8	0	32	36	32	0	52	36	12	0	51	36	13	0	58	35	7	0	62	36	1	0	54	39	6	0	60	29	11	0	59	36	5	0	55	36	9	0
Switzerland	84	12	4	0	20	53	27	0	74	18	9	0	77	14	8	0	87	12	1	0	94	6	0	0	84	12	4	0	85	11	4	0	90	8	2	0	83	13	5	0
the Netherlands	67	19	14	0	41	23	36	0	57	15	28	0	62	17	21	0	72	19	9	0	75	22	3	0	67	19	14	0	67	18	15	0	72	23	5	0	66	17	17	0
United Kingdom, England #	75	19	6	0	44	38	18	0	63	24	13	0	75	18	6	0	78	17	4	0	82	17	1	0	76	19	6	0	73	20	7	0	80	17	3	0	73	20	7	0
United Kingdom, Northern Ireland #	59	17	24	0				0	33	27	39	0	55	20	25	0	70		26	0	75	19		0	57	17	26	0	64	18	19	0	91			0	52	20	28	0
United Kingdom, Scotland	74	17	9	0	13	63	25	0	66	22	12	0	68	19	13	0	84	12	4	0	83	15	2	0	75	17	8	0	72	18	11	0	77	14	9	0	72	19	9	0
United Kingdom, Wales #	77	20	4	0			0	0	57	32	11	0	74	21	5	0	84	16	0	0	88	12	0	0	77	19	3	0	75	20	4	0	85	14		0	73	22	5	0

Abbreviations used: HD: haemodialysis; PD: peritoneal dialysis; Tx: transplant; Un: unknown; DM: diabetes mellitus
Categories may not add up because of missing values or rounding
** Patients younger than 20 years of age are not reported*
|| The incidence of preemptive transplantation is underestimated by approximately 30%
# Values based on 1 to 5 patients are suppressed

Table B.3.1

Incident counts and percentages by age and sex

at day 91

	All			0-19						20-44						45-64						65-74						75+					
	All	Male	Female	All		Male		Female		All		Male		Female		All		Male		Female		All		Male		Female		All		Male		Female	
	N (100%)	N (100%)	N (100%)	N	%	N	%	N	%	N	%	N	%	N	%	N	%	N	%	N	%	N	%	N	%	N	%	N	%	N	%	N	%
Austria §	971	638	333	6	1	4	1	2	1	106	11	58	9	48	14	304	31	213	33	91	27	263	27	173	27	90	27	292	30	190	30	102	31
Belgium, Dutch-speaking *	1065	663	402							74	7	41	6	33	8	225	21	140	21	85	21	293	28	194	29	99	25	473	44	288	43	185	46
Belgium, French-speaking *	845	523	322							66	8	43	8	23	7	202	24	130	25	72	22	246	29	140	27	106	33	331	39	210	40	121	38
Bosnia and Herzegovina	377	237	140	5	1	3	1	2	1	38	10	27	11	11	8	140	37	94	40	46	33	129	34	82	35	47	34	65	17	31	13	34	24
Denmark	673	426	247	9	1	4	1	5	2	88	13	57	13	31	13	232	34	136	32	96	39	159	24	105	25	54	22	185	27	124	29	61	25
Estonia †	111	60	51	2	2	0	0	2	4	13	12	6	10	7	14	45	41	24	40	21	41	36	32	23	38	13	25	15	14	7	12	8	16
France (17 of 18 regions) †	10216	6806	3410	124	1	85	1	39	1	1007	10	660	10	347	10	2684	26	1737	26	947	28	2847	28	1946	29	901	26	3553	35	2378	35	1175	34
Greece	2637	1792	845	12	0	10	1	2	0	138	5	90	5	48	6	539	20	384	21	155	18	694	26	481	27	213	25	1254	48	827	46	427	51
Iceland	30	23	7	3	10	2	9	1	14	7	23	6	26	1	14	10	33	7	30	3	43	4	13	4	17	0	0	6	20	4	17	2	29
Montenegro * †	34	21	14							6	18	5	24	1	7	9	26	6	29	3	21	14	41	8	38	6	43	6	18	2	10	4	29
Norway	527	367	160	5	1	4	1	1	1	57	11	35	10	22	14	164	31	118	32	46	29	149	28	99	27	50	31	152	29	111	30	41	26
Romania	3014	1820	1194	36	1	20	1	16	1	327	11	219	12	108	9	1168	39	748	41	420	35	969	32	546	30	423	35	514	17	287	16	227	19
Serbia	459	298	158	8	2	5	2	3	2	57	12	41	14	16	10	165	36	99	33	65	41	153	33	106	36	46	29	75	16	46	15	28	18
Spain, Andalusia	1241	794	447	11	1	11	1	0	0	131	11	90	11	41	9	377	30	227	29	150	34	348	28	229	29	119	27	374	30	237	30	137	31
Spain, Aragon	186	136	50	5	3	4	3	1	2	20	11	14	10	6	12	51	27	35	26	16	32	57	31	41	30	16	32	53	28	42	31	11	22
Spain, Asturias	184	122	62	0	0	0	0	0	0	8	4	4	3	4	6	50	27	30	25	20	32	66	36	47	39	19	31	60	33	41	34	19	31
Spain, Basque country	276	182	94	1	0	0	0	1	1	33	12	17	9	16	17	98	36	59	32	39	41	72	26	51	28	21	22	72	26	55	30	17	18
Spain, Canary Islands	392	265	127	1	0	0	0	1	1	45	11	25	9	20	16	130	33	92	35	38	30	118	30	81	31	37	29	98	25	67	25	31	24
Spain, Cantabria *	85	52	33							10	12	7	13	3	9	24	28	12	23	12	36	25	29	15	29	10	30	26	31	18	35	8	24
Spain, Castile and León * †	314	217	97							21	7	13	6	8	8	89	28	61	28	28	29	78	25	59	27	19	20	126	40	84	39	42	43
Spain, Castile-La Mancha *	229	148	81							13	6	9	6	4	5	88	38	50	34	38	47	55	24	38	26	17	21	73	32	51	34	22	27
Spain, Catalonia †	1359	902	457	15	1	9	1	6	1	118	9	72	8	46	10	392	29	247	27	145	32	391	29	276	31	115	25	442	33	298	33	145	32
Spain, Community of Madrid	691	455	236	5	1	4	1	1	0	95	14	56	12	39	17	207	30	127	28	80	34	177	26	131	29	46	19	207	30	137	30	70	30
Spain, Extremadura	151	94	57	1	1	1	1	0	0	18	12	9	10	9	16	45	30	23	24	22	39	40	26	27	29	13	23	47	31	34	36	13	23
Spain, Galicia	412	277	135	4	1	3	1	1	1	25	6	12	4	13	10	143	35	95	34	48	36	113	27	75	27	38	28	127	31	92	33	35	26
Spain, La Rioja	40	26	14	0	0	0	0	0	0	3	8	2	8	1	7	13	33	10	38	3	21	10	25	5	19	5	36	14	35	9	35	5	36
Spain, Murcia	233	156	77	1	0	0	0	1	1	22	9	10	6	12	16	68	29	46	29	22	29	66	28	48	31	18	23	76	33	52	33	24	31
Spain, Navarre *	84	55	29							13	15	9	16	4	14	30	36	18	33	12	41	22	26	13	24	9	31	19	23	15	27	4	14
Spain, Valencian region	745	487	258	7	1	5	1	2	1	60	8	41	8	19	7	220	30	140	29	80	31	225	30	147	30	78	30	233	31	154	32	79	31
Sweden	1033	689	344	21	2	13	2	8	2	118	11	83	12	35	10	292	28	190	28	102	30	291	28	185	27	106	31	311	30	218	32	93	27
Switzerland †	882	584	298	15	2	12	2	3	1	87	10	48	8	39	13	248	28	165	28	83	28	207	23	131	22	76	26	325	37	227	39	98	33
the Netherlands &	1829	1183	646	22	1	13	1	9	1	248	14	164	14	84	13	588	32	373	32	215	33	540	30	336	28	204	32	431	24	297	25	134	21
United Kingdom, England † ¶ #	6227	4071	2156	144	2	80	2	64	3	1030	17	655	16	375	17	2246	36	1434	35	811	38	1496	24	981	24	515	24	1312	21	921	23	391	18
United Kingdom, Northern Ireland † #	194	125	69							33	17	24	19	9	13	70	36	44	35	26	38	41	21	23	18	18	26	45	23	32	26	13	19
United Kingdom, Scotland †	604	344	245	8	1	6	2	2	1	102	17	65	19	37	15	234	39	141	41	93	38	147	24	74	22	73	30	97	16	58	17	40	16
United Kingdom, Wales † #	407	250	157	6	1	6	2	0	0	82	20	48	19	34	22	132	32	70	28	62	39	87	21	59	24	28	18	100	25	66	26	34	22

Categories may not add up because of missing values or rounding
§ The incidence is underestimated by approximately 2% due to one haemodialysis centre not submitting data
** Patients younger than 20 years of age are not reported*
† The incident counts at day 91 are estimated (see methods)
& The incidence is underestimated by approximately 8%
¶ The incidence is underestimated by approximately 7.5% due to one centre not providing data and patients opting out of data sharing for research purposes
# Values based on 1 to 5 patients are suppressed

Table B.3.2
Incidence per million (age-related) population by age and sex, unadjusted
at day 91

	All			0-19			20-44			45-64			65-74			75+		
	All	Male	Female	All	Male	Female	All	Male	Female	All	Male	Female	All	Male	Female	All	Male	Female
	Pmp	Pmp	Pmp	Pmarp	Pmarp	Pmarp	Pmarp	Pmarp	Pmarp	Pmarp	Pmarp	Pmarp	Pmarp	Pmarp	Pmarp	Pmarp	Pmarp	Pmarp
Austria	110.3	147.1	74.6	3.5	4.6	2.4	37.2	39.8	34.4	119.7	168.9	71.2	299.5	421.7	192.4	350.7	564.5	205.6
Belgium, Dutch-speaking *	157.8	198.3	118.1				35.8	39.3	32.1	124.4	153.5	94.8	400.6	543.6	264.3	690.2	1002.5	464.8
Belgium, French-speaking *	171.4	216.0	128.3				40.2	52.1	28.2	158.8	204.7	113.1	511.5	626.0	412.0	843.2	1391.2	500.8
Bosnia and Herzegovina	106.8	136.8	77.8	6.4	7.4	5.2	31.1	43.4	18.3	137.4	189.2	88.0	438.7	636.1	284.7	312.5	386.4	266.1
Denmark	114.0	145.1	83.2	7.0	6.0	7.9	47.4	60.4	33.9	149.7	175.6	123.8	253.7	346.4	166.9	320.3	490.9	187.7
Estonia †	82.0	93.2	72.0	6.9	0	14.1	30.1	26.8	33.8	126.6	138.3	115.6	244.6	390.5	146.4	116.6	182.2	89.7
France (17 of 18 regions) †	151.1	207.9	97.8	7.8	10.4	5.1	50.0	66.3	34.1	155.4	205.8	107.2	376.2	553.8	222.3	531.6	896.7	291.5
Greece	252.7	351.2	158.4	6.2	10.0	2.1	45.0	57.7	31.9	176.7	258.0	99.3	588.6	869.3	340.4	1042.5	1649.5	608.7
Iceland	78.5	117.0	37.7	32.1	41.6	22.1	49.6	80.2	15.1	111.1	152.4	68.0	120.3	240.8	0	249.3	362.4	153.5
Montenegro * †	55.6	67.8	43.8				27.2	43.9	9.8	53.8	76.7	32.2	214.1	279.9	159.1	173.7	123.1	206.3
Norway	96.6	133.4	59.1	4.0	6.3	1.7	31.5	37.7	24.9	116.6	164.6	66.7	274.6	368.8	182.4	331.9	552.3	159.6
Romania	158.2	196.7	121.9	8.7	9.4	8.0	56.2	73.1	38.2	217.9	283.1	154.6	427.0	562.1	326.0	351.8	554.7	240.5
Serbia	71.9	96.0	48.2	6.4	7.8	5.0	28.6	40.4	16.4	93.7	115.6	71.9	174.6	269.9	95.2	147.1	228.4	90.8
Spain, Andalusia	145.3	188.7	103.1	6.3	12.3	0	48.7	66.0	30.9	146.5	178.3	115.4	425.4	592.4	275.8	514.0	816.0	313.3
Spain, Aragon	138.5	205.0	73.6	20.2	31.4	8.3	50.8	69.5	31.2	125.5	170.4	79.6	401.3	607.0	214.8	345.5	681.0	119.9
Spain, Asturias	182.9	254.2	117.9	0	0	0	31.2	31.1	31.3	151.6	186.2	118.5	479.0	737.7	256.5	437.2	786.0	223.4
Spain, Basque country	124.7	169.2	82.6	2.5	0	5.2	53.8	54.8	52.9	142.4	173.0	112.3	282.5	428.0	154.7	275.2	542.2	106.1
Spain, Canary Islands	178.3	244.0	114.1	2.6	0	5.4	62.0	69.0	55.0	182.4	257.6	106.9	564.3	808.3	339.7	570.9	943.3	308.0
Spain, Cantabria *	144.8	182.7	109.2				61.4	85.4	37.1	128.6	130.3	126.8	354.3	450.0	268.6	394.5	707.8	197.6
Spain, Castile and León * †	132.3	185.8	80.5				32.9	39.3	26.1	119.1	162.3	75.4	271.6	415.2	131.0	377.2	621.8	210.7
Spain, Castile-La Mancha *	110.7	142.7	78.5				20.4	27.3	13.0	140.8	156.9	124.0	283.0	400.2	171.0	355.8	604.5	182.1
Spain, Catalonia †	174.4	235.3	115.5	9.7	11.3	8.0	47.8	57.2	38.0	172.6	217.4	127.8	517.5	788.6	283.4	593.3	1008.1	321.3
Spain, Community of Madrid	107.8	148.3	70.6	4.0	6.2	1.6	45.7	54.7	36.9	109.0	139.1	81.1	298.7	496.8	139.9	355.8	614.0	195.1
Spain, Extremadura	143.2	180.3	106.9	5.3	10.3	0	57.5	56.4	58.6	138.2	140.2	136.1	365.8	503.1	233.5	400.4	725.1	184.4
Spain, Galicia	152.8	213.5	96.5	9.5	13.9	4.9	34.0	32.5	35.6	171.1	232.4	112.5	344.3	486.6	218.3	335.6	616.3	152.8
Spain, La Rioja	124.6	164.1	86.1	0	0	0	32.4	42.9	21.7	132.9	202.9	61.8	293.9	300.8	287.4	394.0	622.4	237.3
Spain, Murcia	152.1	203.3	100.7	2.9	0	6.1	43.7	38.6	49.2	154.2	206.3	101.0	514.1	790.1	266.2	641.5	1079.0	341.5
Spain, Navarre *	125.7	166.3	85.9				65.6	89.7	40.9	151.0	179.1	122.3	326.0	398.9	257.9	277.1	539.9	98.1
Spain, Valencian region	146.1	194.0	99.7	7.2	9.9	4.2	38.6	52.1	24.8	142.1	181.3	103.1	425.3	592.8	277.5	476.4	774.3	272.2
Sweden	98.5	130.5	66.1	8.6	10.4	6.8	35.1	47.8	21.5	114.0	146.6	80.6	271.5	351.5	194.3	293.1	462.9	157.6
Switzerland †	101.5	135.3	68.2	8.7	13.5	3.6	30.3	33.1	27.4	102.5	136.0	68.8	246.4	326.1	173.0	396.5	658.3	206.4
the Netherlands	112.3	146.2	78.9	6.4	7.4	5.4	48.1	63.0	32.9	133.4	169.6	97.4	299.7	380.0	222.4	291.9	462.5	160.6
United Kingdom, England † #	117.9	157.3	80.0	11.8	12.8	10.7	59.5	77.2	42.6	166.5	216.3	118.3	294.9	402.4	195.4	275.6	444.9	145.4
United Kingdom, Northern Ireland † #	101.4	133.0	70.7				55.1	81.1	29.7	140.8	180.1	103.1	226.9	261.5	193.5	290.3	482.8	145.7
United Kingdom, Scotland †	110.9	130.1	87.3	7.1	10.4	3.6	59.4	77.6	42.1	156.5	194.7	120.6	245.2	257.5	233.9	195.5	272.5	138.6
United Kingdom, Wales † #	130.0	162.9	98.3	9.2	17.8	0	86.7	103.3	70.4	160.9	175.8	146.7	245.3	344.7	152.0	312.0	468.9	189.2

** Patients younger than 20 years of age are not reported*
† The incident counts at day 91, on which the data presented in this table are based, are estimated (see methods)
# Values based on 1 to 5 patients are suppressed

Table B.3.3
Sex, mean age, and median age of incident patients
at day 91

	All						Male						Female					
	%	Mean (years)	SD	Median (years)	P25	P75	%	Mean (years)	SD	Median (years)	P25	P75	%	Mean (years)	SD	Median (years)	P25	P75
Austria	100	65.3	15.4	67.6	57.2	77.1	65.7	65.5	15.0	67.3	57.6	76.9	34.3	64.8	16.0	68.2	54.4	77.3
Belgium, Dutch-speaking *	100	70.4	14.5	73.6	63.6	80.8	62.3	70.6	13.8	73.4	64.0	80.3	37.7	70.2	15.5	73.8	62.2	81.5
Belgium, French-speaking *	100	68.8	14.0	71.9	60.9	78.5	61.9	68.7	14.0	72.2	60.2	78.6	38.1	69.0	14.1	71.6	62.6	78.3
Bosnia and Herzegovina	100	63.2	14.2	65.6	56.7	72.6	62.9	62.2	14.1	64.4	56.7	71.5	37.1	64.8	14.3	68.7	57.3	74.6
Denmark	100	62.9	16.4	65.8	52.6	76.0	63.3	63.7	16.4	67.4	53.9	76.4	36.7	61.6	16.3	63.3	51.0	74.8
Estonia †	100	61.0	15.3	62.6	54.7	71.9	53.9	63.7	11.8	65.1	56.3	72.2	46.1	57.6	18.3	58.7	51.9	69.5
France (17 of 18 regions) †	100	66.8	16.0	70.2	58.2	78.1	66.6	67.1	15.8	70.6	59.0	78.1	33.4	66.2	16.2	69.6	56.9	77.9
Greece	100	71.5	13.9	74.1	64.5	82.1	68.0	71.1	13.9	73.4	64.1	81.4	32.0	72.6	13.9	75.2	65.3	83.1
Iceland	100	54.1	22.0	58.1	43.2	71.8	76.7	54.0	20.7	59.2	38.1	71.8	23.3	54.4	27.7	57.1	43.8	82.2
Montenegro * †	100	60.0	15.2	66.4	47.4	70.2	60.2	57.7	15.5	65.8	45.0	69.8	39.8	63.8	14.9	68.8	53.1	71.4
Norway	100	64.2	15.3	67.3	54.2	76.0	69.6	64.5	15.4	67.7	54.2	76.2	30.4	63.4	15.2	66.4	54.4	75.2
Romania	100	62.3	14.4	64.7	54.3	72.3	60.4	61.4	14.3	63.5	53.0	71.7	39.6	63.7	14.3	66.6	55.8	73.1
Serbia	100	61.7	15.1	64.7	54.2	72.0	64.9	61.8	15.2	66.3	53.5	72.2	34.4	61.4	15.1	63.4	55.1	71.9
Spain, Andalusia	100	64.9	15.5	68.0	55.9	76.7	64.0	64.4	16.1	68.1	54.7	76.7	36.0	65.6	14.2	67.8	57.1	77.0
Spain, Aragon	100	64.7	16.6	68.0	58.4	76.6	73.1	64.9	17.1	69.3	54.6	76.8	26.9	64.3	15.3	66.1	60.3	73.4
Spain, Asturias	100	68.4	11.7	70.9	61.9	76.4	66.3	69.4	11.2	72.1	64.5	76.4	33.7	66.4	12.6	68.9	57.7	76.9
Spain, Basque country	100	63.5	15.2	66.3	54.3	75.4	65.9	65.6	14.5	68.0	57.0	76.1	34.1	59.5	15.9	60.0	49.7	72.5
Spain, Canary Islands	100	64.1	14.6	66.8	55.5	75.0	67.6	65.3	13.6	67.6	56.7	75.2	32.4	61.7	16.4	66.7	51.7	74.2
Spain, Cantabria *	100	65.7	14.6	69.6	56.3	75.9	61.2	65.9	15.6	70.6	55.0	77.4	38.8	65.3	13.2	68.6	57.9	74.9
Spain, Castile and León * †	100	69.0	13.2	71.6	60.9	79.1	69.1	69.2	12.8	71.1	60.9	78.9	30.9	68.5	14.1	73.4	59.7	79.5
Spain, Castile-La Mancha *	100	66.5	13.6	67.7	58.4	77.5	64.6	67.6	13.6	68.9	59.9	77.8	35.4	64.4	13.4	64.4	53.2	77.4
Spain, Catalonia †	100	66.2	15.2	69.1	56.8	77.6	66.4	66.7	14.7	69.5	58.2	77.6	33.6	65.1	16.1	68.1	55.5	77.7
Spain, Community of Madrid	100	64.2	16.2	66.8	54.6	76.6	65.8	64.8	16.1	68.3	56.1	76.4	34.2	63.2	16.3	64.7	51.9	77.8
Spain, Extremadura	100	65.4	15.4	69.1	58.0	77.0	62.3	66.8	15.5	71.0	59.9	78.0	37.7	63.1	15.1	61.9	52.1	74.6
Spain, Galicia	100	66.1	14.1	68.1	57.3	76.6	67.2	67.0	13.9	68.9	58.0	77.5	32.8	64.3	14.6	66.2	54.0	75.2
Spain, La Rioja	100	66.2	13.5	66.7	57.3	77.5	65.0	65.6	12.9	66.2	56.9	77.3	35.0	67.3	14.9	68.5	61.4	81.3
Spain, Murcia	100	65.8	14.6	69.9	56.7	77.6	67.0	67.5	12.2	69.9	59.5	77.9	33.0	62.5	18.1	70.2	51.4	77.3
Spain, Navarre *	100	62.8	15.6	64.7	52.7	74.1	65.5	63.9	15.8	65.9	52.9	76.7	34.5	60.7	15.2	64.4	52.1	72.5
Spain, Valencian region	100	66.4	14.5	69.1	58.3	77.0	65.4	66.6	14.8	69.5	57.9	77.3	34.6	66.2	14.0	68.5	58.7	76.6
Sweden	100	64.3	16.8	68.5	55.9	76.5	66.7	64.5	16.9	68.9	55.0	77.0	33.3	64.0	16.6	67.7	56.9	75.7
Switzerland †	100	66.3	17.2	70.4	57.2	78.9	66.2	66.6	17.6	71.8	57.6	79.2	33.8	65.5	16.2	69.5	56.6	77.9
the Netherlands	100	62.6	15.9	66.2	54.1	74.5	64.7	62.8	16.0	66.4	54.0	75.0	35.3	62.2	15.9	65.7	54.1	73.5
United Kingdom, England †	100	60.3	17.1	63.0	49.6	73.5	65.4	61.0	17.0	63.7	50.1	74.4	34.6	58.8	17.1	61.5	48.3	72.1
United Kingdom, Northern Ireland †	100	61.8	17.5	65.7	51.8	76.0	64.5	62.9	17.6	68.1	52.0	77.9	35.5	59.9	17.2	64.2	49.7	70.8
United Kingdom, Scotland †	100	58.8	16.2	60.9	50.3	71.0	57.0	58.0	16.9	59.6	49.0	71.4	40.5	59.7	15.3	63.0	51.3	70.6
United Kingdom, Wales †	100	60.8	17.5	63.5	46.6	75.3	61.4	62.0	17.9	66.2	49.7	75.8	38.6	58.9	16.8	60.2	46.4	74.3

Categories may not add up because of missing values or rounding
** Patients younger than 20 years of age are not reported*
† The incident counts at day 91, on which the data presented in this table are based, are estimated (see methods)

Table B.3.4

Incidence per million population, adjusted

at day 91, adjusted for age and sex

	All	Male	Female
	Pmp	Pmp	Pmp
Austria	112.5	150.3	76.3
Belgium, Dutch-speaking *	157.2	194.4	121.6
Belgium, French-speaking *	198.9	253.2	147.1
Bosnia and Herzegovina	134.8	179.0	92.6
Denmark	116.1	146.1	87.3
Estonia †	88.9	105.4	73.1
France (17 of 18 regions) †	156.0	212.7	101.9
Greece	231.9	318.9	148.7
Iceland	89.8	133.4	48.1
Montenegro * †	66.4	81.5	51.9
Norway	103.9	142.6	66.8
Romania	166.3	212.3	122.3
Serbia	69.5	93.6	46.4
Spain, Andalusia	156.6	204.0	111.3
Spain, Aragon	134.4	197.4	74.2
Spain, Asturias	151.4	207.1	98.1
Spain, Basque country	117.1	156.6	79.4
Spain, Canary Islands	190.1	260.2	123.1
Spain, Cantabria *	136.3	172.7	101.5
Spain, Castile and León * †	111.3	152.3	72.2
Spain, Castile-La Mancha *	113.4	146.8	81.5
Spain, Catalonia †	184.8	250.6	122.0
Spain, Community of Madrid	117.6	162.2	75.0
Spain, Extremadura	139.2	174.1	105.9
Spain, Galicia	134.1	182.6	87.7
Spain, La Rioja	120.8	151.7	91.4
Spain, Murcia	178.7	245.5	114.9
Spain, Navarre *	126.4	165.9	88.6
Spain, Valencian region	148.2	197.2	101.4
Sweden	101.4	133.0	71.2
Switzerland †	105.1	139.5	72.3
the Netherlands	113.3	145.8	82.1
United Kingdom, England †	126.2	167.0	87.2
United Kingdom, Northern Ireland †	111.1	143.8	79.7
United Kingdom, Scotland †	109.0	130.4	88.6
United Kingdom, Wales †	130.6	161.3	101.3

** Patients younger than 20 years of age are not reported*

† The incident counts at day 91, on which the data presented in this table are based, are estimated (see methods)

Table B.3.5.A
Incidence per million population and percentages by primary renal disease (1995 PRD codes), unadjusted
at day 91

	All		GN		PN		PKD		DM						HT		RVD		Misc		Unkn		Missing	
									Type 1		Type 2		Both											
	Pmp	%	Pmp	%	Pmp	%	Pmp	%	Pmp	%	Pmp	%	Pmp	%	Pmp	%	Pmp	%	Pmp	%	Pmp	%	Pmp	%
Austria	110.3	100	11.3	10.2	2.8	2.6	6.1	5.6	3.5	3.2	22.3	20.2	25.8	23.4	16.6	15.0	0.9	0.8	24.4	22.1	22.0	20.0	0.3	0.3
Belgium, Dutch-speaking *	157.8	100	14.1	8.9	4.9	3.1	7.0	4.4	3.6	2.3	30.5	19.3	34.1	21.6	15.4	9.8	6.5	4.1	53.5	33.9	22.4	14.2	0	0
Belgium, French-speaking *	171.4	100	16.4	9.6	6.1	3.6	11.0	6.4	3.2	1.9	33.1	19.3	36.3	21.2	32.6	19.1	1.4	0.8	42.4	24.7	25.1	14.7	0	0
Bosnia and Herzegovina	106.8	100	9.3	8.8	6.2	5.8	4.5	4.2	6.5	6.1	25.5	23.9	32.0	30.0	15.3	14.3	1.1	1.1	15.3	14.3	22.9	21.5	0	0
Denmark	114.0	100	15.2	13.4	6.6	5.8	8.8	7.7	7.5	6.5	24.2	21.2	31.7	27.8	13.9	12.2	2.7	2.4	20.2	17.7	14.9	13.1	0	0
Estonia †	82.0	100	13.3	16.3	4.4	5.4	9.6	11.7					14.8	18.1	25.0	30.4	0.7	0.9	14.1	17.2	0	0	0	0
France (17 of 18 regions) †	151.1	100	17.1	11.3	7.7	5.1	10.1	6.7					33.6	22.3	35.8	23.7	1.6	1.1	21.9	14.5	23.3	15.4	0	0
Greece	252.7	100	17.4	6.9	13.2	5.2	9.5	3.8	2.4	0.9	56.6	22.4	59.0	23.4	25.0	9.9	5.7	2.2	29.4	11.6	93.4	37.0	0	0
Iceland	78.5	100	5.2	6.7	7.9	10.0	5.2	6.7	2.6	3.3	15.7	20.0	18.3	23.3	20.9	26.7	0	0	18.3	23.3	2.6	3.3	0	0
Montenegro * †	55.6	100	0	0	1.6	2.9	0	0	1.6	2.9	12.4	22.3	14.0	25.2	23.2	41.7	0	0	13.5	24.3	3.2	5.8	0	0
Norway	96.6	100	13.6	14.0	4.2	4.4	9.7	10.1	5.1	5.3	11.2	11.6	16.3	16.9	27.5	28.5	0.5	0.6	18.9	19.5	3.8	4.0	2.0	2.1
Romania	158.2	100	19.8	12.5	5.9	3.7	3.9	2.5					17.1	10.8	8.7	5.5	1.2	0.7	24.9	15.8	76.7	48.5	0	0
Serbia	71.9	100	6.0	8.3	6.0	8.3	2.8	3.9					13.9	19.4	24.4	34.0	0.8	1.1	13.2	18.3	1.7	2.4	3.1	4.4
Spain, Andalusia	145.3	100	17.0	11.7	7.7	5.3	9.7	6.7					37.1	25.5	16.0	11.0	0.4	0.2	25.1	17.2	32.3	22.2	0	0
Spain, Aragon	138.5	100	22.3	16.1	8.2	5.9	9.7	7.0	5.2	3.8	24.6	17.7	29.8	21.5	31.3	22.6	0	0	22.3	16.1	14.9	10.8	0	0
Spain, Asturias	182.9	100	24.9	13.6	8.9	4.9	14.9	8.2	3.0	1.6	41.8	22.8	44.7	24.5	34.8	19.0	2.0	1.1	26.8	14.7	25.8	14.1	0	0
Spain, Basque country	124.7	100	13.6	10.9	8.1	6.5	12.2	9.8	0.5	0.4	25.3	20.3	25.8	20.7	18.5	14.9	0	0	22.1	17.8	24.4	19.6	0	0
Spain, Canary Islands	178.3	100	15.9	8.9	5.0	2.8	15.0	8.4	10.0	5.6	45.9	25.8	55.9	31.4	11.8	6.6	0.5	0.3	35.0	19.6	39.1	21.9	0	0
Spain, Cantabria *	144.8	100	32.4	22.4	10.2	7.1	22.2	15.3	3.4	2.4	22.2	15.3	25.6	17.6	10.2	7.1	3.4	2.4	11.9	8.2	8.5	5.9	20.4	14.1
Spain, Castile and León * † ‡	132.3	100	16.4	12.4	8.9	6.7	8.0	6.1	3.0	2.2	29.1	22.0	32.0	24.2	20.5	15.5	0.4	0.3	28.5	21.5	17.6	13.3	0	0
Spain, Castile-La Mancha *	110.7	100	8.7	7.9	6.3	5.7	8.2	7.4					26.1	23.6	8.7	7.9	1.5	1.3	22.7	20.5	28.5	25.8	0	0
Spain, Catalonia †	174.4	100	16.9	9.7	8.4	4.8	12.2	7.0	2.6	1.5	30.2	17.3	33.5	19.2	10.4	6.0	3.3	1.9	15.3	8.8	36.2	20.7	38.3	22.0
Spain, Community of Madrid	107.8	100	10.3	9.6	2.5	2.3	6.7	6.2	1.6	1.4	20.0	18.5	21.5	20.0	7.5	6.9	1.1	1.0	13.1	12.2	15.4	14.3	29.6	27.5
Spain, Extremadura	143.2	100	22.8	15.9	11.4	7.9	5.7	4.0					28.4	19.9	16.1	11.3	4.7	3.3	24.6	17.2	20.9	14.6	8.5	6.0
Spain, Galicia	152.8	100	20.0	13.1	5.2	3.4	15.2	10.0	7.4	4.9	28.2	18.4	35.6	23.3	16.3	10.7	0	0	24.9	16.3	35.6	23.3	0	0
Spain, La Rioja	124.6	100	9.3	7.5	0	0	12.5	10.0	3.1	2.5	12.5	10.0	15.6	12.5	12.5	10.0	0	0	53.0	42.5	21.8	17.5	0	0
Spain, Murcia	152.1	100	21.5	14.2	3.9	2.6	11.8	7.7	3.9	2.6	36.6	24.0	40.5	26.6	18.9	12.4	0	0	30.0	19.7	25.5	16.7	0	0
Spain, Navarre *	125.7	100	9.0	7.1	1.5	1.2	18.0	14.3	12.0	9.5	23.9	19.0	35.9	28.6	21.0	16.7	0	0	23.9	19.0	16.5	13.1	0	0
Spain, Valencian region	146.1	100	16.7	11.4	6.7	4.6	7.7	5.2					26.3	18.0	23.5	16.1	1.2	0.8	34.1	23.4	30.0	20.5	0	0
Sweden ‡	98.5	100	13.8	14.0	3.6	3.7	7.2	7.4	5.1	5.2	18.2	18.5	23.4	23.7	17.6	17.9	0.9	0.9	23.2	23.5	8.8	8.9	0	0
Switzerland †	101.5	100	14.8	14.6	2.3	2.3	6.2	6.1	2.5	2.5	18.8	18.5	21.3	21.0	21.0	20.7	3.2	3.2	25.1	24.7	7.5	7.4	0	0
the Netherlands	112.3	100	12.3	11.0	4.3	3.8	6.5	5.8	2.6	2.4	19.6	17.4	22.2	19.8	18.0	16.0	4.3	3.8	27.4	24.4	16.0	14.3	1.2	1.0
United Kingdom, England † #	117.9	100	12.7	10.7	5.6	4.8	6.1	5.2					29.9	25.4	8.0	6.8	1.0	0.8	21.0	17.9	15.4	13.1	18.1	15.4
United Kingdom, Northern Ireland † #	101.4	100	14.1	13.9	9.2	9.1	6.8	6.7					17.6	17.4					19.7	19.4	9.9	9.8	20.2	20.0
United Kingdom, Scotland †	110.9	100	13.2	11.9	6.9	6.2	7.3	6.6					25.6	23.1	5.0	4.5	1.8	1.7	24.8	22.4	13.2	11.9	13.0	11.7
United Kingdom, Wales † #	130.0	100	17.7	13.6	8.8	6.8	7.3	5.7					38.2	29.4	5.7	4.4	0	0	29.4	22.6	16.5	12.7	6.3	4.8

Abbreviations used: GN: glomerulonephritis/sclerosis; PN: pyelonephritis; PKD: polycystic kidneys, adult type; DM: diabetes mellitus; HT: hypertension; RVD: renal vascular disease; Misc: miscellaneous; Unkn: unknown
Categories may not add up because of rounding or a limited number of cases (<10%) with diabetes mellitus type unknown; When cells are left empty, (complete) data are unavailable
** Patients younger than 20 years of age are not reported*
† The incident counts at day 91, on which the data presented in this table are based, are estimated (see methods)
‡ Mapping the 2018 PRD codes to the old PRD codes results in a different distribution of PRD groups
# Values based on 1 to 5 patients are suppressed

Table B.3.5.B

Incidence per million population and percentages by primary renal disease (2012/2018 PRD codes), unadjusted

at day 91

	All		Glomerular disease		Tubulo-interstitial disease		DM		HT / RVD		Other systemic diseases		Familial/ hereditary nephropathies		Misc		Unkn		Missing	
	**Pmp**	**%**	**Pmp**	**%**	**Pmp**	**%**	**Pmp**	**%**	**Pmp**	**%**	**Pmp**	**%**	**Pmp**	**%**	**Pmp**	**%**	**Pmp**	**%**	**Pmp**	**%**
Austria	110.3	100	15.5	14.0	6.3	5.7	25.8	23.4	20.1	18.2	6.0	5.5	9.0	8.1	10.1	9.2	17.3	15.7	0.3	0.3
Belgium, Dutch-speaking *	157.8	100	17.5	11.1	9.9	6.3	34.1	21.6	35.6	22.5	21.5	13.6	10.1	6.4	18.2	11.5	11.0	6.9	0	0
Belgium, French-speaking *	171.4	100	19.3	11.2	17.2	10.1	36.3	21.2	35.5	20.7	13.2	7.7	15.2	8.9	19.9	11.6	14.8	8.6	0	0
Bosnia and Herzegovina	106.8	100	10.8	10.1	9.3	8.8	31.4	29.4	15.9	14.9	2.3	2.1	8.8	8.2	4.0	3.7	24.4	22.8	0	0
Denmark	114.0	100	20.7	18.1	11.7	10.3	31.7	27.8	18.0	15.8	1.2	1.0	10.8	9.5	5.4	4.8	14.6	12.8	0	0
Iceland	78.5	100	10.5	13.3	15.7	20.0	18.3	23.3	20.9	26.7	0	0	10.5	13.3	0	0	2.6	3.3	0	0
Montenegro * †	55.6	100	1.6	2.9	1.6	2.9	14.0	25.2	23.2	41.7	2.7	4.9	4.3	7.8	4.9	8.7	3.2	5.8	0	0
Norway	96.6	100	16.5	17.1	8.1	8.3	16.3	16.9	29.3	30.4	5.5	5.7	12.1	12.5	3.8	4.0	2.9	3.0	2.0	2.1
Spain, Andalusia	145.3	100	20.6	14.2	15.0	10.3	37.1	25.5	19.0	13.1	4.6	3.1	13.3	9.2	6.1	4.2	29.6	20.4	0	0
Spain, Aragon	138.5	100	23.8	17.2	12.7	9.1	29.8	21.5	32.8	23.7	6.7	4.8	11.2	8.1	10.4	7.5	11.2	8.1	0	0
Spain, Asturias	182.9	100	28.8	15.8	16.9	9.2	44.7	24.5	38.8	21.2	4.0	2.2	18.9	10.3	5.0	2.7	25.8	14.1	0	0
Spain, Basque country	124.7	100	15.4	12.3	16.3	13.0	25.8	20.7	20.8	16.7	2.7	2.2	15.4	12.3	5.0	4.0	23.5	18.8	0	0
Spain, Canary Islands	178.3	100	19.1	10.7	15.0	8.4	55.9	31.4	19.6	11.0	7.3	4.1	16.8	9.4	5.5	3.1	39.1	21.9	0	0
Spain, Cantabria *	144.8	100	18.7	12.9	13.6	9.4	25.6	17.6	13.6	9.4	0	0	22.2	15.3	6.8	4.7	20.4	14.1	23.9	16.5
Spain, Castile and León * †	132.3	100	23.6	17.8	14.8	11.1	32.0	24.2	23.0	17.4	7.2	5.4	11.0	8.3	5.8	4.4	15.0	11.4	0	0
Spain, Catalonia †	174.4	100	19.8	11.4	10.0	5.7	32.7	18.8	13.9	7.9	3.0	1.7	15.0	8.6	5.8	3.3	35.8	20.5	38.3	22.0
Spain, Community of Madrid	107.8	100	11.5	10.7	7.5	6.9	21.5	20.0	9.0	8.4	2.2	2.0	7.6	7.1	4.7	4.3	14.0	13.0	29.6	27.5
Spain, Galicia	152.8	100	25.2	16.5	11.9	7.8	35.6	23.3	18.2	11.9	4.1	2.7	16.3	10.7	12.2	8.0	29.3	19.2	0	0
Spain, La Rioja	124.6	100	12.5	10.0	18.7	15.0	15.6	12.5	24.9	20.0	3.1	2.5	18.7	15.0	9.3	7.5	21.8	17.5	0	0
Spain, Murcia	152.1	100	24.8	16.3	9.8	6.4	40.5	26.6	32.6	21.5	3.3	2.1	12.4	8.2	3.9	2.6	24.8	16.3	0	0
Spain, Navarre *	125.7	100	16.5	13.1	4.5	3.6	35.9	28.6	21.0	16.7	6.0	4.8	18.0	14.3	7.5	6.0	16.5	13.1	0	0
Spain, Valencian region	146.1	100	21.2	14.5	15.1	10.3	26.3	18.0	28.4	19.5	9.8	6.7	11.2	7.7	6.3	4.3	27.9	19.1	0	0
Sweden	98.5	100	17.7	18.0	9.0	9.1	23.4	23.7	21.8	22.2	3.5	3.6	10.2	10.4	7.3	7.5	5.5	5.6	0	0
United Kingdom, England † #	117.9	100	16.4	13.9	8.6	7.3	29.0	24.6	10.6	9.0	3.7	3.1	7.3	6.2	6.4	5.4	13.3	11.3	22.7	19.2
United Kingdom, Northern Ireland † #	101.4	100	12.6	12.4	11.9	11.7	17.6	17.4	7.3	7.2			7.9	7.7	7.9	7.7	6.8	6.7	27.6	27.2
United Kingdom, Scotland †	110.9	100	16.3	14.7	10.5	9.4	25.6	23.1	12.8	11.6	2.5	2.3	9.7	8.8	4.5	4.1	12.3	11.1	16.6	15.0
United Kingdom, Wales † #	130.0	100	21.5	16.5	11.4	8.8	38.5	29.6	17.5	13.4	3.5	2.7	9.3	7.1	9.4	7.2	12.3	9.5	6.6	5.1

Abbreviations used: DM: diabetes mellitus; HT / RVD: hypertension / renal vascular disease; Misc: miscellaneous renal disorders; Unkn: unknown
** Patients younger than 20 years of age are not reported*
† The incident counts at day 91, on which the data presented in this table are based, are estimated (see methods)
# Values based on 1 to 5 patients are suppressed

Table B.3.6.A
Incidence per million population by primary renal disease (1995 PRD codes), adjusted
at day 91, adjusted for age and sex

	All	GN	PN	PKD	DM			HT	RVD	Misc	Unkn	Missing
					Type 1	Type 2	Both					
	Pmp	Pmp	Pmp	Pmp	Pmp	Pmp	Pmp	Pmp	Pmp	Pmp	Pmp	Pmp
Austria	112.5	11.2	3.0	6.3	3.5	23.0	26.5	16.9	1.0	24.9	22.5	0.4
Belgium, Dutch-speaking *	157.2	14.3	4.9	7.3	3.6	30.5	34.1	15.2	6.4	52.6	22.3	0
Belgium, French-speaking *	198.9	18.5	7.1	12.2	3.4	38.9	42.3	39.2	1.6	48.5	29.6	0
Bosnia and Herzegovina	134.8	10.2	8.2	5.6	7.3	32.0	39.3	21.1	1.5	19.1	29.9	0
Denmark	116.1	16.0	6.4	9.3	7.8	24.9	32.7	13.5	2.9	20.3	14.9	0
Estonia †	88.9	14.3	4.8	10.4			15.7	28.2	0.8	14.7	0	0
France (17 of 18 regions) †	156.0	17.9	7.9	10.8			34.8	36.6	1.7	22.5	23.9	0
Greece	231.9	16.8	12.1	9.1	2.3	52.3	54.6	22.5	5.1	27.1	84.5	0
Iceland	89.8	4.9	9.8	6.4	2.5	19.1	21.6	27.0	0	18.3	1.8	0
Montenegro * †	66.4	0	1.8	0	1.4	15.5	16.9	28.3	0	14.9	6.2	0
Norway	103.9	14.4	4.4	10.7	5.3	12.2	17.4	29.7	0.7	20.3	4.1	2.1
Romania	166.3	20.6	6.4	3.9			17.9	9.2	1.3	26.4	80.6	0
Serbia	69.5	5.7	5.8	2.6			13.1	23.6	0.8	12.8	1.8	3.2
Spain, Andalusia	156.6	18.5	8.3	10.2			39.8	17.1	0.4	27.1	35.2	0
Spain, Aragon	134.4	22.0	8.1	9.0	5.2	23.9	29.1	29.7	0	21.8	14.7	0
Spain, Asturias	151.4	21.5	7.5	12.6	3.2	34.3	37.6	27.5	1.5	22.2	21.0	0
Spain, Basque country	117.1	13.3	7.4	11.0	0.4	23.4	23.8	17.5	0	20.9	23.2	0
Spain, Canary Islands	190.1	16.2	5.8	14.2	9.2	50.6	59.8	13.2	0.5	38.6	41.8	0
Spain, Cantabria *	136.3	31.0	9.0	20.6	3.1	21.0	24.1	9.5	3.2	12.0	7.4	19.7
Spain, Castile and León * † ‡	111.3	14.7	7.1	7.3	2.5	24.1	26.7	16.8	0.4	23.9	14.6	0
Spain, Castile-La Mancha *	113.4	8.7	6.3	8.5			26.7	8.3	1.5	23.6	29.7	0
Spain, Catalonia †	184.8	17.7	8.9	12.1	2.6	33.0	36.2	11.1	3.6	15.8	38.8	40.7
Spain, Community of Madrid	117.6	10.6	2.6	6.7	1.8	22.7	24.4	8.2	1.2	14.4	17.1	32.4
Spain, Extremadura	139.2	21.6	11.3	5.3			27.7	15.8	4.2	24.6	20.7	8.0
Spain, Galicia	134.1	18.6	4.3	13.4	7.3	24.9	32.2	13.7	0	21.8	30.1	0
Spain, La Rioja	120.8	9.5	0	11.3	2.9	12.6	15.5	11.8	0	51.3	21.5	0
Spain, Murcia	178.7	23.3	4.2	11.4	3.8	46.0	49.8	24.2	0	36.0	29.8	0
Spain, Navarre *	126.4	9.1	1.6	18.1	11.6	23.9	35.4	21.1	0	24.2	16.9	0
Spain, Valencian region	148.2	16.4	6.7	7.4			26.5	24.4	1.2	34.7	30.9	0
Sweden ‡	101.4	14.1	3.6	7.8	5.5	19.0	24.4	17.9	0.9	23.8	9.0	0
Switzerland †	105.1	15.0	2.4	6.5	2.5	19.7	22.2	21.7	3.3	26.1	8.0	0
the Netherlands	113.3	12.3	4.3	6.8	2.7	19.4	22.1	18.4	4.4	27.6	16.3	1.2
United Kingdom, England † #	126.2	13.3	5.9	6.7			32.4	8.6	1.1	22.5	16.5	19.2
United Kingdom, Northern Ireland † #	111.1	15.1	10.4	7.2			19.3			21.8	11.2	22.4
United Kingdom, Scotland †	109.0	12.8	6.8	7.4			25.7	5.2	1.9	25.0	13.1	11.2
United Kingdom, Wales † #	130.7	18.4	9.1	7.9			37.9	5.8	0	29.0	16.3	6.6

Abbreviations used: GN: glomerulonephritis/sclerosis; PN: pyelonephritis; PKD: polycystic kidneys, adult type; DM: diabetes mellitus; HT: hypertension; RVD: renal vascular disease; Misc: miscellaneous; Unkn: unknown
Categories may not add up because of rounding or a limited number of cases (<10%) with diabetes mellitus type unknown; When cells are left empty, (complete) data are unavailable
** Patients younger than 20 years of age are not reported*
† The incident counts at day 91, on which the data presented in this table are based, are estimated (see methods)
‡ Mapping the 2018 PRD codes to the old PRD codes results in a different distribution of PRD groups
# Values based on 1 to 5 patients are suppressed

Table B.3.6.B

Incidence per million population by primary renal disease (2012/2018 PRD codes), adjusted

at day 91, adjusted for age and sex

	All	Glomerular disease	Tubulo-interstitial disease	DM	HT / RVD	Other systemic diseases	Familial/ hereditary nephropathies	Misc	Unkn	Missing
	Pmp	Pmp	Pmp	Pmp	Pmp	Pmp	Pmp	Pmp	Pmp	Pmp
Austria	112.5	15.4	6.4	26.5	20.6	6.1	9.1	10.3	17.6	0.4
Belgium, Dutch-speaking *	157.2	17.7	9.9	34.1	34.9	21.1	10.5	18.1	11.0	0
Belgium, French-speaking *	198.9	21.6	19.8	42.3	42.5	15.6	16.6	23.2	17.3	0
Bosnia and Herzegovina	134.8	11.8	12.7	38.5	22.0	3.0	10.3	4.8	31.7	0
Denmark	116.1	21.6	11.6	32.7	17.7	1.2	11.3	5.4	14.6	0
Iceland	89.8	10.4	18.1	21.6	27.0	0	11.0	0	1.8	0
Montenegro * †	66.4	1.8	1.8	16.9	28.3	3.2	4.9	6.7	4.5	0
Norway	103.9	17.7	8.6	17.4	31.9	5.7	13.1	4.2	3.1	2.1
Spain, Andalusia	156.6	22.3	16.0	39.8	20.4	5.0	14.1	6.8	32.2	0
Spain, Aragon	134.4	23.3	12.3	29.1	31.2	6.8	10.6	10.2	10.9	0
Spain, Asturias	151.4	24.8	14.5	37.6	30.6	3.0	16.2	3.7	21.0	0
Spain, Basque country	117.1	15.2	15.1	23.8	19.7	2.6	13.9	4.5	22.4	0
Spain, Canary Islands	190.1	19.4	16.7	59.8	22.1	8.3	16.1	5.9	41.8	0
Spain, Cantabria *	136.3	18.4	12.1	24.1	12.6	0	21.1	6.5	18.5	23.0
Spain, Castile and León * †	111.3	20.5	12.0	26.7	18.9	6.1	10.1	5.0	12.3	0
Spain, Catalonia †	184.8	20.7	10.4	35.4	14.8	3.2	14.8	6.3	38.5	40.7
Spain, Community of Madrid	117.6	12.0	8.1	24.4	10.0	2.4	7.6	5.3	15.3	32.4
Spain, Galicia	134.1	22.9	10.6	32.2	15.4	3.4	14.4	10.8	24.4	0
Spain, La Rioja	120.8	12.2	19.2	15.5	24.3	3.4	16.8	7.9	21.5	0
Spain, Murcia	178.7	26.7	10.8	49.8	41.5	3.8	12.2	4.6	29.2	0
Spain, Navarre *	126.4	16.4	4.7	35.4	21.1	6.1	18.1	7.7	16.9	0
Spain, Valencian region	148.2	21.0	15.4	26.5	29.5	10.0	10.8	6.4	28.6	0
Sweden	101.4	18.2	9.1	24.4	22.2	3.6	10.7	7.4	5.8	0
United Kingdom, England † #	126.2	17.2	9.0	31.4	11.4	4.0	7.9	6.9	14.1	24.2
United Kingdom, Northern Ireland † #	111.1	13.5	12.7	19.3	8.6		8.3	8.8	7.6	30.5
United Kingdom, Scotland †	109.0	16.0	10.5	25.7	13.2	2.5	9.8	4.4	12.2	14.9
United Kingdom, Wales † #	130.7	22.2	11.5	38.2	17.1	3.6	9.8	9.3	12.3	6.9

Abbreviations used: DM: diabetes mellitus; HT / RVD: hypertension / renal vascular disease; Misc: miscellaneous renal disorders; Unkn: unknown
** Patients younger than 20 years of age are not reported*
† The incident counts at day 91, on which the data presented in this table are based, are estimated (see methods)
# Values based on 1 to 5 patients are suppressed

Table B.3.7

Incident counts by treatment modality

at day 91

	All	Haemodialysis						Peritoneal dialysis				Kidney transplant				Unkn	Missing
		HD hospital/ centre	HD home	HD type Unkn	HF	HDF	Total HD/ HF/HDF	APD	CAPD	PD type Unkn	Total PD	Living donor	Deceased donor	Tx type Unkn	Total Tx		
	N	N	N	N	N	N	N	N	N	N	N	N	N	N	N	N	N
Austria §	971	745	0	0	1	114	860	27	52	1	80	18	13	0	31	0	0
Belgium, Dutch-speaking *	1065	619	10	0	0	305	934	59	52	0	111	10	10	0	20	0	0
Belgium, French-speaking *	845	404	11	0	12	308	735	49	42	0	91	4	15	0	19	0	0
Bosnia and Herzegovina	377	303	0	0	0	58	361	0	15	0	15	1	0	0	1	0	0
Denmark	673	392	0	0			392	92	115	10	217	30	33	0	63	1	0
Estonia †	111	94	0	0	0	0	94	13	0	0	13	1	3	0	4	0	0
France (17 of 18 regions) †	10216	6290	20	9	16	2353	8689	289	743	13	1045	213	260	0	473	9	0
Greece	2637	2346	1	0	0	146	2493	50	74	0	124	19	1	0	20	0	0
Iceland	30	21	0	0			21	0	5	0	5	3	1	0	4	0	0
Montenegro * †	34	34	0	0	0	0	34	0	0	0	0	0	0	0	0	0	0
Norway	527	337	0	0			337			143	143	17	30	0	47	0	0
Romania ‖	3014	2922	0	0	0	35	2957	1	35	0	36	18	3	0	21	0	0
Serbia	459	395	0	4	0	8	407	4	45	0	49	3	0	0	3	0	0
Spain, Andalusia	1241	968	12	0			980			192	192	24	45	0	69	0	0
Spain, Aragon	186	117	0	0		27	144	10	19	0	29	4	9	0	13	0	0
Spain, Asturias	184	141	0	0		1	142	4	32	0	36	1	5	0	6	0	0
Spain, Basque country	276	185	7	0			192	1	66	0	67	10	7	0	17	0	0
Spain, Canary Islands	392	273	2	0	0	0	275	0	0	111	111	2	4	0	6	0	0
Spain, Cantabria *	85	62	1	0			63	3	5	0	8	1	13	0	14	0	0
Spain, Castile and León * †	314	244	3	0			247	18	37	0	55	5	7	0	12	0	0
Spain, Castile-La Mancha *	229	185	1	0			186	5	37	0	42	0	1	0	1	0	0
Spain, Catalonia †	1359	418	9	0		593	1020	35	135	0	170	85	85	0	169	0	0
Spain, Community of Madrid	691	480	4	0		57	541	33	73	0	106	19	25	0	44	0	0
Spain, Extremadura	151	132	4	0			136	6	6	0	12	2	1	0	3	0	0
Spain, Galicia	412	313	4	0	0	0	317	5	71	0	76	15	4	0	19	0	0
Spain, La Rioja	40	29	1	0	0	0	30	3	5	0	8	2	0	0	2	0	0
Spain, Murcia	233	155	0	0	0	40	195	1	33	0	34	3	1	0	4	0	0
Spain, Navarre *	84	57	0	0			57	23	1	0	24	1	2	0	3	0	0
Spain, Valencian region	745	519	16	0			535	23	118	0	141	7	62	0	69	0	0
Sweden	1033	549	3	0			552			384	384	39	58	0	97	0	0
Switzerland †	882	703	9	7	12	0	732	49	52	2	103	41	6	0	47	1	0
the Netherlands &	1829	1095	8	0		79	1182	196	159	0	355	255	37	0	292	0	0
United Kingdom, England † ¶ #	6227	3575	40			892	4511	675	554	21	1251	206	248	10	464		0
United Kingdom, Northern Ireland † #	194	111	0	0	0		112	25		0	30	36	16	0	52	0	0
United Kingdom, Scotland †	604	442	1	0			443	58	41	2	101	30	24	6	60	0	0
United Kingdom, Wales † #	407	284		0	0	25	311	32	43	0	75	11	11	0	21	0	0

Abbreviations used: HD: haemodialysis; Unkn: unknown; HF: haemofiltration; HDF: haemodiafiltration; APD: automated peritoneal dialysis; CAPD: continuous ambulatory peritoneal dialysis; PD: peritoneal dialysis; Tx: transplant
Categories may not add up because of rounding; When cells are left empty, (complete) data are unavailable
§ The incidence is underestimated by approximately 2% due to an estimated 2% underestimation of the incidence of haemodialysis
** Patients younger than 20 years of age are not reported*
† The incident counts at day 91 are estimated (see methods)
‖ The incidence of preemptive transplantation is underestimated by approximately 30%
& The incidence is underestimated by approximately 8%
¶ The incidence is underestimated by approximately 7.5% due to one centre not providing data and patients opting out of data sharing for research purposes
# Values based on 1 to 5 patients are suppressed

Table B.3.8

Incidence per million population by treatment modality, unadjusted

at day 91

	All	Haemodialysis						Peritoneal dialysis				Kidney transplant				Unkn	Missing
		HD hospital/ centre	HD home	HD type Unkn	HF	HDF	Total HD/ HF/HDF	APD	CAPD	PD type Unkn	Total PD	Living donor	Deceased donor	Tx type Unkn	Total Tx		
	Pmp	Pmp	Pmp	Pmp	Pmp	Pmp	Pmp	Pmp	Pmp	Pmp	Pmp	Pmp	Pmp	Pmp	Pmp	Pmp	Pmp
Austria	110.3	84.7	0	0	0.1	13.0	97.7	3.1	5.9	0.1	9.1	2.0	1.5	0	3.5	0	0
Belgium, Dutch-speaking *	157.8	91.7	1.5	0	0	45.2	138.4	8.7	7.7	0	16.4	1.5	1.5	0	3.0	0	0
Belgium, French-speaking *	171.4	81.9	2.2	0	2.4	62.5	149.0	9.9	8.5	0	18.5	0.8	3.0	0	3.9	0	0
Bosnia and Herzegovina	106.8	85.8	0	0	0	16.4	102.2	0	4.2	0	4.2	0.3	0	0	0.3	0	0
Denmark	114.0	66.4	0	0			66.4	15.6	19.5	1.7	36.8	5.1	5.6	0	10.7	0.2	0
Estonia †	82.0	69.9	0	0	0	0	69.9	9.4	0	0	9.4	0.7	2.0	0	2.7	0	0
France (17 of 18 regions) †	151.1	93.0	0.3	0.1	0.2	34.8	128.5	4.3	11.0	0.2	15.5	3.2	3.8	0	7.0	0.1	0
Greece	252.7	224.8	0.1	0	0	14.0	238.9	4.8	7.1	0	11.9	1.8	0.1	0	1.9	0	0
Iceland	78.5	55.0	0	0			55.0	0	13.1	0	13.1	7.9	2.6	0	10.5	0	0
Montenegro * †	55.6	55.6	0	0	0	0	55.6	0	0	0	0	0	0	0	0	0	0
Norway	96.6	61.8	0	0			61.8			26.2	26.2	3.1	5.5	0	8.6	0	0
Romania ‖	158.2	153.4	0	0	0	1.8	155.2	0.1	1.8	0	1.9	0.9	0.2	0	1.1	0	0
Serbia	71.9	61.9	0	0.6	0	1.3	63.8	0.6	7.0	0	7.7	0.5	0	0	0.5	0	0
Spain, Andalusia	145.3	113.3	1.4	0			114.7			22.5	22.5	2.8	5.3	0	8.1	0	0
Spain, Aragon	138.5	87.1	0	0		20.1	107.2	7.4	14.1	0	21.6	3.0	6.7	0	9.7	0	0
Spain, Asturias	182.9	140.2	0	0		1.0	141.2	4.0	31.8	0	35.8	1.0	5.0	0	6.0	0	0
Spain, Basque country	124.7	83.6	3.2	0			86.7	0.5	29.8	0	30.3	4.5	3.2	0	7.7	0	0
Spain, Canary Islands	178.3	124.2	0.9	0	0	0	125.1	0	0	50.5	50.5	0.9	1.8	0	2.7	0	0
Spain, Cantabria *	144.8	105.6	1.7	0			107.3	5.1	8.5	0	13.6	1.7	22.2	0	23.9	0	0
Spain, Castile and León * †	132.3	102.8	1.3	0			104.1	7.7	15.5	0	23.2	2.2	2.8	0	5.1	0	0
Spain, Castile-La Mancha *	110.7	89.4	0.5	0			89.9	2.4	17.9	0	20.3	0	0.5	0	0.5	0	0
Spain, Catalonia †	174.4	53.6	1.1	0		76.1	130.9	4.5	17.3	0	21.8	10.9	10.9	0	21.7	0	0
Spain, Community of Madrid	107.8	74.9	0.6	0		8.9	84.4	5.1	11.4	0	16.5	3.0	3.9	0	6.9	0	0
Spain, Extremadura	143.2	125.1	3.8	0			128.9	5.7	5.7	0	11.4	1.9	0.9	0	2.8	0	0
Spain, Galicia	152.8	116.1	1.5	0	0	0	117.6	1.9	26.3	0	28.2	5.6	1.5	0	7.0	0	0
Spain, La Rioja	124.6	90.3	3.1	0	0	0	93.5	9.3	15.6	0	24.9	6.2	0	0	6.2	0	0
Spain, Murcia	152.1	101.2	0	0	0	26.1	127.3	0.7	21.5	0	22.2	2.0	0.7	0	2.6	0	0
Spain, Navarre *	125.7	85.3	0	0			85.3	34.4	1.5	0	35.9	1.5	3.0	0	4.5	0	0
Spain, Valencian region	146.1	101.8	3.1	0			104.9	4.5	23.1	0	27.7	1.4	12.2	0	13.5	0	0
Sweden	98.5	52.4	0.3	0			52.6			36.6	36.6	3.7	5.5	0	9.2	0	0
Switzerland †	101.5	80.9	1.1	0.8	1.4	0	84.2	5.6	5.9	0.2	11.8	4.7	0.7	0	5.4	0.1	0
the Netherlands	112.3	67.2	0.5	0		4.9	72.6	12.0	9.8	0	21.8	15.7	2.3	0	17.9	0	0
United Kingdom, England † #	117.9	67.7	0.8			16.9	85.4	12.8	10.5	0.4	23.7	3.9	4.7	0.2	8.8		0
United Kingdom, Northern Ireland † #	101.4	58.3	0	0	0		58.6	13.3		0	15.5	18.8	8.4	0	27.2	0	0
United Kingdom, Scotland †	110.9	81.1	0.2	0			81.3	10.6	7.5	0.4	18.5	5.5	4.4	1.1	11.0	0	0
United Kingdom, Wales † #	130.0	90.7		0	0	7.9	99.2	10.3	13.7	0	23.9	3.4	3.4	0	6.8	0	0

Abbreviations used: HD: haemodialysis; Unkn: unknown; HF: haemofiltration; HDF: haemodiafiltration; APD: automated peritoneal dialysis; CAPD: continuous ambulatory peritoneal dialysis; PD: peritoneal dialysis; Tx: transplant
Categories may not add up because of rounding; When cells are left empty, (complete) data are unavailable
** Patients younger than 20 years of age are not reported*
† The incident counts at day 91, on which the data presented in this table are based, are estimated (see methods)
‖ The incidence of preemptive transplantation is underestimated by approximately 30%
# Values based on 1 to 5 patients are suppressed

Table B.3.9

Incidence per million population by treatment modality, adjusted

at day 91, adjusted for age and sex

	All	Haemodialysis						Peritoneal dialysis				Kidney transplant				Unkn	Missing
		HD hospital/ centre	HD home	HD type Unkn	HF	HDF	Total HD/ HF/HDF	APD	CAPD	PD type Unkn	Total PD	Living donor	Deceased donor	Tx type Unkn	Total Tx		
	Pmp	Pmp	Pmp	Pmp	Pmp	Pmp	Pmp	Pmp	Pmp	Pmp	Pmp	Pmp	Pmp	Pmp	Pmp	Pmp	Pmp
Austria	112.5	86.6	0	0	0.1	13.2	99.9	3.1	5.9	0.1	9.2	2.0	1.5	0	3.4	0	0
Belgium, Dutch-speaking *	157.2	91.3	1.5	0	0	44.9	137.7	8.7	7.7	0	16.4	1.5	1.5	0	3.0	0	0
Belgium, French-speaking *	198.9	95.5	2.5	0	2.8	72.7	173.5	11.5	9.9	0	21.4	0.9	3.1	0	4.0	0	0
Bosnia and Herzegovina	134.8	107.3	0	0	0	22.8	130.1	0	4.5	0	4.5	0.3	0	0	0.3	0	0
Denmark	116.1	68.0	0	0			68.0	15.3	19.7	1.7	36.7	5.3	5.8	0	11.1	0.2	0
Estonia †	88.9	75.8	0	0	0	0	75.8	10.3	0	0	10.3	0.9	2.2	0	3.0	0	0
France (17 of 18 regions) †	156.0	95.8	0.3	0.2	0.3	36.0	132.5	4.5	11.3	0.2	16.0	3.3	4.1	0	7.4	0.1	0
Greece	231.9	205.2	0.1	0	0	13.4	218.6	4.7	6.6	0	11.3	1.8	0.1	0	2.0	0	0
Iceland	89.8	68.1	0	0			68.1	0	12.4	0	12.4	6.2	3.0	0	9.2	0	0
Montenegro * †	66.4	66.4	0	0	0	0	66.4	0	0	0	0	0	0	0	0	0	0
Norway	103.9	66.6	0	0			66.6			28.3	28.3	3.1	5.8	0	9.0	0	0
Romania ||	166.3	161.3	0	0	0	1.9	163.2	0	1.9	0	2.0	0.9	0.1	0	1.1	0	0
Serbia	69.5	59.6	0	0.6	0	1.3	61.5	0.7	6.8	0	7.4	0.5	0	0	0.5	0	0
Spain, Andalusia	156.6	122.3	1.5	0			123.7			24.2	24.2	3.0	5.7	0	8.6	0	0
Spain, Aragon	134.4	84.5	0	0		19.6	104.0	7.3	13.6	0	20.9	2.9	6.6	0	9.5	0	0
Spain, Asturias	151.4	115.5	0	0		0.7	116.2	3.4	25.8	0	29.2	0.8	5.1	0	5.9	0	0
Spain, Basque country	117.1	78.7	3.0	0			81.7	0.4	28.1	0	28.5	4.2	2.7	0	6.9	0	0
Spain, Canary Islands	190.1	134.0	1.1	0	0	0	135.2	0	0	52.3	52.3	0.8	1.9	0	2.7	0	0
Spain, Cantabria *	136.3	99.6	1.8	0			101.5	4.5	8.2	0	12.6	2.1	20.2	0	22.2	0	0
Spain, Castile and León * †	111.3	85.5	1.3	0			86.8	6.6	13.4	0	19.9	2.0	2.6	0	4.7	0	0
Spain, Castile-La Mancha *	113.4	91.9	0.4	0			92.4	2.4	18.2	0	20.6	0	0.5	0	0.5	0	0
Spain, Catalonia †	184.8	57.4	1.2	0		81.5	140.0	4.7	18.1	0	22.7	10.9	11.3	0	22.2	0	0
Spain, Community of Madrid	117.6	82.8	0.7	0		9.6	93.1	5.3	12.3	0	17.5	2.9	4.2	0	7.0	0	0
Spain, Extremadura	139.2	121.7	3.7	0			125.4	5.7	5.6	0	11.2	1.7	0.9	0	2.6	0	0
Spain, Galicia	134.1	101.2	1.2	0	0	0	102.4	1.8	23.4	0	25.2	5.1	1.3	0	6.5	0	0
Spain, La Rioja	120.8	88.3	2.9	0	0	0	91.2	8.5	15.2	0	23.8	5.8	0	0	5.8	0	0
Spain, Murcia	178.7	120.8	0	0	0	30.3	151.1	0.7	24.4	0	25.0	2.0	0.7	0	2.6	0	0
Spain, Navarre *	126.4	86.3	0	0			86.3	33.8	1.8	0	35.6	1.3	3.1	0	4.5	0	0
Spain, Valencian region	148.2	103.8	3.1	0			106.9	4.3	23.7	0	28.0	1.4	12.0	0	13.4	0	0
Sweden	101.4	54.2	0.3	0			54.5			37.1	37.1	3.8	6.0	0	9.8	0	0
Switzerland †	105.1	84.3	1.2	1.0	1.4	0	87.7	5.6	6.1	0.2	12.0	4.6	0.7	0	5.3	0.1	0
the Netherlands	113.3	68.0	0.6	0		4.9	73.5	11.9	9.8	0	21.7	15.8	2.3	0	18.1	0	0
United Kingdom, England † #	126.2	72.9	0.8			18.1	92.0	13.5	11.2	0.4	25.1	4.0	5.0	0.2	9.2		0
United Kingdom, Northern Ireland † #	111.1	65.2	0	0	0		65.6	14.4		0	17.0	19.8	9.0	0	28.9	0	0
United Kingdom, Scotland †	109.0	79.6	0.2	0			79.9	11.0	7.2	0.4	18.2	5.5	4.6	0.9	11.0	0	0
United Kingdom, Wales † #	130.7	90.6		0	0	7.8	98.8	10.7	14.6	0	24.9	3.6	3.6	0	7.2	0	0

Abbreviations used: HD: haemodialysis; Unkn: unknown; HF: haemofiltration; HDF: haemodiafiltration; APD: automated peritoneal dialysis; CAPD: continuous ambulatory peritoneal dialysis; PD: peritoneal dialysis; Tx: transplant
Categories may not add up because of rounding; When cells are left empty, (complete) data are unavailable
** Patients younger than 20 years of age are not reported*
† The incident counts at day 91, on which the data presented in this table are based, are estimated (see methods)
|| The incidence of preemptive transplantation is underestimated by approximately 30%
# Values based on 1 to 5 patients are suppressed

Table B.3.10
Treatment modality distribution, unadjusted
at day 91

	All	Haemodialysis						Peritoneal dialysis				Kidney transplant				Unkn	Missing
		HD hospital/ centre	HD home	HD type Unkn	HF	HDF	Total HD/ HF/HDF	APD	CAPD	PD type Unkn	Total PD	Living donor	Deceased donor	Tx type Unkn	Total Tx		
	%	%	%	%	%	%	%	%	%	%	%	%	%	%	%	%	%
Austria	100	76.7	0	0	0.1	11.7	88.6	2.8	5.4	0.1	8.2	1.9	1.3	0	3.2	0	0
Belgium, Dutch-speaking *	100	58.1	0.9	0	0	28.6	87.7	5.5	4.9	0	10.4	0.9	0.9	0	1.9	0	0
Belgium, French-speaking *	100	47.8	1.3	0	1.4	36.4	87.0	5.8	5.0	0	10.8	0.5	1.8	0	2.2	0	0
Bosnia and Herzegovina	100	80.4	0	0	0	15.4	95.8	0	4.0	0	4.0	0.3	0	0	0.3	0	0
Denmark	100	58.2	0	0			58.2	13.7	17.1	1.5	32.2	4.5	4.9	0	9.4	0.1	0
Estonia †	100	85.2	0	0	0	0	85.2	11.4	0	0	11.4	0.9	2.4	0	3.3	0	0
France (17 of 18 regions) †	100	61.6	0.2	0.1	0.2	23.0	85.1	2.8	7.3	0.1	10.2	2.1	2.5	0	4.6	0.1	0
Greece	100	89.0	0	0	0	5.5	94.5	1.9	2.8	0	4.7	0.7	0	0	0.8	0	0
Iceland	100	70.0	0	0			70.0	0	16.7	0	16.7	10.0	3.3	0	13.3	0	0
Montenegro * †	100	100	0	0	0	0	100	0	0	0	0	0	0	0	0	0	0
Norway	100	63.9	0	0			63.9			27.1	27.1	3.2	5.7	0	8.9	0	0
Romania ||	100	96.9	0	0	0	1.2	98.1	0	1.2	0	1.2	0.6	0.1	0	0.7	0	0
Serbia	100	86.1	0	0.9	0	1.7	88.7	0.9	9.8	0	10.7	0.7	0	0	0.7	0	0
Spain, Andalusia	100	78.0	1.0	0			79.0			15.5	15.5	1.9	3.6	0	5.6	0	0
Spain, Aragon	100	62.9	0	0		14.5	77.4	5.4	10.2	0	15.6	2.2	4.8	0	7.0	0	0
Spain, Asturias	100	76.6	0	0		0.5	77.2	2.2	17.4	0	19.6	0.5	2.7	0	3.3	0	0
Spain, Basque country	100	67.0	2.5	0			69.6	0.4	23.9	0	24.3	3.6	2.5	0	6.2	0	0
Spain, Canary Islands	100	69.6	0.5	0	0	0	70.2	0	0	28.3	28.3	0.5	1.0	0	1.5	0	0
Spain, Cantabria *	100	72.9	1.2	0			74.1	3.5	5.9	0	9.4	1.2	15.3	0	16.5	0	0
Spain, Castile and León * †	100	77.7	1.0	0			78.7	5.8	11.7	0	17.5	1.7	2.1	0	3.8	0	0
Spain, Castile-La Mancha *	100	80.8	0.4	0			81.2	2.2	16.2	0	18.3	0	0.4	0	0.4	0	0
Spain, Catalonia †	100	30.8	0.6	0		43.6	75.0	2.6	9.9	0	12.5	6.2	6.2	0	12.5	0	0
Spain, Community of Madrid	100	69.5	0.6	0		8.2	78.3	4.8	10.6	0	15.3	2.7	3.6	0	6.4	0	0
Spain, Extremadura	100	87.4	2.6	0			90.1	4.0	4.0	0	7.9	1.3	0.7	0	2.0	0	0
Spain, Galicia	100	76.0	1.0	0	0	0	76.9	1.2	17.2	0	18.4	3.6	1.0	0	4.6	0	0
Spain, La Rioja	100	72.5	2.5	0	0	0	75.0	7.5	12.5	0	20.0	5.0	0	0	5.0	0	0
Spain, Murcia	100	66.5	0	0	0	17.2	83.7	0.4	14.2	0	14.6	1.3	0.4	0	1.7	0	0
Spain, Navarre *	100	67.9	0	0			67.9	27.4	1.2	0	28.6	1.2	2.4	0	3.6	0	0
Spain, Valencian region	100	69.7	2.1	0			71.8	3.1	15.8	0	18.9	0.9	8.3	0	9.3	0	0
Sweden	100	53.1	0.3	0			53.4			37.2	37.2	3.8	5.6	0	9.4	0	0
Switzerland †	100	79.7	1.1	0.8	1.4	0	82.9	5.6	5.9	0.2	11.6	4.6	0.7	0	5.3	0.1	0
the Netherlands	100	59.9	0.4	0		4.3	64.6	10.7	8.7	0	19.4	13.9	2.0	0	16.0	0	0
United Kingdom, England † #	100	57.4	0.6			14.3	72.4	10.8	8.9	0.3	20.1	3.3	4.0	0.2	7.4		0
United Kingdom, Northern Ireland † #	100	57.5	0	0	0		57.8	13.1		0	15.3	18.6	8.3	0	26.9	0	0
United Kingdom, Scotland †	100	73.1	0.2	0			73.3	9.6	6.7	0.4	16.7	5.0	4.0	1.0	9.9	0	0
United Kingdom, Wales † #	100	69.8		0	0	6.1	76.3	7.9	10.6	0	18.4	2.6	2.6	0	5.2	0	0

Abbreviations used: HD: haemodialysis; Unkn: unknown; HF: haemofiltration; HDF: haemodiafiltration; APD: automated peritoneal dialysis; CAPD: continuous ambulatory peritoneal dialysis; PD: peritoneal dialysis; Tx: transplant
Categories may not add up because of rounding; When cells are left empty, (complete) data are unavailable
** Patients younger than 20 years of age are not reported*
† The incident counts at day 91, on which the data presented in this table are based, are estimated (see methods)
|| The incidence of preemptive transplantation is underestimated by approximately 30%
# Values based on 1 to 5 patients are suppressed

Table B.3.11
Treatment modality distribution by age, sex, and primary renal disease, unadjusted
at day 91

	All				0-19				20-44				45-64				65-74				75+				Male				Female				DM				Non-DM			
	HD	PD	Tx	Un	HD	PD	Tx	Un	HD	PD	Tx	Un	HD	PD	Tx	Un	HD	PD	Tx	Un	HD	PD	Tx	Un	HD	PD	Tx	Un	HD	PD	Tx	Un	HD	PD	Tx	Un	HD	PD	Tx	Un
	%	%	%	%	%	%	%	%	%	%	%	%	%	%	%	%	%	%	%	%	%	%	%	%	%	%	%	%	%	%	%	%	%	%	%	%	%	%	%	%
Austria	89	8	3	0	67	0	33	0	80	9	10	0	83	12	5	0	93	6	1	0	94	6	0	0	88	9	3	0	90	7	3	0	93	5	2	0	87	9	4	0
Belgium, Dutch-speaking *	88	10	2	0					69	23	8	0	81	15	4	0	89	10	1	0	93	7	0	0	86	12	2	0	90	7	2	0	90	10	1	0	87	11	2	0
Belgium, French-speaking *	87	11	2	0					74	14	12	0	84	13	3	0	91	8	1	0	88	11	1	0	88	11	2	0	86	11	3	0	91	8	1	0	86	11	3	0
Bosnia and Herzegovina	96	4	0	0	100	0	0	0	97	0	3	0	91	9	0	0	98	2	0	0	98	2	0	0	95	5	0	0	96	3	1	0	95	5	0	0	96	3	0	0
Denmark	58	32	9	0	22	56	22	0	51	32	17	0	49	35	16	0	68	27	5	0	67	32	1	0	57	35	9	0	61	28	11	0	68	27	5	0	55	34	11	0
Estonia †	85	11	3	0	100	0	0	0	77	15	8	0	78	16	6	0	94	6	0	0	89	11	0	0	84	12	4	0	86	11	3	0	100	0	0	0	82	14	4	0
France (17 of 18 regions) †	85	10	5	0	54	20	25	1	70	13	16	0	82	11	7	0	89	8	2	0	89	10	1	0	86	10	4	0	84	11	5	0	92	7	1	0	83	11	6	0
Greece	95	5	1	0	67	25	8	0	80	9	12	0	93	6	0	0	95	5	0	0	97	3	0	0	94	5	1	0	95	4	1	0	96	4	0	0	94	5	1	0
Iceland	70	17	13	0	33	0	67	0	43	43	14	0	80	10	10	0	75	25	0	0	100	0	0	0	65	22	13	0	86	0	14	0	86	14	0	0	65	17	17	0
Montenegro * †	100	0	0	0					100	0	0	0	100	0	0	0	100	0	0	0	100	0	0	0	100	0	0	0	100	0	0	0	100	0	0	0	100	0	0	0
Norway	64	27	9	0	40	20	40	0	53	21	26	0	65	23	12	0	67	28	5	0	65	34	1	0	65	28	7	0	63	24	13	0	67	29	3	0	63	27	10	0
Romania ‖	98	1	1	0	78	14	8	0	94	2	4	0	98	1	1	0	99	1	0	0	99	1	0	0	98	1	1	0	98	2	1	0	98	2	0	0	98	1	1	0
Serbia	89	11	1	0	50	50	0	0	89	9	2	0	84	15	1	0	92	8	0	0	96	4	0	0	90	10	1	0	87	13	1	0	87	13	0	0	89	10	1	0
Spain, Andalusia	79	15	6	0	55	18	27	0	79	17	5	0	78	15	7	0	79	16	5	0	81	15	4	0	78	16	6	0	80	15	5	0	87	12	1	0	76	17	7	0
Spain, Aragon	77	16	7	0	20	20	60	0	65	5	30	0	73	24	4	0	79	19	2	0	91	8	2	0	76	17	7	0	82	12	6	0	95	5	0	0	73	18	9	0
Spain, Asturias	77	20	3	0	0	0	0	0	63	13	25	0	74	20	6	0	77	23	0	0	82	17	2	0	78	20	2	0	76	18	6	0	69	29	2	0	80	17	4	0
Spain, Basque country	70	24	6	0	100	0	0	0	79	18	3	0	61	28	11	0	71	26	3	0	75	21	4	0	70	24	6	0	69	24	6	0	82	18	0	0	66	26	8	0
Spain, Canary Islands	70	28	2	0	100	0	0	0	64	33	2	0	65	32	2	0	68	31	2	0	82	18	0	0	72	27	1	0	66	31	2	0	82	17	1	0	65	33	2	0
Spain, Cantabria *	74	9	16	0					70	0	30	0	75	17	8	0	60	12	28	0	88	4	8	0	83	0	17	0	61	24	15	0	80	0	20	0	73	11	16	0
Spain, Castile and León * †	79	18	4	0					68	16	16	0	61	30	9	0	81	18	1	0	91	9	0	0	78	18	5	0	80	18	2	0	85	8	7	0	77	21	3	0
Spain, Castile-La Mancha *	81	18	0	0					62	38	0	0	73	26	1	0	78	22	0	0	97	3	0	0	80	19	1	0	83	17	0	0	89	11	0	0	79	21	1	0
Spain, Catalonia †	75	13	12	0	22	16	62	0	69	11	20	0	63	17	20	0	76	13	11	0	88	8	3	0	76	12	12	0	74	13	14	0	86	9	5	0	72	13	14	0
Spain, Community of Madrid	78	15	6	0	40	20	40	0	72	19	9	0	69	22	9	0	79	15	6	0	91	7	2	0	80	15	4	0	74	15	11	0	89	7	4	0	76	18	7	0
Spain, Extremadura	90	8	2	0	100	0	0	0	83	11	6	0	91	4	4	0	88	13	0	0	94	6	0	0	87	11	2	0	95	4	2	0	93	3	3	0	89	9	2	0
Spain, Galicia	77	18	5	0	50	50	0	0	76	16	8	0	69	22	9	0	79	19	3	0	85	14	1	0	78	18	4	0	75	19	7	0	85	15	0	0	74	20	6	0
Spain, La Rioja	75	20	5	0	0	0	0	0	67	33	0	0	54	31	15	0	70	30	0	0	100	0	0	0	65	27	8	0	93	7	0	0	60	40	0	0	77	17	6	0
Spain, Murcia	84	15	2	0	100	0	0	0	68	32	0	0	76	18	6	0	85	15	0	0	93	7	0	0	85	13	2	0	82	17	1	0	85	15	0	0	83	15	2	0
Spain, Navarre *	68	29	4	0					62	23	15	0	70	27	3	0	68	32	0	0	68	32	0	0	71	25	4	0	62	34	3	0	83	17	0	0	62	33	5	0
Spain, Valencian region	72	19	9	0	29	29	43	0	62	20	18	0	65	21	15	0	68	24	8	0	87	11	2	0	73	19	8	0	69	19	12	0	82	13	5	0	70	20	10	0
Sweden	53	37	9	0	14	43	43	0	46	40	14	0	49	37	14	0	58	33	9	0	59	40	1	0	51	41	8	0	58	30	12	0	57	37	6	0	52	37	10	0
Switzerland †	83	12	5	0	20	44	36	0	65	20	15	0	75	14	10	0	88	11	1	0	93	6	0	0	82	12	6	0	85	10	5	0	90	8	2	0	81	13	6	0
the Netherlands	65	19	16	0	32	23	45	0	52	17	31	0	59	18	23	0	70	19	10	0	74	23	3	0	64	20	16	0	65	18	17	0	72	22	6	0	63	19	19	0
United Kingdom, England † #	72	20	7		38	40	21	0	58	26	16	0	74	19	8		75	19	6	0	82	17	1	0	74	20	7	0	70	21	9		79	18	3	0	70	21	9	
United Kingdom, Northern Ireland † #	58	15	27	0				0	25	23	52	0	58	16	26	0	66		30	0	76	17		0	55	15	29	0	63	15	22	0	91			0	51	17	32	0
United Kingdom, Scotland †	73	17	10	0	13	63	25	0	65	21	13	0	67	18	15	0	83	13	4	0	86	10	3	0	75	16	9	0	71	17	12	0	78	12	10	0	72	18	10	0
United Kingdom, Wales † #	76	18	5	0				0	57	28	14	0	74	20	5	0	84	14		0	89	11	0	0	76	18	6	0	76	19	4	0	84	15		0	73	20	7	0

Abbreviations used: HD: haemodialysis; PD: peritoneal dialysis; Tx: transplant; Un: unknown; DM: diabetes mellitus
Categories may not add up because of missing values or rounding
** Patients younger than 20 years of age are not reported*
† The incident counts at day 91, on which the data presented in this table are based, are estimated (see methods)
‖ The incidence of preemptive transplantation is underestimated by approximately 30%
# Values based on 1 to 5 patients are suppressed

Table B.4.1
Prevalent counts and percentages by age and sex
prevalent patients on December 31

	All			0-19						20-44						45-64						65-74						75+					
	All	Male	Female	All		Male		Female		All		Male		Female		All		Male		Female		All		Male		Female		All		Male		Female	
	N (100%)	N (100%)	N (100%)	N	%	N	%	N	%	N	%	N	%	N	%	N	%	N	%	N	%	N	%	N	%	N	%	N	%	N	%	N	%
Austria §	8950	5745	3205	79	1	53	1	26	1	1089	12	665	12	424	13	3562	40	2305	40	1257	39	2298	26	1493	26	805	25	1922	21	1229	21	693	22
Belgium, Dutch-speaking *	8702	5347	3355							879	10	520	10	359	11	2740	31	1726	32	1014	30	2216	25	1383	26	833	25	2867	33	1718	32	1149	34
Belgium, French-speaking *	7083	4348	2735							684	10	393	9	291	11	2307	33	1464	34	843	31	1975	28	1221	28	754	28	2117	30	1270	29	847	31
Bosnia and Herzegovina	2441	1497	944	16	1	10	1	6	1	376	15	241	16	135	14	1029	42	665	44	364	39	669	27	400	27	269	28	351	14	181	12	170	18
Denmark	5848	3645	2203	70	1	44	1	26	1	962	16	612	17	350	16	2460	42	1531	42	929	42	1294	22	801	22	493	22	1062	18	657	18	405	18
Estonia	1136	660	476	12	1	6	1	6	1	169	15	98	15	71	15	493	43	299	45	194	41	265	23	144	22	121	25	197	17	113	17	84	18
France (17 of 18 regions)	93486	58119	35367	969	1	596	1	373	1	12026	13	7308	13	4718	13	32215	34	19972	34	12243	35	24285	26	15263	26	9022	26	23991	26	14980	26	9011	25
Greece	15440	10270	5170	82	1	55	1	27	1	1368	9	899	9	469	9	5053	33	3399	33	1654	32	3913	25	2657	26	1256	24	5024	33	3260	32	1764	34
Iceland	312	202	110	8	3	6	3	2	2	64	21	38	19	26	24	125	40	77	38	48	44	65	21	46	23	19	17	50	16	35	17	15	14
Montenegro *	305	188	117							47	15	26	14	21	18	122	40	72	38	50	43	96	31	65	35	31	26	40	13	25	13	15	13
Norway	5442	3518	1924	86	2	53	2	33	2	810	15	507	14	303	16	2155	40	1354	38	801	42	1342	25	895	25	447	23	1049	19	709	20	340	18
Romania	24054	13878	10176	138	1	75	1	63	1	2684	11	1606	12	1078	11	8374	35	5119	37	3255	32	6773	28	3821	28	2952	29	6085	25	3257	23	2828	28
Serbia	6000	3732	2252	63	1	42	1	21	1	732	12	465	12	266	12	2296	38	1406	38	884	39	1794	30	1135	30	653	29	1112	19	681	18	428	19
Spain, Andalusia	11412	7039	4373	119	1	79	1	40	1	1487	13	915	13	572	13	4587	40	2895	41	1692	39	2741	24	1726	25	1015	23	2478	22	1424	20	1054	24
Spain, Aragon	2014	1346	668	16	1	8	1	8	1	169	8	115	9	54	8	682	34	426	32	256	38	522	26	373	28	149	22	625	31	424	32	201	30
Spain, Asturias	1492	971	521	10	1	8	1	2	0	112	8	77	8	35	7	524	35	330	34	194	37	486	33	317	33	169	32	360	24	239	25	121	23
Spain, Basque country	2887	1857	1030	33	1	18	1	15	1	362	13	223	12	139	13	1078	37	683	37	395	38	773	27	508	27	265	26	641	22	425	23	216	21
Spain, Canary Islands	3497	2301	1196	9	0	6	0	3	0	374	11	232	10	142	12	1433	41	945	41	488	41	911	26	615	27	296	25	770	22	503	22	267	22
Spain, Cantabria *	707	481	226							72	10	45	9	27	12	279	39	184	38	95	42	185	26	130	27	55	24	171	24	122	25	49	22
Spain, Castile and León *	3165	2090	1075							274	9	180	9	94	9	1114	35	720	34	394	37	810	26	547	26	263	24	967	31	643	31	324	30
Spain, Castile-La Mancha *	2551	1582	969							236	9	142	9	94	10	1039	41	649	41	390	40	640	25	422	27	218	22	636	25	369	23	267	28
Spain, Catalonia	12027	7659	4368	147	1	86	1	61	1	1260	10	793	10	467	11	4568	38	2915	38	1653	38	3009	25	1970	26	1039	24	3043	25	1895	25	1148	26
Spain, Community of Madrid	8194	5194	3000	88	1	64	1	24	1	1003	12	575	11	428	14	3166	39	2002	39	1164	39	1962	24	1277	25	685	23	1975	24	1276	25	699	23
Spain, Extremadura	1512	963	549	7	0	4	0	3	1	134	9	77	8	57	10	599	40	375	39	224	41	405	27	276	29	129	23	366	24	231	24	135	25
Spain, Galicia	4017	2580	1437	21	1	15	1	6	0	366	9	222	9	144	10	1566	39	1012	39	554	39	1065	27	691	27	374	26	999	25	640	25	359	25
Spain, La Rioja	397	258	139	1	0	0	0	1	1	46	12	33	13	13	9	166	42	105	41	61	44	90	23	60	23	30	22	94	24	60	23	34	24
Spain, Murcia	2230	1450	780	26	1	17	1	9	1	237	11	145	10	92	12	883	40	574	40	309	40	546	24	361	25	185	24	538	24	353	24	185	24
Spain, Navarre *	933	604	329							113	12	60	10	53	16	340	36	227	38	113	34	255	27	175	29	80	24	225	24	142	24	83	25
Spain, Valencian region	7582	4794	2788	69	1	49	1	20	1	796	10	517	11	279	10	2729	36	1716	36	1013	36	1945	26	1237	26	708	25	2043	27	1275	27	768	28
Sweden	10573	6785	3788	173	2	101	1	72	2	1673	16	1063	16	610	16	4016	38	2575	38	1441	38	2495	24	1591	23	904	24	2216	21	1455	21	761	20
Switzerland	8885	5617	3268	105	1	68	1	37	1	1095	12	688	12	407	12	3179	36	2010	36	1169	36	2262	25	1408	25	854	26	2244	25	1443	26	801	25
the Netherlands &	18096	10996	7100	207	1	121	1	86	1	2562	14	1561	14	1001	14	7075	39	4314	39	2761	39	4686	26	2788	25	1898	27	3566	20	2212	20	1354	19
United Kingdom, England ¶	55950	34431	21519	1149	2	698	2	451	2	10306	18	6239	18	4067	19	23926	43	14700	43	9226	43	11746	21	7254	21	4492	21	8823	16	5540	16	3283	15
United Kingdom, Northern Ireland	2099	1280	819	40	2	28	2	12	1	384	18	232	18	152	19	893	43	545	43	348	42	386	18	236	18	150	18	396	19	239	19	157	19
United Kingdom, Scotland	5671	3390	2227	87	2	61	2	26	1	1043	18	640	19	403	18	2538	45	1493	44	1045	47	1244	22	767	23	477	21	705	12	429	13	276	12
United Kingdom, Wales	3360	2112	1248	56	2	36	2	20	2	659	20	405	19	254	20	1353	40	840	40	513	41	720	21	463	22	257	21	572	17	368	17	204	16

Categories may not add up because of missing values or rounding
§ The prevalence is underestimated by approximately 2% due to one haemodialysis centre not submitting data
** Patients younger than 20 years of age are not reported*
& The prevalence is underestimated by approximately 4%
¶ The prevalence is underestimated by approximately 8.4% due to one centre not providing data and patients opting out of data sharing for research purposes

Table B.4.2
Prevalence per million (age-related) population by age and sex, unadjusted
prevalent patients on December 31

	All			0-19			20-44			45-64			65-74			75+		
	All	Male	Female	All	Male	Female	All	Male	Female	All	Male	Female	All	Male	Female	All	Male	Female
	Pmp	Pmp	Pmp	Pmarp	Pmarp	Pmarp	Pmarp	Pmarp	Pmarp	Pmarp	Pmarp	Pmarp	Pmarp	Pmarp	Pmarp	Pmarp	Pmarp	Pmarp
Austria	1017.1	1324.7	718.2	46.6	60.7	31.6	381.7	456.7	303.6	1402.5	1827.3	983.4	2617.0	3639.1	1720.7	2308.2	3651.4	1396.9
Belgium, Dutch-speaking *	1289.4	1599.0	985.3				424.8	498.8	349.6	1515.1	1892.7	1131.0	3029.7	3875.2	2224.0	4183.6	5980.3	2886.8
Belgium, French-speaking *	1436.3	1795.9	1089.5				416.6	476.1	356.4	1813.9	2305.2	1323.9	4106.9	5459.8	2930.9	5392.8	8413.4	3505.6
Bosnia and Herzegovina	691.3	864.2	524.8	20.3	24.8	15.7	307.3	387.2	224.6	1009.5	1338.6	696.6	2275.3	3102.7	1629.3	1687.7	2256.2	1330.7
Denmark	990.7	1241.5	742.5	54.2	66.4	41.3	518.1	648.9	383.2	1587.5	1977.2	1198.3	2064.8	2642.7	1523.5	1838.7	2601.2	1246.1
Estonia	842.2	1030.6	671.9	41.3	40.3	42.4	391.5	437.1	342.3	1397.7	1747.2	1068.4	1784.0	2410.2	1362.7	1566.5	3088.9	942.0
France (17 of 18 regions)	1382.7	1775.3	1014.1	60.7	72.9	48.0	597.3	734.0	463.6	1864.6	2366.0	1385.7	3208.4	4343.5	2224.7	3589.8	5648.9	2235.3
Greece	1479.4	2012.7	969.2	42.2	55.0	28.7	446.5	576.7	311.6	1656.9	2283.3	1059.6	3318.9	4801.7	2007.5	4176.7	6502.1	2514.7
Iceland	816.7	1027.8	593.1	85.6	124.7	44.1	453.4	508.0	391.9	1388.3	1675.9	1088.7	1954.2	2769.7	1140.9	2077.4	3170.9	1151.2
Montenegro *	494.2	616.5	374.7				225.8	244.5	206.4	757.5	920.8	603.4	1468.0	2183.0	870.3	1157.9	1846.4	714.1
Norway	997.2	1278.6	711.1	69.5	83.4	54.8	446.9	545.4	343.2	1532.5	1888.4	1162.2	2473.6	3334.1	1630.8	2290.8	3528.0	1323.1
Romania	1262.8	1500.0	1038.7	33.4	35.3	31.3	461.1	536.3	381.5	1562.3	1937.1	1197.8	2984.8	3933.3	2274.8	4164.5	6295.2	2996.4
Serbia	940.0	1201.8	687.0	50.6	65.5	34.8	367.5	458.7	272.0	1304.2	1641.7	977.9	2047.7	2889.9	1350.9	2181.6	3382.0	1388.0
Spain, Andalusia	1336.0	1673.2	1008.7	68.6	88.4	47.5	553.1	671.4	431.6	1783.1	2273.9	1302.1	3350.8	4464.7	2352.7	3405.5	4903.1	2410.6
Spain, Aragon	1499.5	2028.5	983.0	64.7	62.7	66.7	429.1	571.0	280.7	1678.1	2074.2	1273.4	3674.8	5522.4	1999.9	4074.5	6874.4	2191.6
Spain, Asturias	1483.4	2023.5	990.6	69.2	107.5	28.5	436.8	599.3	273.6	1588.5	2048.2	1149.6	3527.2	4975.6	2281.5	2623.4	4581.7	1422.4
Spain, Basque country	1304.4	1726.5	905.3	83.4	88.3	78.1	590.7	718.9	459.3	1566.5	2003.1	1137.8	3032.9	4263.4	1952.6	2450.1	4189.8	1348.5
Spain, Canary Islands	1590.4	2118.6	1074.8	23.7	30.7	16.3	515.3	640.2	390.8	2010.6	2646.0	1372.3	4356.2	6137.1	2717.7	4485.3	7081.7	2652.9
Spain, Cantabria *	1204.6	1690.2	747.6				442.2	548.9	334.0	1494.5	1998.4	1004.1	2621.6	3899.9	1477.2	2594.5	4797.1	1210.6
Spain, Castile and León *	1334.0	1789.6	892.3				435.5	559.0	306.1	1490.9	1915.8	1060.9	2820.9	3849.2	1813.4	2886.9	4740.6	1625.5
Spain, Castile-La Mancha *	1233.0	1525.3	939.2				370.8	431.4	305.9	1662.2	2037.2	1272.4	3292.7	4444.2	2192.9	3099.5	4373.6	2209.8
Spain, Catalonia	1543.4	1997.8	1103.4	95.5	108.3	81.8	508.5	630.5	382.8	2009.2	2565.2	1453.6	3978.9	5622.2	2560.1	4081.6	6417.7	2549.6
Spain, Community of Madrid	1277.8	1692.6	897.1	70.0	99.2	39.2	482.0	561.6	404.9	1666.5	2193.2	1179.3	3310.7	4842.4	2082.6	3394.2	5719.0	1948.4
Spain, Extremadura	1433.5	1846.7	1029.4	37.0	41.2	32.6	428.0	482.4	371.4	1839.0	2285.2	1386.0	3704.2	5142.6	2317.4	3117.9	4926.7	1914.9
Spain, Galicia	1490.1	1988.4	1027.7	50.1	69.5	29.5	498.3	600.6	394.6	1874.2	2476.2	1297.9	3244.8	4483.4	2148.3	2640.3	4287.1	1567.1
Spain, La Rioja	1236.8	1628.4	855.1	16.4	0	33.8	496.4	707.2	282.6	1697.3	2130.6	1257.2	2645.4	3609.0	1724.5	2645.6	4149.4	1613.6
Spain, Murcia	1455.7	1889.5	1020.3	76.2	96.4	54.6	471.3	559.6	377.4	2002.7	2574.1	1418.0	4253.3	5942.6	2735.8	4541.3	7324.9	2632.4
Spain, Navarre *	1396.5	1826.7	975.0				570.4	597.9	542.1	1711.8	2258.5	1151.7	3778.5	5369.6	2292.5	3281.5	5110.9	2035.2
Spain, Valencian region	1487.3	1910.0	1077.3	70.5	97.1	42.2	512.6	657.4	364.1	1762.8	2222.8	1305.2	3676.1	4988.2	2518.6	4176.8	6410.4	2646.1
Sweden	1008.2	1285.2	727.4	71.2	80.7	61.0	497.7	612.6	375.1	1567.7	1987.0	1138.4	2328.0	3022.9	1657.5	2088.2	3089.3	1289.3
Switzerland	1022.5	1301.6	747.2	60.6	76.4	44.0	380.8	470.6	287.9	1312.2	1653.2	968.7	2692.6	3496.5	1952.4	2737.9	4184.4	1687.2
the Netherlands	1064.9	1301.9	830.7	57.6	65.7	49.0	476.3	574.3	376.1	1538.5	1879.9	1198.5	2492.6	3021.3	1982.9	2314.4	3301.4	1554.9
United Kingdom, England	1069.6	1343.2	806.7	95.0	112.6	76.5	601.9	742.4	466.4	1791.4	2238.5	1359.0	2338.5	3003.7	1722.4	1871.6	2703.2	1232.1
United Kingdom, Northern Ireland	1098.6	1361.8	843.8	83.2	113.5	51.2	640.7	784.2	500.8	1805.2	2248.0	1379.7	2153.2	2682.9	1642.9	2535.5	3568.6	1759.9
United Kingdom, Scotland	1041.0	1280.9	795.1	76.9	105.3	47.1	605.7	759.9	458.1	1695.5	2056.8	1355.3	2075.1	2669.4	1528.1	1415.9	2027.2	964.1
United Kingdom, Wales	1072.9	1376.0	781.6	81.0	101.3	59.5	696.5	866.0	530.8	1649.1	2100.0	1220.1	2038.2	2704.8	1411.5	1784.8	2614.4	1135.1

** Patients younger than 20 years of age are not reported*

Table B.4.3

Sex, mean age, and median age

prevalent patients on December 31

	All						Male						Female					
	%	Mean (years)	SD	Median (years)	P25	P75	%	Mean (years)	SD	Median (years)	P25	P75	%	Mean (years)	SD	Median (years)	P25	P75
Austria	100	62.4	15.2	63.9	53.5	73.7	64.2	62.5	15.1	64.0	54.0	73.7	35.8	62.2	15.3	63.6	52.7	73.7
Belgium, Dutch-speaking *	100	66.6	15.2	68.5	57.1	77.9	61.4	66.5	15.0	68.4	57.0	77.5	38.6	66.8	15.5	68.8	57.3	78.5
Belgium, French-speaking *	100	66.2	14.8	68.0	57.0	76.7	61.4	66.1	14.5	67.7	56.9	76.5	38.6	66.4	15.3	68.6	57.4	77.4
Bosnia and Herzegovina	100	60.3	14.5	62.0	50.8	70.6	61.3	59.4	14.4	61.2	50.0	69.5	38.7	61.6	14.5	63.4	52.5	72.3
Denmark	100	59.6	15.9	60.9	49.5	72.0	62.3	59.4	15.9	60.5	49.5	72.0	37.7	59.8	15.8	61.5	49.6	71.8
Estonia	100	60.3	15.5	61.6	50.8	72.2	58.1	60.2	15.3	61.3	51.3	71.5	41.9	60.4	15.9	62.1	50.0	72.6
France (17 of 18 regions)	100	63.5	16.1	65.7	53.4	75.3	62.2	63.6	16.0	65.9	53.5	75.3	37.8	63.4	16.3	65.5	53.2	75.2
Greece	100	66.4	15.2	68.4	56.6	77.6	66.5	66.2	15.0	68.2	56.7	77.3	33.5	66.7	15.5	68.6	56.5	78.5
Iceland	100	57.5	17.6	58.6	45.9	71.8	64.7	58.4	17.8	59.7	47.6	72.2	35.3	55.9	17.0	56.5	44.8	68.1
Montenegro *	100	60.7	13.9	63.5	51.5	70.5	61.6	61.4	14.0	64.5	53.0	70.5	38.4	59.5	13.8	61.5	49.5	69.5
Norway	100	60.4	15.9	62.3	50.6	72.5	64.6	60.9	15.9	62.9	51.2	72.9	35.4	59.5	16.0	61.2	49.3	71.5
Romania	100	64.4	15.6	66.4	54.2	75.1	57.7	63.7	15.4	65.4	53.5	74.4	42.3	65.4	15.8	67.5	55.0	76.3
Serbia	100	62.1	15.0	64.4	52.8	72.5	62.2	62.0	15.1	64.6	52.5	72.5	37.5	62.3	14.8	64.0	53.2	72.6
Spain, Andalusia	100	62.0	15.3	63.4	52.4	73.8	61.7	61.7	15.2	63.0	52.3	73.3	38.3	62.4	15.6	63.9	52.5	74.6
Spain, Aragon	100	66.0	15.1	67.8	56.8	77.7	66.8	66.4	14.9	68.5	57.5	77.8	33.2	65.3	15.7	66.3	55.4	77.7
Spain, Asturias	100	65.1	13.8	67.0	56.9	74.7	65.1	65.1	14.1	67.0	57.6	74.9	34.9	65.2	13.3	67.3	55.5	74.2
Spain, Basque country	100	62.5	15.4	64.6	52.5	74.0	64.3	62.9	15.3	65.0	53.1	74.1	35.7	61.8	15.5	63.8	51.5	73.6
Spain, Canary Islands	100	63.1	14.3	64.1	53.6	73.8	65.8	63.2	14.1	64.6	54.2	73.7	34.2	62.8	14.6	63.8	52.5	74.0
Spain, Cantabria *	100	64.0	13.8	65.1	54.8	74.8	68.0	64.5	13.8	66.0	55.8	75.1	32.0	63.0	13.7	64.2	53.8	74.5
Spain, Castile and León *	100	66.2	14.3	67.2	57.1	77.3	66.0	66.3	14.2	67.6	57.2	77.2	34.0	65.9	14.5	66.5	56.8	77.5
Spain, Castile-La Mancha *	100	64.3	14.0	65.0	55.0	75.0	62.0	64.3	13.6	65.0	55.2	74.5	38.0	64.4	14.7	65.0	54.8	76.0
Spain, Catalonia	100	63.5	15.5	65.1	53.6	75.1	63.7	63.5	15.3	65.2	53.8	74.9	36.3	63.5	15.9	65.0	53.5	75.6
Spain, Community of Madrid	100	62.8	15.7	64.2	53.1	74.7	63.4	63.2	15.6	64.6	54.0	74.9	36.6	62.2	15.9	63.3	51.5	74.5
Spain, Extremadura	100	64.5	14.2	65.3	55.6	74.8	63.7	64.9	13.8	66.0	56.5	74.8	36.3	63.8	14.9	64.5	54.6	74.8
Spain, Galicia	100	64.1	14.3	65.5	55.0	75.0	64.2	64.2	14.1	65.5	55.4	75.0	35.8	64.0	14.7	65.3	54.0	75.0
Spain, La Rioja	100	62.9	14.7	63.6	52.2	74.5	65.0	62.7	14.6	63.6	52.0	74.0	35.0	63.4	14.9	63.6	52.2	74.8
Spain, Murcia	100	63.2	15.2	64.3	53.9	74.7	65.0	63.4	15.0	64.6	54.5	74.8	35.0	62.6	15.6	63.9	53.5	74.5
Spain, Navarre *	100	63.6	14.5	65.5	53.6	74.6	64.7	64.3	14.0	66.1	54.5	74.5	35.3	62.5	15.5	64.1	51.7	75.0
Spain, Valencian region	100	64.2	15.2	66.0	54.7	75.7	63.2	64.0	15.3	66.0	54.5	75.5	36.8	64.6	15.0	66.0	54.8	76.0
Sweden	100	60.6	16.4	62.6	50.5	73.3	64.2	60.8	16.3	62.7	50.8	73.5	35.8	60.2	16.6	62.5	50.1	72.9
Switzerland	100	63.2	16.0	65.3	54.0	75.1	63.2	63.2	16.0	65.3	54.0	75.3	36.8	63.0	15.9	65.2	53.7	74.7
the Netherlands	100	61.3	15.6	63.3	52.0	73.1	60.8	61.3	15.5	63.3	52.0	73.2	39.2	61.2	15.6	63.4	52.2	73.0
United Kingdom, England	100	58.3	16.3	59.8	48.2	70.3	61.5	58.5	16.3	60.0	48.5	70.5	38.5	57.9	16.3	59.4	47.9	70.0
United Kingdom, Northern Ireland	100	58.9	16.7	60.1	48.0	71.5	61.0	58.7	16.8	60.0	48.0	71.2	39.0	59.1	16.4	60.5	48.5	72.2
United Kingdom, Scotland	100	57.7	15.3	59.5	48.7	68.9	59.8	57.7	15.6	59.5	48.4	69.1	39.3	57.9	14.8	59.5	49.2	68.4
United Kingdom, Wales	100	58.4	16.5	59.5	47.7	71.2	62.9	58.6	16.5	59.8	48.5	71.6	37.1	58.0	16.3	59.0	47.0	70.6

Categories may not add up because of missing values or rounding
** Patients younger than 20 years of age are not reported*

Table B.4.4
Prevalence per million population, adjusted
prevalent patients on December 31, adjusted for age and sex

	All	Male	Female
	Pmp	Pmp	Pmp
Austria	1034.1	1349.2	733.0
Belgium, Dutch-speaking *	1292.9	1580.7	1017.8
Belgium, French-speaking *	1641.7	2068.3	1233.9
Bosnia and Herzegovina	824.8	1047.3	612.1
Denmark	1020.6	1273.5	778.8
Estonia	907.3	1148.7	676.4
France (17 of 18 regions)	1440.1	1836.6	1060.9
Greece	1389.2	1877.9	922.1
Iceland	959.2	1224.7	705.4
Montenegro *	559.2	714.2	411.1
Norway	1067.9	1365.8	783.2
Romania	1377.1	1680.7	1086.9
Serbia	931.6	1200.1	675.0
Spain, Andalusia	1398.2	1752.0	1060.0
Spain, Aragon	1434.6	1937.2	954.1
Spain, Asturias	1256.2	1689.7	841.7
Spain, Basque country	1222.3	1603.1	858.2
Spain, Canary Islands	1652.1	2193.9	1134.2
Spain, Cantabria *	1118.9	1555.4	701.6
Spain, Castile and León *	1143.0	1504.5	797.5
Spain, Castile-La Mancha *	1258.1	1553.8	975.4
Spain, Catalonia	1607.3	2087.5	1148.3
Spain, Community of Madrid	1371.1	1822.5	939.6
Spain, Extremadura	1371.8	1755.9	1004.7
Spain, Galicia	1319.2	1735.0	921.7
Spain, La Rioja	1195.3	1559.3	847.4
Spain, Murcia	1657.4	2179.0	1158.7
Spain, Navarre *	1404.9	1823.8	1004.4
Spain, Valencian region	1490.5	1912.1	1087.6
Sweden	1059.5	1346.7	785.0
Switzerland	1059.5	1340.3	791.0
the Netherlands	1085.1	1313.8	866.5
United Kingdom, England	1149.5	1434.2	877.3
United Kingdom, Northern Ireland	1204.3	1484.2	936.6
United Kingdom, Scotland	1044.0	1290.7	808.3
United Kingdom, Wales	1091.6	1392.1	804.2

** Patients younger than 20 years of age are not reported*

Table B.4.5

Prevalence per million population and percentages by primary renal disease (1995 PRD codes), unadjusted

prevalent patients on December 31

	All		GN		PN		PKD		DM						HT		RVD		Misc		Unkn		Missing	
									Type 1		Type 2		Both											
	Pmp	%	Pmp	%	Pmp	%	Pmp	%	Pmp	%	Pmp	%	Pmp	%	Pmp	%	Pmp	%	Pmp	%	Pmp	%	Pmp	%
Austria	1017.1	100	220.8	21.7	65.3	6.4	83.1	8.2	49.0	4.8	139.1	13.7	188.1	18.5	113.2	11.1	31.9	3.1	204.8	20.1	109.2	10.7	0.7	0.1
Belgium, Dutch-speaking *	1289.4	100	223.4	17.3	80.5	6.2	136.5	10.6	47.1	3.7	162.2	12.6	209.4	16.2	110.2	8.5	35.9	2.8	351.9	27.3	141.7	11.0	0	0
Belgium, French-speaking *	1436.3	100	226.5	15.8	90.6	6.3	123.9	8.6	37.3	2.6	212.7	14.8	250.0	17.4	215.4	15.0	17.4	1.2	346.6	24.1	165.9	11.5	0	0
Bosnia and Herzegovina	691.3	100	132.8	19.2	82.4	11.9	40.2	5.8	38.5	5.6	89.2	12.9	127.7	18.5	91.2	13.2	6.5	0.9	105.1	15.2	105.3	15.2	0	0
Denmark	990.7	100	209.4	21.1	66.7	6.7	97.4	9.8	68.1	6.9	99.8	10.1	167.9	16.9	98.4	9.9	5.3	0.5	169.9	17.2	175.7	17.7	0	0
Estonia	842.2	100	215.7	25.6	92.7	11.0	82.3	9.8					152.0	18.0	128.3	15.2	46.7	5.5	123.8	14.7	0.7	0.1	0	0
France (17 of 18 regions)	1382.7	100	275.4	19.9	102.6	7.4	143.1	10.3					227.2	16.4	227.4	16.4	12.0	0.9	236.6	17.1	158.3	11.4	0	0
Greece	1479.4	100	240.1	16.2	88.9	6.0	120.5	8.1	24.9	1.7	236.6	16.0	261.5	17.7	137.2	9.3	19.5	1.3	163.4	11.0	448.2	30.3	0	0
Iceland	816.7	100	178.0	21.8	55.0	6.7	94.2	11.5	55.0	6.7	52.4	6.4	107.3	13.1	133.5	16.3	18.3	2.2	193.7	23.7	36.6	4.5	0	0
Montenegro *	494.2	100	51.8	10.5	21.1	4.3	17.8	3.6	27.5	5.6	64.8	13.1	92.4	18.7	168.5	34.1	0	0	74.5	15.1	68.1	13.8	0	0
Norway	997.2	100	277.6	27.8	64.5	6.5	113.8	11.4					129.7	13.0	175.4	17.6	4.9	0.5	199.6	20.0	27.1	2.7	4.6	0.5
Romania	1262.8	100	203.1	16.1	88.0	7.0	48.9	3.9					114.9	9.1	68.1	5.4	6.9	0.5	195.6	15.5	537.3	42.6	0	0
Serbia	940.0	100	162.0	17.2	86.6	9.2	60.3	6.4					165.4	17.6	240.0	25.5	9.6	1.0	142.6	15.2	69.7	7.4	3.8	0.4
Spain, Andalusia	1336.0	100	266.8	20.0	115.0	8.6	130.4	9.8					226.6	17.0	107.1	8.0	5.3	0.4	213.9	16.0	270.9	20.3	0	0
Spain, Aragon	1499.5	100	302.3	20.2	78.9	5.3	131.8	8.8	63.3	4.2	202.5	13.5	265.8	17.7	174.2	11.6	4.5	0.3	187.6	12.5	354.4	23.6	0	0
Spain, Asturias	1483.4	100	313.2	21.1	109.4	7.4	162.1	10.9	51.7	3.5	213.8	14.4	265.5	17.9	205.8	13.9	2.0	0.1	218.7	14.7	206.8	13.9	0	0
Spain, Basque country	1304.4	100	219.6	16.8	103.9	8.0	159.9	12.3	34.8	2.7	145.5	11.2	180.3	13.8	140.5	10.8	2.7	0.2	213.7	16.4	283.7	21.8	0	0
Spain, Canary Islands	1590.4	100	237.4	14.9	83.2	5.2	91.0	5.7	40.9	2.6	370.6	23.3	411.6	25.9	65.9	4.1	4.1	0.3	347.5	21.8	341.5	21.5	8.2	0.5
Spain, Cantabria *	1204.6	100	374.9	31.1	92.0	7.6	141.4	11.7	39.2	3.3	153.3	12.7	192.5	16.0	100.5	8.3	40.9	3.4	129.5	10.7	92.0	7.6	40.9	3.4
Spain, Castile and León * ‡	1334.0	100	255.4	19.1	88.9	6.7	121.8	9.1	57.3	4.3	161.0	12.1	218.3	16.4	147.9	11.1	2.5	0.2	212.8	16.0	286.2	21.5	0	0
Spain, Castile-La Mancha *	1233.0	100	240.2	19.5	133.9	10.9	146.5	11.9					214.1	17.4	60.9	4.9	21.3	1.7	157.1	12.7	256.7	20.8	2.4	0.2
Spain, Catalonia	1543.4	100	302.1	19.6	126.9	8.2	165.8	10.7	39.0	2.5	193.0	12.5	235.2	15.2	114.0	7.4	30.7	2.0	170.5	11.1	328.1	21.3	70.1	4.5
Spain, Community of Madrid	1277.8	100	277.3	21.7	84.5	6.6	143.3	11.2	44.1	3.5	172.9	13.5	217.1	17.0	127.9	10.0	12.0	0.9	220.0	17.2	165.3	12.9	30.4	2.4
Spain, Extremadura	1433.5	100	283.5	19.8	101.4	7.1	133.7	9.3					239.9	16.7	86.3	6.0	25.6	1.8	176.3	12.3	368.8	25.7	18.0	1.3
Spain, Galicia	1490.1	100	271.9	18.2	91.3	6.1	185.5	12.4	60.1	4.0	191.0	12.8	251.1	16.9	113.9	7.6	4.5	0.3	266.3	17.9	305.7	20.5	0	0
Spain, La Rioja	1236.8	100	342.7	27.7	46.7	3.8	121.5	9.8	43.6	3.5	118.4	9.6	162.0	13.1	99.7	8.1	3.1	0.3	380.1	30.7	81.0	6.5	0	0
Spain, Murcia	1455.7	100	376.0	25.8	110.3	7.6	122.7	8.4	44.4	3.0	196.5	13.5	240.9	16.5	99.2	6.8	1.3	0.1	250.0	17.2	255.2	17.5	0	0
Spain, Navarre *	1396.5	100	239.5	17.1	43.4	3.1	193.1	13.8	79.3	5.7	155.7	11.1	235.0	16.8	160.2	11.5	3.0	0.2	288.9	20.7	233.5	16.7	0	0
Spain, Valencian region	1487.3	100	193.2	13.0	116.9	7.9	139.7	9.4					218.1	14.7	187.5	12.6	5.5	0.4	247.7	16.7	378.6	25.5	0	0
Sweden ‡	1008.2	100	255.7	25.4	50.8	5.0	108.6	10.8					169.2	16.8	114.5	11.4	4.1	0.4	209.5	20.8	95.7	9.5	0	0
Switzerland	1022.5	100	201.3	19.7	39.6	3.9	34.2	3.3	22.3	2.2	124.8	12.2	147.1	14.4	132.9	13.0	16.9	1.7	339.0	33.2	103.0	10.1	8.5	0.8
the Netherlands	1064.9	100	175.5	16.5	62.2	5.8	92.8	8.7	39.0	3.7	101.2	9.5	140.1	13.2	130.1	12.2	47.3	4.4	254.8	23.9	141.5	13.3	20.7	1.9
United Kingdom, England #	1069.6	100	196.8	18.4	97.8	9.1	99.1	9.3					191.6	17.9	71.4	6.7	8.8	0.8	204.8	19.1	145.9	13.6	53.5	5.0
United Kingdom, Northern Ireland #	1098.6	100	213.0	19.4	164.4	15.0	134.5	12.2					152.3	13.9	32.5	3.0	7.9	0.7	228.2	20.8	133.5	12.1	32.5	3.0
United Kingdom, Scotland	1041.0	100	217.0	20.8	112.3	10.8	114.9	11.0					171.6	16.5	36.5	3.5	7.5	0.7	211.3	20.3	106.7	10.2	63.1	6.1
United Kingdom, Wales #	1072.9	100	266.6	24.9	105.7	9.9	108.9	10.1					199.9	18.6	38.6	3.6	7.7	0.7	217.8	20.3	119.7	11.2	8.0	0.7

Abbreviations used: GN: glomerulonephritis/sclerosis; PN: pyelonephritis; PKD: polycystic kidneys, adult type; DM: diabetes mellitus; HT: hypertension; RVD: renal vascular disease; Misc: miscellaneous; Unkn: unknown
Categories may not add up because of rounding or a limited number of cases (<10%) with diabetes mellitus type unknown; When cells are left empty, (complete) data are unavailable
** Patients younger than 20 years of age are not reported*
‡ Mapping the 2018 PRD codes to the old PRD codes results in a different distribution of PRD groups
# Values based on 1 to 5 patients are suppressed

Table B.4.6

Prevalence per million population by primary renal disease (1995 PRD codes), adjusted

prevalent patients on December 31, adjusted for age and sex

	All	GN	PN	PKD	DM			HT	RVD	Misc	Unkn	Missing
					Type 1	Type 2	Both					
	Pmp	Pmp	Pmp	Pmp	Pmp	Pmp	Pmp	Pmp	Pmp	Pmp	Pmp	Pmp
Austria	1034.1	221.6	65.7	84.7	49.0	144.9	193.9	115.9	33.2	207.1	111.2	0.7
Belgium, Dutch-speaking *	1292.9	225.1	81.4	138.7	47.9	160.9	208.9	109.3	35.1	351.8	142.5	0
Belgium, French-speaking *	1641.7	252.7	102.1	140.3	40.5	249.7	290.2	254.8	20.8	390.2	190.5	0
Bosnia and Herzegovina	824.8	141.4	98.6	45.6	43.1	113.4	156.5	113.7	7.5	133.4	128.1	0
Denmark	1020.6	218.4	68.0	101.7	72.1	100.8	173.0	99.9	5.5	174.1	180.0	0
Estonia	907.3	230.0	98.6	86.3			159.6	148.3	51.8	131.8	0.9	0
France (17 of 18 regions)	1440.1	290.6	107.5	150.5			235.2	233.1	12.5	246.4	164.4	0
Greece	1389.2	231.6	84.8	115.7	24.0	218.6	242.6	126.3	18.1	156.0	414.2	0
Iceland	959.2	203.5	68.0	115.2	61.0	62.6	123.5	173.8	27.0	211.7	36.5	0
Montenegro *	559.2	54.9	25.7	19.6	27.8	75.0	102.7	192.4	0	89.0	75.0	0
Norway	1067.9	293.4	68.4	126.0			139.5	192.4	5.5	209.6	28.4	4.7
Romania	1377.1	212.6	98.3	51.1			125.3	76.7	8.1	216.2	588.8	0
Serbia	931.6	158.2	87.6	58.8			161.3	239.2	9.5	141.5	71.5	4.0
Spain, Andalusia	1398.2	271.3	117.8	136.0			240.9	116.5	6.0	223.1	286.7	0
Spain, Aragon	1434.6	292.3	76.1	126.2	61.3	194.5	255.8	162.6	3.8	179.8	338.0	0
Spain, Asturias	1256.2	268.9	96.0	134.6	45.6	174.3	219.9	163.0	1.5	193.5	178.7	0
Spain, Basque country	1222.3	207.8	97.6	148.2	32.2	134.0	166.2	129.2	2.5	204.3	266.4	0
Spain, Canary Islands	1652.1	235.2	81.1	86.8	36.8	402.7	439.5	72.7	4.3	367.2	357.3	8.0
Spain, Cantabria *	1118.9	346.2	86.5	129.6	35.7	144.2	179.9	91.0	38.6	121.9	86.6	38.7
Spain, Castile and León * ‡	1143.0	226.4	77.7	107.8	52.1	130.1	182.2	119.6	2.0	186.0	241.4	0
Spain, Castile-La Mancha *	1258.1	242.4	134.7	153.7			219.9	61.4	21.1	160.1	262.5	2.4
Spain, Catalonia	1607.3	310.9	128.6	173.2	38.4	209.0	250.8	119.8	33.1	172.9	344.0	74.0
Spain, Community of Madrid	1371.1	292.5	87.3	154.8	43.4	197.7	241.1	140.3	13.3	228.3	180.5	33.1
Spain, Extremadura	1371.8	271.2	98.1	128.4			225.8	82.9	24.0	170.1	353.9	17.3
Spain, Galicia	1319.2	247.0	82.2	164.5	55.7	163.4	219.0	96.4	3.7	243.4	263.0	0
Spain, La Rioja	1195.3	329.9	44.4	117.7	40.3	115.6	155.9	95.8	2.5	370.2	78.8	0
Spain, Murcia	1657.4	419.5	120.6	132.5	42.6	237.8	280.4	121.6	1.7	283.3	297.8	0
Spain, Navarre *	1404.9	239.7	43.9	196.3	77.4	159.9	237.2	157.5	2.7	293.5	234.0	0
Spain, Valencian region	1490.5	190.8	115.8	138.1			219.7	191.8	5.5	248.1	380.8	0
Sweden ‡	1059.5	269.2	52.4	118.0			180.7	116.7	4.2	218.5	99.8	0
Switzerland	1059.5	205.7	41.1	36.1	23.2	130.6	153.8	138.6	17.8	350.9	106.7	8.8
the Netherlands	1085.1	178.3	63.8	94.4	40.4	102.3	142.7	133.5	48.3	258.5	144.6	21.2
United Kingdom, England #	1149.5	210.4	103.7	109.3			209.1	77.7	9.6	215.7	157.3	56.9
United Kingdom, Northern Ireland #	1204.3	232.0	175.7	149.5			169.0	36.9	9.3	247.6	149.6	34.7
United Kingdom, Scotland	1044.0	219.1	113.6	115.7			174.2	37.1	7.6	213.4	108.4	54.8
United Kingdom, Wales #	1091.6	272.8	110.4	111.2			203.4	38.8	7.5	218.7	120.7	8.0

Abbreviations used: GN: glomerulonephritis/sclerosis; PN: pyelonephritis; PKD: polycystic kidneys, adult type; DM: diabetes mellitus; HT: hypertension; RVD: renal vascular disease; Misc: miscellaneous; Unkn: unknown
Categories may not add up because of rounding or a limited number of cases (<10%) with diabetes mellitus type unknown; When cells are left empty, (complete) data are unavailable
** Patients younger than 20 years of age are not reported*
‡ Mapping the 2018 PRD codes to the old PRD codes results in a different distribution of PRD groups
# Values based on 1 to 5 patients are suppressed

Table B.4.7
Prevalent counts by treatment modality
prevalent patients on December 31

	All	Haemodialysis						Peritoneal dialysis				Kidney transplant				Unkn	Missing
		HD hospital/ centre	HD home	HD type Unkn	HF	HDF	Total HD/ HF/HDF	APD	CAPD	PD type Unkn	Total PD	Living donor	Deceased donor	Tx type Unkn	Total Tx		
	N	N	N	N	N	N	N	N	N	N	N	N	N	N	N	N	N
Austria §	8950	3162	1	5	8	1110	4286	176	118	5	299	817	3536	6	4359	6	0
Belgium, Dutch-speaking *	8702	2708	86	0	0	1768	4562	229	125	0	354	355	3410	21	3786	0	0
Belgium, French-speaking *	7083	2159	147	0	46	1459	3811	151	135	0	286	282	2528	167	2977	9	0
Bosnia and Herzegovina	2441	1164	0	0	0	785	1949	1	69	3	73	268	143	8	419	0	0
Denmark	5848	1889	166	0			2055	253	239	24	516	1247	2025	0	3272	5	0
Estonia	1136	382	0	0	0	0	382	43	12	0	55	33	666	0	699	0	0
France (17 of 18 regions)	93486	30157	725	85	74	17295	48336	1071	1890	18	2979	6023	34429	1245	41697	474	0
Greece	15440	7990	1	0	0	3958	11949	402	220	0	622	1233	1636	0	2869	0	0
Iceland	312	76	0	0			76	0	12	0	12	122	102	0	224	0	0
Montenegro *	305	289	0	0	0	6	295	0	0	0	0	9	1	0	10	0	0
Norway	5442	1365	47	0			1412			403	403	1340	2268	7	3615	12	0
Romania ||	24054	19777	0	0	23	1204	21004	58	957	0	1015	541	909	576	2026	9	0
Serbia	6000	3843	34	17	26	709	4629	57	479	4	540	445	369	3	817	7	7
Spain, Andalusia	11412	4579	52	0			4631			422	422	613	5736	10	6359	0	0
Spain, Aragon	2014	480	5	0		139	624	22	67	0	89	120	1180	0	1300	1	0
Spain, Asturias	1492	549	8	0	0	13	570	18	60	0	78	47	795	2	844	0	0
Spain, Basque country	2887	787	21	0			808	19	142	0	161	248	1670	0	1918	0	0
Spain, Canary Islands	3497	1617	3	0	0	0	1620	0	0	263	263	68	1546	0	1614	0	0
Spain, Cantabria *	707	227	4	0			231	5	22	0	27	23	425	0	448	1	0
Spain, Castile and León *	3165	1208	18	0			1226	77	65	0	142	111	1619	0	1730	67	0
Spain, Castile-La Mancha *	2551	989	3	0			992	22	116	0	138	44	1326	50	1420	1	0
Spain, Catalonia	12027	1028	54	0		3169	4251	166	241	0	407	1588	5781	0	7369	0	0
Spain, Community of Madrid	8194	2861	47	0		250	3158	141	263	0	404	426	4196	0	4622	10	0
Spain, Extremadura	1512	798	14	0			812	29	20	0	49	29	399	222	650	1	0
Spain, Galicia	4017	1711	25	0	0	0	1736	107	160	0	267	308	1630	76	2014	0	0
Spain, La Rioja	397	141	1	0	0	0	142	12	12	0	24	14	217	0	231	0	0
Spain, Murcia	2230	436	0	0	0	664	1100	16	92	0	108	73	949	0	1022	0	0
Spain, Navarre *	933	298	23	0			321	45	2	0	47	40	525	0	565	0	0
Spain, Valencian region	7582	3506	103	0			3609	79	355	0	434	160	2919	443	3522	17	0
Sweden	10573	3118	107	0			3225			958	958	2578	3811	0	6389	1	0
Switzerland	8885	3496	68	0	71	0	3635	155	148	0	303	1905	3027	5	4937	10	0
the Netherlands &	18096	4248	254	0		722	5224	633	334	0	967	6458	5436	9	11903	2	0
United Kingdom, England ¶ #	55950	15508	1207	8	15	4989	21727	1858	1295	43	3196	9920	20363	736	31019	8	0
United Kingdom, Northern Ireland #	2099	531	13	0	0	9	553	48	16		65	672	752	57	1481	0	0
United Kingdom, Scotland	5671	1989	47	0			2036	135	82	10	227	1121	2191	92	3404	4	0
United Kingdom, Wales #	3360	1107	124	0	0	116	1347	84	64		149	604	1243	17	1864	0	0

Abbreviations used: HD: haemodialysis; Unkn: unknown; HF: haemofiltration; HDF: haemodiafiltration; APD: automated peritoneal dialysis; CAPD: continuous ambulatory peritoneal dialysis; PD: peritoneal dialysis; Tx: transplant
When cells are left empty, (complete) data are unavailable
§ The prevalence is underestimated by approximately 2% due to an estimated 2% underestimation of the prevalence of haemodialysis
** Patients younger than 20 years of age are not reported*
|| The prevalence of patients living on a functioning graft is underestimated by approximately 30%
& The prevalence is underestimated by approximately 4%
¶ The prevalence is underestimated by approximately 8.4% due to one centre not providing data and patients opting out of data sharing for research purposes
# Values based on 1 to 5 patients are suppressed

Table B.4.8
Prevalence per million population by treatment modality, unadjusted
prevalent patients on December 31

	All	Haemodialysis						Peritoneal dialysis				Kidney transplant				Unkn	Missing
		HD hospital/ centre	HD home	HD type Unkn	HF	HDF	Total HD/ HF/HDF	APD	CAPD	PD type Unkn	Total PD	Living donor	Deceased donor	Tx type Unkn	Total Tx		
	Pmp	Pmp	Pmp	Pmp	Pmp	Pmp	Pmp	Pmp	Pmp	Pmp	Pmp	Pmp	Pmp	Pmp	Pmp	Pmp	Pmp
Austria	1017.1	359.3	0.1	0.6	0.9	126.1	487.1	20.0	13.4	0.6	34.0	92.8	401.8	0.7	495.4	0.7	0
Belgium, Dutch-speaking *	1289.4	401.3	12.7	0	0	262.0	676.0	33.9	18.5	0	52.5	52.6	505.3	3.1	561.0	0	0
Belgium, French-speaking *	1436.3	437.8	29.8	0	9.3	295.9	772.8	30.6	27.4	0	58.0	57.2	512.6	33.9	603.7	1.8	0
Bosnia and Herzegovina	691.3	329.6	0	0	0	222.3	551.9	0.3	19.5	0.8	20.7	75.9	40.5	2.3	118.7	0	0
Denmark	990.7	320.0	28.1	0			348.1	42.9	40.5	4.1	87.4	211.2	343.0	0	554.3	0.8	0
Estonia	842.2	283.2	0	0	0	0	283.2	31.9	8.9	0	40.8	24.5	493.8	0	518.2	0	0
France (17 of 18 regions)	1382.7	446.0	10.7	1.3	1.1	255.8	714.9	15.8	28.0	0.3	44.1	89.1	509.2	18.4	616.7	7.0	0
Greece	1479.4	765.6	0.1	0	0	379.2	1144.9	38.5	21.1	0	59.6	118.1	156.8	0	274.9	0	0
Iceland	816.7	199.0	0	0			199.0	0	31.4	0	31.4	319.4	267.0	0	586.4	0	0
Montenegro *	494.2	468.3	0	0	0	9.7	478.0	0	0	0	0	14.6	1.6	0	16.2	0	0
Norway	997.2	250.1	8.6	0			258.7			73.8	73.8	245.6	415.6	1.3	662.4	2.2	0
Romania ||	1262.8	1038.2	0	0	1.2	63.2	1102.7	3.0	50.2	0	53.3	28.4	47.7	30.2	106.4	0.5	0
Serbia	940.0	602.0	5.3	2.7	4.1	111.1	725.2	8.9	75.0	0.6	84.6	69.7	57.8	0.5	128.0	1.1	1.1
Spain, Andalusia	1336.0	536.0	6.1	0			542.1			49.4	49.4	71.8	671.5	1.2	744.4	0	0
Spain, Aragon	1499.5	357.4	3.7	0		103.5	464.6	16.4	49.9	0	66.3	89.3	878.6	0	967.9	0.7	0
Spain, Asturias	1483.4	545.8	8.0	0	0	12.9	566.7	17.9	59.7	0	77.5	46.7	790.4	2.0	839.1	0	0
Spain, Basque country	1304.4	355.6	9.5	0			365.1	8.6	64.2	0	72.7	112.1	754.5	0	866.6	0	0
Spain, Canary Islands	1590.4	735.4	1.4	0	0	0	736.7	0	0	119.6	119.6	30.9	703.1	0	734.0	0	0
Spain, Cantabria *	1204.6	386.8	6.8	0			393.6	8.5	37.5	0	46.0	39.2	724.1	0	763.3	1.7	0
Spain, Castile and León *	1334.0	509.1	7.6	0			516.7	32.5	27.4	0	59.8	46.8	682.4	0	729.1	28.2	0
Spain, Castile-La Mancha *	1233.0	478.0	1.5	0			479.5	10.6	56.1	0	66.7	21.3	640.9	24.2	686.4	0.5	0
Spain, Catalonia	1543.4	131.9	6.9	0		406.7	545.5	21.3	30.9	0	52.2	203.8	741.9	0	945.6	0	0
Spain, Community of Madrid	1277.8	446.1	7.3	0		39.0	492.5	22.0	41.0	0	63.0	66.4	654.3	0	720.7	1.6	0
Spain, Extremadura	1433.5	756.6	13.3	0			769.8	27.5	19.0	0	46.5	27.5	378.3	210.5	616.2	0.9	0
Spain, Galicia	1490.1	634.7	9.3	0	0	0	644.0	39.7	59.4	0	99.0	114.3	604.6	28.2	747.1	0	0
Spain, La Rioja	1236.8	439.3	3.1	0	0	0	442.4	37.4	37.4	0	74.8	43.6	676.0	0	719.6	0	0
Spain, Murcia	1455.7	284.6	0	0	0	433.5	718.1	10.4	60.1	0	70.5	47.7	619.5	0	667.2	0	0
Spain, Navarre *	1396.5	446.1	34.4	0			480.5	67.4	3.0	0	70.4	59.9	785.8	0	845.7	0	0
Spain, Valencian region	1487.3	687.7	20.2	0			707.9	15.5	69.6	0	85.1	31.4	572.6	86.9	690.9	3.3	0
Sweden	1008.2	297.3	10.2	0			307.5			91.4	91.4	245.8	363.4	0	609.2	0.1	0
Switzerland	1022.5	402.3	7.8	0	8.2	0	418.3	17.8	17.0	0	34.9	219.2	348.4	0.6	568.2	1.2	0
the Netherlands	1064.9	250.0	14.9	0		42.5	307.4	37.3	19.7	0	56.9	380.0	319.9	0.5	700.5	0.1	0
United Kingdom, England #	1069.6	296.5	23.1	0.2	0.3	95.4	415.4	35.5	24.8	0.8	61.1	189.6	389.3	14.1	593.0	0.2	0
United Kingdom, Northern Ireland #	1098.6	277.9	6.8	0	0	4.7	289.4	25.1	8.4		34.0	351.7	393.6	29.8	775.2	0	0
United Kingdom, Scotland	1041.0	365.1	8.6	0			373.7	24.8	15.1	1.8	41.7	205.8	402.2	16.9	624.9	0.7	0
United Kingdom, Wales #	1072.9	353.5	39.6	0	0	37.0	430.1	26.8	20.4		47.6	192.9	396.9	5.4	595.2	0	0

Abbreviations used: HD: haemodialysis; Unkn: unknown; HF: haemofiltration; HDF: haemodiafiltration; APD: automated peritoneal dialysis; CAPD: continuous ambulatory peritoneal dialysis; PD: peritoneal dialysis; Tx: transplant
Categories may not add up because of rounding; When cells are left empty, (complete) data are unavailable
** Patients younger than 20 years of age are not reported*
|| The prevalence of patients living on a functioning graft is underestimated by approximately 30%
# Values based on 1 to 5 patients are suppressed

Table B.4.9

Prevalence per million population by treatment modality, adjusted

prevalent patients on December 31, adjusted for age and sex

	All	Haemodialysis						Peritoneal dialysis				Kidney transplant				Unkn	Missing
		HD hospital/ centre	HD home	HD type Unkn	HF	HDF	Total HD/ HF/HDF	APD	CAPD	PD type Unkn	Total PD	Living donor	Deceased donor	Tx type Unkn	Total Tx		
	Pmp	Pmp	Pmp	Pmp	Pmp	Pmp	Pmp	Pmp	Pmp	Pmp	Pmp	Pmp	Pmp	Pmp	Pmp	Pmp	Pmp
Austria	1034.1	370.6	0.1	0.5	0.9	128.1	500.2	20.1	13.6	0.6	34.3	91.9	406.4	0.6	498.9	0.7	0
Belgium, Dutch-speaking *	1292.9	398.2	12.9	0	0	259.0	670.1	34.1	18.5	0	52.7	53.9	513.1	3.2	570.2	0	0
Belgium, French-speaking *	1641.7	513.8	32.5	0	10.8	345.4	902.5	34.8	31.3	0	66.1	60.3	571.9	38.9	671.0	2.0	0
Bosnia and Herzegovina	824.8	401.3	0	0	0	278.5	679.7	0.4	24.5	1.0	25.9	75.0	41.9	2.2	119.2	0	0
Denmark	1020.6	327.5	29.5	0			357.0	42.7	41.0	4.1	87.8	218.8	356.0	0	574.9	0.9	0
Estonia	907.3	314.2	0	0	0	0	314.2	34.8	9.2	0	44.0	24.9	524.2	0	549.1	0	0
France (17 of 18 regions)	1440.1	459.6	11.4	1.3	1.1	263.0	736.5	16.4	28.6	0.3	45.2	94.5	537.3	19.2	651.0	7.3	0
Greece	1389.2	699.9	0.1	0	0	364.1	1064.0	36.9	19.8	0	56.7	116.3	152.2	0	268.5	0	0
Iceland	959.2	256.0	0	0			256.0	0	36.0	0	36.0	363.4	303.8	0	667.2	0	0
Montenegro *	559.2	533.4	0	0	0	10.2	543.6	0	0	0	0	14.0	1.6	0	15.6	0	0
Norway	1067.9	273.4	9.1	0			282.6			80.5	80.5	256.2	445.1	1.4	702.7	2.2	0
Romania ||	1377.1	1142.9	0	0	1.4	64.8	1209.1	3.4	58.4	0	61.8	27.8	47.4	30.6	105.8	0.4	0
Serbia	931.6	597.7	5.2	2.6	4.0	109.9	719.4	9.0	74.8	0.6	84.4	68.9	56.3	0.5	125.6	1.0	1.2
Spain, Andalusia	1398.2	584.4	6.2	0			590.6			53.4	53.4	70.7	682.3	1.2	754.2	0	0
Spain, Aragon	1434.6	338.3	3.5	0		98.6	440.4	16.0	45.8	0	61.8	87.8	843.8	0	931.6	0.8	0
Spain, Asturias	1256.2	445.9	7.3	0	0	10.9	464.1	16.1	48.8	0	64.8	44.6	680.9	1.7	727.2	0	0
Spain, Basque country	1222.3	328.7	9.2	0			337.9	8.4	59.8	0	68.2	108.3	707.9	0	816.2	0	0
Spain, Canary Islands	1652.1	803.6	1.6	0	0	0	805.2	0	0	124.9	124.9	28.0	694.0	0	722.1	0	0
Spain, Cantabria *	1118.9	360.3	7.8	0			368.1	8.6	34.8	0	43.4	37.6	668.2	0	705.9	1.5	0
Spain, Castile and León *	1143.0	416.3	7.0	0			423.3	28.1	23.3	0	51.3	44.9	605.2	0	650.1	18.2	0
Spain, Castile-La Mancha *	1258.1	490.0	1.3	0			491.4	10.3	57.8	0	68.2	21.3	652.2	24.6	698.1	0.5	0
Spain, Catalonia	1607.3	139.9	7.0	0		428.4	575.2	22.4	32.5	0	54.9	205.3	771.8	0	977.2	0	0
Spain, Community of Madrid	1371.1	490.7	7.7	0		42.5	540.8	22.7	44.6	0	67.3	66.1	695.1	0	761.2	1.7	0
Spain, Extremadura	1371.8	721.6	12.7	0			734.2	26.1	18.3	0	44.4	26.9	363.9	201.5	592.3	0.9	0
Spain, Galicia	1319.2	538.8	8.3	0	0	0	547.0	36.1	51.4	0	87.5	106.6	547.7	30.3	684.7	0	0
Spain, La Rioja	1195.3	421.5	3.1	0	0	0	424.6	35.4	37.7	0	73.1	43.2	654.4	0	697.6	0	0
Spain, Murcia	1657.4	335.4	0	0	0	514.4	849.7	11.5	69.3	0	80.8	49.1	677.7	0	726.8	0	0
Spain, Navarre *	1404.9	449.0	34.7	0			483.7	67.7	3.3	0	71.0	59.0	791.2	0	850.2	0	0
Spain, Valencian region	1490.5	699.4	19.8	0			719.3	15.2	70.2	0	85.4	30.8	565.1	86.6	682.5	3.3	0
Sweden	1059.5	308.0	11.1	0			319.1			92.4	92.4	259.8	388.1	0	647.9	0.1	0
Switzerland	1059.5	421.9	8.0	0	8.2	0	438.1	18.2	17.6	0	35.8	223.1	360.8	0.6	584.5	1.1	0
the Netherlands	1085.1	257.7	15.5	0		43.8	316.9	37.9	20.2	0	58.0	384.9	324.6	0.5	710.1	0.1	0
United Kingdom, England #	1149.5	321.6	24.6	0.2	0.3	103.6	450.3	37.8	26.6	0.8	65.1	200.2	418.9	14.9	633.9	0.2	0
United Kingdom, Northern Ireland #	1204.3	318.2	7.4	0	0	5.5	331.1	28.1	10.0		38.7	373.9	428.8	31.8	834.5	0	0
United Kingdom, Scotland	1044.0	363.0	8.7	0			371.7	25.2	14.5	1.1	40.7	208.2	406.4	16.3	630.9	0.7	0
United Kingdom, Wales #	1091.6	352.5	40.3	0	0	36.7	429.5	27.1	20.7		48.1	200.0	407.8	6.1	614.0	0	0

Abbreviations used: HD: haemodialysis; Unkn: unknown; HF: haemofiltration; HDF: haemodiafiltration; APD: automated peritoneal dialysis; CAPD: continuous ambulatory peritoneal dialysis; PD: peritoneal dialysis; Tx: transplant
Categories may not add up because of rounding; When cells are left empty, (complete) data are unavailable
** Patients younger than 20 years of age are not reported*
|| The prevalence of patients living on a functioning graft is underestimated by approximately 30%
# Values based on 1 to 5 patients are suppressed

Table B.4.10
Treatment modality distribution, unadjusted
prevalent patients on December 31

	All	Haemodialysis						Peritoneal dialysis				Kidney transplant				Unkn	Missing
		HD hospital/ centre	HD home	HD type Unkn	HF	HDF	Total HD/ HF/HDF	APD	CAPD	PD type Unkn	Total PD	Living donor	Deceased donor	Tx type Unkn	Total Tx		
	%	%	%	%	%	%	%	%	%	%	%	%	%	%	%	%	%
Austria	100	35.3	0	0.1	0.1	12.4	47.9	2.0	1.3	0.1	3.3	9.1	39.5	0.1	48.7	0.1	0
Belgium, Dutch-speaking *	100	31.1	1.0	0	0	20.3	52.4	2.6	1.4	0	4.1	4.1	39.2	0.2	43.5	0	0
Belgium, French-speaking *	100	30.5	2.1	0	0.6	20.6	53.8	2.1	1.9	0	4.0	4.0	35.7	2.4	42.0	0.1	0
Bosnia and Herzegovina	100	47.7	0	0	0	32.2	79.8	0	2.8	0.1	3.0	11.0	5.9	0.3	17.2	0	0
Denmark	100	32.3	2.8	0			35.1	4.3	4.1	0.4	8.8	21.3	34.6	0	56.0	0.1	0
Estonia	100	33.6	0	0	0	0	33.6	3.8	1.1	0	4.8	2.9	58.6	0	61.5	0	0
France (17 of 18 regions)	100	32.3	0.8	0.1	0.1	18.5	51.7	1.1	2.0	0	3.2	6.4	36.8	1.3	44.6	0.5	0
Greece	100	51.7	0	0	0	25.6	77.4	2.6	1.4	0	4.0	8.0	10.6	0	18.6	0	0
Iceland	100	24.4	0	0			24.4	0	3.8	0	3.8	39.1	32.7	0	71.8	0	0
Montenegro *	100	94.8	0	0	0	2.0	96.7	0	0	0	0	3.0	0.3	0	3.3	0	0
Norway	100	25.1	0.9	0			25.9			7.4	7.4	24.6	41.7	0.1	66.4	0.2	0
Romania ||	100	82.2	0	0	0.1	5.0	87.3	0.2	4.0	0	4.2	2.2	3.8	2.4	8.4	0	0
Serbia	100	64.1	0.6	0.3	0.4	11.8	77.2	1.0	8.0	0.1	9.0	7.4	6.2	0.1	13.6	0.1	0.1
Spain, Andalusia	100	40.1	0.5	0			40.6			3.7	3.7	5.4	50.3	0.1	55.7	0	0
Spain, Aragon	100	23.8	0.2	0		6.9	31.0	1.1	3.3	0	4.4	6.0	58.6	0	64.5	0	0
Spain, Asturias	100	36.8	0.5	0	0	0.9	38.2	1.2	4.0	0	5.2	3.2	53.3	0.1	56.6	0	0
Spain, Basque country	100	27.3	0.7	0			28.0	0.7	4.9	0	5.6	8.6	57.8	0	66.4	0	0
Spain, Canary Islands	100	46.2	0.1	0	0	0	46.3	0	0	7.5	7.5	1.9	44.2	0	46.2	0	0
Spain, Cantabria *	100	32.1	0.6	0			32.7	0.7	3.1	0	3.8	3.3	60.1	0	63.4	0.1	0
Spain, Castile and León *	100	38.2	0.6	0			38.7	2.4	2.1	0	4.5	3.5	51.2	0	54.7	2.1	0
Spain, Castile-La Mancha *	100	38.8	0.1	0			38.9	0.9	4.5	0	5.4	1.7	52.0	2.0	55.7	0	0
Spain, Catalonia	100	8.5	0.4	0		26.3	35.3	1.4	2.0	0	3.4	13.2	48.1	0	61.3	0	0
Spain, Community of Madrid	100	34.9	0.6	0		3.1	38.5	1.7	3.2	0	4.9	5.2	51.2	0	56.4	0.1	0
Spain, Extremadura	100	52.8	0.9	0			53.7	1.9	1.3	0	3.2	1.9	26.4	14.7	43.0	0.1	0
Spain, Galicia	100	42.6	0.6	0	0	0	43.2	2.7	4.0	0	6.6	7.7	40.6	1.9	50.1	0	0
Spain, La Rioja	100	35.5	0.3	0	0	0	35.8	3.0	3.0	0	6.0	3.5	54.7	0	58.2	0	0
Spain, Murcia	100	19.6	0	0	0	29.8	49.3	0.7	4.1	0	4.8	3.3	42.6	0	45.8	0	0
Spain, Navarre *	100	31.9	2.5	0			34.4	4.8	0.2	0	5.0	4.3	56.3	0	60.6	0	0
Spain, Valencian region	100	46.2	1.4	0			47.6	1.0	4.7	0	5.7	2.1	38.5	5.8	46.5	0.2	0
Sweden	100	29.5	1.0	0			30.5			9.1	9.1	24.4	36.0	0	60.4	0	0
Switzerland	100	39.3	0.8	0	0.8	0	40.9	1.7	1.7	0	3.4	21.4	34.1	0.1	55.6	0.1	0
the Netherlands	100	23.5	1.4	0		4.0	28.9	3.5	1.8	0	5.3	35.7	30.0	0	65.8	0	0
United Kingdom, England #	100	27.7	2.2	0	0	8.9	38.8	3.3	2.3	0.1	5.7	17.7	36.4	1.3	55.4	0	0
United Kingdom, Northern Ireland #	100	25.3	0.6	0	0	0.4	26.3	2.3	0.8		3.1	32.0	35.8	2.7	70.6	0	0
United Kingdom, Scotland	100	35.1	0.8	0			35.9	2.4	1.4	0.2	4.0	19.8	38.6	1.6	60.0	0.1	0
United Kingdom, Wales #	100	32.9	3.7	0	0	3.5	40.1	2.5	1.9		4.4	18.0	37.0	0.5	55.5	0	0

Abbreviations used: HD: haemodialysis; Unkn: unknown; HF: haemofiltration; HDF: haemodiafiltration; APD: automated peritoneal dialysis; CAPD: continuous ambulatory peritoneal dialysis; PD: peritoneal dialysis; Tx: transplant
Categories may not add up because of rounding; When cells are left empty, (complete) data are unavailable
** Patients younger than 20 years of age are not reported*
|| The prevalence of patients living on a functioning graft is underestimated by approximately 30%
# Values based on 1 to 5 patients are suppressed

Table B.4.11

Treatment modality distribution by age, sex, and primary renal disease, unadjusted

prevalent patients on December 31

	All				0-19				20-44				45-64				65-74				75+				Male				Female				DM				Non-DM			
	HD	PD	Tx	Un	HD	PD	Tx	Un	HD	PD	Tx	Un	HD	PD	Tx	Un	HD	PD	Tx	Un	HD	PD	Tx	Un	HD	PD	Tx	Un	HD	PD	Tx	Un	HD	PD	Tx	Un	HD	PD	Tx	Un
	%	%	%	%	%	%	%	%	%	%	%	%	%	%	%	%	%	%	%	%	%	%	%	%	%	%	%	%	%	%	%	%	%	%	%	%	%	%	%	%
Austria	48	3	49	0	8	4	89	0	32	4	64	0	36	4	60	0	52	3	45	0	76	3	21	0	48	3	48	0	47	3	50	0	65	2	33	0	44	4	52	0
Belgium, Dutch-speaking *	52	4	44	0					28	6	65	0	33	4	63	0	48	4	48	0	82	4	14	0	52	4	43	0	53	4	44	0	70	4	26	0	49	4	47	0
Belgium, French-speaking *	54	4	42	0					38	5	57	0	40	3	57	0	54	4	41	0	74	4	22	0	53	4	43	0	54	4	41	0	70	4	26	0	50	4	45	0
Bosnia and Herzegovina	80	3	17	0	56	0	44	0	57	2	41	0	76	4	20	0	91	2	7	0	95	4	1	0	79	2	19	0	82	4	14	0	88	5	6	0	78	2	20	0
Denmark	35	9	56	0	4	7	89	0	21	6	73	0	28	7	65	0	40	9	51	0	60	17	23	0	35	9	55	0	35	8	57	0	52	10	37	0	32	8	60	0
Estonia	34	5	62	0	0	8	92	0	24	4	73	0	25	3	71	0	37	7	55	0	60	6	34	0	33	5	62	0	34	5	61	0	34	6	60	0	34	5	62	0
France (17 of 18 regions)	52	3	45	1	18	5	76	0	31	2	66	0	39	2	58	1	56	3	40	1	76	5	18	1	52	3	44	0	51	3	45	1	74	3	23	0	47	3	49	1
Greece	77	4	19	0	30	15	55	0	48	5	47	0	67	4	29	0	81	5	14	0	93	3	3	0	78	4	18	0	75	5	20	0	92	3	4	0	74	4	22	0
Iceland	24	4	72	0	13	13	75	0	16	5	80	0	14	2	84	0	23	5	72	0	64	6	30	0	26	4	70	0	22	3	75	0	39	5	56	0	22	4	74	0
Montenegro *	97	0	3	0					85	0	15	0	98	0	2	0	99	0	1	0	100	0	0	0	97	0	3	0	96	0	4	0	100	0	0	0	96	0	4	0
Norway	26	7	66	0	2	2	94	1	14	6	80	1	21	6	73	0	28	8	64	0	45	12	43	0	27	8	65	0	24	7	69	0	36	9	54	0	24	7	68	0
Romania ||	87	4	8	0	62	11	27	0	72	3	24	0	84	3	13	0	93	4	3	0	93	6	1	0	87	4	9	0	88	5	7	0	91	6	3	0	87	4	9	0
Serbia	77	9	14	0	25	41	33	0	57	8	34	0	72	9	18	0	85	8	6	0	91	8	1	0	78	8	13	0	75	11	14	0	79	14	6	0	77	8	15	0
Spain, Andalusia	41	4	56	0	13	3	84	0	28	3	69	0	29	3	69	0	43	4	53	0	69	5	26	0	40	4	57	0	42	4	54	0	60	4	35	0	37	4	60	0
Spain, Aragon	31	4	65	0	6	13	81	0	22	4	74	0	23	4	72	0	30	4	66	0	43	5	52	0	32	5	63	0	28	4	68	0	49	4	47	0	27	4	68	0
Spain, Asturias	38	5	57	0	10	10	80	0	18	1	81	0	27	4	69	0	38	7	55	0	61	6	33	0	38	5	57	0	39	5	56	0	53	9	37	0	35	4	61	0
Spain, Basque country	28	6	66	0	6	3	91	0	24	4	72	0	20	5	75	0	25	6	69	0	49	8	44	0	29	6	65	0	26	5	69	0	53	7	41	0	24	5	71	0
Spain, Canary Islands	46	8	46	0	33	22	44	0	33	8	59	0	35	7	58	0	47	9	43	0	72	7	21	0	46	7	47	0	47	8	45	0	60	8	32	0	41	8	51	0
Spain, Cantabria *	33	4	63	0					25	1	74	0	16	3	81	0	30	4	65	1	66	6	28	0	34	3	63	0	30	6	64	0	44	4	51	0	30	4	66	0
Spain, Castile and León *	39	4	55	2					26	4	70	1	25	4	70	0	35	5	59	1	61	5	29	5	40	5	53	2	37	4	57	3	55	4	37	4	36	5	58	2
Spain, Castile-La Mancha *	39	5	56	0					23	7	70	0	28	6	65	0	39	6	55	0	61	3	35	0	40	5	55	0	37	6	57	0	56	7	37	0	35	5	60	0
Spain, Catalonia	35	3	61	0	8	1	91	0	22	3	75	0	23	3	74	0	34	4	62	0	62	4	34	0	36	3	61	0	35	3	62	0	51	3	45	0	32	3	64	0
Spain, Community of Madrid	39	5	56	0	6	2	92	0	27	6	67	0	28	5	67	0	39	5	56	0	62	4	33	0	40	5	55	0	36	5	60	0	59	3	38	0	34	5	60	0
Spain, Extremadura	54	3	43	0	29	0	71	0	31	2	67	0	41	5	54	0	58	3	39	0	78	2	20	0	55	3	42	0	52	3	45	0	72	3	25	0	50	3	47	0
Spain, Galicia	43	7	50	0	10	10	81	0	26	6	68	0	29	6	64	0	45	7	48	0	70	7	22	0	44	6	50	0	42	7	51	0	64	6	31	0	39	7	54	0
Spain, La Rioja	36	6	58	0	0	0	100	0	28	7	65	0	22	6	72	0	29	10	61	0	70	2	28	0	34	6	60	0	39	6	55	0	60	8	33	0	32	6	62	0
Spain, Murcia	49	5	46	0	23	0	77	0	36	9	55	0	35	4	61	0	50	6	44	0	79	4	17	0	50	4	46	0	49	6	46	0	72	4	23	0	45	5	50	0
Spain, Navarre *	34	5	61	0					26	4	70	0	21	4	75	0	35	7	58	0	58	5	36	0	36	4	59	0	31	6	63	0	57	6	38	0	30	5	65	0
Spain, Valencian region	48	6	46	0	14	6	80	0	25	6	69	0	35	6	59	0	47	6	46	0	74	5	20	0	48	6	46	0	47	5	48	0	65	6	28	0	45	6	50	0
Sweden	31	9	60	0	4	6	90	0	20	6	75	0	23	6	72	0	33	9	58	0	52	18	30	0	30	10	60	0	31	8	61	0	46	12	43	0	27	9	64	0
Switzerland	41	3	56	0	6	11	82	1	22	4	74	0	28	3	69	0	40	3	56	0	71	4	25	0	42	4	55	0	40	3	57	0	59	4	37	0	38	3	59	0
the Netherlands	29	5	66	0	8	4	88	0	17	3	79	0	21	4	74	0	29	5	65	0	53	9	38	0	29	5	66	0	29	6	65	0	48	8	45	0	26	5	69	0
United Kingdom, England	39	6	55	0	14	11	75	0	27	5	68		33	5	62	0	42	6	52		67	8	25	0	40	5	55		38	6	56		58	7	35	0	35	5	60	0
United Kingdom, Northern Ireland	26	3	71	0			83	0	11	2	87	0	17	2	81	0	30	2	68	0	59	8	33	0	27	3	70	0	26	3	71	0	46	3	50	0	23	3	74	0
United Kingdom, Scotland	36	4	60	0	13	8	79	0	22	4	74	0	29	3	68	0	44	3	53	0	66	7	27	0	36	4	60	0	35	4	61	0	53	4	43	0	32	4	63	0
United Kingdom, Wales	40	4	55	0	11		82	0	29	5	67	0	34	3	63	0	45	5	50	0	66	6	29	0	40	4	56	0	40	4	55	0	59	5	35	0	36	4	60	0

Abbreviations used: HD: haemodialysis; PD: peritoneal dialysis; Tx: transplant; Un: unknown; DM: diabetes mellitus
Categories may not add up because of missing values or rounding
** Patients younger than 20 years of age are not reported*
|| The prevalence of patients living on a functioning graft is underestimated by approximately 30%

Table B.5.1
Kidney transplant counts and percentages by donor type

	All		Living donor								Deceased donor		Donor type Unkn	
			Related		Unrelated		Type Unkn		All					
	N	%	N	%	N	%	N	%	N	%	N	%	N	%
Austria §	310	100	25	8.1	23	7.4	0	0	48	15.5	262	84.5	0	0
Belgium, Dutch-speaking *	234	100	16	6.8	10	4.3	0	0	26	11.1	186	79.5	22	9.4
Belgium, French-speaking *	184	100	10	5.4	5	2.7	0	0	15	8.2	167	90.8	2	1.1
Bosnia and Herzegovina	15	100	10	66.7	1	6.7	0	0	11	73.3	3	20.0	1	6.7
Denmark	242	100	51	21.1	27	11.2	0	0	78	32.2	164	67.8	0	0
Estonia	35	100	4	11.4	0	0	0	0	4	11.4	31	88.6	0	0
France (17 of 18 regions)	3352	100	299	8.9	215	6.4	0	0	514	15.3	2833	84.5	5	0.1
Greece	194	100	61	31.4	21	10.8	0	0	82	42.3	112	57.7	0	0
Iceland	8	100	3	37.5	0	0	0	0	3	37.5	5	62.5	0	0
Montenegro *	4	100	3	75.0	0	0	0	0	3	75.0	1	25.0	0	0
Norway	229	100	26	11.4	17	7.4	0	0	43	18.8	186	81.2	0	0
Romania ||	199	100					66	33.2	66	33.2	133	66.8	0	0
Serbia ¤	18	100	10	55.6	0	0	6	33.3	16	88.9	2	11.1	0	0
Spain, Andalusia	566	100	19	3.4	22	3.9	0	0	41	7.2	525	92.8	0	0
Spain, Aragon	86	100	5	5.8	3	3.5	0	0	8	9.3	78	90.7	0	0
Spain, Asturias	68	100	3	4.4	1	1.5	0	0	4	5.9	64	94.1	0	0
Spain, Basque country	166	100	15	9.0	5	3.0	0	0	20	12.0	146	88.0	0	0
Spain, Canary Islands	116	100					2	1.7	2	1.7	114	98.3	0	0
Spain, Cantabria *	64	100	1	1.6	0	0	1	1.6	2	3.1	62	96.9	0	0
Spain, Castile and León *	139	100	0	0	2	1.4	6	4.3	8	5.8	131	94.2	0	0
Spain, Castile-La Mancha *	80	100	0	0	0	0	0	0	0	0	80	100	0	0
Spain, Catalonia	949	100	77	8.1	101	10.6	0	0	178	18.8	771	81.2	0	0
Spain, Community of Madrid	443	100					47	10.6	47	10.6	396	89.4	0	0
Spain, Extremadura	40	100	0	0	0	0	2	5.0	2	5.0	38	95.0	0	0
Spain, Galicia	151	100	3	2.0	0	0	18	11.9	21	13.9	130	86.1	0	0
Spain, La Rioja	12	100					2	16.7	2	16.7	10	83.3	0	0
Spain, Murcia	93	100	3	3.2	0	0	2	2.2	5	5.4	88	94.6	0	0
Spain, Navarre *	47	100					3	6.4	3	6.4	44	93.6	0	0
Spain, Valencian region	305	100	1	0.3	0	0	8	2.6	9	3.0	296	97.0	0	0
Sweden	459	100	59	12.9	39	8.5	0	0	98	21.4	361	78.6	0	0
Switzerland	343	100	52	15.2	59	17.2	0	0	111	32.4	231	67.3	1	0.3
the Netherlands &	959	100					494	51.5	494	51.5	465	48.5	0	0
United Kingdom, England ¶ #	2474	100	325	13.1	201	8.1	61	2.5	587	23.7	1872	75.7	15	0.6
United Kingdom, Northern Ireland #	106	100	29	27.4	21	19.8			54	50.9	52	49.1	0	0
United Kingdom, Scotland	243	100	37	15.2	42	17.3	9	3.7	88	36.2	149	61.3	6	2.5
United Kingdom, Wales #	110	100	21	19.1	20	18.2	0	0	41	37.3	69	62.7	0	0

Categories may not add up because of rounding; When cells are left empty, (complete) data are unavailable
§ Data based on residents and non-residents
** Patients younger than 20 years of age are not reported*
|| The transplant activity is underestimated by approximately 30%
¤ The transplant activity is underestimated by approximately 16% for deceased donor transplants and by approximately 12% for living donor transplants due to centres not submitting complete data
& The transplant activity is underestimated by approximately 2%
¶ The transplant activity is underestimated by approximately 7.1% due to patients opting out of data sharing for research purposes
# Values based on 1 to 5 patients are suppressed

Table B.5.2
Kidney transplants per million population by donor type, unadjusted

	All	Living donor				Deceased donor	Donor type Unkn
		Related	Unrelated	Type Unkn	All		
	Pmp	Pmp	Pmp	Pmp	Pmp	Pmp	Pmp
Austria §	35.1	2.8	2.6	0	5.5	29.7	0
Belgium, Dutch-speaking *	34.7	2.4	1.5	0	3.9	27.6	3.3
Belgium, French-speaking *	37.3	2.0	1.0	0	3.0	33.9	0.4
Bosnia and Herzegovina	4.2	2.8	0.3	0	3.1	0.8	0.3
Denmark	41.0	8.6	4.6	0	13.2	27.8	0
Estonia	25.9	3.0	0	0	3.0	23.0	0
France (17 of 18 regions)	49.6	4.4	3.2	0	7.6	41.9	0.1
Greece	18.6	5.8	2.0	0	7.9	10.7	0
Iceland	20.9	7.9	0	0	7.9	13.1	0
Montenegro *	6.5	4.9	0	0	4.9	1.6	0
Norway	42.0	4.8	3.1	0	7.9	34.1	0
Romania ||	10.4			3.5	3.5	7.0	0
Serbia ¤	2.8	1.6	0	0.9	2.5	0.3	0
Spain, Andalusia	66.3	2.2	2.6	0	4.8	61.5	0
Spain, Aragon	64.0	3.7	2.2	0	6.0	58.1	0
Spain, Asturias	67.6	3.0	1.0	0	4.0	63.6	0
Spain, Basque country	75.0	6.8	2.3	0	9.0	66.0	0
Spain, Canary Islands	52.8			0.9	0.9	51.8	0
Spain, Cantabria *	109.0	1.7	0	1.7	3.4	105.6	0
Spain, Castile and León *	58.6	0	0.8	2.5	3.4	55.2	0
Spain, Castile-La Mancha *	38.7	0	0	0	0	38.7	0
Spain, Catalonia	121.8	9.9	13.0	0	22.8	98.9	0
Spain, Community of Madrid	69.1			7.3	7.3	61.8	0
Spain, Extremadura	37.9	0	0	1.9	1.9	36.0	0
Spain, Galicia	56.0	1.1	0	6.7	7.8	48.2	0
Spain, La Rioja	37.4			6.2	6.2	31.2	0
Spain, Murcia	60.7	2.0	0	1.3	3.3	57.4	0
Spain, Navarre *	70.4			4.5	4.5	65.9	0
Spain, Valencian region	59.8	0.2	0	1.6	1.8	58.1	0
Sweden	43.8	5.6	3.7	0	9.3	34.4	0
Switzerland	39.5	6.0	6.8	0	12.8	26.6	0.1
the Netherlands	55.3			28.5	28.5	26.8	0
United Kingdom, England #	46.6	6.1	3.8	1.1	11.1	35.3	0.3
United Kingdom, Northern Ireland #	55.5	15.2	11.0		28.3	27.2	0
United Kingdom, Scotland	44.6	6.8	7.7	1.7	16.2	27.4	1.1
United Kingdom, Wales #	35.1	6.7	6.4	0	13.1	22.0	0

Categories may not add up because of rounding; When cells are left empty, (complete) data are unavailable
§ The number of transplants differs from table B.5.1 as non-residents are excluded for this analysis
** Patients younger than 20 years of age are not reported*
|| The transplant activity is underestimated by approximately 30%
¤ The transplant activity is underestimated by approximately 16% for deceased donor transplants and by approximately 12% for living donor transplants due to centres not submitting complete data
# Values based on 1 to 5 patients are suppressed

Table B.5.3

Sex, mean age, and median age of kidney transplant recipients

at time of transplantation

	All						Male						Female					
	%	Mean (years)	SD	Median (years)	P25	P75	%	Mean (years)	SD	Median (years)	P25	P75	%	Mean (years)	SD	Median (years)	P25	P75
Austria	100	51.8	15.1	54.0	43.7	62.6	65.5	52.5	15.6	55.5	43.8	63.6	34.5	50.6	14.1	53.2	41.9	59.8
Belgium, Dutch-speaking *	100	55.2	13.6	58.7	45.2	65.7	65.4	55.5	13.4	58.6	45.1	66.3	34.6	54.6	14.1	59.4	45.7	64.9
Belgium, French-speaking *	100	54.2	13.2	56.8	46.7	64.0	59.2	55.6	13.0	57.3	48.6	65.1	40.8	52.3	13.5	53.9	43.6	63.1
Bosnia and Herzegovina	100	42.4	11.4	40.6	34.4	52.0	73.3	40.6	11.4	39.5	34.4	50.5	26.7	47.1	11.3	48.5	38.2	56.0
Denmark	100	49.7	14.5	52.4	41.3	60.1	59.5	49.6	14.5	52.4	39.9	60.3	40.5	49.8	14.5	52.3	43.2	60.0
Estonia	100	46.5	18.6	49.3	36.3	59.9	48.6	51.7	17.5	54.6	48.2	61.2	51.4	41.6	18.7	47.5	30.6	49.5
France (17 of 18 regions)	100	53.1	16.7	54.9	42.1	66.2	63.5	53.6	16.9	55.5	42.5	67.1	36.5	52.2	16.4	53.8	41.4	64.7
Greece	100	47.7	13.2	49.5	39.2	57.3	62.4	48.2	12.8	48.4	40.5	57.3	37.6	46.9	13.9	50.5	38.6	56.6
Iceland	100	38.3	18.5	41.6	23.1	52.7	75.0	38.3	19.0	41.6	25.3	50.0	25.0	38.2	24.4	38.2	21.0	55.5
Montenegro *	100	39.4	14.6	37.9	28.0	50.8	25.0	24.3	0	24.3	24.3	24.3	75.0	44.4	12.9	44.2	31.6	57.5
Norway	100	52.9	16.3	54.2	42.5	66.2	56.3	51.7	17.1	52.6	39.3	66.7	43.7	54.5	15.2	56.1	46.0	66.2
Romania	100	42.2	13.3	43.6	33.4	53.0	68.8	42.7	12.8	44.1	34.4	52.6	31.2	40.9	14.4	43.0	29.9	53.3
Serbia	100	34.6	11.0	36.5	27.5	42.4	77.8	35.4	10.8	36.5	27.5	42.4	22.2	31.9	12.9	32.8	21.9	41.9
Spain, Andalusia	100	59.4	15.8	62.5	50.0	70.8	63.3	59.8	15.5	62.4	51.6	70.5	36.7	58.9	16.3	62.9	47.4	70.9
Spain, Aragon	100	58.1	15.1	61.6	48.6	69.9	62.8	57.5	15.8	61.9	46.7	69.1	37.2	59.1	14.0	61.3	54.6	70.3
Spain, Asturias	100	58.3	13.9	60.6	49.7	67.7	67.6	58.5	14.1	60.7	49.9	67.6	32.4	57.9	13.8	57.7	49.5	69.4
Spain, Basque country	100	59.2	15.9	62.7	49.4	72.0	64.5	61.4	13.8	63.8	52.3	72.8	35.5	55.3	18.6	59.8	43.9	69.5
Spain, Canary Islands	100	57.8	12.1	59.8	51.0	66.9	71.6	58.2	12.4	60.7	50.6	67.8	28.4	56.8	11.4	59.4	51.5	64.7
Spain, Cantabria *	100	58.1	13.4	61.3	50.1	68.7	68.8	60.1	11.9	61.7	54.1	69.6	31.3	53.9	15.6	56.2	41.8	67.2
Spain, Castile and León *	100	60.3	12.4	62.4	52.6	68.6	68.3	61.0	12.3	63.2	54.4	69.3	31.7	58.9	12.7	60.1	52.6	67.4
Spain, Castile-La Mancha *	100	58.2	13.3	57.8	48.9	69.1	60.0	57.9	14.0	57.4	49.2	70.8	40.0	58.8	12.4	58.6	48.6	68.5
Spain, Catalonia	100	57.9	15.9	60.0	48.1	70.3	62.5	58.5	15.7	61.1	48.2	70.8	37.5	56.7	16.2	58.9	47.8	69.0
Spain, Community of Madrid	100	55.6	16.8	58.0	47.8	67.2	61.2	54.6	17.2	58.0	47.4	66.2	38.8	57.0	16.2	58.0	49.4	69.0
Spain, Extremadura	100	57.5	12.0	60.1	50.2	64.7	52.5	58.7	10.7	62.3	51.4	64.3	47.5	56.2	13.4	59.7	47.7	65.1
Spain, Galicia	100	58.7	11.8	60.3	50.6	67.4	61.6	58.7	12.3	60.3	51.1	67.4	38.4	58.6	11.0	60.0	49.6	67.4
Spain, La Rioja	100	59.8	8.3	60.8	58.2	64.0	83.3	58.5	8.1	60.8	56.2	61.5	16.7	66.5	8.2	66.5	60.7	72.3
Spain, Murcia	100	52.4	14.0	55.4	45.4	62.3	75.3	52.0	14.5	55.0	44.9	62.5	24.7	53.6	12.5	57.1	48.3	61.6
Spain, Navarre *	100	57.3	14.7	61.1	45.3	68.0	72.3	57.5	14.8	61.9	45.3	70.2	27.7	56.6	15.1	60.8	45.7	65.1
Spain, Valencian region	100	54.0	15.6	56.8	45.3	64.7	62.3	53.6	16.1	56.8	43.3	64.9	37.7	54.7	14.8	56.6	46.1	64.6
Sweden	100	52.2	16.3	55.3	43.2	65.3	63.2	52.0	16.2	54.3	41.9	65.1	36.8	52.5	16.4	56.4	46.0	65.5
Switzerland	100	53.2	15.9	56.5	42.5	64.7	68.5	54.0	16.1	57.5	44.4	65.6	31.5	51.3	15.2	54.1	42.0	63.2
the Netherlands	100	53.9	15.7	56.9	44.0	66.2	64.1	54.9	15.5	58.0	44.4	67.3	35.9	52.1	15.8	54.7	42.8	64.4
United Kingdom, England	100	49.9	16.2	52.5	38.6	62.8	62.5	50.4	16.2	53.2	39.2	63.3	37.5	49.1	16.3	51.9	37.7	61.5
United Kingdom, Northern Ireland	100	51.9	15.6	52.3	41.1	65.2	67.0	53.7	14.5	52.7	42.4	67.8	33.0	48.3	17.1	49.4	37.3	59.3
United Kingdom, Scotland	100	49.9	15.6	52.2	40.7	61.1	56.0	48.8	15.9	51.9	37.9	59.8	43.2	51.4	15.1	53.0	43.2	62.3
United Kingdom, Wales	100	49.0	14.5	51.8	37.5	61.3	61.8	49.5	14.5	51.3	37.7	61.4	38.2	48.2	14.7	51.9	34.8	61.3

Categories may not add up because of missing values or rounding
** Patients younger than 20 years of age are not reported*

Table B.6.1

Incident KRT patients, from day 1, unadjusted

by age, sex, and primary renal disease

	Survival probabilities as % (95% CI)						
	cohort 2013 - 2017				cohort 2016 - 2020		
	90 day	1 year	2 year	5 year	90 day	1 year	2 year
0-19	99.5 (99.1-99.7)	98.1 (97.4-98.5)	97.0 (96.3-97.6)	94.6 (93.6-95.4)	99.3 (98.9-99.6)	97.7 (97.0-98.3)	97.0 (96.2-97.6)
20-44	99.2 (99.1-99.3)	97.2 (96.9-97.4)	95.0 (94.7-95.3)	89.4 (88.9-89.8)	99.2 (99.1-99.3)	97.0 (96.8-97.2)	94.5 (94.2-94.8)
45-64	97.5 (97.3-97.6)	91.8 (91.6-92.1)	85.8 (85.5-86.1)	69.3 (68.9-69.7)	97.6 (97.5-97.8)	92.3 (92.1-92.5)	85.9 (85.6-86.2)
65-74	95.0 (94.8-95.2)	84.7 (84.3-85.0)	73.9 (73.5-74.3)	46.2 (45.7-46.7)	95.2 (95.0-95.4)	85.0 (84.7-85.3)	74.3 (73.9-74.7)
75+	91.3 (91.1-91.5)	75.4 (75.0-75.7)	59.5 (59.1-60.0)	25.3 (24.9-25.6)	91.6 (91.4-91.8)	76.2 (75.9-76.6)	60.6 (60.2-61.0)
Male	95.1 (94.9-95.2)	85.1 (84.9-85.3)	74.9 (74.6-75.1)	50.3 (50.0-50.6)	95.2 (95.1-95.3)	85.4 (85.2-85.6)	75.0 (74.8-75.3)
Female	95.0 (94.8-95.2)	85.6 (85.3-85.9)	76.2 (75.9-76.5)	53.6 (53.2-54.0)	95.3 (95.1-95.4)	86.3 (86.0-86.6)	76.8 (76.5-77.1)
Diabetes	96.0 (95.8-96.2)	86.3 (86.0-86.7)	74.8 (74.4-75.2)	44.8 (44.3-45.2)	96.1 (95.9-96.3)	86.5 (86.2-86.8)	74.7 (74.3-75.1)
Hypertension / renal vascular disease	94.7 (94.5-94.9)	83.9 (83.5-84.3)	72.1 (71.6-72.6)	43.3 (42.8-43.9)	95.2 (94.9-95.4)	84.9 (84.5-85.3)	73.1 (72.6-73.6)
Glomerulonephritis	97.7 (97.5-97.9)	92.9 (92.6-93.3)	87.8 (87.3-88.2)	72.6 (71.9-73.2)	98.0 (97.8-98.2)	93.5 (93.2-93.9)	88.2 (87.8-88.7)
Other causes	94.3 (94.2-94.5)	84.0 (83.7-84.3)	74.4 (74.1-74.7)	53.6 (53.2-53.9)	94.5 (94.3-94.6)	84.3 (84.1-84.6)	74.7 (74.4-75.0)
All	95.0 (94.9-95.1)	85.3 (85.1-85.5)	75.3 (75.1-75.5)	51.5 (51.2-51.7)	95.2 (95.1-95.3)	85.7 (85.6-85.9)	75.7 (75.5-75.9)

** Based on data from Austria, Belgium (Dutch-speaking), Belgium (French-speaking), Bosnia and Herzegovina, Denmark, Estonia, France, Greece, Iceland, Norway, Spain (Andalusia), Spain (Aragon), Spain (Asturias), Spain (Basque country), Spain (Canary Islands), Spain (Cantabria), Spain (Castile and León), Spain (Castile-La Mancha), Spain (Catalonia), Spain (Community of Madrid), Spain (Extremadura), Spain (Galicia), Spain (Murcia), Spain (Navarre), Spain (Valencian Region), Sweden, the Netherlands, United Kingdom (England/Northern Ireland/Wales) and United Kingdom (Scotland)*

Table B.6.2

Incident KRT patients, from day 1, adjusted

adjusted for age, sex, and primary renal disease

	Survival probabilities as % (95% CI)						
	cohort 2013 - 2017				cohort 2016 - 2020		
	**90 day**	**1 year**	**2 year**	**5 year**	**90 day**	**1 year**	**2 year**
0-19	99.5 (99.2-99.8)	98.0 (97.4-98.6)	97.0 (96.3-97.7)	94.3 (93.4-95.3)	99.3 (98.9-99.6)	97.6 (97.0-98.2)	96.8 (96.1-97.6)
20-44	99.2 (99.1-99.4)	97.2 (96.9-97.4)	94.9 (94.6-95.3)	89.0 (88.5-89.5)	99.2 (99.1-99.3)	97.0 (96.7-97.2)	94.4 (94.1-94.7)
45-64	97.6 (97.5-97.8)	92.2 (91.9-92.4)	86.2 (86.0-86.5)	69.7 (69.3-70.1)	97.8 (97.7-98.0)	92.7 (92.4-92.9)	86.3 (86.0-86.6)
65-74	95.4 (95.2-95.6)	85.5 (85.2-85.8)	75.1 (74.7-75.5)	47.6 (47.1-48.0)	95.5 (95.4-95.7)	85.8 (85.5-86.1)	75.3 (74.9-75.7)
75+	91.7 (91.4-91.9)	76.2 (75.8-76.5)	60.5 (60.1-60.9)	26.1 (25.7-26.5)	92.1 (91.9-92.3)	77.1 (76.8-77.5)	61.7 (61.3-62.1)
Male	96.2 (96.1-96.3)	88.0 (87.8-88.2)	78.8 (78.5-79.0)	52.8 (52.4-53.1)	96.3 (96.2-96.4)	88.1 (87.9-88.2)	78.6 (78.4-78.9)
Female	96.2 (96.0-96.3)	88.5 (88.2-88.7)	80.1 (79.8-80.4)	56.6 (56.1-57.0)	96.3 (96.2-96.5)	88.8 (88.5-89.0)	80.2 (79.9-80.5)
Diabetes	96.6 (96.5-96.8)	88.2 (87.9-88.5)	77.6 (77.2-78.0)	46.7 (46.2-47.2)	96.7 (96.5-96.9)	88.3 (88.0-88.6)	77.5 (77.1-77.9)
Hypertension / renal vascular disease	96.8 (96.6-96.9)	89.8 (89.5-90.0)	81.4 (81.0-81.8)	56.1 (55.6-56.6)	96.9 (96.7-97.0)	89.9 (89.6-90.2)	81.2 (80.8-81.6)
Glomerulonephritis	97.4 (97.2-97.6)	91.8 (91.4-92.2)	85.5 (85.0-86.0)	65.5 (64.7-66.2)	97.7 (97.5-97.9)	92.5 (92.1-92.9)	86.0 (85.5-86.6)
Other causes	95.3 (95.1-95.4)	86.2 (85.9-86.4)	77.1 (76.8-77.4)	54.1 (53.7-54.5)	95.4 (95.2-95.5)	86.3 (86.1-86.5)	77.2 (76.9-77.4)
All	96.2 (96.1-96.3)	88.2 (88.0-88.3)	79.3 (79.1-79.5)	54.2 (53.9-54.4)	96.3 (96.2-96.4)	88.3 (88.2-88.5)	79.2 (79.0-79.4)

** Based on data from Austria, Belgium (Dutch-speaking), Belgium (French-speaking), Bosnia and Herzegovina, Denmark, Estonia, France, Greece, Iceland, Norway, Spain (Andalusia), Spain (Aragon), Spain (Asturias), Spain (Basque country), Spain (Canary Islands), Spain (Cantabria), Spain (Castile and León), Spain (Castile-La Mancha), Spain (Catalonia), Spain (Community of Madrid), Spain (Extremadura), Spain (Galicia), Spain (Murcia), Spain (Navarre), Spain (Valencian Region), Sweden, the Netherlands, United Kingdom (England/Northern Ireland/Wales) and United Kingdom (Scotland)*

Table B.6.3

Incident KRT patients, from day 91, unadjusted

by age, sex, and primary renal disease

	Survival probabilities as % (95% CI)				
	cohort 2013 - 2017			cohort 2016 - 2020	
	1 year	2 year	5 year	1 year	2 year
0-19	98.3 (97.7-98.8)	97.4 (96.6-98.0)	94.9 (94.0-95.8)	98.2 (97.5-98.6)	97.5 (96.8-98.1)
20-44	97.3 (97.1-97.5)	95.2 (94.9-95.5)	89.6 (89.2-90.1)	97.2 (96.9-97.4)	94.7 (94.4-95.0)
45-64	92.8 (92.5-93.0)	86.6 (86.3-86.9)	69.9 (69.5-70.3)	92.9 (92.7-93.2)	86.5 (86.2-86.8)
65-74	86.4 (86.0-86.7)	75.2 (74.8-75.6)	46.7 (46.2-47.2)	86.7 (86.4-87.0)	75.5 (75.1-75.9)
75+	78.4 (78.1-78.8)	61.5 (61.1-62.0)	25.7 (25.4-26.1)	79.2 (78.8-79.5)	62.4 (62.0-62.8)
Male	86.9 (86.7-87.1)	76.3 (76.1-76.6)	51.4 (51.1-51.7)	87.2 (87.0-87.4)	76.4 (76.1-76.6)
Female	87.8 (87.5-88.0)	78.1 (77.8-78.4)	55.1 (54.6-55.5)	88.2 (88.0-88.5)	78.5 (78.2-78.8)
Diabetes	87.0 (86.6-87.3)	75.1 (74.7-75.6)	44.7 (44.2-45.2)	87.2 (86.9-87.5)	74.8 (74.4-75.2)
Hypertension / renal vascular disease	85.6 (85.2-86.0)	73.5 (73.0-74.0)	44.0 (43.4-44.5)	86.3 (85.9-86.7)	74.0 (73.5-74.5)
Glomerulonephritis	93.9 (93.5-94.2)	88.5 (88.1-89.0)	73.1 (72.5-73.7)	94.1 (93.7-94.4)	88.8 (88.4-89.3)
Other causes	86.6 (86.3-86.8)	76.8 (76.4-77.1)	55.6 (55.2-55.9)	86.8 (86.6-87.0)	76.9 (76.6-77.2)
All	87.2 (87.1-87.4)	77.0 (76.8-77.2)	52.7 (52.4-52.9)	87.5 (87.4-87.7)	77.1 (76.9-77.3)

** Based on data from Austria, Belgium (Dutch-speaking), Belgium (French-speaking), Bosnia and Herzegovina, Denmark, Estonia, France, Greece, Iceland, Norway, Spain (Andalusia), Spain (Aragon), Spain (Asturias), Spain (Basque country), Spain (Canary Islands), Spain (Cantabria), Spain (Castile and León), Spain (Castile-La Mancha), Spain (Catalonia), Spain (Community of Madrid), Spain (Extremadura), Spain (Galicia), Spain (Murcia), Spain (Navarre), Spain (Valencian Region), Sweden, the Netherlands, United Kingdom (England/Northern Ireland/Wales) and United Kingdom (Scotland)*

Table B.6.4

Incident KRT patients, from day 91, adjusted

adjusted for age, sex, and primary renal disease

	Survival probabilities as % (95% CI)				
	cohort 2013 - 2017			cohort 2016 - 2020	
	1 year	2 year	5 year	1 year	2 year
0-19	98.3 (97.8-98.9)	97.3 (96.6-98.0)	94.6 (93.7-95.6)	98.1 (97.5-98.7)	97.4 (96.7-98.0)
20-44	97.2 (97.0-97.5)	95.1 (94.8-95.4)	89.2 (88.7-89.7)	97.1 (96.8-97.3)	94.6 (94.2-94.9)
45-64	93.0 (92.8-93.2)	87.0 (86.7-87.3)	70.3 (69.8-70.7)	93.1 (92.9-93.4)	86.8 (86.5-87.1)
65-74	87.0 (86.7-87.4)	76.3 (75.9-76.7)	48.1 (47.6-48.6)	87.3 (87.0-87.6)	76.4 (76.0-76.8)
75+	79.1 (78.7-79.4)	62.5 (62.0-62.9)	26.6 (26.2-27.0)	79.9 (79.5-80.2)	63.4 (63.0-63.8)
Male	89.2 (89.1-89.4)	79.7 (79.4-79.9)	53.1 (52.8-53.4)	89.2 (89.1-89.4)	79.4 (79.2-79.7)
Female	90.0 (89.8-90.2)	81.4 (81.0-81.7)	57.2 (56.8-57.6)	90.1 (89.8-90.3)	81.2 (80.9-81.5)
Diabetes	88.6 (88.3-88.9)	77.7 (77.3-78.1)	46.2 (45.7-46.7)	88.8 (88.5-89.1)	77.3 (77.0-77.7)
Hypertension / renal vascular disease	90.8 (90.5-91.1)	82.2 (81.8-82.6)	56.2 (55.6-56.7)	90.7 (90.5-91.0)	81.7 (81.3-82.0)
Glomerulonephritis	92.7 (92.3-93.1)	86.2 (85.7-86.7)	65.6 (64.9-66.4)	93.0 (92.6-93.4)	86.6 (86.1-87.1)
Other causes	88.2 (88.0-88.4)	78.8 (78.5-79.1)	55.3 (54.9-55.6)	88.3 (88.0-88.5)	78.8 (78.5-79.1)
All	89.5 (89.4-89.7)	80.3 (80.1-80.5)	54.6 (54.4-54.9)	89.6 (89.4-89.7)	80.1 (79.9-80.3)

** Based on data from Austria, Belgium (Dutch-speaking), Belgium (French-speaking), Bosnia and Herzegovina, Denmark, Estonia, France, Greece, Iceland, Norway, Spain (Andalusia), Spain (Aragon), Spain (Asturias), Spain (Basque country), Spain (Canary Islands), Spain (Cantabria), Spain (Castile and León), Spain (Castile-La Mancha), Spain (Catalonia), Spain (Community of Madrid), Spain (Extremadura), Spain (Galicia), Spain (Murcia), Spain (Navarre), Spain (Valencian Region), Sweden, the Netherlands, United Kingdom (England/Northern Ireland/Wales) and United Kingdom (Scotland)*

Table B.6.5

Incident dialysis patients, from day 1, unadjusted

by age, sex, and primary renal disease

	Survival probabilities as % (95% CI)						
	cohort 2013 - 2017				cohort 2016 - 2020		
	**90 day**	**1 year**	**2 year**	**5 year**	**90 day**	**1 year**	**2 year**
0-19	99.4 (98.9-99.7)	97.3 (96.4-98.0)	95.0 (93.5-96.2)	85.8 (81.7-89.0)	99.4 (98.9-99.6)	97.0 (96.0-97.7)	95.9 (94.7-96.9)
20-44	99.1 (98.9-99.2)	96.6 (96.3-96.9)	93.1 (92.6-93.5)	79.7 (78.7-80.7)	99.1 (99.0-99.3)	96.4 (96.0-96.7)	92.4 (91.9-92.9)
45-64	97.3 (97.2-97.4)	91.0 (90.7-91.2)	83.5 (83.2-83.9)	58.9 (58.3-59.4)	97.5 (97.3-97.6)	91.5 (91.3-91.8)	83.7 (83.4-84.1)
65-74	94.9 (94.7-95.1)	84.1 (83.8-84.4)	72.5 (72.1-72.9)	41.0 (40.5-41.5)	95.0 (94.8-95.2)	84.5 (84.1-84.8)	72.9 (72.5-73.3)
75+	91.3 (91.0-91.5)	75.3 (74.9-75.6)	59.3 (58.9-59.7)	24.6 (24.2-25.0)	91.6 (91.3-91.8)	76.1 (75.7-76.4)	60.3 (59.9-60.7)
Male	94.8 (94.7-94.9)	84.0 (83.8-84.2)	72.1 (71.8-72.4)	39.9 (39.5-40.2)	95.0 (94.8-95.1)	84.4 (84.2-84.6)	72.5 (72.2-72.8)
Female	94.7 (94.5-94.9)	84.5 (84.2-84.8)	73.5 (73.1-73.9)	43.7 (43.3-44.2)	95.0 (94.8-95.2)	85.2 (84.9-85.5)	74.3 (73.9-74.6)
Diabetes	95.9 (95.7-96.1)	85.9 (85.6-86.3)	73.6 (73.1-74.0)	38.7 (38.2-39.2)	96.0 (95.9-96.2)	86.2 (85.8-86.5)	73.6 (73.2-74.0)
Hypertension / renal vascular disease	94.6 (94.4-94.9)	83.4 (83.0-83.8)	70.8 (70.3-71.3)	37.6 (37.1-38.2)	95.1 (94.9-95.3)	84.5 (84.0-84.9)	71.9 (71.3-72.4)
Glomerulonephritis	97.5 (97.3-97.7)	91.9 (91.5-92.3)	85.1 (84.5-85.6)	59.2 (58.2-60.1)	97.8 (97.6-98.0)	92.6 (92.2-93.0)	85.6 (85.0-86.1)
Other causes	93.9 (93.7-94.1)	82.3 (82.1-82.6)	70.7 (70.4-71.1)	41.7 (41.3-42.1)	94.1 (93.9-94.2)	82.8 (82.5-83.0)	71.3 (70.9-71.6)
All	94.8 (94.7-94.9)	84.2 (84.0-84.4)	72.6 (72.4-72.8)	41.3 (41.0-41.5)	95.0 (94.9-95.1)	84.7 (84.5-84.9)	73.1 (72.9-73.3)

** Based on data from Austria, Belgium (Dutch-speaking), Belgium (French-speaking), Bosnia and Herzegovina, Denmark, Estonia, France, Greece, Iceland, Norway, Spain (Andalusia), Spain (Aragon), Spain (Asturias), Spain (Basque country), Spain (Canary Islands), Spain (Cantabria), Spain (Castile and León), Spain (Castile-La Mancha), Spain (Catalonia), Spain (Community of Madrid), Spain (Extremadura), Spain (Galicia), Spain (Murcia), Spain (Navarre), Spain (Valencian Region), Sweden, the Netherlands, United Kingdom (England/Northern Ireland/Wales) and United Kingdom (Scotland)*

Table B.6.6

Incident dialysis patients, from day 1, adjusted

adjusted for age, sex, and primary renal disease

	Survival probabilities as % (95% CI)						
	cohort 2013 - 2017				cohort 2016 - 2020		
	90 day	1 year	2 year	5 year	90 day	1 year	2 year
0-19	99.4 (99.1-99.8)	97.3 (96.5-98.2)	95.0 (93.7-96.4)	85.5 (81.9-89.3)	99.3 (98.9-99.7)	96.8 (95.9-97.8)	95.7 (94.6-96.9)
20-44	99.1 (99.0-99.3)	96.6 (96.3-96.9)	93.2 (92.7-93.6)	79.6 (78.6-80.6)	99.2 (99.0-99.3)	96.4 (96.1-96.7)	92.4 (91.9-92.9)
45-64	97.5 (97.3-97.6)	91.3 (91.1-91.6)	84.0 (83.7-84.4)	59.6 (59.1-60.2)	97.7 (97.6-97.8)	91.9 (91.7-92.2)	84.2 (83.9-84.6)
65-74	95.2 (95.0-95.4)	84.8 (84.5-85.1)	73.5 (73.1-73.9)	42.2 (41.7-42.7)	95.4 (95.2-95.6)	85.2 (84.8-85.5)	73.9 (73.5-74.3)
75+	91.6 (91.4-91.8)	75.9 (75.6-76.3)	60.0 (59.6-60.4)	25.1 (24.8-25.5)	92.0 (91.8-92.2)	76.9 (76.5-77.2)	61.2 (60.8-61.6)
Male	95.7 (95.6-95.8)	86.3 (86.1-86.5)	75.7 (75.4-76.0)	45.1 (44.7-45.5)	95.9 (95.8-96.0)	86.8 (86.6-87.0)	76.2 (76.0-76.5)
Female	95.6 (95.5-95.8)	86.8 (86.6-87.1)	77.1 (76.8-77.5)	49.4 (48.9-49.9)	95.9 (95.8-96.1)	87.5 (87.3-87.8)	77.9 (77.6-78.2)
Diabetes	96.3 (96.2-96.5)	87.2 (86.9-87.5)	75.6 (75.2-76.1)	41.5 (41.0-42.0)	96.5 (96.3-96.7)	87.5 (87.2-87.8)	76.0 (75.6-76.4)
Hypertension / renal vascular disease	96.2 (96.0-96.4)	88.0 (87.7-88.3)	78.3 (77.9-78.7)	49.1 (48.5-49.7)	96.5 (96.4-96.7)	88.7 (88.4-89.0)	78.9 (78.5-79.4)
Glomerulonephritis	97.2 (96.9-97.4)	90.9 (90.5-91.4)	83.6 (83.0-84.2)	57.1 (56.2-58.1)	97.5 (97.3-97.7)	91.7 (91.3-92.2)	84.2 (83.6-84.8)
Other causes	94.6 (94.5-94.8)	84.2 (84.0-84.5)	73.6 (73.3-74.0)	46.0 (45.6-46.4)	94.9 (94.7-95.0)	84.8 (84.6-85.1)	74.4 (74.1-74.7)
All	95.7 (95.6-95.8)	86.5 (86.4-86.7)	76.3 (76.0-76.5)	46.7 (46.4-47.0)	95.9 (95.8-96.0)	87.1 (87.0-87.3)	76.9 (76.7-77.1)

** Based on data from Austria, Belgium (Dutch-speaking), Belgium (French-speaking), Bosnia and Herzegovina, Denmark, Estonia, France, Greece, Iceland, Norway, Spain (Andalusia), Spain (Aragon), Spain (Asturias), Spain (Basque country), Spain (Canary Islands), Spain (Cantabria), Spain (Castile and León), Spain (Castile-La Mancha), Spain (Catalonia), Spain (Community of Madrid), Spain (Extremadura), Spain (Galicia), Spain (Murcia), Spain (Navarre), Spain (Valencian Region), Sweden, the Netherlands, United Kingdom (England/Northern Ireland/Wales) and United Kingdom (Scotland)*

Table B.6.7

Incident dialysis patients, from day 91, unadjusted

by age, sex, and primary renal disease

	Survival probabilities as % (95% CI)				
	cohort 2013 - 2017			cohort 2016 - 2020	
	**1 year**	**2 year**	**5 year**	**1 year**	**2 year**
0-19	97.5 (96.4-98.2)	95.5 (93.9-96.6)	85.6 (81.3-89.0)	97.2 (96.1-97.9)	96.3 (95.0-97.2)
20-44	96.5 (96.1-96.8)	92.9 (92.3-93.3)	78.8 (77.8-79.8)	96.3 (96.0-96.6)	92.3 (91.8-92.8)
45-64	91.8 (91.5-92.0)	83.9 (83.6-84.3)	58.6 (58.0-59.1)	92.0 (91.7-92.2)	84.0 (83.7-84.4)
65-74	85.7 (85.4-86.0)	73.6 (73.1-74.0)	41.0 (40.5-41.6)	86.1 (85.8-86.4)	74.0 (73.5-74.4)
75+	78.3 (77.9-78.7)	61.2 (60.8-61.7)	25.0 (24.6-25.4)	79.0 (78.7-79.4)	62.0 (61.6-62.4)
Male	85.7 (85.5-85.9)	73.1 (72.8-73.4)	39.9 (39.6-40.3)	86.0 (85.8-86.2)	73.4 (73.1-73.7)
Female	86.5 (86.2-86.8)	75.0 (74.6-75.4)	44.1 (43.7-44.6)	87.0 (86.7-87.3)	75.6 (75.2-76.0)
Diabetes	86.4 (86.1-86.8)	73.6 (73.1-74.0)	38.0 (37.5-38.5)	86.7 (86.4-87.1)	73.4 (73.0-73.9)
Hypertension / renal vascular disease	85.0 (84.6-85.4)	71.9 (71.4-72.5)	37.6 (37.0-38.2)	85.8 (85.3-86.2)	72.6 (72.1-73.1)
Glomerulonephritis	92.7 (92.3-93.1)	85.4 (84.8-85.9)	58.6 (57.6-59.6)	93.0 (92.6-93.3)	85.7 (85.2-86.3)
Other causes	84.8 (84.5-85.1)	72.5 (72.2-72.9)	42.5 (42.1-42.9)	85.1 (84.9-85.4)	73.1 (72.7-73.4)
All	86.0 (85.8-86.2)	73.8 (73.5-74.0)	41.4 (41.1-41.7)	86.4 (86.2-86.5)	74.2 (74.0-74.4)

** Based on data from Austria, Belgium (Dutch-speaking), Belgium (French-speaking), Bosnia and Herzegovina, Denmark, Estonia, France, Greece, Iceland, Norway, Spain (Andalusia), Spain (Aragon), Spain (Asturias), Spain (Basque country), Spain (Canary Islands), Spain (Cantabria), Spain (Castile and León), Spain (Castile-La Mancha), Spain (Catalonia), Spain (Community of Madrid), Spain (Extremadura), Spain (Galicia), Spain (Murcia), Spain (Navarre), Spain (Valencian Region), Sweden, the Netherlands, United Kingdom (England/Northern Ireland/Wales) and United Kingdom (Scotland)*

Table B.6.8
Incident dialysis patients, from day 91, adjusted
adjusted for age, sex, and primary renal disease

	Survival probabilities as % (95% CI)				
	cohort 2013 - 2017			cohort 2016 - 2020	
	**1 year**	**2 year**	**5 year**	**1 year**	**2 year**
0-19	97.5 (96.6-98.4)	95.4 (94.0-96.7)	85.1 (81.2-89.2)	97.0 (96.1-98.0)	96.0 (94.9-97.2)
20-44	96.4 (96.1-96.8)	92.9 (92.4-93.4)	78.6 (77.6-79.7)	96.3 (95.9-96.6)	92.2 (91.7-92.7)
45-64	92.0 (91.8-92.3)	84.4 (84.1-84.8)	59.3 (58.8-59.9)	92.2 (92.0-92.5)	84.5 (84.1-84.8)
65-74	86.3 (85.9-86.6)	74.5 (74.0-74.9)	42.3 (41.8-42.8)	86.6 (86.3-86.9)	74.8 (74.4-75.2)
75+	78.8 (78.4-79.1)	61.9 (61.4-62.3)	25.5 (25.1-25.9)	79.6 (79.2-79.9)	62.8 (62.4-63.2)
Male	87.6 (87.4-87.8)	76.4 (76.1-76.6)	44.9 (44.5-45.2)	88.0 (87.8-88.2)	76.8 (76.5-77.1)
Female	88.4 (88.2-88.7)	78.3 (77.9-78.6)	49.5 (49.0-50.0)	88.8 (88.6-89.1)	78.8 (78.4-79.1)
Diabetes	87.6 (87.2-87.9)	75.5 (75.1-75.9)	40.6 (40.0-41.1)	88.0 (87.7-88.3)	75.7 (75.2-76.1)
Hypertension / renal vascular disease	89.1 (88.8-89.4)	78.9 (78.5-79.4)	48.6 (48.0-49.3)	89.5 (89.2-89.8)	79.2 (78.8-79.7)
Glomerulonephritis	91.9 (91.4-92.3)	84.0 (83.4-84.6)	56.7 (55.7-57.7)	92.2 (91.7-92.6)	84.5 (83.9-85.1)
Other causes	86.3 (86.1-86.6)	75.1 (74.8-75.5)	46.5 (46.0-46.9)	86.8 (86.5-87.0)	75.8 (75.5-76.2)
All	87.9 (87.7-88.1)	77.1 (76.9-77.3)	46.6 (46.3-46.9)	88.3 (88.1-88.5)	77.5 (77.3-77.8)

** Based on data from Austria, Belgium (Dutch-speaking), Belgium (French-speaking), Bosnia and Herzegovina, Denmark, Estonia, France, Greece, Iceland, Norway, Spain (Andalusia), Spain (Aragon), Spain (Asturias), Spain (Basque country), Spain (Canary Islands), Spain (Cantabria), Spain (Castile and León), Spain (Castile-La Mancha), Spain (Catalonia), Spain (Community of Madrid), Spain (Extremadura), Spain (Galicia), Spain (Murcia), Spain (Navarre), Spain (Valencian Region), Sweden, the Netherlands, United Kingdom (England/Northern Ireland/Wales) and United Kingdom (Scotland)*

Table B.6.9

First transplant patients (deceased donor), from day of transplant, unadjusted

by age, sex, and primary renal disease

	Survival probabilities as % (95% CI)				
	cohort 2013 - 2017			cohort 2016 - 2020	
	1 year	2 year	5 year	1 year	2 year
0-19	99.0 (98.1-99.4)	98.5 (97.6-99.1)	97.4 (96.2-98.2)	98.7 (97.8-99.2)	98.4 (97.5-99.0)
20-44	98.8 (98.6-99.0)	98.2 (97.9-98.5)	95.9 (95.5-96.3)	98.9 (98.7-99.1)	98.2 (97.9-98.4)
45-64	96.9 (96.6-97.1)	95.1 (94.8-95.4)	87.5 (87.1-88.0)	96.9 (96.6-97.1)	94.8 (94.5-95.1)
65+	92.6 (92.1-93.1)	88.0 (87.3-88.6)	69.7 (68.8-70.5)	92.4 (91.9-92.8)	86.6 (85.9-87.2)
Male	96.3 (96.1-96.5)	93.8 (93.5-94.1)	84.2 (83.8-84.7)	96.0 (95.7-96.2)	92.8 (92.5-93.1)
Female	96.2 (95.9-96.5)	94.5 (94.1-94.9)	86.7 (86.1-87.2)	96.2 (95.9-96.5)	94.1 (93.7-94.5)
Diabetes	94.6 (94.0-95.1)	91.2 (90.5-91.8)	77.2 (76.2-78.2)	93.5 (92.9-94.0)	89.1 (88.4-89.8)
Hypertension / renal vascular disease	95.5 (94.9-96.0)	92.0 (91.2-92.7)	80.4 (79.2-81.5)	95.3 (94.7-95.9)	91.3 (90.5-92.1)
Glomerulonephritis	97.5 (97.2-97.8)	96.0 (95.5-96.4)	89.3 (88.6-90.0)	97.4 (97.1-97.7)	95.5 (95.0-95.9)
Other causes	96.6 (96.3-96.8)	94.8 (94.5-95.1)	87.2 (86.7-87.7)	96.7 (96.4-96.9)	94.4 (94.1-94.7)
All	96.3 (96.1-96.5)	94.1 (93.8-94.3)	85.1 (84.7-85.4)	96.1 (95.9-96.3)	93.3 (93.1-93.5)

** Based on data from Austria, Belgium (Dutch-speaking), Belgium (French-speaking), Bosnia and Herzegovina, Denmark, Estonia, France, Greece, Iceland, Norway, Spain (Andalusia), Spain (Aragon), Spain (Asturias), Spain (Basque country), Spain (Canary Islands), Spain (Cantabria), Spain (Castile and León), Spain (Castile-La Mancha), Spain (Catalonia), Spain (Community of Madrid), Spain (Extremadura), Spain (Galicia), Spain (Murcia), Spain (Navarre), Spain (Valencian Region), Sweden, the Netherlands, United Kingdom (England/Northern Ireland/Wales) and United Kingdom (Scotland)*

Table B.6.10

First transplant patients (deceased donor), from day of transplant, adjusted

adjusted for age, sex, and primary renal disease

	Survival probabilities as % (95% CI)				
	cohort 2013 - 2017			cohort 2016 - 2020	
	1 year	2 year	5 year	1 year	2 year
0-19	98.9 (98.2-99.5)	98.3 (97.5-99.2)	97.1 (96.0-98.2)	98.6 (97.8-99.3)	98.2 (97.4-99.1)
20-44	98.8 (98.6-99.1)	98.3 (98.0-98.5)	96.0 (95.6-96.4)	99.0 (98.7-99.2)	98.2 (98.0-98.5)
45-64	97.1 (96.8-97.3)	95.4 (95.1-95.7)	88.2 (87.8-88.7)	97.1 (96.8-97.3)	95.1 (94.9-95.4)
65+	93.3 (92.8-93.8)	89.0 (88.4-89.6)	71.8 (70.9-72.7)	93.1 (92.6-93.5)	87.8 (87.2-88.4)
Male	98.2 (98.1-98.3)	96.9 (96.8-97.1)	91.6 (91.3-91.9)	98.2 (98.0-98.3)	96.6 (96.5-96.8)
Female	98.0 (97.8-98.1)	97.0 (96.8-97.2)	92.3 (91.9-92.7)	98.1 (97.9-98.2)	96.9 (96.7-97.1)
Diabetes	97.2 (97.0-97.5)	95.5 (95.1-95.8)	87.4 (86.7-88.0)	96.9 (96.7-97.2)	94.8 (94.4-95.2)
Hypertension / renal vascular disease	98.2 (98.0-98.4)	96.7 (96.4-97.1)	91.4 (90.8-92.0)	98.2 (98.0-98.5)	96.6 (96.3-96.9)
Glomerulonephritis	98.5 (98.3-98.7)	97.5 (97.3-97.8)	93.2 (92.7-93.6)	98.5 (98.3-98.7)	97.3 (97.0-97.6)
Other causes	98.0 (97.9-98.2)	97.0 (96.8-97.2)	92.2 (91.9-92.6)	98.2 (98.0-98.3)	96.9 (96.7-97.1)
All	98.1 (98.0-98.2)	97.0 (96.8-97.1)	91.9 (91.6-92.2)	98.1 (98.0-98.3)	96.8 (96.6-96.9)

** Based on data from Austria, Belgium (Dutch-speaking), Belgium (French-speaking), Bosnia and Herzegovina, Denmark, Estonia, France, Greece, Iceland, Norway, Spain (Andalusia), Spain (Aragon), Spain (Asturias), Spain (Basque country), Spain (Canary Islands), Spain (Cantabria), Spain (Castile and León), Spain (Castile-La Mancha), Spain (Catalonia), Spain (Community of Madrid), Spain (Extremadura), Spain (Galicia), Spain (Murcia), Spain (Navarre), Spain (Valencian Region), Sweden, the Netherlands, United Kingdom (England/Northern Ireland/Wales) and United Kingdom (Scotland)*

Table B.6.11

First transplant patients (living donor), from day of transplant, unadjusted

by age, sex, and primary renal disease

	Survival probabilities as % (95% CI)				
	cohort 2013 - 2017			cohort 2016 - 2020	
	1 year	2 year	5 year	1 year	2 year
0-19	99.2 (98.4-99.6)	99.0 (98.1-99.5)	98.2 (97.1-98.9)	99.2 (98.3-99.6)	98.6 (97.6-99.2)
20-44	99.6 (99.3-99.7)	99.3 (99.0-99.5)	98.3 (97.9-98.7)	99.7 (99.5-99.8)	99.3 (99.0-99.6)
45-64	98.7 (98.4-99.0)	97.9 (97.4-98.2)	93.8 (93.2-94.4)	98.6 (98.3-98.9)	97.6 (97.1-98.0)
65+	96.7 (95.7-97.5)	94.1 (92.8-95.2)	81.7 (79.7-83.5)	97.2 (96.3-97.9)	94.9 (93.7-95.9)
Male	98.6 (98.4-98.9)	97.8 (97.5-98.1)	94.0 (93.5-94.5)	98.6 (98.3-98.8)	97.7 (97.3-98.0)
Female	99.1 (98.8-99.4)	98.3 (97.9-98.6)	94.6 (93.9-95.2)	99.3 (99.0-99.5)	98.3 (97.8-98.6)
Diabetes	97.6 (96.4-98.4)	95.8 (94.4-96.9)	85.8 (83.4-87.8)	97.7 (96.4-98.5)	95.1 (93.5-96.3)
Hypertension / renal vascular disease	98.1 (97.0-98.8)	96.1 (94.8-97.2)	89.1 (87.0-90.8)	97.3 (96.0-98.2)	95.7 (94.2-96.8)
Glomerulonephritis	98.8 (98.4-99.2)	98.3 (97.8-98.7)	95.9 (95.2-96.6)	99.1 (98.7-99.4)	98.6 (98.1-99.0)
Other causes	99.1 (98.9-99.3)	98.5 (98.2-98.8)	95.5 (95.0-96.0)	99.1 (98.8-99.3)	98.3 (98.0-98.6)
All	98.8 (98.6-99.0)	98.0 (97.7-98.2)	94.2 (93.8-94.6)	98.8 (98.6-99.0)	97.9 (97.6-98.1)

** Based on data from Austria, Belgium (Dutch-speaking), Belgium (French-speaking), Bosnia and Herzegovina, Denmark, Estonia, France, Greece, Iceland, Norway, Spain (Andalusia), Spain (Aragon), Spain (Asturias), Spain (Basque country), Spain (Canary Islands), Spain (Cantabria), Spain (Castile and León), Spain (Castile-La Mancha), Spain (Catalonia), Spain (Community of Madrid), Spain (Extremadura), Spain (Galicia), Spain (Murcia), Spain (Navarre), Spain (Valencian Region), Sweden, the Netherlands, United Kingdom (England/Northern Ireland/Wales) and United Kingdom (Scotland)*

Table B.6.12

First transplant patients (living donor), from day of transplant, adjusted

adjusted for age, sex, and primary renal disease

	Survival probabilities as % (95% CI)				
	cohort 2013 - 2017			cohort 2016 - 2020	
	1 year	2 year	5 year	1 year	2 year
0-19	99.2 (98.5-99.8)	98.9 (98.2-99.7)	98.0 (96.9-99.0)	99.2 (98.5-99.8)	98.5 (97.6-99.4)
20-44	99.5 (99.3-99.7)	99.3 (99.0-99.6)	98.2 (97.8-98.6)	99.7 (99.5-99.9)	99.3 (99.0-99.6)
45-64	98.8 (98.5-99.1)	98.0 (97.6-98.4)	94.1 (93.4-94.7)	98.6 (98.3-99.0)	97.7 (97.3-98.1)
65+	97.0 (96.2-97.8)	94.6 (93.6-95.7)	83.2 (81.4-85.1)	97.5 (96.8-98.2)	95.4 (94.5-96.4)
Male	99.0 (98.8-99.2)	98.4 (98.2-98.7)	95.5 (95.0-95.9)	99.0 (98.8-99.2)	98.3 (98.0-98.6)
Female	99.2 (99.0-99.5)	98.5 (98.2-98.8)	95.1 (94.5-95.8)	99.4 (99.2-99.6)	98.6 (98.2-98.9)
Diabetes	98.6 (98.0-99.1)	97.5 (96.7-98.3)	90.9 (89.4-92.5)	98.7 (98.1-99.2)	97.2 (96.3-98.0)
Hypertension / renal vascular disease	99.0 (98.5-99.4)	97.9 (97.2-98.6)	93.8 (92.6-95.0)	98.6 (98.1-99.2)	97.8 (97.1-98.5)
Glomerulonephritis	98.8 (98.5-99.2)	98.3 (97.9-98.8)	95.8 (95.1-96.5)	99.1 (98.8-99.5)	98.6 (98.2-99.1)
Other causes	99.2 (99.1-99.4)	98.7 (98.4-98.9)	96.0 (95.6-96.5)	99.2 (99.0-99.4)	98.5 (98.3-98.8)
All	99.1 (98.9-99.2)	98.4 (98.2-98.7)	95.3 (95.0-95.7)	99.1 (99.0-99.3)	98.4 (98.2-98.6)

** Based on data from Austria, Belgium (Dutch-speaking), Belgium (French-speaking), Bosnia and Herzegovina, Denmark, Estonia, France, Greece, Iceland, Norway, Spain (Andalusia), Spain (Aragon), Spain (Asturias), Spain (Basque country), Spain (Canary Islands), Spain (Cantabria), Spain (Castile and León), Spain (Castile-La Mancha), Spain (Catalonia), Spain (Community of Madrid), Spain (Extremadura), Spain (Galicia), Spain (Murcia), Spain (Navarre), Spain (Valencian Region), Sweden, the Netherlands, United Kingdom (England/Northern Ireland/Wales) and United Kingdom (Scotland)*

Table B.6.13

First graft (deceased donor), from day of transplant, unadjusted

by age, sex, and primary renal disease

	Survival probabilities as % (95% CI)				
	cohort 2013 - 2017			cohort 2016 - 2020	
	1 year	2 year	5 year	1 year	2 year
0-19	94.8 (93.3-95.9)	92.5 (90.8-93.9)	85.8 (83.5-87.7)	94.8 (93.3-95.9)	92.5 (90.8-94.0)
20-44	94.3 (93.8-94.8)	92.5 (91.9-93.0)	85.5 (84.7-86.2)	95.1 (94.7-95.6)	93.1 (92.6-93.6)
45-64	92.1 (91.7-92.4)	89.2 (88.8-89.6)	79.1 (78.5-79.7)	92.2 (91.8-92.5)	89.1 (88.7-89.6)
65+	85.8 (85.1-86.5)	80.4 (79.6-81.1)	61.3 (60.4-62.3)	85.8 (85.1-86.4)	79.4 (78.6-80.1)
Male	91.0 (90.7-91.4)	87.5 (87.1-87.9)	75.4 (74.8-75.9)	90.8 (90.5-91.2)	86.8 (86.4-87.2)
Female	91.1 (90.6-91.5)	88.3 (87.7-88.8)	77.6 (76.9-78.3)	91.5 (91.1-92.0)	88.2 (87.7-88.7)
Diabetes	89.3 (88.6-90.1)	85.0 (84.1-85.9)	69.4 (68.3-70.5)	88.4 (87.7-89.1)	83.3 (82.4-84.1)
Hypertension / renal vascular disease	89.1 (88.2-89.9)	84.6 (83.5-85.6)	70.1 (68.7-71.3)	89.7 (88.8-90.5)	84.7 (83.7-85.6)
Glomerulonephritis	92.0 (91.4-92.5)	89.2 (88.5-89.8)	78.9 (78.0-79.8)	92.2 (91.6-92.7)	89.1 (88.4-89.7)
Other causes	91.7 (91.3-92.1)	88.9 (88.5-89.3)	78.8 (78.2-79.3)	91.9 (91.5-92.3)	88.7 (88.3-89.2)
All	91.0 (90.8-91.3)	87.8 (87.4-88.1)	76.2 (75.7-76.6)	91.1 (90.8-91.3)	87.3 (87.0-87.7)

** Based on data from Austria, Belgium (Dutch-speaking), Belgium (French-speaking), Bosnia and Herzegovina, Denmark, Estonia, France, Greece, Iceland, Norway, Spain (Andalusia), Spain (Aragon), Spain (Asturias), Spain (Basque country), Spain (Canary Islands), Spain (Cantabria), Spain (Castile and León), Spain (Castile-La Mancha), Spain (Catalonia), Spain (Community of Madrid), Spain (Extremadura), Spain (Galicia), Spain (Murcia), Spain (Navarre), Spain (Valencian Region), Sweden, the Netherlands, United Kingdom (England/Northern Ireland/Wales) and United Kingdom (Scotland)*

Table B.6.14

First graft (deceased donor), from day of transplant, adjusted

adjusted for age, sex, and primary renal disease

	Survival probabilities as % (95% CI)				
	cohort 2013 - 2017			cohort 2016 - 2020	
	1 year	2 year	5 year	1 year	2 year
0-19	94.3 (92.8-95.7)	91.8 (90.1-93.6)	84.6 (82.4-86.9)	94.3 (92.8-95.7)	91.8 (90.1-93.6)
20-44	94.4 (93.9-94.8)	92.5 (92.0-93.1)	85.6 (84.8-86.3)	95.2 (94.7-95.6)	93.2 (92.6-93.7)
45-64	92.3 (91.9-92.7)	89.6 (89.1-90.0)	79.7 (79.2-80.3)	92.4 (92.1-92.8)	89.5 (89.1-89.9)
65+	86.6 (85.9-87.2)	81.4 (80.6-82.1)	62.9 (62.0-63.9)	86.6 (86.0-87.2)	80.4 (79.7-81.1)
Male	93.2 (93.0-93.5)	90.5 (90.2-90.8)	80.6 (80.1-81.1)	93.4 (93.1-93.7)	90.4 (90.1-90.8)
Female	92.9 (92.5-93.3)	90.5 (90.1-91.0)	81.4 (80.8-82.1)	93.5 (93.1-93.8)	90.8 (90.4-91.3)
Diabetes	91.9 (91.4-92.5)	88.5 (87.9-89.2)	75.7 (74.7-76.6)	91.7 (91.2-92.3)	87.9 (87.2-88.6)
Hypertension / renal vascular disease	92.7 (92.1-93.3)	89.5 (88.8-90.3)	78.7 (77.7-79.8)	93.4 (92.8-93.9)	90.0 (89.3-90.7)
Glomerulonephritis	93.2 (92.7-93.7)	90.8 (90.2-91.4)	81.7 (80.9-82.5)	93.6 (93.1-94.1)	90.9 (90.4-91.5)
Other causes	93.2 (92.8-93.5)	90.8 (90.4-91.2)	82.0 (81.5-82.5)	93.6 (93.3-93.9)	91.0 (90.6-91.4)
All	93.1 (92.9-93.4)	90.5 (90.2-90.8)	80.9 (80.5-81.3)	93.4 (93.2-93.7)	90.6 (90.3-90.9)

** Based on data from Austria, Belgium (Dutch-speaking), Belgium (French-speaking), Bosnia and Herzegovina, Denmark, Estonia, France, Greece, Iceland, Norway, Spain (Andalusia), Spain (Aragon), Spain (Asturias), Spain (Basque country), Spain (Canary Islands), Spain (Cantabria), Spain (Castile and León), Spain (Castile-La Mancha), Spain (Catalonia), Spain (Community of Madrid), Spain (Extremadura), Spain (Galicia), Spain (Murcia), Spain (Navarre), Spain (Valencian Region), Sweden, the Netherlands, United Kingdom (England/Northern Ireland/Wales) and United Kingdom (Scotland)*

Table B.6.15

First graft (living donor), from day of transplant, unadjusted

by age, sex, and primary renal disease

	Survival probabilities as % (95% CI)				
	cohort 2013 - 2017			cohort 2016 - 2020	
	**1 year**	**2 year**	**5 year**	**1 year**	**2 year**
0-19	96.4 (95.0-97.4)	94.6 (92.9-95.9)	88.7 (86.5-90.6)	96.6 (95.1-97.6)	94.7 (93.0-96.0)
20-44	96.8 (96.2-97.3)	95.7 (95.1-96.2)	89.8 (88.9-90.7)	97.3 (96.8-97.8)	96.2 (95.6-96.8)
45-64	96.8 (96.3-97.2)	95.2 (94.7-95.8)	89.4 (88.6-90.2)	96.6 (96.0-97.0)	95.0 (94.4-95.6)
65+	95.1 (93.9-96.0)	92.1 (90.6-93.3)	78.4 (76.3-80.4)	95.7 (94.6-96.6)	92.7 (91.3-93.8)
Male	96.4 (95.9-96.8)	95.0 (94.5-95.5)	88.5 (87.8-89.2)	96.6 (96.1-96.9)	95.1 (94.6-95.5)
Female	96.8 (96.3-97.3)	94.9 (94.2-95.5)	87.5 (86.5-88.4)	97.0 (96.4-97.4)	95.0 (94.3-95.7)
Diabetes	95.4 (93.9-96.6)	92.5 (90.7-94.0)	81.1 (78.4-83.4)	95.8 (94.2-96.9)	92.5 (90.6-94.1)
Hypertension / renal vascular disease	96.3 (94.9-97.2)	93.2 (91.5-94.5)	82.7 (80.2-84.9)	95.7 (94.2-96.8)	92.9 (91.0-94.4)
Glomerulonephritis	96.4 (95.7-97.0)	95.3 (94.5-95.9)	88.7 (87.5-89.7)	96.7 (96.0-97.3)	95.5 (94.7-96.2)
Other causes	96.8 (96.4-97.2)	95.5 (95.0-96.0)	89.8 (89.0-90.5)	96.9 (96.5-97.3)	95.5 (95.0-96.0)
All	96.5 (96.2-96.8)	95.0 (94.6-95.3)	88.1 (87.6-88.7)	96.7 (96.4-97.0)	95.1 (94.7-95.4)

** Based on data from Austria, Belgium (Dutch-speaking), Belgium (French-speaking), Bosnia and Herzegovina, Denmark, Estonia, France, Greece, Iceland, Norway, Spain (Andalusia), Spain (Aragon), Spain (Asturias), Spain (Basque country), Spain (Canary Islands), Spain (Cantabria), Spain (Castile and León), Spain (Castile-La Mancha), Spain (Catalonia), Spain (Community of Madrid), Spain (Extremadura), Spain (Galicia), Spain (Murcia), Spain (Navarre), Spain (Valencian Region), Sweden, the Netherlands, United Kingdom (England/Northern Ireland/Wales) and United Kingdom (Scotland)*

Table B.6.16
First graft (living donor), from day of transplant, adjusted
adjusted for age, sex, and primary renal disease

	Survival probabilities as % (95% CI)				
	cohort 2013 - 2017			cohort 2016 - 2020	
	1 year	2 year	5 year	1 year	2 year
0-19	95.9 (94.5-97.3)	93.8 (92.1-95.5)	87.1 (84.7-89.5)	96.1 (94.7-97.5)	94.1 (92.4-95.8)
20-44	96.7 (96.2-97.2)	95.6 (95.0-96.2)	89.4 (88.5-90.4)	97.1 (96.6-97.7)	96.0 (95.3-96.6)
45-64	96.8 (96.3-97.2)	95.3 (94.8-95.9)	89.5 (88.7-90.3)	96.5 (96.0-97.0)	95.0 (94.4-95.6)
65+	95.4 (94.3-96.4)	92.4 (91.2-93.8)	79.3 (77.3-81.4)	95.9 (95.0-96.9)	93.0 (91.8-94.2)
Male	96.3 (95.9-96.8)	95.0 (94.5-95.5)	88.3 (87.6-89.1)	96.5 (96.1-96.9)	95.0 (94.5-95.5)
Female	96.6 (96.0-97.1)	94.5 (93.8-95.2)	86.5 (85.5-87.5)	96.7 (96.2-97.3)	94.8 (94.0-95.5)
Diabetes	95.8 (94.5-97.0)	93.0 (91.5-94.6)	82.2 (79.9-84.6)	96.2 (95.0-97.4)	93.2 (91.6-94.8)
Hypertension / renal vascular disease	96.5 (95.5-97.6)	93.7 (92.3-95.1)	83.8 (81.7-86.1)	96.2 (95.0-97.3)	93.6 (92.2-95.2)
Glomerulonephritis	96.1 (95.4-96.8)	94.9 (94.1-95.7)	87.8 (86.6-89.0)	96.4 (95.7-97.1)	95.2 (94.4-96.0)
Other causes	96.7 (96.3-97.1)	95.4 (94.9-95.9)	89.4 (88.7-90.1)	96.8 (96.4-97.2)	95.3 (94.8-95.9)
All	96.4 (96.1-96.8)	94.8 (94.4-95.2)	87.7 (87.1-88.3)	96.6 (96.2-96.9)	94.9 (94.5-95.3)

** Based on data from Austria, Belgium (Dutch-speaking), Belgium (French-speaking), Bosnia and Herzegovina, Denmark, Estonia, France, Greece, Iceland, Norway, Spain (Andalusia), Spain (Aragon), Spain (Asturias), Spain (Basque country), Spain (Canary Islands), Spain (Cantabria), Spain (Castile and León), Spain (Castile-La Mancha), Spain (Catalonia), Spain (Community of Madrid), Spain (Extremadura), Spain (Galicia), Spain (Murcia), Spain (Navarre), Spain (Valencian Region), Sweden, the Netherlands, United Kingdom (England/Northern Ireland/Wales) and United Kingdom (Scotland)*

Table B.7.1

Expected remaining years of life in the general population and in prevalent dialysis and kidney transplant patients (cohort 2018-2022)

by age and sex

General population				ESRD: Dialysis				ESRD: Transplant			
Age	All	Male	Female	Age	All	Male	Female	Age	All	Male	Female
20-24	62.1	59.8	64.4	20-24	20.8	21.2	20.2	20-24	41.4	41.3	41.7
25-29	57.2	54.9	59.4	25-29	17.9	18.1	17.5	25-29	37.1	36.9	37.3
30-34	52.3	50.1	54.5	30-34	15.7	15.8	15.7	30-34	32.9	32.6	33.6
35-39	47.5	45.3	49.6	35-39	13.8	13.8	13.9	35-39	28.8	28.5	29.5
40-44	42.6	40.5	44.7	40-44	11.9	11.7	12.1	40-44	24.8	24.4	25.5
45-49	37.9	35.8	39.9	45-49	10.1	10.0	10.4	45-49	21.0	20.6	21.8
50-54	33.2	31.2	35.2	50-54	8.6	8.4	8.9	50-54	17.5	17.1	18.3
55-59	28.7	26.7	30.5	55-59	7.2	7.0	7.6	55-59	14.4	14.0	15.1
60-64	24.3	22.5	26.0	60-64	6.2	6.0	6.5	60-64	11.5	11.2	12.1
65-69	20.2	18.5	21.7	65-69	5.4	5.2	5.7	65-69	9.1	8.8	9.7
70-74	16.2	14.8	17.5	70-74	4.7	4.6	5.0	70-74	7.1	6.9	7.4
75-79	12.6	11.4	13.5	75-79	4.0	3.9	4.2	75-79	5.3	5.2	5.5
80-84	9.2	8.3	9.9	80-84	3.3	3.3	3.5	80-84	3.6	3.6	3.6
85-89	6.4	5.8	6.8	85-89	2.1	2.1	2.2	85-89	2.0	2.2	1.8
90-94	4.3	3.9	4.5	90-94	1.5	1.5	1.6				

Based on data from Austria, Belgium (Dutch-speaking), Belgium (French-speaking), Bosnia and Herzegovina, Denmark, Estonia, France, Greece, Iceland, Norway, Spain (Andalusia), Spain (Aragon), Spain (Asturias), Spain (Basque country), Spain (Canary Islands), Spain (Cantabria), Spain (Castile and León), Spain (Castile-La Mancha), Spain (Catalonia), Spain (Community of Madrid), Spain (Extremadura), Spain (Galicia), Spain (Murcia), Spain (Navarre), Spain (Valencian Region), Sweden, the Netherlands, United Kingdom (England/Northern Ireland/Wales) and United Kingdom (Scotland)

Section C: Aggregated data reference tables

Table C.1.1
General population data and number of renal centres

	General population of country/region in thousands	% Coverage of general population by the registry	Total number of renal centres in country/region	Number of renal centres collaborating with the registry
Belarus ‡	9228	92.0	54	50
Croatia	3856	82.0	47	42
Cyprus	905	100	7	7
Czech Republic	10828	98.0	115	113
Finland	5564	100	30	30
Hungary	9689	100	68	68
Israel	9557	100	79	79
Italy (8 of 20 regions) §	59030	46.2	785	309
Kosovo	1740	97.0	9	8
Latvia	1876	89.0	33	28
Lithuania	2806	100	66	66
North Macedonia	1830	100	23	23
Poland	37827	100	280	280
Portugal	10467	100	135	135
Slovakia	5432	80.3	76	61
Spain	47475	100	362	353
Tunisia, Sfax region	1023	100	18	18
Turkey	85280	100	1129	1129
Ukraine	40998	50.4	139	101

‡ Data from one pediatric centre and one centre with acute kidney injury patients are excluded
§ The data in this report covers 8 out of 20 regions (Emilia-Romagna, Liguria, Marche, Piedmont, Puglia, Sicilia, Valle d'Aosta and Veneto); the coverage for all regions was 100% except within Piedmont (95% coverage)

Table C.2.1
Incident counts and percentages by age and sex
at day 1

	All			0-19						20-44						45-64						65-74						75+					
	All	Male	Female	All		Male		Female		All		Male		Female		All		Male		Female		All		Male		Female		All		Male		Female	
	N (100%)	N (100%)	N (100%)	N	%	N	%	N	%	N	%	N	%	N	%	N	%	N	%	N	%	N	%	N	%	N	%	N	%	N	%	N	%
Belarus *	888	514	374	2	0	0	0	2	1	172	19	110	21	62	17	386	43	216	42	170	45	267	30	152	30	115	31	61	7	36	7	25	7
Croatia †	472	312	160	1	0	1	0	0	0	32	7	18	6	14	9	94	20	65	21	29	18	124	26	88	28	36	23	221	47	140	45	81	51
Cyprus	277	197	80	1	0	1	1	0	0	15	5	8	4	7	9	62	22	53	27	9	11	84	30	65	33	19	24	115	42	70	36	45	56
Czech Republic †	2502																																
Finland	465	298	167	7	2	4	1	3	2	62	13	43	14	19	11	153	33	94	32	59	35	125	27	79	27	46	28	118	25	78	26	40	24
Hungary	2186	1276	910	24	1	17	1	7	1	178	8	106	8	72	8	665	30	413	32	252	28	719	33	442	35	277	30	600	27	298	23	302	33
Israel	1703	1122	581	36	2	21	2	15	3	146	9	91	8	55	9	477	28	306	27	171	29	518	30	359	32	159	27	526	31	345	31	181	31
Italy (8 of 20 regions)	4362	2860	1502	22	1	13	0	9	1	281	6	188	7	93	6	1222	28	798	28	424	28	1121	26	752	26	369	25	1716	39	1109	39	607	40
Kosovo *	254	150	104	2	1	0	0	2	2	34	13	22	15	12	12	85	33	51	34	34	33	88	35	50	33	38	37	45	18	27	18	18	17
Latvia	114	69	45	1	1	0	0	1	2	18	16	10	14	8	18	44	39	27	39	17	38	33	29	24	35	9	20	18	16	8	12	10	22
Lithuania	227	144	83	2	1	1	1	1	1	29	13	21	15	8	10	79	35	49	34	30	36	63	28	43	30	20	24	54	24	30	21	24	29
North Macedonia	385	246	139	0	0	0	0	0	0	43	11	29	12	14	10	132	34	88	36	44	32	129	34	74	30	55	40	81	21	55	22	26	19
Poland	6068																																
Portugal	2515																																
Slovakia †	710	442	268	5	1	1	0	4	1	81	11	54	12	27	10	250	35	163	37	87	32	227	32	140	32	87	32	147	21	84	19	63	24
Spain §	7136	4722	2414	34	0	23	0	11	0	686	10	411	9	275	11	2189	31	1398	30	791	33	1987	28	1369	29	618	26	2240	31	1521	32	719	30
Tunisia, Sfax region †	184	112	72	1	1	0	0	1	1	33	18	22	20	11	15	55	30	40	36	15	21	41	22	22	20	19	26	54	29	28	25	26	36
Turkey	13725	8139	5586	523	4	294	4	229	4	2309	17	1435	18	874	16	5091	37	3185	39	1906	34	3384	25	1950	24	1434	26	2418	18	1275	16	1143	20
Ukraine *	1257	717	540	9	1	7	1	2	0	267	21	144	20	123	23	528	42	321	45	207	38	382	30	208	29	174	32	71	6	37	5	34	6

Categories may not add up because of missing values or rounding
When cells are left empty, the data are unavailable
** Patients younger than 18 years of age are not reported*
† Data include dialysis patients only
§ Data presented in column 0-19 refer to patients aged 0-14 years and data presented in column 20-44 refer to patients aged 15-44 years

Table C.2.2

Incidence per million (age-related) population by age and sex, unadjusted

at day 1

	All			0-19			20-44			45-64			65-74			75+		
	All	Male	Female	All	Male	Female	All	Male	Female	All	Male	Female	All	Male	Female	All	Male	Female
	Pmp	Pmp	Pmp	Pmarp	Pmarp	Pmarp	Pmarp	Pmarp	Pmarp	Pmarp	Pmarp	Pmarp	Pmarp	Pmarp	Pmarp	Pmarp	Pmarp	Pmarp
Belarus *	104.6	131.0	81.9	1.1	0	2.2	59.6	76.7	42.7	163.1	200.6	131.8	300.9	453.1	208.3	124.6	291.8	68.2
Croatia †	149.3	204.5	97.8	1.6	3.2	0	33.5	37.0	29.8	106.5	151.4	64.0	296.4	464.3	157.3	745.2	1292.6	430.3
Cyprus	306.2	446.6	172.6	5.2	10.1	0	43.3	47.8	39.1	286.7	505.5	80.8	982.1	1558.7	433.5	1798.0	2499.5	1251.6
Czech Republic †	235.8																	
Finland	83.6	108.2	59.4	6.1	6.8	5.3	35.6	47.8	22.6	110.9	135.7	85.9	180.2	240.0	126.2	196.4	318.9	112.3
Hungary	225.6	274.7	180.4	12.6	17.4	7.6	56.9	66.1	47.2	249.4	317.4	184.5	611.3	898.1	405.0	736.9	1104.7	554.6
Israel	178.2	236.3	120.8	10.5	12.0	9.0	46.6	57.8	35.4	260.6	342.4	182.6	752.9	1124.7	431.1	1064.8	1658.7	632.9
Italy (8 of 20 regions)	160.0	214.9	107.7	4.6	5.3	3.9	37.1	48.7	25.0	145.3	193.0	99.2	352.0	499.4	219.8	519.3	827.3	309.1
Kosovo *	150.5	176.5	124.1	3.1	0	6.5	52.6	68.0	37.2	297.9	362.5	235.0	1165.1	1368.7	974.4	1191.9	1643.9	843.9
Latvia	68.3	89.2	50.2	2.9	0	5.9	35.5	38.4	32.4	94.9	124.9	68.7	178.0	336.2	79.0	109.9	177.5	84.2
Lithuania	80.9	110.2	55.4	3.7	3.6	3.8	33.2	47.2	18.7	95.7	127.0	68.2	211.7	365.2	111.2	205.3	384.8	129.7
North Macedonia	210.4	271.2	150.6	0	0	0	72.3	95.9	47.8	263.5	357.2	172.8	619.5	756.8	498.0	703.8	1124.4	392.9
Poland	160.4																	
Portugal	240.3																	
Slovakia †	162.8	207.2	120.3	5.5	2.1	9.0	54.4	70.6	37.3	209.7	275.3	144.9	465.8	655.3	317.9	522.8	876.3	339.9
Spain §	150.3	203.0	99.7	3.8	4.9	2.5	46.9	55.6	38.0	152.9	196.7	109.7	416.2	611.0	244.0	476.1	812.9	253.7
Tunisia, Sfax region †	179.3	218.1	140.4	3.1	0	6.4	90.1	121.3	59.5	232.0	338.6	126.1	603.4	675.9	536.8	1600.8	1773.0	1449.1
Turkey	160.9	190.6	131.2	20.9	22.9	18.8	71.1	87.2	54.6	263.8	330.5	197.2	621.0	763.1	495.5	805.4	1067.0	632.4
Ukraine *	60.9	74.9	48.8	2.2	3.3	1.0	37.2	39.7	34.7	91.8	122.9	66.0	176.3	256.9	128.2	48.1	88.8	32.1

When cells are left empty, the data are unavailable
** Patients younger than 18 years of age are not reported*
† Data include dialysis patients only
§ Data presented in column 0-19 refer to patients aged 0-14 years and data presented in column 20-44 refer to patients aged 15-44 years

Table C.2.3

Sex, mean age, and median age of incident patients

at day 1

	All						Male						Female					
	%	Mean (years)	SD	Median (years)	P25	P75	%	Mean (years)	SD	Median (years)	P25	P75	%	Mean (years)	SD	Median (years)	P25	P75
Belarus *	100						57.9						42.1					
Croatia †	100	71.0	14.6	72.0	61.0	79.0	66.1	70.5	13.9	72.0	60.0	77.0	33.9	72.0	14.4	73.0	62.0	79.0
Cyprus	100	69.6	14.1	71.0	64.0	80.0	71.1	68.7	13.4	70.0	63.0	78.0	28.9	71.7	15.4	75.5	66.0	82.3
Czech Republic																		
Finland	100	62.1	16.4	65.1	54.0	74.1	64.1	62.1	16.6	65.6	53.1	74.0	35.9	62.0	16.2	65.0	55.6	74.3
Hungary	100	65.0	14.7	68.0	57.0	75.0	58.4	63.9	14.5	67.0	56.0	74.0	41.6	66.4	14.8	70.0	59.0	77.0
Israel	100	65.8	16.5	69.4	58.5	76.8	65.9	66.0	16.1	69.5	58.7	76.5	34.1	65.4	17.2	68.7	57.5	77.4
Italy (8 of 20 regions)	100	68.7	14.8	71.4	59.9	79.5	65.6	68.6	14.7	71.0	59.9	79.3	34.4	68.9	15.0	71.8	59.9	80.0
Kosovo *	100	62.4	14.7	66.0	56.0	73.0	59.1	62.2	15.1	65.0	55.0	72.0	40.9	62.7	14.2	66.5	58.0	73.0
Latvia	100	59.8	15.6	62.0	49.0	72.0	60.5	60.4	13.1	63.0	53.0	70.0	39.5	58.9	19.1	59.0	47.0	74.0
Lithuania	100	62.6	16.3	64.3	54.6	73.2	63.4	61.9	15.9	64.2	54.2	71.7	36.6	63.9	16.9	65.6	55.2	77.0
North Macedonia	100	63.6	13.9	66.0	55.0	74.0	63.9	63.1	14.2	65.0	54.0	73.3	36.1	64.6	13.2	67.0	57.0	74.0
Poland																		
Portugal																		
Slovakia †	100	62.9	14.8	65.0	54.0	73.0	62.3	62.2	14.8	65.0	52.8	73.0	37.7	64.1	14.9	67.0	56.0	74.0
Spain	100	63.8	14.9	68.3	54.7	77.3	66.2	64.4	14.6	68.9	55.7	77.5	33.8	62.6	15.4	67.1	53.0	76.9
Tunisia, Sfax region †	100	61.9	17.4	65.0	49.3	77.0	60.9	60.3	17.4	62.0	48.0	74.8	39.1	64.4	17.3	68.5	51.8	78.8
Turkey	100						59.3						40.7					
Ukraine *	100	54.5	14.1	56.0	45.0	64.0	57.0	53.9	13.2	56.0	45.0	64.0	43.0	55.2	15.2	56.0	45.0	66.0

Categories may not add up because of missing values or rounding
When cells are left empty, the data are unavailable
** Patients younger than 18 years of age are not reported*
† Data include dialysis patients only

Table C.2.4
Incidence per million population, adjusted
at day 1, adjusted for age and sex

	All	Male	Female
	Pmp	Pmp	Pmp
Belarus			
Croatia †	152.6	202.8	104.6
Cyprus	375.9	526.4	232.0
Czech Republic			
Finland	81.5	105.7	58.4
Hungary	239.4	301.5	180.1
Israel	281.5	376.0	191.2
Italy (8 of 20 regions)	143.3	190.7	98.0
Kosovo *	355.2	425.4	288.1
Latvia	71.8	96.4	48.3
Lithuania	85.6	119.4	53.4
North Macedonia	234.2	303.1	168.2
Poland			
Portugal			
Slovakia †	184.6	243.0	128.7
Spain			
Tunisia, Sfax region †	243.1	285.0	203.1
Turkey			
Ukraine			

When cells are left empty, the data are unavailable
** Patients younger than 18 years of age are not reported*
† Data include dialysis patients only

Table C.2.5

Incidence per million population and percentages by primary renal disease (1995 PRD codes), unadjusted

at day 1

	All		GN		PN		PKD		DM						HT		RVD		Misc		Unkn		Missing	
									Type 1		Type 2		Both											
	Pmp	%	Pmp	%	Pmp	%	Pmp	%	Pmp	%	Pmp	%	Pmp	%	Pmp	%	Pmp	%	Pmp	%	Pmp	%	Pmp	%
Belarus *	104.6	100	23.8	22.7	9.1	8.7	8.0	7.7	4.7	4.5	14.1	13.5	18.8	18.0	12.5	11.9	6.8	6.5	24.9	23.8	0.7	0.7	0	0
Croatia †	149.3	100	18.3	12.3	1.6	1.1	12.7	8.5	5.7	3.8	39.2	26.3	44.9	30.1	34.2	22.9	3.5	2.3	26.9	18.0	7.3	4.9	0	0
Cyprus	306.2	100	24.3	7.9	3.3	1.1	9.9	3.2					118.3	38.6	47.5	15.5	14.4	4.7	32.1	10.5	56.4	18.4	0	0
Czech Republic																								
Finland	83.6	100	10.1	12.0	1.8	2.2	8.8	10.5	11.0	13.1	14.7	17.6	25.7	30.8	6.6	8.0	1.4	1.7	12.8	15.3	13.7	16.3	2.7	3.2
Hungary	225.6	100	40.7	18.0	40.5	17.9	15.3	6.8	36.4	16.1	74.0	32.8	110.4	48.9	8.2	3.6	0.7	0.3	1.2	0.5	4.3	1.9	4.3	1.9
Israel	178.2	100	9.0	5.0	2.4	1.4	4.4	2.5	3.5	1.9	75.0	42.1	78.5	44.0	19.6	11.0	1.0	0.6	19.7	11.0	32.9	18.4	10.8	6.0
Italy (8 of 20 regions)	160.0	100	12.8	8.0	6.9	4.3	9.3	5.8					21.2	13.2	16.7	10.4	5.2	3.3	13.5	8.4	56.3	35.2	18.1	11.3
Kosovo *	150.5	100	13.0	8.7	39.7	26.4	5.9	3.9	1.2	0.8	56.3	37.4	57.5	38.2	23.1	15.4	3.0	2.0	6.5	4.3	1.8	1.2	0	0
Latvia	68.3	100	15.6	22.8	5.4	7.9	8.4	12.3	3.6	5.3	10.8	15.8	14.4	21.1	6.6	9.6	0	0	17.4	25.4	0.6	0.9	0	0
Lithuania	80.9	100	12.8	15.9	6.8	8.4	7.5	9.3					13.2	16.3	15.3	18.9	2.1	2.6	16.7	20.7	6.4	7.9	0	0
North Macedonia	210.4	100	9.3	4.4	10.4	4.9	13.7	6.5	4.9	2.3	41.5	19.7	46.4	22.1	69.9	33.2	1.6	0.8	25.7	12.2	33.3	15.8	0	0
Poland	160.4	100					8.3	5.2					40.3	25.1									111.8	69.7
Portugal ‡ + &	240.3	100	26.5	11.0			14.8	6.2					69.8	29.1	31.9	13.3			52.6	21.9	40.3	16.8	2.1	0.9
Slovakia †	162.8	100	14.9	9.2	18.1	11.1	3.2	2.0					49.3	30.3	11.2	6.9	11.2	6.9	53.6	33.0	1.1	0.7	0	0
Spain ¶ §	150.3	100	21.4	14.2									33.2	22.1	20.4	13.6			65.2	43.4			10.2	6.8
Tunisia, Sfax region † $	179.3	100	14.6	8.2	25.3	14.1	8.8	4.9					54.6	30.4			5.8	3.3	1.0	0.5	56.5	31.5	12.7	7.1
Turkey	160.9	100	3.4	2.1	0.6	0.4	3.2	2.0					29.3	18.2	25.1	15.6	0.5	0.3	8.5	5.3	9.4	5.8	80.9	50.3
Ukraine * +	60.9	100	11.8	19.3	8.6	14.2	5.5	9.1	3.4	5.6	10.9	17.9	14.3	23.5	7.4	12.1			11.0	18.1	1.6	2.7	0.6	1.0

Abbreviations used: GN: glomerulonephritis/sclerosis; PN: pyelonephritis; PKD: polycystic kidneys, adult type; DM: diabetes mellitus; HT: hypertension; RVD: renal vascular disease; Misc: miscellaneous; Unkn: unknown
Categories may not add up because of rounding
When cells are left empty, the data are unavailable
** Patients younger than 18 years of age are not reported*
† Data include dialysis patients only
‡ Data on primary renal disease are available for dialysis patients only (N=2492, 99.1% of total)
+ Renal vascular disease is not reported separately, but is included in miscellaneous
& Pyelonephritis is not reported separately, but is included in miscellaneous
¶ Renal vascular disease is not reported separately, but is included in hypertension
§ Miscellaneous renal disorders include: Tubulointerstitial disease (n=643); Other systemic diseases affecting the kidney (n=286), Familial/Hereditary nephropathies (n=615), and Miscellaneous renal disorders (n=1551)
$ Hypertension is not reported separately, but is included in renal vascular disease

Table C.2.6

Incident counts by treatment modality

at day 1

	All	Haemodialysis						Peritoneal dialysis				Preemptive kidney transplant				Missing
		HD hospital/ centre	HD home	HD type Unkn	HF	HDF	Total HD/ HF/HDF	APD	CAPD	PD type Unkn	Total PD	Living donor	Deceased donor	Tx type Unkn	Total Tx	
	N	N	N	N	N	N	N	N	N	N	N	N	N	N	N	N
Belarus *	888	790	0	0	0	36	826	1	20	0	21	3	38	0	41	0
Croatia †	472	381	0	0	0	61	442			30	30					0
Cyprus	277	96	0	0	0	166	262	0	3	12	15	0	0	0	0	0
Czech Republic †	2502			2410			2410			92	92					0
Finland	465	294	11	0	0	8	313	33	90	0	123	10	19	0	29	0
Hungary	2186	1665	0	0	56	324	2045	0	0	123	123	12	6	0	18	0
Israel	1703	1413	0	0	0	56	1469	27	123	1	151	79	4	0	83	0
Italy (8 of 20 regions)	4362	2926	8	0	150	611	3695	271	337	4	612	26	29	0	55	0
Kosovo *	254	251	0	0	0	0	251	0	0	0	0	3	0	0	3	0
Latvia	114	95	0	0	0	0	95	7	11	0	18	0	1	0	1	0
Lithuania	227	200	0	0	0	0	200			19	19	2	6	0	8	0
North Macedonia	385	379	0	0	0	2	381	0	4	0	4	0	0	0	0	0
Poland † §	6068	5735	0	0	0	0	5735	0	0	333	333					0
Portugal	2515			2235			2235			257	257			23	23	0
Slovakia †	710	135	0	0	0	552	687	15	6	0	21					2
Spain	7136			5047			5047			1722	1722			367	367	0
Tunisia, Sfax region †	184	180	0	0	0	0	180	2	2	0	4					0
Turkey	13725	10145	53	125	3	14	10340	425	942	0	1367	2017	1	0	2018	0
Ukraine *	1257	863	0	0	0	292	1155	17	85	0	102	0	0	0	0	0

Abbreviations used: HD: haemodialysis; Unkn: unknown; HF: haemofiltration; HDF: haemodiafiltration; APD: automated peritoneal dialysis; CAPD: continuous ambulatory peritoneal dialysis; PD: peritoneal dialysis; Tx: transplant
When cells are left empty, the data are unavailable
** Patients younger than 18 years of age are not reported*
† Data include dialysis patients only
§ Additionally, 88 preemptive kidney transplantations (from unknown donor type) were performed in Poland in 2022

Table C.2.7

Incidence per million population by treatment modality, unadjusted

at day 1

	All	Haemodialysis						Peritoneal dialysis				Preemptive kidney transplant				Missing
		HD hospital/ centre	HD home	HD type Unkn	HF	HDF	Total HD/ HF/HDF	APD	CAPD	PD type Unkn	Total PD	Living donor	Deceased donor	Tx type Unkn	Total Tx	
	Pmp	Pmp	Pmp	Pmp	Pmp	Pmp	Pmp	Pmp	Pmp	Pmp	Pmp	Pmp	Pmp	Pmp	Pmp	Pmp
Belarus *	104.6	93.1	0	0	0	4.2	97.3	0.1	2.4	0	2.5	0.4	4.5	0	4.8	0
Croatia †	149.3	120.5	0	0	0	19.3	139.8			9.5	9.5					0
Cyprus	306.2	106.1	0	0	0	183.5	289.6	0	3.3	13.3	16.6	0	0	0	0	0
Czech Republic †	235.8			227.1			227.1			8.7	8.7					0
Finland	83.6	52.8	2.0	0	0	1.4	56.3	5.9	16.2	0	22.1	1.8	3.4	0	5.2	0
Hungary	225.6	171.8	0	0	5.8	33.4	211.1	0	0	12.7	12.7	1.2	0.6	0	1.9	0
Israel	178.2	147.8	0	0	0	5.9	153.7	2.8	12.9	0.1	15.8	8.3	0.4	0	8.7	0
Italy (8 of 20 regions)	160.0	107.3	0.3	0	5.5	22.4	135.5	9.9	12.4	0.1	22.4	1.0	1.1	0	2.0	0
Kosovo *	150.5	148.7	0	0	0	0	148.7	0	0	0	0	1.8	0	0	1.8	0
Latvia	68.3	56.9	0	0	0	0	56.9	4.2	6.6	0	10.8	0	0.6	0	0.6	0
Lithuania	80.9	71.3	0	0	0	0	71.3			6.8	6.8	0.7	2.1	0	2.9	0
North Macedonia	210.4	207.1	0	0	0	1.1	208.2	0	2.2	0	2.2	0	0	0	0	0
Poland † §	160.4	151.6	0	0	0	0	151.6	0	0	8.8	8.8					0
Portugal	240.3			213.5			213.5			24.6	24.6			2.2	2.2	0
Slovakia †	162.8	31.0	0	0	0	126.6	157.5	3.4	1.4	0	4.8					0.5
Spain	150.3			106.3			106.3			36.3	36.3			7.7	7.7	0
Tunisia, Sfax region †	179.3	175.4	0	0	0	0	175.4	1.9	1.9	0	3.9					0
Turkey	160.9	119.0	0.6	1.5	0	0.2	121.2	5.0	11.0	0	16.0	23.7	0	0	23.7	0
Ukraine *	60.9	41.8	0	0	0	14.1	55.9	0.8	4.1	0	4.9	0	0	0	0	0

Abbreviations used: HD: haemodialysis; Unkn: unknown; HF: haemofiltration; HDF: haemodiafiltration; APD: automated peritoneal dialysis; CAPD: continuous ambulatory peritoneal dialysis; PD: peritoneal dialysis; Tx: transplant
Categories may not add up because of rounding
When cells are left empty, the data are unavailable
** Patients younger than 18 years of age are not reported*
† Data include dialysis patients only
§ Additionally, 88 preemptive kidney transplantations (from unknown donor type) were performed in Poland in 2022

Table C.2.8
Treatment modality distribution, unadjusted
at day 1

	All	Haemodialysis						Peritoneal dialysis				Preemptive kidney transplant				Missing
		HD hospital/ centre	HD home	HD type Unkn	HF	HDF	Total HD/ HF/HDF	APD	CAPD	PD type Unkn	Total PD	Living donor	Deceased donor	Tx type Unkn	Total Tx	
	%	%	%	%	%	%	%	%	%	%	%	%	%	%	%	%
Belarus *	100	89.0	0	0	0	4.1	93.0	0.1	2.3	0	2.4	0.3	4.3	0	4.6	0
Croatia †	100	80.7	0	0	0	12.9	93.6			6.4	6.4					0
Cyprus	100	34.7	0	0	0	59.9	94.6	0	1.1	4.3	5.4	0	0	0	0	0
Czech Republic †	100			96.3			96.3			3.7	3.7					0
Finland	100	63.2	2.4	0	0	1.7	67.3	7.1	19.4	0	26.5	2.2	4.1	0	6.2	0
Hungary	100	76.2	0	0	2.6	14.8	93.5	0	0	5.6	5.6	0.5	0.3	0	0.8	0
Israel	100	83.0	0	0	0	3.3	86.3	1.6	7.2	0.1	8.9	4.6	0.2	0	4.9	0
Italy (8 of 20 regions)	100	67.1	0.2	0	3.4	14.0	84.7	6.2	7.7	0.1	14.0	0.6	0.7	0	1.3	0
Kosovo *	100	98.8	0	0	0	0	98.8	0	0	0	0	1.2	0	0	1.2	0
Latvia	100	83.3	0	0	0	0	83.3	6.1	9.6	0	15.8	0	0.9	0	0.9	0
Lithuania	100	88.1	0	0	0	0	88.1			8.4	8.4	0.9	2.6	0	3.5	0
North Macedonia	100	98.4	0	0	0	0.5	99.0	0	1.0	0	1.0	0	0	0	0	0
Poland † §	100	94.5	0	0	0	0	94.5	0	0	5.5	5.5					0
Portugal	100			88.9			88.9			10.2	10.2			0.9	0.9	0
Slovakia †	100	19.0	0	0	0	77.7	96.8	2.1	0.8	0	3.0					0.3
Spain	100			70.7			70.7			24.1	24.1			5.1	5.1	0
Tunisia, Sfax region †	100	97.8	0	0	0	0	97.8	1.1	1.1	0	2.2					0
Turkey	100	73.9	0.4	0.9	0	0.1	75.3	3.1	6.9	0	10.0	14.7	0	0	14.7	0
Ukraine *	100	68.7	0	0	0	23.2	91.9	1.4	6.8	0	8.1	0	0	0	0	0

Abbreviations used: HD: haemodialysis; Unkn: unknown; HF: haemofiltration; HDF: haemodiafiltration; APD: automated peritoneal dialysis; CAPD: continuous ambulatory peritoneal dialysis; PD: peritoneal dialysis; Tx: transplant
Categories may not add up because of rounding
When cells are left empty, the data are unavailable
** Patients younger than 18 years of age are not reported*
† Data include dialysis patients only
§ Additionally, 88 preemptive kidney transplantations (from unknown donor type) were performed in Poland in 2022

Table C.3.1
Incident counts and percentages by age and sex
at day 91

	All			0-19						20-44						45-64						65-74						75+					
	All	Male	Female	All		Male		Female		All		Male		Female		All		Male		Female		All		Male		Female		All		Male		Female	
	N (100%)	N (100%)	N (100%)	N	%	N	%	N	%	N	%	N	%	N	%	N	%	N	%	N	%	N	%	N	%	N	%	N	%	N	%	N	%
Belarus *	714	409	305	2	0	0	0	2	1	157	22	101	25	56	18	308	43	172	42	136	45	197	28	109	27	88	29	50	7	27	7	23	8
Croatia †	408	272	136	1	0	1	0	0	0	31	8	18	7	13	10	91	22	63	23	28	21	112	27	79	29	33	24	173	42	111	41	62	46
Cyprus	270	190	80	1	0	1	1	0	0	15	6	8	4	7	9	61	23	52	27	9	11	83	31	64	34	19	24	110	41	65	34	45	56
Czech Republic																																	
Finland	457	293	164	7	2	4	1	3	2	62	14	43	15	19	12	150	33	91	31	59	36	122	27	78	27	44	27	116	25	77	26	39	24
Hungary	1582	936	646	24	2	17	2	7	1	166	10	99	11	67	10	545	34	346	37	199	31	505	32	304	32	201	31	342	22	170	18	172	27
Israel	1600	1068	532	36	2	21	2	15	3	146	9	91	9	55	10	463	29	299	28	164	31	487	30	345	32	142	27	468	29	312	29	156	29
Italy (8 of 20 regions)	4041	2653	1388	21	1	12	0	9	1	266	7	174	7	92	7	1182	29	771	29	411	30	1051	26	703	26	348	25	1521	38	993	37	528	38
Kosovo *	206	126	80	2	1	0	0	2	3	30	15	22	17	8	10	77	37	46	37	31	39	72	35	44	35	28	35	25	12	14	11	11	14
Latvia	107	66	41	1	1	0	0	1	2	18	17	10	15	8	20	40	37	26	39	14	34	30	28	22	33	8	20	18	17	8	12	10	24
Lithuania	222	141	81	2	1	1	1	1	1	29	13	21	15	8	10	78	35	48	34	30	37	62	28	43	30	19	23	51	23	28	20	23	28
North Macedonia	315	205	110	0	0	0	0	0	0	40	13	28	14	12	11	121	38	82	40	39	35	97	31	56	27	41	37	57	18	39	19	18	16
Poland																																	
Portugal	2404																																
Slovakia †	520	318	202	2	0	1	0	1	0	62	12	36	11	26	13	181	35	117	37	64	32	166	32	102	32	64	32	109	21	62	19	47	23
Spain																																	
Tunisia, Sfax region †	161	100	61	1	1	0	0	1	2	32	20	22	22	10	16	52	32	38	38	14	23	36	22	19	19	17	28	40	25	21	21	19	31
Turkey																																	
Ukraine																																	

Categories may not add up because of missing values or rounding
When cells are left empty, the data are unavailable
** Patients younger than 18 years of age are not reported*
† Data include dialysis patients only

Table C.3.2
Incidence per million (age-related) population by age and sex, unadjusted
at day 91

	All			0-19			20-44			45-64			65-74			75+		
	All	Male	Female	All	Male	Female	All	Male	Female	All	Male	Female	All	Male	Female	All	Male	Female
	Pmp	Pmp	Pmp	Pmarp	Pmarp	Pmarp	Pmarp	Pmarp	Pmarp	Pmarp	Pmarp	Pmarp	Pmarp	Pmarp	Pmarp	Pmarp	Pmarp	Pmarp
Belarus *	84.1	104.3	66.8	1.1	0	2.2	54.4	70.5	38.5	130.1	159.7	105.4	222.0	324.9	159.4	102.1	218.9	62.8
Croatia †	129.0	178.3	83.1	1.6	3.2	0	32.4	37.0	27.6	103.1	146.8	61.8	267.7	416.8	144.2	583.3	1024.8	329.3
Cyprus	298.4	430.8	172.6	5.2	10.1	0	43.3	47.8	39.1	282.0	495.9	80.8	970.4	1534.7	433.5	1719.9	2320.9	1251.6
Czech Republic																		
Finland	82.1	106.4	58.4	6.1	6.8	5.3	35.6	47.8	22.6	108.7	131.4	85.9	175.9	236.9	120.7	193.1	314.8	109.5
Hungary	163.3	201.5	128.1	12.6	17.4	7.6	53.1	61.7	44.0	204.4	265.9	145.7	429.4	617.7	293.9	420.0	630.2	315.9
Israel	167.4	224.9	110.6	10.5	12.0	9.0	46.6	57.8	35.4	253.0	334.6	175.1	707.8	1080.8	385.0	947.4	1500.0	545.5
Italy (8 of 20 regions)	148.2	199.3	99.5	4.4	4.9	3.9	35.1	45.0	24.7	140.6	186.4	96.2	330.0	466.9	207.3	460.3	740.8	268.9
Kosovo *	122.1	148.3	95.5	3.1	0	6.5	46.4	68.0	24.8	269.8	327.0	214.2	953.3	1204.5	718.0	662.2	852.4	515.7
Latvia	64.1	85.3	45.7	2.9	0	5.9	35.5	38.4	32.4	86.3	120.2	56.6	161.9	308.2	70.2	109.9	177.5	84.2
Lithuania	79.1	107.9	54.0	3.7	3.6	3.8	33.2	47.2	18.7	94.4	124.4	68.2	208.3	365.2	105.6	193.9	359.1	124.3
North Macedonia	172.1	226.0	119.2	0	0	0	67.2	92.6	41.0	241.5	332.9	153.2	465.9	572.7	371.2	495.2	797.3	272.0
Poland																		
Portugal	229.7																	
Slovakia †	119.2	149.1	90.6	2.2	2.1	2.2	41.6	47.1	35.9	151.8	197.6	106.6	340.7	477.4	233.9	387.7	646.8	253.6
Spain																		
Tunisia, Sfax region †	156.8	194.7	118.9	3.1	0	6.4	87.4	121.3	54.1	219.3	321.7	117.7	529.8	583.7	480.3	1185.7	1329.8	1059.0
Turkey																		
Ukraine																		

When cells are left empty, the data are unavailable
** Patients younger than 18 years of age are not reported*
† Data include dialysis patients only

Table C.3.3
Sex, mean age, and median age of incident patients
at day 91

	All						Male						Female					
	%	Mean (years)	SD	Median (years)	P25	P75	%	Mean (years)	SD	Median (years)	P25	P75	%	Mean (years)	SD	Median (years)	P25	P75
Belarus *	100						57.3						42.7					
Croatia †	100	70.2	14.3	70.0	61.0	79.0	66.7	69.9	13.5	71.0	61.0	76.0	33.3	70.6	14.8	72.0	63.0	79.0
Cyprus	100	69.3	14.0	71.0	64.0	79.8	70.4	68.3	13.2	69.5	63.0	77.0	29.6	71.7	15.4	75.5	66.0	82.3
Czech Republic																		
Finland	100	62.0	16.5	65.1	53.9	74.0	64.1	62.1	16.7	65.7	53.1	74.0	35.9	61.7	16.2	64.9	55.4	74.1
Hungary	100	62.6	15.2	66.0	54.0	73.0	59.2	61.7	15.0	65.0	53.0	72.0	40.8	64.0	15.5	67.0	55.0	75.0
Israel	100	65.2	16.6	68.8	57.5	76.3	66.8	65.5	16.1	69.2	58.1	76.2	33.3	64.5	17.5	67.6	56.4	76.6
Italy (8 of 20 regions)	100	68.2	12.8	70.8	59.3	79.1	65.7	68.2	12.7	70.6	59.4	78.9	34.3	68.2	12.9	71.1	59.1	79.4
Kosovo *	100	63.6	13.3	66.5	54.0	70.8	61.2	62.7	14.4	66.0	53.3	70.0	38.8	65.0	11.4	68.0	54.0	71.5
Latvia	100	59.6	16.1	62.0	48.5	72.5	61.7	60.2	13.3	62.5	52.3	69.8	38.3	58.6	19.9	59.0	46.0	74.0
Lithuania	100	62.3	16.2	64.3	54.4	72.6	63.5	61.5	15.8	64.2	53.4	71.1	36.5	63.6	17.0	64.5	54.9	76.4
North Macedonia	100	62.1	14.0	64.0	53.0	72.0	65.1	61.6	14.4	63.0	51.0	71.5	34.9	63.2	13.0	66.0	55.0	72.5
Poland																		
Portugal																		
Slovakia †	100	62.9	14.7	65.0	54.0	73.0	61.2	62.4	14.5	65.0	53.0	73.0	38.8	63.5	15.0	66.0	55.0	74.0
Spain																		
Tunisia, Sfax region †	100	60.2	17.4	63.0	47.5	74.5	62.1	58.6	17.2	59.0	47.0	73.0	37.9	62.8	17.5	67.0	49.0	77.0
Turkey																		
Ukraine																		

Categories may not add up because of missing values or rounding
When cells are left empty, the data are unavailable
** Patients younger than 18 years of age are not reported*
† Data include dialysis patients only

Table C.3.4

Incidence per million population, adjusted

at day 91, adjusted for age and sex

	All	Male	Female
	Pmp	Pmp	Pmp
Belarus			
Croatia †	130.7	181.0	82.6
Cyprus	365.5	505.2	232.0
Czech Republic			
Finland	80.1	103.9	57.4
Hungary	170.8	216.9	126.8
Israel	262.7	356.2	173.2
Italy (8 of 20 regions)	133.1	177.3	90.9
Kosovo *	267.7	326.7	211.3
Latvia	67.5	92.1	44.0
Lithuania	83.5	116.5	52.0
North Macedonia	186.0	249.3	125.6
Poland			
Portugal			
Slovakia †	135.2	175.2	97.0
Spain			
Tunisia, Sfax region †	212.6	252.9	174.1
Turkey			
Ukraine			

When cells are left empty, the data are unavailable
** Patients younger than 18 years of age are not reported*
† Data include dialysis patients only

Table C.3.5

Incidence per million population and percentages by primary renal disease (1995 PRD codes), unadjusted

at day 91

	All		GN		PN		PKD		DM						HT		RVD		Misc		Unkn		Missing	
									Type 1		Type 2		Both											
	Pmp	%	Pmp	%	Pmp	%	Pmp	%	Pmp	%	Pmp	%	Pmp	%	Pmp	%	Pmp	%	Pmp	%	Pmp	%	Pmp	%
Belarus *	84.1	100	22.9	27.2	6.4	7.6	7.1	8.4	3.1	3.6	10.0	11.9	13.1	15.5	12.1	14.4	4.7	5.6	17.7	21.0	0.2	0.3	0	0
Croatia †	129.0	100	17.7	13.7	1.6	1.2	12.3	9.6	5.4	4.2	34.8	27.0	40.2	31.1	29.7	23.0	2.8	2.2	19.0	14.7	5.7	4.4	0	0
Cyprus	298.4	100	24.3	8.1	3.3	1.1	9.9	3.3					112.7	37.8	47.5	15.9	13.3	4.4	32.1	10.7	55.3	18.5	0	0
Czech Republic																								
Finland	82.1	100	10.1	12.3	1.6	2.0	8.8	10.7	10.6	12.9	14.6	17.7	25.2	30.6	6.6	8.1	1.1	1.3	12.8	15.5	13.5	16.4	2.5	3.1
Hungary	163.3	100	32.8	20.1	27.8	17.0	13.7	8.4	23.1	14.2	50.0	30.6	73.1	44.8	6.3	3.9	0.6	0.4	1.1	0.7	3.5	2.1	4.3	2.7
Israel	167.4	100	8.6	5.1	2.3	1.4	4.4	2.6	3.3	2.0	69.6	41.6	72.9	43.6	18.2	10.9	1.0	0.6	18.4	11.0	31.3	18.7	10.3	6.1
Italy (8 of 20 regions)	148.2	100	12.5	8.5	6.2	4.2	9.3	6.3					20.1	13.5	15.7	10.6	5.0	3.3	12.7	8.6	50.5	34.1	16.2	10.9
Kosovo *	122.1	100	12.4	10.2	30.2	24.8	4.7	3.9	1.2	1.0	46.8	38.3	48.0	39.3	19.6	16.0	2.4	1.9	4.1	3.4	0.6	0.5	0	0
Latvia	64.1	100	15.6	24.3	5.4	8.4	7.8	12.1	3.6	5.6	10.2	15.9	13.8	21.5	6.0	9.3	0	0	15.0	23.4	0.6	0.9	0	0
Lithuania	79.1	100	12.8	16.2	6.1	7.7	7.5	9.5					12.8	16.2	15.0	18.9	2.1	2.7	16.4	20.7	6.4	8.1	0	0
North Macedonia	172.1	100	9.3	5.4	7.7	4.4	13.1	7.6	4.4	2.5	36.6	21.3	41.0	23.8	54.1	31.4	1.1	0.6	21.9	12.7	24.0	14.0	0	0
Poland																								
Portugal																								
Slovakia †	119.2	100	11.2	9.4	14.2	11.9	2.5	2.1					36.7	30.8	9.4	7.9	8.5	7.1	36.0	30.2	0.7	0.6	0	0
Spain																								
Tunisia, Sfax region † $	156.8	100	13.6	8.7	22.4	14.3	8.8	5.6					46.8	29.8			5.8	3.7	1.0	0.6	46.8	29.8	11.7	7.5
Turkey																								
Ukraine																								

Abbreviations used: GN: glomerulonephritis/sclerosis; PN: pyelonephritis; PKD: polycystic kidneys, adult type; DM: diabetes mellitus; HT: hypertension; RVD: renal vascular disease; Misc: miscellaneous; Unkn: unknown
Categories may not add up because of rounding
When cells are left empty, the data are unavailable
** Patients younger than 18 years of age are not reported*
† Data include dialysis patients only
$ Hypertension is not reported separately, but is included in renal vascular disease

Table C.3.6

Incident counts by treatment modality

at day 91

	All	Haemodialysis						Peritoneal dialysis				Kidney transplant				Missing
		HD hospital/ centre	HD home	HD type Unkn	HF	HDF	Total HD/ HF/HDF	APD	CAPD	PD type Unkn	Total PD	Living donor	Deceased donor	Tx type Unkn	Total Tx	
	N	N	N	N	N	N	N	N	N	N	N	N	N	N	N	N
Belarus *	714	558	0	0	8	84	650	2	21	0	23	3	38	0	41	0
Croatia †	408	322	0	0	0	61	383			25	25					0
Cyprus	270	94	0	0	0	161	255	0	3	12	15	0	0	0	0	0
Czech Republic																
Finland	457	251	11	0	0	44	306	58	58	0	116	11	23	1	35	0
Hungary	1582	1187	0	0	33	225	1445	0	0	119	119	12	6	0	18	0
Israel	1600	1312	0	0	0	52	1364	27	116	0	143	89	4	0	93	0
Italy (8 of 20 regions)	4041	2688	7	4	149	541	3389	266	332	0	598	28	26	0	54	0
Kosovo *	206	193	0	0	0	0	193	0	0	0	0	13	0	0	13	0
Latvia	107	88	0	0	0	0	88	7	11	0	18	0	1	0	1	0
Lithuania	222	195	0	0	0	0	195			19	19	2	6	0	8	0
North Macedonia	315	308	0	0	0	3	311	0	4	0	4	0	0	0	0	0
Poland																
Portugal #	2404			2130			2130			251	251			23	23	0
Slovakia †	520	78	0	0	0	424	502	14	4	0	18					0
Spain																
Tunisia, Sfax region †	161	157	0	0	0	0	157	2	2	0	4					0
Turkey																
Ukraine																

Abbreviations used: HD: haemodialysis; Unkn: unknown; HF: haemofiltration; HDF: haemodiafiltration; APD: automated peritoneal dialysis; CAPD: continuous ambulatory peritoneal dialysis; PD: peritoneal dialysis; Tx: transplant
When cells are left empty, the data are unavailable
** Patients younger than 18 years of age are not reported*
† Data include dialysis patients only
# Only preemptive transplantations (at day 1) are included

Table C.3.7

Incidence per million population by treatment modality, unadjusted

at day 91

	All	Haemodialysis						Peritoneal dialysis				Kidney transplant				Missing
		HD hospital/ centre	HD home	HD type Unkn	HF	HDF	Total HD/ HF/HDF	APD	CAPD	PD type Unkn	Total PD	Living donor	Deceased donor	Tx type Unkn	Total Tx	
	Pmp	Pmp	Pmp	Pmp	Pmp	Pmp	Pmp	Pmp	Pmp	Pmp	Pmp	Pmp	Pmp	Pmp	Pmp	Pmp
Belarus *	84.1	65.7	0	0	0.9	9.9	76.6	0.2	2.5	0	2.7	0.4	4.5	0	4.8	0
Croatia †	129.0	101.8	0	0	0	19.3	121.1			7.9	7.9					0
Cyprus	298.4	103.9	0	0	0	178.0	281.9	0	3.3	13.3	16.6	0	0	0	0	0
Czech Republic																
Finland	82.1	45.1	2.0	0	0	7.9	55.0	10.4	10.4	0	20.8	2.0	4.1	0.2	6.3	0
Hungary	163.3	122.5	0	0	3.4	23.2	149.1	0	0	12.3	12.3	1.2	0.6	0	1.9	0
Israel	167.4	137.3	0	0	0	5.4	142.7	2.8	12.1	0	15.0	9.3	0.4	0	9.7	0
Italy (8 of 20 regions)	148.2	98.6	0.3	0.1	5.5	19.8	124.3	9.8	12.2	0	21.9	1.0	1.0	0	2.0	0
Kosovo *	122.1	114.4	0	0	0	0	114.4	0	0	0	0	7.7	0	0	7.7	0
Latvia	64.1	52.7	0	0	0	0	52.7	4.2	6.6	0	10.8	0	0.6	0	0.6	0
Lithuania	79.1	69.5	0	0	0	0	69.5			6.8	6.8	0.7	2.1	0	2.9	0
North Macedonia	172.1	168.3	0	0	0	1.6	169.9	0	2.2	0	2.2	0	0	0	0	0
Poland																
Portugal #	229.7			203.5			203.5			24.0	24.0			2.2	2.2	0
Slovakia †	119.2	17.9	0	0	0	97.2	115.1	3.2	0.9	0	4.1					0
Spain																
Tunisia, Sfax region †	156.8	153.0	0	0	0	0	153.0	1.9	1.9	0	3.9					0
Turkey																
Ukraine																

Abbreviations used: HD: haemodialysis; Unkn: unknown; HF: haemofiltration; HDF: haemodiafiltration; APD: automated peritoneal dialysis; CAPD: continuous ambulatory peritoneal dialysis; PD: peritoneal dialysis; Tx: transplant
Categories may not add up because of rounding
When cells are left empty, the data are unavailable
** Patients younger than 18 years of age are not reported*
† Data include dialysis patients only
# Only preemptive transplantations (at day 1) are included

Table C.3.8
Treatment modality distribution, unadjusted
at day 91

	All	Haemodialysis						Peritoneal dialysis				Kidney transplant				Missing
		HD hospital/ centre	HD home	HD type Unkn	HF	HDF	Total HD/ HF/HDF	APD	CAPD	PD type Unkn	Total PD	Living donor	Deceased donor	Tx type Unkn	Total Tx	
	%	%	%	%	%	%	%	%	%	%	%	%	%	%	%	%
Belarus *	100	78.2	0	0	1.1	11.8	91.0	0.3	2.9	0	3.2	0.4	5.3	0	5.7	0
Croatia †	100	78.9	0	0	0	15.0	93.9			6.1	6.1					0
Cyprus	100	34.8	0	0	0	59.6	94.4	0	1.1	4.4	5.6	0	0	0	0	0
Czech Republic																
Finland	100	54.9	2.4	0	0	9.6	67.0	12.7	12.7	0	25.4	2.4	5.0	0.2	7.7	0
Hungary	100	75.0	0	0	2.1	14.2	91.3	0	0	7.5	7.5	0.8	0.4	0	1.1	0
Israel	100	82.0	0	0	0	3.3	85.3	1.7	7.3	0	8.9	5.6	0.3	0	5.8	0
Italy (8 of 20 regions)	100	66.5	0.2	0.1	3.7	13.4	83.9	6.6	8.2	0	14.8	0.7	0.6	0	1.3	0
Kosovo *	100	93.7	0	0	0	0	93.7	0	0	0	0	6.3	0	0	6.3	0
Latvia	100	82.2	0	0	0	0	82.2	6.5	10.3	0	16.8	0	0.9	0	0.9	0
Lithuania	100	87.8	0	0	0	0	87.8			8.6	8.6	0.9	2.7	0	3.6	0
North Macedonia	100	97.8	0	0	0	1.0	98.7	0	1.3	0	1.3	0	0	0	0	0
Poland																
Portugal #	100			88.6			88.6			10.4	10.4			1.0	1.0	0
Slovakia †	100	15.0	0	0	0	81.5	96.5	2.7	0.8	0	3.5					0
Spain																
Tunisia, Sfax region †	100	97.5	0	0	0	0	97.5	1.2	1.2	0	2.5					0
Turkey																
Ukraine																

Abbreviations used: HD: haemodialysis; Unkn: unknown; HF: haemofiltration; HDF: haemodiafiltration; APD: automated peritoneal dialysis; CAPD: continuous ambulatory peritoneal dialysis; PD: peritoneal dialysis; Tx: transplant
Categories may not add up because of rounding
When cells are left empty, the data are unavailable
** Patients younger than 18 years of age are not reported*
† Data include dialysis patients only
# Only preemptive transplantations (at day 1) are included

Table C.4.1

Prevalent counts and percentages by age and sex

prevalent patients on December 31

	All			0-19						20-44						45-64						65-74						75+					
	All	Male	Female	All		Male		Female		All		Male		Female		All		Male		Female		All		Male		Female		All		Male		Female	
	N (100%)	N (100%)	N (100%)	N	%	N	%	N	%	N	%	N	%	N	%	N	%	N	%	N	%	N	%	N	%	N	%	N	%	N	%	N	%
Belarus *	4108	2309	1799	19	0	11	0	8	0	1219	30	705	31	514	29	1981	48	1103	48	878	49	732	18	403	17	329	18	157	4	87	4	70	4
Croatia †	2018	1211	807	1	0	1	0	0	0	224	11	125	10	99	12	589	29	392	32	197	24	706	35	415	34	291	36	498	25	278	23	220	27
Cyprus																																	
Czech Republic	11157																																
Finland	5240	3242	1998	121	2	74	2	47	2	820	16	503	16	317	16	1975	38	1210	37	765	38	1343	26	836	26	507	25	981	19	619	19	362	18
Hungary	9485	5444	4041	125	1	75	1	50	1	1417	15	858	16	559	14	3896	41	2377	44	1519	38	2521	27	1413	26	1108	27	1526	16	721	13	805	20
Israel †	6963	4378	2585	50	1	27	1	23	1	453	7	268	6	185	7	1943	28	1242	28	701	27	2057	30	1320	30	737	29	2460	35	1521	35	939	36
Italy (8 of 20 regions)	31758	20285	11473	349	1	216	1	133	1	3741	12	2344	12	1397	12	11395	36	7271	36	4124	36	7782	25	5015	25	2767	24	8491	27	5439	27	3052	27
Kosovo *	1044	612	432	4	0	2	0	2	0	160	15	102	17	58	13	451	43	272	44	179	41	309	30	175	29	134	31	120	11	61	10	59	14
Latvia	1046	584	462	14	1	8	1	6	1	239	23	148	25	91	20	454	43	257	44	197	43	228	22	120	21	108	23	111	11	51	9	60	13
Lithuania	2318																																
North Macedonia	1731	1110	621	3	0	1	0	2	0	243	14	153	14	90	14	786	45	520	47	266	43	477	28	292	26	185	30	222	13	144	13	78	13
Poland †	20198	12302	7896	78	0					2284	11					6290	31					6467	32					5079	25				
Portugal	21198	12985	8213																														
Slovakia †	3154	1936	1218	9	0	4	0	5	0	318	10	216	11	102	8	1027	33	694	36	333	27	1047	33	630	33	417	34	753	24	392	20	361	30
Spain §	66856	42529	24193	409	1	253	1	156	1	9103	14	5606	13	3497	14	25725	38	16429	39	9296	38	16252	24	10645	25	5607	23	15233	23	9596	23	5637	23
Tunisia, Sfax region †	1043	623	420	10	1	6	1	4	1	209	20	122	20	87	21	437	42	275	44	162	39	230	22	143	23	87	21	157	15	77	12	80	19
Turkey	86665																																
Ukraine *	7625	4209	3416	123	2	60	1	63	2	2209	29	1258	30	951	28	3448	45	1943	46	1505	44	1542	20	784	19	758	22	303	4	164	4	139	4

Categories may not add up because of missing values or rounding
When cells are left empty, the data are unavailable
** Patients younger than 18 years of age are not reported*
† Data include dialysis patients only
§ Data presented in column 0-19 refer to patients aged 0-14 years and data presented in column 20-44 refer to patients aged 15-44 years

Table C.4.2
Prevalence per million (age-related) population by age and sex, unadjusted
prevalent patients on December 31

	All			0-19			20-44			45-64			65-74			75+		
	All	Male	Female	All	Male	Female	All	Male	Female	All	Male	Female	All	Male	Female	All	Male	Female
	Pmp	Pmp	Pmp	Pmarp	Pmarp	Pmarp	Pmarp	Pmarp	Pmarp	Pmarp	Pmarp	Pmarp	Pmarp	Pmarp	Pmarp	Pmarp	Pmarp	Pmarp
Belarus *	483.9	588.5	393.9	10.2	11.5	8.8	422.3	491.8	353.8	837.1	1024.4	680.7	824.8	1201.3	596.0	320.6	705.2	191.1
Croatia †	638.3	793.8	493.3	1.6	3.2	0	234.2	257.2	210.4	667.4	913.2	434.6	1687.5	2189.4	1271.8	1679.2	2566.7	1168.6
Cyprus																		
Czech Republic	1051.5																	
Finland	941.8	1177.4	710.9	105.4	126.0	83.7	470.8	558.9	376.6	1431.7	1747.1	1113.7	1936.1	2539.4	1391.1	1632.7	2530.8	1016.1
Hungary	978.9	1172.0	801.1	65.7	76.7	54.0	452.9	534.8	366.7	1461.0	1827.0	1112.3	2143.5	2871.0	1620.0	1874.1	2672.9	1478.5
Israel †	728.6	921.9	537.6	14.6	15.4	13.8	144.7	170.1	119.0	1061.6	1389.7	748.5	2989.8	4135.3	1998.4	4979.8	7312.5	3283.2
Italy (8 of 20 regions)	1165.0	1524.2	822.3	73.0	87.7	57.3	493.4	606.6	375.8	1355.3	1758.3	965.3	2443.5	3330.6	1647.9	2569.7	4057.4	1554.1
Kosovo *	618.6	720.3	515.5	6.2	6.0	6.5	247.5	315.5	179.6	1580.4	1933.5	1237.1	4091.2	4790.6	3436.2	3178.4	3714.1	2766.0
Latvia	626.3	755.0	515.3	40.0	44.4	35.4	470.8	567.8	368.5	979.2	1188.6	796.3	1230.1	1681.2	947.5	677.6	1131.7	505.2
Lithuania	826.1																	
North Macedonia	945.9	1223.8	672.9	7.3	4.7	10.1	408.3	505.9	307.5	1568.9	2110.9	1044.6	2290.9	2986.5	1675.0	1928.8	2943.9	1178.6
Poland †	534.0	672.9	404.0	10.2			175.4			636.5			1430.4			1857.8		
Portugal	2025.2	2596.1	1502.7															
Slovakia †	723.1	907.6	546.5	9.9	8.6	11.2	213.6	282.5	140.8	861.3	1172.2	554.7	2148.6	2948.9	1523.8	2678.0	4089.5	1947.9
Spain §	1408.2	1828.0	999.3	45.3	54.4	35.6	621.7	757.8	482.7	1796.6	2312.0	1288.9	3404.5	4750.7	2213.6	3237.4	5128.9	1988.8
Tunisia, Sfax region †	1016.1	1213.2	818.8	31.1	36.2	25.7	570.6	672.8	470.3	1843.2	2327.9	1361.8	3385.1	4393.5	2457.8	4654.0	4875.8	4458.9
Turkey	1016.2																	
Ukraine *	369.3	439.7	308.5	30.2	28.6	31.9	307.8	346.4	268.3	599.8	743.8	479.8	711.7	968.3	558.6	205.5	393.5	131.4

When cells are left empty, the data are unavailable
** Patients younger than 18 years of age are not reported*
† Data include dialysis patients only
§ Data presented in column 0-19 refer to patients aged 0-14 years and data presented in column 20-44 refer to patients aged 15-44 years

Table C.4.3

Sex, mean age, and median age

prevalent patients on December 31

	All						Male						Female					
	%	Mean (years)	SD	Median (years)	P25	P75	%	Mean (years)	SD	Median (years)	P25	P75	%	Mean (years)	SD	Median (years)	P25	P75
Belarus *	100						56.2						43.8					
Croatia †	100	66.8	14.2	70.0	59.0	79.0	60.0	66.1	14.1	67.0	57.0	78.0	40.0	67.9	14.9	68.7	60.0	79.0
Cyprus																		
Czech Republic																		
Finland	100	60.1	16.5	62.6	50.8	72.6	61.9	60.3	16.5	62.7	50.7	72.9	38.1	59.8	16.7	62.6	51.0	72.1
Hungary	100	59.4	15.4	61.0	49.0	71.0	57.4	58.4	15.1	60.0	48.0	69.0	42.6	60.9	15.7	63.0	50.0	73.0
Israel †	100	68.0	14.5	70.4	60.4	78.0	62.9	68.1	14.2	70.2	60.5	77.9	37.1	67.9	15.0	70.8	60.3	78.0
Italy (8 of 20 regions)	100	63.6	16.3	65.6	53.5	75.8	63.9	63.7	16.2	65.7	53.7	75.8	36.1	63.5	16.4	65.3	53.0	75.7
Kosovo *	100	59.3	14.0	62.0	51.0	70.0	58.6	58.0	14.1	61.0	49.0	75.0	41.4	61.0	13.8	63.0	54.0	71.3
Latvia	100	56.1	15.9	58.0	45.0	68.0	55.8	54.7	15.5	56.0	43.0	67.0	44.2	57.9	16.1	60.0	47.0	70.0
Lithuania																		
North Macedonia	100	59.8	13.7	62.0	51.0	69.0	64.1	59.7	13.5	61.0	51.0	69.0	35.9	60.0	13.8	62.0	51.0	70.5
Poland †	100						60.9						39.1					
Portugal	100	67.6					61.3						38.7					
Slovakia †	100	63.9	14.2	66.0	56.0	74.0	61.4	62.6	14.1	65.0	54.0	73.0	38.6	65.2	14.9	68.0	58.0	76.0
Spain	100	60.1	15.7	63.5	50.6	74.6	63.6	60.4	15.6	63.8	50.8	74.5	36.2	59.9	16.0	63.2	50.2	74.7
Tunisia, Sfax region †	100	58.0	15.7	60.0	47.0	69.0	59.7	57.5	15.5	59.0	47.0	68.0	40.3	58.7	16.5	61.0	46.0	71.0
Turkey																		
Ukraine *	100	53.0	13.7	54.0	42.0	63.0	55.2	52.8	13.6	54.0	43.0	63.0	44.8	53.3	13.9	55.0	42.0	64.0

Categories may not add up because of missing values or rounding
When cells are left empty, the data are unavailable
** Patients younger than 18 years of age are not reported*
† Data include dialysis patients only

Table C.4.4

Prevalence per million population, adjusted

prevalent patients on December 31, adjusted for age and sex

	All	Male	Female
	Pmp	Pmp	Pmp
Belarus			
Croatia †	612.9	769.4	463.3
Cyprus			
Czech Republic			
Finland	932.0	1163.3	711.0
Hungary	1008.3	1234.7	791.8
Israel †	1185.3	1506.0	878.7
Italy (8 of 20 regions)	1071.9	1389.9	767.9
Kosovo *	1313.1	1501.3	1133.1
Latvia	640.7	789.5	498.4
Lithuania			
North Macedonia	1000.0	1306.6	706.9
Poland			
Portugal			
Slovakia †	822.7	1062.5	593.4
Spain			
Tunisia, Sfax region †	1378.4	1624.7	1142.9
Turkey			
Ukraine			

When cells are left empty, the data are unavailable
** Patients younger than 18 years of age are not reported*
† Data include dialysis patients only

Table C.4.5

Prevalence per million population and percentages by primary renal disease (1995 PRD codes), unadjusted

prevalent patients on December 31

	All		GN		PN		PKD		DM						HT		RVD		Misc		Unkn		Missing	
									Type 1		Type 2		Both											
	Pmp	%	Pmp	%	Pmp	%	Pmp	%	Pmp	%	Pmp	%	Pmp	%	Pmp	%	Pmp	%	Pmp	%	Pmp	%	Pmp	%
Belarus *	483.9	100	209.7	43.3	33.2	6.9	49.9	10.3	24.3	5.0	30.0	6.2	54.3	11.2	39.9	8.3	11.7	2.4	83.5	17.3	1.4	0.3	0.2	0
Croatia †	638.3	100	126.5	19.8	41.4	6.5	69.6	10.9	14.2	2.2	148.0	23.2	162.3	25.4	127.5	20.0	18.7	2.9	58.8	9.2	23.1	3.6	10.4	1.6
Cyprus																								
Czech Republic																								
Finland	941.8	100	200.6	21.3	40.3	4.3	140.7	14.9	143.4	15.2	82.3	8.7	225.7	24.0	43.9	4.7	5.6	0.6	187.1	19.9	93.8	10.0	4.1	0.4
Hungary	978.9	100	140.6	14.4	90.4	9.2	88.0	9.0	93.2	9.5	109.5	11.2	202.7	20.7	37.5	3.8	1.3	0.1	7.0	0.7	11.2	1.1	400.1	40.9
Israel †	728.6	100	54.4	7.5	15.1	2.1	24.1	3.3	27.4	3.8	308.8	42.4	336.2	46.1	85.4	11.7	8.7	1.2	80.3	11.0	119.6	16.4	4.9	0.7
Italy (8 of 20 regions)	1165.0	100	212.3	18.2	71.8	6.2	110.7	9.5					122.1	10.5	121.1	10.4	30.0	2.6	116.0	10.0	286.9	24.6	94.2	8.1
Kosovo *	618.6	100	49.2	8.0	197.9	32.0	35.0	5.7	13.0	2.1	155.2	25.1	168.3	27.2	101.9	16.5	18.4	3.0	31.4	5.1	16.6	2.7	0	0
Latvia	626.3	100	186.2	29.7	71.9	11.5	90.4	14.4	37.1	5.9	36.5	5.8	73.7	11.8	58.1	9.3	3.0	0.5	122.2	19.5	21.0	3.3	0	0
Lithuania																								
North Macedonia	945.9	100	109.8	11.6	55.7	5.9	91.8	9.7	12.0	1.3	156.8	16.6	168.9	17.9	270.5	28.6	6.0	0.6	89.6	9.5	153.6	16.2	0	0
Poland †	534.0	100	71.7	13.4	30.8	5.8	38.5	7.2	20.5	3.8	96.8	18.1	117.2	22.0	62.2	11.7	21.4	4.0	68.9	12.9	123.3	23.1	0	0
Portugal ‡ + &	2025.2	100	183.1	9.0			87.2	4.3					358.6	17.7	163.7	8.1			273.6	13.5	245.8	12.1	2.4	0.1
Slovakia †	723.1	100	95.1	13.2	91.9	12.7	37.1	5.1					195.6	27.0	103.9	14.4	50.9	7.0	97.7	13.5	50.9	7.0	0	0
Spain ¶ §	1408.2	100	306.7	21.8									233.8	16.6	154.4	11.0			666.9	47.4	43.6	3.1	2.8	0.2
Tunisia, Sfax region † $	1016.1	100	124.7	12.3	175.4	17.3	81.8	8.1					214.3	21.1			42.9	4.2	23.4	2.3	285.4	28.1	68.2	6.7
Turkey	1016.2	100	9.9	1.0	1.5	0.2	8.6	0.8					72.6	7.1	65.2	6.4	1.7	0.2	21.7	2.1	24.2	2.4	810.9	79.8
Ukraine * +	369.3	100	113.5	30.7	53.3	14.4	42.7	11.6	24.8	6.7	37.3	10.1	62.1	16.8	30.0	8.1			55.8	15.1	4.1	1.1	7.7	2.1

Abbreviations used: GN: glomerulonephritis/sclerosis; PN: pyelonephritis; PKD: polycystic kidneys, adult type; DM: diabetes mellitus; HT: hypertension; RVD: renal vascular disease; Misc: miscellaneous; Unkn: unknown
Categories may not add up because of rounding
When cells are left empty, the data are unavailable
** Patients younger than 18 years of age are not reported*
† Data include dialysis patients only
‡ Data on primary renal disease are available for dialysis patients only (N=13759, 65.0% of total)
+ Renal vascular disease is not reported separately, but is included in miscellaneous
& Pyelonephritis is not reported separately, but is included in miscellaneous
¶ Renal vascular disease is not reported separately, but is included in hypertension
§ Miscellaneous renal disorders include: Tubulointerstitial disease (n=7696); Other systemic diseases affecting the kidney (n=1272), Familial/Hereditary nephropathies (n=9153), and Miscellaneous renal disorders (n=13541)
$ Hypertension is not reported separately, but is included in renal vascular disease

Table C.4.6
Prevalent counts by treatment modality
prevalent patients on December 31

	All	Haemodialysis						Peritoneal dialysis				Kidney transplant				Missing
		HD hospital/ centre	HD home	HD type Unkn	HF	HDF	Total HD/ HF/HDF	APD	CAPD	PD type Unkn	Total PD	Living donor	Deceased donor	Tx type Unkn	Total Tx	
	N	N	N	N	N	N	N	N	N	N	N	N	N	N	N	N
Belarus *	4108	1382	0	0	0	354	1736	6	113	0	119	94	2159	0	2253	0
Croatia †	2018	1598	0	0	0	280	1878			140	140					0
Cyprus																
Czech Republic	11157	1413	53	0	0	4642	6108			267	267			4779	4779	3
Finland	5240	754	107	0	0	678	1539	170	140	0	310	315	2750	326	3391	0
Hungary	9485	4898	0	0	12	544	5454			728	728			3282	3282	21
Israel †	6963	5911	0	0	0	486	6397	199	366	1	566					0
Italy (8 of 20 regions)	31758	12633	48	1	678	5062	18422	1161	971	7	2139	1156	10039	2	11197	0
Kosovo *	1044	894	0	0	0	0	894	0	0	0	0	150	0	0	150	0
Latvia	1046	409	0	0	0	0	409	50	44	0	94	73	466	4	543	0
Lithuania	2318	1164	0	0	0	0	1164	35	23	0	58			1096	1096	0
North Macedonia	1731	1313	0	0	0	164	1477	5	8	0	13	200	41	0	241	0
Poland †	20198	19389	0	0	0	0	19389			809	809					0
Portugal	21198			12878			12878			881	881	820	6619	0	7439	0
Slovakia †	3154	317	0	0	41	2743	3101	35	18	0	53					0
Spain	66856			26549			26549			3209	3209			37098	37098	0
Tunisia, Sfax region †	1043	1020	0	0	0	0	1020	8	15	0	23					0
Turkey	86665	60101	1257	283	6	76	61723	1248	2304	0	3552			21390	21390	0
Ukraine *	7625	4477	0	0	0	1659	6136	100	306	0	406	539	526	18	1083	0

Abbreviations used: HD: haemodialysis; Unkn: unknown; HF: haemofiltration; HDF: haemodiafiltration; APD: automated peritoneal dialysis; CAPD: continuous ambulatory peritoneal dialysis; PD: peritoneal dialysis; Tx: transplant
When cells are left empty, the data are unavailable
** Patients younger than 18 years of age are not reported*
† Data include dialysis patients only

Table C.4.7
Prevalence per million population by treatment modality, unadjusted
prevalent patients on December 31

	All	Haemodialysis						Peritoneal dialysis				Kidney transplant				Missing
		HD hospital/ centre	HD home	HD type Unkn	HF	HDF	Total HD/ HF/HDF	APD	CAPD	PD type Unkn	Total PD	Living donor	Deceased donor	Tx type Unkn	Total Tx	
	Pmp	Pmp	Pmp	Pmp	Pmp	Pmp	Pmp	Pmp	Pmp	Pmp	Pmp	Pmp	Pmp	Pmp	Pmp	Pmp
Belarus *	483.9	162.8	0	0	0	41.7	204.5	0.7	13.3	0	14.0	11.1	254.3	0	265.4	0
Croatia †	638.3	505.4	0	0	0	88.6	594.0			44.3	44.3					0
Cyprus																
Czech Republic	1051.5	133.2	5.0	0	0	437.5	575.6			25.2	25.2			450.4	450.4	0.3
Finland	941.8	135.5	19.2	0	0	121.9	276.6	30.6	25.2	0	55.7	56.6	494.3	58.6	609.5	0
Hungary	978.9	505.5	0	0	1.2	56.1	562.9			75.1	75.1			338.7	338.7	2.2
Israel †	728.6	618.5	0	0	0	50.9	669.3	20.8	38.3	0.1	59.2					0
Italy (8 of 20 regions)	1165.0	463.4	1.8	0	24.9	185.7	675.8	42.6	35.6	0.3	78.5	42.4	368.3	0.1	410.7	0
Kosovo *	618.6	529.7	0	0	0	0	529.7	0	0	0	0	88.9	0	0	88.9	0
Latvia	626.3	244.9	0	0	0	0	244.9	29.9	26.3	0	56.3	43.7	279.0	2.4	325.1	0
Lithuania	826.1	414.8	0	0	0	0	414.8	12.5	8.2	0	20.7			390.6	390.6	0
North Macedonia	945.9	717.5	0	0	0	89.6	807.1	2.7	4.4	0	7.1	109.3	22.4	0	131.7	0
Poland †	534.0	512.6	0	0	0	0	512.6			21.4	21.4					0
Portugal	2025.2			1230.3			1230.3			84.2	84.2	78.3	632.3	0	710.7	0
Slovakia †	723.1	72.7	0	0	9.4	628.9	711.0	8.0	4.1	0	12.2					0
Spain	1408.2			559.2			559.2			67.6	67.6			781.4	781.4	0
Tunisia, Sfax region †	1016.1	993.7	0	0	0	0	993.7	7.8	14.6	0	22.4					0
Turkey	1016.2	704.8	14.7	3.3	0.1	0.9	723.8	14.6	27.0	0	41.7			250.8	250.8	0
Ukraine *	369.3	216.8	0	0	0	80.4	297.2	4.8	14.8	0	19.7	26.1	25.5	0.9	52.5	0

Abbreviations used: HD: haemodialysis; Unkn: unknown; HF: haemofiltration; HDF: haemodiafiltration; APD: automated peritoneal dialysis; CAPD: continuous ambulatory peritoneal dialysis; PD: peritoneal dialysis; Tx: transplant
Categories may not add up because of rounding
When cells are left empty, the data are unavailable
** Patients younger than 18 years of age are not reported*
† Data include dialysis patients only

Table C.4.8

Treatment modality distribution, unadjusted

prevalent patients on December 31

	All	Haemodialysis						Peritoneal dialysis				Kidney transplant				Missing
		HD hospital/ centre	HD home	HD type Unkn	HF	HDF	Total HD/ HF/HDF	APD	CAPD	PD type Unkn	Total PD	Living donor	Deceased donor	Tx type Unkn	Total Tx	
	%	%	%	%	%	%	%	%	%	%	%	%	%	%	%	%
Belarus *	100	33.6	0	0	0	8.6	42.3	0.1	2.8	0	2.9	2.3	52.6	0	54.8	0
Croatia †	100	79.2	0	0	0	13.9	93.1			6.9	6.9					0
Cyprus																
Czech Republic	100	12.7	0.5	0	0	41.6	54.7			2.4	2.4			42.8	42.8	0
Finland	100	14.4	2.0	0	0	12.9	29.4	3.2	2.7	0	5.9	6.0	52.5	6.2	64.7	0
Hungary	100	51.6	0	0	0.1	5.7	57.5			7.7	7.7			34.6	34.6	0.2
Israel †	100	84.9	0	0	0	7.0	91.9	2.9	5.3	0	8.1					0
Italy (8 of 20 regions)	100	39.8	0.2	0	2.1	15.9	58.0	3.7	3.1	0	6.7	3.6	31.6	0	35.3	0
Kosovo *	100	85.6	0	0	0	0	85.6	0	0	0	0	14.4	0	0	14.4	0
Latvia	100	39.1	0	0	0	0	39.1	4.8	4.2	0	9.0	7.0	44.6	0.4	51.9	0
Lithuania	100	50.2	0	0	0	0	50.2	1.5	1.0	0	2.5			47.3	47.3	0
North Macedonia	100	75.9	0	0	0	9.5	85.3	0.3	0.5	0	0.8	11.6	2.4	0	13.9	0
Poland †	100	96.0	0	0	0	0	96.0			4.0	4.0					0
Portugal	100			60.8			60.8			4.2	4.2	3.9	31.2	0	35.1	0
Slovakia †	100	10.1	0	0	1.3	87.0	98.3	1.1	0.6	0	1.7					0
Spain	100			39.7			39.7			4.8	4.8			55.5	55.5	0
Tunisia, Sfax region †	100	97.8	0	0	0	0	97.8	0.8	1.4	0	2.2					0
Turkey	100	69.3	1.5	0.3	0	0.1	71.2	1.4	2.7	0	4.1			24.7	24.7	0
Ukraine *	100	58.7	0	0	0	21.8	80.5	1.3	4.0	0	5.3	7.1	6.9	0.2	14.2	0

Abbreviations used: HD: haemodialysis; Unkn: unknown; HF: haemofiltration; HDF: haemodiafiltration; APD: automated peritoneal dialysis; CAPD: continuous ambulatory peritoneal dialysis; PD: peritoneal dialysis; Tx: transplant
Categories may not add up because of rounding
When cells are left empty, the data are unavailable
** Patients younger than 18 years of age are not reported*
† Data include dialysis patients only

Table C.5.1
Kidney transplant counts and percentages by donor type

	All		Living donor								Deceased donor		Donor type Unkn	
			Related		Unrelated		Type Unkn		All					
	N	%	N	%	N	%	N	%	N	%	N	%	N	%
Belarus *	307	100	0	0	0	0	5	1.6	5	1.6	302	98.4	0	0
Croatia	112	100											112	100
Cyprus	16	100	4	25.0	4	25.0	0	0	8	50.0	8	50.0	0	0
Czech Republic	520	100					40	7.7	40	7.7	480	92.3	0	0
Finland	250	100	31	12.4	11	4.4	0	0	42	16.8	208	83.2	0	0
Hungary	244	100					56	23.0	56	23.0	188	77.0	0	0
Israel	498	100					339	68.1	339	68.1	149	29.9	10	2.0
Italy (8 of 20 regions)	685	100	31	4.5	77	11.2	0	0	108	15.8	538	78.5	39	5.7
Kosovo	16	100	15	93.8	1	6.3	0	0	16	100	0	0	0	0
Latvia	43	100	3	7.0	0	0	0	0	3	7.0	40	93.0	0	0
Lithuania	82	100					5	6.1	5	6.1	77	93.9	0	0
North Macedonia	22	100	12	54.5	0	0	0	0	12	54.5	10	45.5	0	0
Poland	874	100					73	8.4	73	8.4	801	91.6	0	0
Portugal	487	100					58	11.9	58	11.9	429	88.1	0	0
Slovakia +	153	100	18	11.8	12	7.8	0	0	30	19.6	123	80.4	0	0
Spain ¤	3404	100	326	9.6	24	0.7			350	10.3	3054	89.7	0	0
Tunisia, Sfax region	13	100	6	46.2	7	53.8	0	0	13	100	0	0	0	0
Turkey	3621	100	2774	76.6	566	15.6	0	0	3340	92.2	281	7.8	0	0
Ukraine *	196	100	82	41.8	3	1.5	0	0	85	43.4	111	56.6	0	0

Categories may not add up because of rounding
When cells are left empty, the data are unavailable
** Patients younger than 18 years of age are not reported*
+ Data from: National Transplant Organization (NTO)
¤ Data from: Organización Nacional de Trasplantes (ONT)

Table C.5.2
Kidney transplants per million population by donor type, unadjusted

	All	Living donor				Deceased donor	Donor type Unkn
		Related	Unrelated	Type Unkn	All		
	Pmp	Pmp	Pmp	Pmp	Pmp	Pmp	Pmp
Belarus *	36.2	0	0	0.6	0.6	35.6	0
Croatia	35.4						35.4
Cyprus	17.7	4.4	4.4	0	8.8	8.8	0
Czech Republic	49.0			3.8	3.8	45.2	0
Finland	44.9	5.6	2.0	0	7.5	37.4	0
Hungary	25.2			5.8	5.8	19.4	0
Israel	52.1			35.5	35.5	15.6	1.0
Italy (8 of 20 regions)	25.1	1.1	2.8	0	4.0	19.7	1.4
Kosovo	9.5	8.9	0.6	0	9.5	0	0
Latvia	25.7	1.8	0	0	1.8	24.0	0
Lithuania	29.2			1.8	1.8	27.4	0
North Macedonia	12.0	6.6	0	0	6.6	5.5	0
Poland	23.1			1.9	1.9	21.2	0
Portugal	46.5			5.5	5.5	41.0	0
Slovakia +	35.1	4.1	2.8	0	6.9	28.2	0
Spain ¤	71.7	6.9	0.5		7.4	64.3	0
Tunisia, Sfax region	12.7	5.8	6.8	0	12.7	0	0
Turkey	42.5	32.5	6.6	0	39.2	3.3	0
Ukraine *	9.5	4.0	0.1	0	4.1	5.4	0

Categories may not add up because of rounding
When cells are left empty, the data are unavailable
** Patients younger than 18 years of age are not reported*
+ Data from: National Transplant Organization (NTO)
¤ Data from: Organización Nacional de Trasplantes (ONT)

Section D: Paediatric data reference tables

Section D: Paediatric data reference tables

The incidence and prevalence of KRT in paediatric patients are based on data from 27 registries from 15 countries that provided individual patient data on children to the ERA Registry, including Austria, Bosnia and Herzegovina, Denmark, Estonia, France, Greece, Iceland, Norway, Romania, Serbia, Spain (Andalusia), Spain (Aragon), Spain (Asturias), Spain (Basque country), Spain (Canary Islands), Spain (Catalonia), Spain (Community of Madrid), Spain (Extremadura), Spain (Galicia), Spain (La Rioja), Spain (Murcia), Spain (Valencian Region), Sweden, Switzerland, the Netherlands, United Kingdom (England/Northern Ireland/Wales), and United Kingdom (Scotland). In the analyses the data collected between 2017 and 2022 were used. Since not all countries provided data or had full coverage over the entire period and to allow for comparison across years, data are shown both including and excluding the data from Austria, Romania, Serbia, Spain (Community of Madrid), Spain (La Rioja), Switzerland, and the Netherlands. The methods applied to the paediatric patient data were similar to those applied to the adult patient data and are described in the Methods section. However, as not all regions in Spain had specialized paediatric centres, non-residents were also included in the incidence and prevalence.

D1 GENERAL POPULATION AGE DISTRIBUTION

Table D.1.1

Population covered (in thousands)

by age and cohort

Cohort	All	0-4	5-9	10-14	15-19
	N	N	N	N	N
2017	46448	11225	12086	11648	11490
2018	46492	11085	12044	11853	11510
2019	46568	10943	11979	12057	11590
2020	46533	10740	11885	12222	11685
2021	46012	10385	11557	12229	11842
2022	46000	10251	11446	12291	12012
2017 *	60045	14485	15456	15080	15024
2018 *	60161	14383	15416	15305	15056
2019 *	60298	14261	15344	15545	15148
2020 *	60118	14020	15202	15686	15210
2021 *	59742	13664	14928	15752	15398
2022 *	59723	13475	14845	15823	15581
2017-2022 *	360089	84289	91191	93192	91417

** Additionally including data from Austria (coverage 99% in 2020-2021, 98% in 2022, and 100% for all other years), Romania (coverage 97% in 2017, 98% in 2018, 99% in 2019, 95% in 2020, and 100% in 2021-2022), Serbia (97% in 2017, 90% in 2018-2020, and 95% in 2021-2022), Spain (Community of Madrid) (coverage 95% in 2022), Spain (la Rioja) since 2019, Switzerland (coverage 97% in 2021, 99% in 2022, and 100% in all other years), and the Netherlands (coverage 93% for incidence in 2017-2019, 97% for prevalence in 2017, 96% for prevalence in 2018-2022 and 94% for the incidence in 2020, and 92% for incidence in 2021-2022)*

Table D.2.1
Incident counts by age and cohort
at day 1

Cohort	All	0-4	5-9	10-14	15-19
	N	N	N	N	N
2017-2018	883	191	126	207	359
2019-2020	828	146	125	197	360
2021-2022	836	138	115	220	363
2017-2018 *	1070	217	155	249	449
2019-2020 *	1012	168	154	234	456
2021-2022 *	1029	169	148	267	445
2017-2022 *	3111	554	457	750	1350

** Additionally including data from Austria (coverage 99% in 2020-2021, 98% in 2022, and 100% for all other years), Romania (coverage 97% in 2017, 98% in 2018, 99% in 2019, 95% in 2020, and 100% in 2021-2022), Serbia (97% in 2017, 90% in 2018-2020, and 95% in 2021-2022), Spain (Community of Madrid) (coverage 95% in 2022), Spain (la Rioja) since 2019, Switzerland (coverage 97% in 2021, 99% in 2022, and 100% in all other years), and the Netherlands (coverage 93% in 2017-2019, 94% in 2020, and 92% in 2021-2022)*

Table D.2.2
Incident counts by age, treatment modality, and cohort
at day 1

Cohort	All			0-4			5-9			10-14			15-19		
	HD	PD	Tx	HD	PD	Tx	HD	PD	Tx	HD	PD	Tx	HD	PD	Tx
	N	N	N	N	N	N	N	N	N	N	N	N	N	N	N
2017-2018	399	266	208	56	106	26	49	35	40	105	54	46	189	71	96
2019-2020	392	243	186	47	76	22	53	32	37	86	64	47	206	71	80
2021-2022	397	255	180	52	72	14	45	37	31	93	62	64	207	84	71
2017-2018 *	505	298	257	65	118	31	62	41	50	132	61	54	246	78	122
2019-2020 *	505	279	221	55	88	24	68	40	43	110	70	54	272	81	100
2021-2022 *	500	307	217	59	90	20	58	51	37	120	70	76	263	96	84
2017-2022 *	1510	884	695	179	296	75	188	132	130	362	201	184	781	255	306

For 22 patients information on treatment modality at start was unavailable
** Additionally including data from Austria (coverage 99% in 2020-2021, 98% in 2022, and 100% for all other years), Romania (coverage 97% in 2017, 98% in 2018, 99% in 2019, 95% in 2020, and 100% in 2021-2022), Serbia (97% in 2017, 90% in 2018-2020, and 95% in 2021-2022), Spain (Community of Madrid) (coverage 95% in 2022), Spain (la Rioja) since 2019, Switzerland (coverage 97% in 2021, 99% in 2022, and 100% in all other years), and the Netherlands (coverage 93% in 2017-2019, 94% in 2020, and 92% in 2021-2022)*

Table D.2.3
Incident counts by age and primary renal disease
between 2017 and 2022, at day 1

	All	0-4	5-9	10-14	15-19
	N	N	N	N	N
CAKUT	907	194	163	235	315
Glomerulonephritis	513	79	58	92	284
Cystic kidney disease	315	72	55	100	88
Hereditary Nephropathy	249	53	36	45	115
Ischaemic Renal Failure	51	14	4	13	20
HUS	93	23	24	22	24
Metabolic disorders	69	14	9	20	26
Vasculitis	89		10	15	64
Miscellaneous	246	46	18	48	134
Missing, unknown	579	59	80	160	280

Abbreviations used: CAKUT: congenital anomalies of the kidney and urinary tract; HUS: haemolytic uraemic syndrome
Additionally including data from Austria (coverage 99% in 2020-2021, 98% in 2022, and 100% for all other years), Romania (coverage 97% in 2017, 98% in 2018, 99% in 2019, 95% in 2020, and 100% in 2021-2022), Serbia (97% in 2017, 90% in 2018-2020, and 95% in 2021-2022), Spain (Community of Madrid) (coverage 95% in 2022), Spain (la Rioja) since 2019, Switzerland (coverage 97% in 2021, 99% in 2022, and 100% in all other years), and the Netherlands (coverage 93% in 2017-2019, 94% in 2020, and 92% in 2021-2022)

Table D.2.4

Incidence per million age-related population by age and cohort

at day 1

Cohort	All	0-4	5-9	10-14	15-19
	Pmarp	Pmarp	Pmarp	Pmarp	Pmarp
2017-2018	9.5	8.6	5.2	8.8	15.6
2019-2020	8.9	6.7	5.2	8.1	15.5
2021-2022	9.1	6.7	5.0	9.0	15.2
2017-2018 *	8.9	7.5	5.0	8.2	14.9
2019-2020 *	8.4	5.9	5.0	7.5	15.0
2021-2022 *	8.6	6.2	5.0	8.5	14.4
2017-2022 *	8.6	6.6	5.0	8.0	14.8

** Additionally including data from Austria (coverage 99% in 2020-2021, 98% in 2022, and 100% for all other years), Romania (coverage 97% in 2017, 98% in 2018, 99% in 2019, 95% in 2020, and 100% in 2021-2022), Serbia (97% in 2017, 90% in 2018-2020, and 95% in 2021-2022), Spain (Community of Madrid) (coverage 95% in 2022), Spain (la Rioja) since 2019, Switzerland (coverage 97% in 2021, 99% in 2022, and 100% in all other years), and the Netherlands (coverage 93% in 2017-2019, 94% in 2020, and 92% in 2021-2022)*

Table D.2.5

Incidence per million age-related population by age, treatment modality, and cohort

at day 1

Cohort	All			0-4			5-9			10-14			15-19		
	HD	PD	Tx	HD	PD	Tx	HD	PD	Tx	HD	PD	Tx	HD	PD	Tx
	Pmarp	Pmarp	Pmarp	Pmarp	Pmarp	Pmarp	Pmarp	Pmarp	Pmarp	Pmarp	Pmarp	Pmarp	Pmarp	Pmarp	Pmarp
2017-2018	4.3	2.9	2.2	2.5	4.8	1.2	2.0	1.5	1.7	4.5	2.3	2.0	8.2	3.1	4.2
2019-2020	4.2	2.6	2.0	2.2	3.5	1.0	2.2	1.3	1.6	3.5	2.6	1.9	8.9	3.1	3.4
2021-2022	4.3	2.8	2.0	2.5	3.5	0.7	2.0	1.6	1.3	3.8	2.5	2.6	8.7	3.5	3.0
2017-2018 *	4.2	2.5	2.1	2.3	4.1	1.1	2.0	1.3	1.6	4.3	2.0	1.8	8.2	2.6	4.1
2019-2020 *	4.2	2.3	1.8	1.9	3.1	0.8	2.2	1.3	1.4	3.5	2.2	1.7	9.0	2.7	3.3
2021-2022 *	4.2	2.6	1.8	2.2	3.3	0.7	1.9	1.7	1.2	3.8	2.2	2.4	8.5	3.1	2.7
2017-2022 *	4.2	2.5	1.9	2.1	3.5	0.9	2.1	1.4	1.4	3.9	2.2	2.0	8.5	2.8	3.3

For 22 patients information on treatment modality at start was unavailable

** Additionally including data from Austria (coverage 99% in 2020-2021, 98% in 2022, and 100% for all other years), Romania (coverage 97% in 2017, 98% in 2018, 99% in 2019, 95% in 2020, and 100% in 2021-2022), Serbia (97% in 2017, 90% in 2018-2020, and 95% in 2021-2022), Spain (Community of Madrid) (coverage 95% in 2022), Spain (la Rioja) since 2019, Switzerland (coverage 97% in 2021, 99% in 2022, and 100% in all other years), and the Netherlands (coverage 93% in 2017-2019, 94% in 2020, and 92% in 2021-2022)*

Table D.2.6

Incidence per million age-related population by age and primary renal disease

between 2017 and 2022, at day 1

	All	0-4	5-9	10-14	15-19
	Pmarp	Pmarp	Pmarp	Pmarp	Pmarp
CAKUT	2.5	2.3	1.8	2.5	3.4
Glomerulonephritis	1.4	0.9	0.6	1.0	3.1
Cystic kidney disease	0.9	0.9	0.6	1.1	1.0
Hereditary Nephropathy	0.7	0.6	0.4	0.5	1.3
Ischaemic Renal Failure	0.1	0.2	0.0	0.1	0.2
HUS	0.3	0.3	0.3	0.2	0.3
Metabolic disorders	0.2	0.2	0.1	0.2	0.3
Vasculitis	0.2		0.1	0.2	0.7
Miscellaneous	0.7	0.5	0.2	0.5	1.5
Missing, unknown	1.6	0.7	0.9	1.7	3.1

Abbreviations used: CAKUT: congenital anomalies of the kidney and urinary tract; HUS: haemolytic uraemic syndrome

Additionally including data from Austria (coverage 99% in 2020-2021, 98% in 2022, and 100% for all other years), Romania (coverage 97% in 2017, 98% in 2018, 99% in 2019, 95% in 2020, and 100% in 2021-2022), Serbia (97% in 2017, 90% in 2018-2020, and 95% in 2021-2022), Spain (Community of Madrid) (coverage 95% in 2022), Spain (la Rioja) since 2019, Switzerland (coverage 97% in 2021, 99% in 2022, and 100% in all other years), and the Netherlands (coverage 93% in 2017-2019, 94% in 2020, and 92% in 2021-2022)

Table D.3.1
Prevalent counts by age and cohort
prevalent patients on December 31

Cohort	All	0-4	5-9	10-14	15-19
	N	N	N	N	N
2017	3192	250	573	894	1475
2018	3212	266	559	908	1479
2019	3224	244	564	914	1502
2020	3251	222	554	942	1533
2021	3250	219	521	967	1543
2022	3226	194	521	982	1529
2017 *	3779	287	680	1044	1768
2018 *	3796	308	658	1065	1765
2019 *	3797	277	654	1095	1771
2020 *	3834	253	647	1119	1815
2021 *	3835	258	609	1158	1810
2022 *	3895	244	619	1189	1843

** Additionally including data from Austria (coverage 99% in 2020-2021, 98% in 2022, and 100% for all other years), Romania (coverage 97% in 2017, 98% in 2018, 99% in 2019, 95% in 2020, and 100% in 2021-2022), Serbia (97% in 2017, 90% in 2018-2020, and 95% in 2021-2022), Spain (Community of Madrid) (coverage 95% in 2022), Spain (la Rioja) since 2019, Switzerland (coverage 97% in 2021, 99% in 2022, and 100% in all other years), and the Netherlands (coverage 97% in 2017, and 96% in 2018-2022)*

Table D.3.2
Prevalent counts by age, treatment modality, and cohort
prevalent patients on December 31

Cohort	All			0-4			5-9			10-14			15-19		
	HD	PD	Tx	HD	PD	Tx	HD	PD	Tx	HD	PD	Tx	HD	PD	Tx
	N	N	N	N	N	N	N	N	N	N	N	N	N	N	N
2017	434	255	2449	64	91	92	65	43	460	95	54	734	210	67	1163
2018	411	272	2477	55	103	104	57	41	458	97	59	741	202	69	1174
2019	426	263	2498	52	88	102	64	47	449	104	60	743	206	68	1204
2020	449	263	2507	56	77	88	61	42	448	98	62	770	234	82	1201
2021	448	259	2511	51	77	90	67	37	411	94	62	805	236	83	1205
2022	455	234	2521	54	58	82	61	36	423	98	59	819	242	81	1197
2017 *	555	300	2869	74	98	112	76	53	546	130	61	842	275	88	1369
2018 *	544	317	2882	62	114	128	73	51	531	134	69	851	275	83	1372
2019 *	564	303	2893	61	98	116	80	51	519	145	74	869	278	80	1389
2020 *	587	310	2905	62	87	103	78	53	513	134	73	900	313	97	1389
2021 *	578	320	2904	63	90	104	84	53	466	124	74	954	307	103	1380
2022 *	590	300	2988	68	75	100	77	48	493	125	80	978	320	97	1417

** Additionally including data from Austria (coverage 99% in 2020-2021, 98% in 2022, and 100% for all other years), Romania (coverage 97% in 2017, 98% in 2018, 99% in 2019, 95% in 2020, and 100% in 2021-2022), Serbia (97% in 2017, 90% in 2018-2020, and 95% in 2021-2022), Spain (Community of Madrid) (coverage 95% in 2022), Spain (la Rioja) since 2019, Switzerland (coverage 97% in 2021, 99% in 2022, and 100% in all other years), and the Netherlands (coverage 97% in 2017, and 96% in 2018-2022)*

Table D.3.3
Prevalent counts by age and primary renal disease
prevalent patients on December 31, 2022

	All	0-4	5-9	10-14	15-19
	N	N	N	N	N
CAKUT	1437	80	259	454	644
Glomerulonephritis	531	35	87	136	273
Cystic kidney disease	450	34	70	138	208
Hereditary Nephropathy	318	18	62	101	137
Ischaemic Renal Failure	78	6	14	20	38
HUS	123	4	24	43	52
Metabolic disorders	102	11	10	33	48
Vasculitis	45	0	3	12	30
Miscellaneous	297	23	47	76	151
Missing, unknown	514	33	43	176	262

Abbreviations used: CAKUT: congenital anomalies of the kidney and urinary tract; HUS: haemolytic uraemic syndrome
Including data from all countries, also those with incomplete coverage

Table D.3.4
Prevalence per million age-related population by age and cohort
prevalent patients on December 31

Cohort	All	0-4	5-9	10-14	15-19
	Pmarp	Pmarp	Pmarp	Pmarp	Pmarp
2017	68.7	22.3	47.4	76.8	128.4
2018	69.1	24.0	46.4	76.6	128.5
2019	69.2	22.3	47.1	75.8	129.6
2020	69.9	20.7	46.6	77.1	131.2
2021	70.6	21.1	45.1	79.1	130.3
2022	70.1	18.9	45.5	79.9	127.3
2017 *	62.8	19.8	43.9	69.1	117.4
2018 *	63.0	21.4	42.6	69.5	117.0
2019 *	62.9	19.4	42.5	70.3	116.7
2020 *	63.7	18.0	42.5	71.3	119.2
2021 *	64.0	18.8	40.7	73.3	117.2
2022 *	65.1	18.1	41.6	75.0	118.0

** Additionally including data from Austria (coverage 99% in 2020-2021, 98% in 2022, and 100% for all other years), Romania (coverage 97% in 2017, 98% in 2018, 99% in 2019, 95% in 2020, and 100% in 2021-2022), Serbia (97% in 2017, 90% in 2018-2020, and 95% in 2021-2022), Spain (Community of Madrid) (coverage 95% in 2022), Spain (la Rioja) since 2019, Switzerland (coverage 97% in 2021, 99% in 2022, and 100% in all other years), and the Netherlands (coverage 97% in 2017, and 96% in 2018-2022)*

Table D.3.5
Prevalence per million age-related population by age, treatment modality, and cohort
prevalent patients on December 31

Cohort	All			0-4			5-9			10-14			15-19		
	HD	PD	Tx	HD	PD	Tx	HD	PD	Tx	HD	PD	Tx	HD	PD	Tx
	Pmarp	Pmarp	Pmarp	Pmarp	Pmarp	Pmarp	Pmarp	Pmarp	Pmarp	Pmarp	Pmarp	Pmarp	Pmarp	Pmarp	Pmarp
2017	9.3	5.5	52.7	5.7	8.1	8.2	5.4	3.6	38.1	8.2	4.6	63.0	18.3	5.8	101.2
2018	8.8	5.9	53.3	5.0	9.3	9.4	4.7	3.4	38.0	8.2	5.0	62.5	17.5	6.0	102.0
2019	9.1	5.6	53.6	4.8	8.0	9.3	5.3	3.9	37.5	8.6	5.0	61.6	17.8	5.9	103.9
2020	9.6	5.7	53.9	5.2	7.2	8.2	5.1	3.5	37.7	8.0	5.1	63.0	20.0	7.0	102.8
2021	9.7	5.6	54.6	4.9	7.4	8.7	5.8	3.2	35.6	7.7	5.1	65.8	19.9	7.0	101.8
2022	9.9	5.1	54.8	5.3	5.7	8.0	5.3	3.1	37.0	8.0	4.8	66.6	20.1	6.7	99.6
2017 *	9.2	5.0	47.7	5.1	6.7	7.7	4.9	3.4	35.2	8.6	4.0	55.7	18.3	5.8	90.9
2018 *	9.0	5.3	47.8	4.3	7.9	8.9	4.7	3.3	34.4	8.7	4.5	55.5	18.2	5.5	90.9
2019 *	9.3	5.0	47.9	4.3	6.9	8.1	5.2	3.3	33.8	9.3	4.8	55.8	18.3	5.3	91.5
2020 *	9.8	5.2	48.3	4.4	6.2	7.3	5.1	3.5	33.7	8.5	4.6	57.3	20.5	6.4	91.2
2021 *	9.7	5.3	48.5	4.6	6.6	7.6	5.6	3.5	31.1	7.9	4.7	60.4	19.9	6.7	89.4
2022 *	9.9	5.0	49.9	5.0	5.6	7.4	5.2	3.2	33.1	7.9	5.0	61.7	20.5	6.2	90.7

** Additionally including data from Austria (coverage 99% in 2020-2021, 98% in 2022, and 100% for all other years), Romania (coverage 97% in 2017, 98% in 2018, 99% in 2019, 95% in 2020, and 100% in 2021-2022), Serbia (97% in 2017, 90% in 2018-2020, and 95% in 2021-2022), Spain (Community of Madrid) (coverage 95% in 2022), Spain (la Rioja) since 2019, Switzerland (coverage 97% in 2021, 99% in 2022, and 100% in all other years), and the Netherlands (coverage 97% in 2017, and 96% in 2018-2022)*

Table D.3.6
Prevalence per million age-related population by age and primary renal disease
prevalent patients on December 31, 2022

	All	0-4	5-9	10-14	15-19
	Pmarp	Pmarp	Pmarp	Pmarp	Pmarp
CAKUT	24.0	5.9	17.4	28.6	41.2
Glomerulonephritis	8.9	2.6	5.8	8.6	17.5
Cystic kidney disease	7.5	2.5	4.7	8.7	13.3
Hereditary Nephropathy	5.3	1.3	4.2	6.4	8.8
Ischaemic Renal Failure	1.3	0.4	0.9	1.3	2.4
HUS	2.1	0.3	1.6	2.7	3.3
Metabolic disorders	1.7	0.8	0.7	2.1	3.1
Vasculitis	0.8		0.2	0.8	1.9
Miscellaneous	5.0	1.7	3.2	4.8	9.7
Missing, unknown	8.6	2.4	2.9	11.1	16.8

Abbreviations used: CAKUT: congenital anomalies of the kidney and urinary tract; HUS: haemolytic uraemic syndrome
Including data from all countries, also those with incomplete coverage

Methods

VI Methods

The ERA Registry is located at the Department of Medical Informatics in the Amsterdam University Medical Centres, location Academic Medical Centre in Amsterdam, the Netherlands. This department hosts several clinical registries which share an ISO 9001:2015 certified quality management system with standard operating procedures for data collection, data storage, data analysis, and the production of the annual report. In addition, the clinical registries share an ISO 27001 certified information security management system, including procedures to ensure a lasting level of information security and data protection.

Data collection and preparation

Individual patient data

General Data Protection Regulation (GDPR)

All registries participating with individual patient data in this annual report have signed a Data Transfer Agreement with the AMC Medical Research B.V., where the ERA Registry is located.

Data collection

On an annual basis data sets containing individual patient data are uploaded to the ERA Registry office in Amsterdam. Supported file formats for data delivery include Microsoft Access, Microsoft Excel, SPSS, SAS, and delimited text files. Collaborating registries upload their data via a secure data upload portal.

Variables

The ERA Registry asks national and regional registries for the data shown in Table 1.

Table 1: Variables collected for individual patients

Patient data	Transaction data
Patient identifier	Patient identifier
Country of registry	Date of event
Month and year of birth	Type of event / treatment
Sex	Cause of death
Primary Renal Disease	Treatment centre
Date of first KRT	Source registry
	Destination registry

Collaborating registries and participating countries

Individual patient data from 34 national and regional registries in 17 countries were used to create the tables in Section B of this annual report.

Registry data are subject to continuous alteration and improvement. Small differences between ERA Registry data and the data presented in the national and regional registry's own annual reports may result from different times of data extraction.

The data on France presented in Section B of this report, are based on one dataset containing data from 17 of 18 regions, including Auvergne-Rhône-Alpes, Nouvelle-Aquitaine, Bourgogne- Franche-Comté, Bretagne, Centre-Val de Loire, Corse, Grand Est, Hauts-de-France, Île-de-France, Normandie, Occitanie, Pays de la Loire, Provence-Alpes-Côte d'Azur, and the overseas departments of French Guiana, Martinique, Mayotte and Réunion.

Differences between collaborating registries

Definitions and collection of data

In the comparison of data between registries it should be recognized that there may be small differences between registries in definitions and in the collection of their data. For example, the different registries do not collect data at the same level of detail, especially with regard to the various subtypes of treatment modalities.

Patient population

The data from Belgium (Dutch-speaking), Belgium (French-speaking), Spain (Cantabria), Spain (Castile and León), Spain (Castile-La Mancha) and Spain (Navarre) is comprised of patients older than 20 years of age. It has been estimated that the inclusion of data on children would add approximately 2 per million population (Pmp) to the incidence rate and about 13 Pmp to the prevalence.

For the calculation of the incidence of those alive and on KRT on day 91, registries were asked to provide data with follow-up until 31st March 2022. For the following registries data were only available until 31st December 2022: Estonia, France, Montenegro, Spain (Castile and León), Spain (Catalonia), Switzerland, United Kingdom (England/Northern Ireland/Wales) and United Kingdom (Scotland). For these registries the incidence on day 91 was estimated as explained in the paragraph on statistical analyses.

Data loading and cleaning

The data from the registries were imported using an in-house developed import utility. This program first uniforms the data and then translates any non-standard codes. Consistency checks were performed, and thereafter the data were converted to the desired format in order to make the storage of the data in the ERA Registry database possible. The new data, provided by the national and regional registries, replaced all existing data in the database. In this way changes in the data sets made by the national and regional registries were also adopted by the ERA Registry.

After data analysis, the results were checked by the national and regional registries. In the case of discrepancies potential problems regarding (the interpretation of) the data set were solved. Thereafter, more detailed reports were produced which needed approval from the registry representative before publication in this ERA Registry Annual Report.

ERA Registry database

Microsoft SQL Server (a database management system) was used to manage the ERA Registry database. This relational and event-driven database consists of a patient and a transaction table which can be extended according to future needs.

General population data

Midyear population data of the contributing countries were provided by Eurostat [1] or the national bureau of statistics. The sources of the population data used for the analyses in Section B are shown in Table 2. For countries and regions where the coverage of the general population was lower than 100%, population data were adjusted for this lower coverage where applicable.

Reference population

In this year's Annual Report, the age and sex distribution of the EU27 of 2022 as provided by Eurostat [1] was used for the adjustment of the incidence and prevalence for age and sex. The age and sex distribution of EU27 in 2022 is shown in Table 3.

Table 2: Sources of the general population data for the national and regional registries participating with individual patient data in Section B

Registry	Sources of general population data of 2022
Austria	www.statistik.at
Belgium, Dutch-speaking	www.ec.europa.eu/eurostat
Belgium, French-speaking	www.ec.europa.eu/eurostat
Bosnia and Herzegovina	www.popis2013.ba
Denmark	www.ec.europa.eu/eurostat
Greenland	www.stat.gl
Estonia	www.ec.europa.eu/eurostat
France	www.ec.europa.eu/eurostat
Greece	www.ec.europa.eu/eurostat
Iceland	www.ec.europa.eu/eurostat
Montenegro	www.ec.europa.eu/eurostat
Norway	www.ec.europa.eu/eurostat
Romania	www.ec.europa.eu/eurostat
Serbia	www.ec.europa.eu/eurostat
Spain, Andalusia	www.ine.es
Spain, Aragon	www.ine.es
Spain, Asturias	www.ine.es
Spain, Basque country	www.ine.es
Spain, Canary Islands	www.ine.es
Spain, Cantabria	www.ine.es
Spain, Castile and León	www.ine.es
Spain, Castile-La Mancha	www.ine.es
Spain, Catalonia	www.ine.es
Spain, Community of Madrid	www.ine.es
Spain, Extremadura	www.ine.es
Spain, Galicia	www.ine.es
Spain, La Rioja	www.ine.es
Spain, Murcia	www.ine.es
Spain, Navarre	www.ine.es
Spain, Valencian Region	www.ine.es
Sweden	www.ec.europa.eu/eurostat
Switzerland	www.ec.europa.eu/eurostat
the Netherlands	www.ec.europa.eu/eurostat
United Kingdom, England/Northern Ireland/Wales	www.ons.gov.uk
United Kingdom, Scotland	www.ons.gov.uk

Table 3: The age and sex distribution of the reference population EU27 (2022)

Age groups	All	Male	Female
	N	N	N
0-4	20.834.819	10.687.875	10.146.944
5-9	22.411.109	11.503.817	10.907.292
10-14	23.673.861	12.159.723	11.514.138
15-19	23.220.320	11.953.413	11.266.907
20-24	23.900.313	12.337.593	11.562.720
25-29	25.335.811	12.953.454	12.382.357
30-34	28.134.334	14.271.548	13.862.786
35-39	29.110.270	14.644.455	14.465.815
40-44	30.646.596	15.366.935	15.279.661
45-49	31.391.394	15.711.728	15.679.666
50-54	32.409.024	16.137.114	16.271.910
55-59	31.794.915	15.675.743	16.119.172
60-64	29.530.602	14.253.247	15.277.355
65-69	26.569.096	12.463.233	14.105.863
70-74	23.458.925	10.695.632	12.763.293
75-79	17.210.908	7.518.978	9.691.930
80-84	13.874.309	5.619.574	8.254.735
85+	13.228.685	4.390.932	8.837.753
Total	446.735.291	218.344.994	228.390.297

Aggregated data

Data collection

Section C relates to national and regional registries providing the ERA Registry with aggregated data. Data were provided via a Microsoft Excel template and imported for inclusion in this annual report.

Collaborating registries

Aggregated data from 19 national and regional registries were used for the preparation of the tables in Section C. The following countries participated in Section C of this year's annual report: Belarus, Croatia, Cyprus, Czech Republic, Finland, Hungary, Israel, Italy, Kosovo*, Latvia, Lithuania, North Macedonia, Poland, Portugal, Slovakia, Spain, Tunisia, Turkey and Ukraine.

Data from Italy are based on the regions Emilia-Romagna, Liguria, Marche, Piedmont, Puglia, Sicily, Veneto, and Valle d'Aosta.

Data loading and cleaning

The Microsoft Excel template used for data collection includes automated consistency checks. Additional checks were performed manually and data were compared with previous reports. In case of inconsistencies potential problems regarding (the interpretation of) the data set were solved together with the collaborating registry.

General population data

The population data needed for the calculation of incidence and prevalence Pmp were reported by the collaborating registries. If the coverage of the general population in a country by the registry was lower than 100%, population counts were adjusted for this where applicable.

Reference population

As in Section B, we used the age and sex distribution of the EU27 of 2022 (see Table 3) as provided by Eurostat [1] for the adjustment of incidence rates and prevalence.

Coding systems

Primary renal disease

Primary renal diseases (PRD) were defined according to both the old (Appendix 1, from 1995) and new (Appendix 2, from 2018) ERA PRD codes and subsequently categorized into groups. Tables showing the old ERA PRD categorization were based on countries that provided either the old or new ERA PRD codes (note that the new ERA PRD codes were assigned to the old ERA PRD groups resulting in a slightly different distribution of PRD groups). Tables showing the new ERA PRD categorization were based only on countries that provided the new ERA PRD codes. Because the new ERA PRD codes were not available for prevalent patients who started KRT before the introduction of the new ERA PRD coding, tables showing the distribution of PRDs according to the new ERA PRD categorization were presented for incident patients only.

Causes of death

Causes of death were defined according to the ERA coding systems and subsequently classified into groups (Appendix 3).

Event type codes

Appendix 4 provides an overview of the coding currently used for event type codes in the ERA Registry database.

Statistical analyses

An overview of the renal registries contributing data for the different types of analyses is given in Appendix 5. For the data analyses SAS 9.4 [2] was used. This statistical software package is able to maintain large datasets, is syntax driven which increases reproducibility of results, and has extensive capabilities with regard to statistics and data management.

** This designation is without prejudice to positions on status, and is in line with UNSCR 1244/99 and the ICJ Opinion on the Kosovo declaration of independence*

Summary section

The tables and figures in the summary section are based on data both from registries providing individual patient data and registries providing aggregated data. Summary statistics were calculated for all registries providing individual patient data, for all registries providing aggregated data and for all registries combined. Where possible these summary statistics were calculated as the sum of the individual values. In all other cases summary statistics were calculated as weighted averages. The weights were dependent on the denominator (i.e. the population size for Pmp values and the total incident count or prevalent count for percentages) and were calculated as the contribution of the registry to the total of the registries.

Individual patient data

Incidence and prevalence

Methods for calculating incidence and prevalence

The incidence and the prevalence of those alive and on KRT were calculated for the total population and for subgroups based on sex, age group, treatment modality, and primary renal disease. The incidence or prevalence Pmp is the observed incident or prevalent count divided by the general population in that year and multiplied by one million. For the incidence or prevalence per million age- related population (Pmarp) the observed incident or prevalent count per age category was divided by the general population in that age category and multiplied by one million.

For those registries that had no data available with a follow-up until March 31st, 2023, the incidence of KRT on day 91 was estimated. We assumed that the relative difference between the incidence on day 1 and the incidence on day 91 was similar for patients who started KRT in the first nine months of 2022, when compared to patients who started KRT in the last three months of 2022.

For the calculation of the mean and median age for incident patients on day 91, we used the actual age on day 91.

Adjustment of incidence and prevalence

Differences in the unadjusted incidence and prevalence across countries might be due to the differences in the age and sex distribution of the general population. The EU27 age and sex distribution of the European population (Table 3) was therefore used to adjust incidence and prevalence for age and sex [1]. Adjusted rates were derived by applying the weights of the reference population to the observed variable-specific rates (e.g. incidence rate per age group) in a country. This weighted average provides a single summary rate for each country that would be expected if that country had the age and sex distribution of the reference population. The following example shows how to calculate the age-adjusted incidence rate. In this example the incidence rate of country A is 269 Pmp, the age-specific rates of country A and the reference population distribution with respect to age are shown in Table 4.

Table 4: Calculation of the adjusted incidence rate

Age groups	Incidence rate country A	Reference population
	Pmp	%
0-19	332	31.7
20-44	261	39.3
45-64	64	29.0
Total	269	100

The adjusted incident rate of country A is: (0.317 * 332) + (0.393 * 261) + (0.29 * 64) = 226 Pmp

Performed kidney transplants

For all participating countries and regions the number of kidney transplants performed during the year was presented by donor type. In Iceland the number of transplants was somewhat lower, because several patients received transplants in other countries. Rates are expressed as numbers, percentages and Pmp.

Survival probability

In this annual report, patient survival on KRT, patient survival on dialysis, patient survival after the first transplant, and graft survival after the first transplant are presented in tables by age, sex and primary renal disease. In addition, patient survival is presented in figures by primary renal disease and by treatment modality. Survival probabilities are presented as percentages from 0 to 100.

For this report the survival analyses were based on data from registries that provided individual patients data for a sufficiently long follow-up period, including Austria, Belgium (Dutch-speaking), Belgium (French-speaking), Bosnia and Herzegovina, Denmark, Estonia, France, Greece, Iceland, Norway, Spain (Andalusia), Spain (Aragon), Spain (Asturias), Spain (Basque country), Spain (Canary Islands), Spain (Cantabria), Spain (Castile and León), Spain (Castile-La Mancha), Spain (Catalonia), Spain (Community of Madrid), Spain (Extremadura), Spain (Galicia), Spain (Murcia), Spain (Navarre), Spain (Valencian region), Sweden, the Netherlands, United Kingdom (England/Northern Ireland/Wales) and United Kingdom (Scotland). The differences between the survival probabilities in this annual report and survival probabilities published in earlier annual reports of the ERA Registry may be partly due to differences in participating countries.

Survival was analysed from day 1 and from day 91 onwards for those patients alive and on KRT on those days. For the analysis of survival data two five-year cohorts were used:

- 2013-2017 - for the presentation of 90-day, one-, two- and five-year survival probabilities.
- 2016-2020 - for the presentation of 90-day, one- and two-year survival probabilities.

31st December 2022 was applied as censoring date for all survival analyses. For the different types of survival analysis the events and reasons for censoring were defined as shown in Table 5.

Table 5: Overview of the events and reasons for censoring as defined for the survival analyses in Section B

Survival type	Event	Censoring
Patients on kidney replacement therapy	Death of patient	Recovery of renal function *
		Loss to follow-up
		End of follow-up time
Patients on dialysis		
1)	Death of patient	Transplantation
		Recovery of renal function *
		Loss to follow-up
		End of follow-up time
2)	Death of patient	Recovery of renal function *
		Loss to follow-up
		End of follow-up time
First transplant recipients	Death of patient	Loss to follow-up
		End of follow-up time
First graft	Death of patient	Loss to follow-up
	Graft failure	End of follow-up time
	Re-transplantation	

** This was only considered as a censored observation when a patient's renal function had recovered for a period of more than 30 days.*

Unadjusted survival probabilities

Unadjusted survival probabilities were calculated using the Kaplan-Meier method.

Adjusted survival probabilities

The Cox regression model was used to calculate survival probabilities while accounting for confounders [3]. Survival probabilities were adjusted for age, sex and primary renal disease. As the KRT population changes over time, the survival models are adjusted for fixed values for age, sex and PRD to make the results comparable across time periods. The fixed values of variables used for the survival tables are shown in Table 6.

Patients for whom age, sex, or primary renal disease was missing were excluded.

Table 6: Overview of the variables used to adjust the survival probabilities in Section B

Survival type	Age	Sex	Renal disease
Patients on kidney replacement therapy	67 years	63% male	24% Diabetes<br>19% Hypertension / renal vascular disease<br>11% Glomerulonephritis<br>46% Other causes
Patients on dialysis	67 years	63% male	24% Diabetes<br>19% Hypertension / renal vascular disease<br>11% Glomerulonephritis<br>46% Other causes
First transplant recipients (deceased donor)	50 years	63% male	14% Diabetes<br>10% Hypertension / renal vascular disease<br>23% Glomerulonephritis<br>53% Other causes
First transplant recipients (living donor)	50 years	63% male	14% Diabetes<br>10% Hypertension / renal vascular disease<br>23% Glomerulonephritis<br>53% Other causes

Confidence intervals

When survival probabilities were calculated using the Kaplan-Meier method or Cox regression method, the corresponding confidence intervals were calculated according to Bie et al. [4], using the standard error.

Expected remaining lifetimes

Expected remaining lifetimes represent the average number of years of life remaining for those who have reached a given age. In this annual report, expected remaining lifetimes were calculated for patients receiving KRT and for the general population. For the calculation of expected remaining lifetimes of both groups, we adopted the methodology used by the United States Renal Data System (USRDS)[5].

In order to calculate expected remaining lifetimes we used data from registries providing complete follow-up data for the period from 2018 to 2022. The data of the following national or regional registries from 12 countries were included for this analysis: Austria, Belgium (Dutch- speaking), Belgium (French- speaking), Bosnia and Herzegovina, Denmark, Estonia, France, Greece, Iceland, Norway, Spain (Andalusia), Spain (Aragon), Spain (Asturias), Spain (Basque Country), Spain (Canary Islands), Spain (Cantabria), Spain (Castile and León), Spain (Castile-La Mancha), Spain (Catalonia), Spain (Community of Madrid), Spain (Extremadura), Spain (Galicia), Spain (Murcia), Spain (Navarre), Spain (Valencian region), Sweden, the Netherlands, United Kingdom (England/Northern Ireland/Wales), and United Kingdom (Scotland). With regard to the inclusion of the data, it should be noted that the differences between the expected remaining lifetimes of this annual report and expected remaining lifetimes published in earlier annual reports of the ERA Registry may be partly due to differences in participating countries.

KRT population

The expected remaining lifetimes for KRT patients are presented by age, sex and treatment modality. The expected remaining lifetime for a certain patient group is the average of the remaining life expectancies for the patients in that group. Although this cannot be known until all the patients in the group have died, the expected remaining lifetime can be projected by assuming that patients in the patient group will die at the same rates as those observed among older groups of recent prevalent KRT patients.

General population

The number of deaths Pmp by sex and age of the general population of the contributing countries were provided by Eurostat [1]. The size of the contribution of each country to the general population that was used to calculate expected remaining lifetimes was in proportion to the size of the general population covered by its renal registry.

Aggregated data

Incidence and prevalence counts were provided by the contributing registries. To increase the consistency of the data, general explanations of how to complete the Microsoft Excel template and how the adjusted incidence and prevalence were calculated were provided to support the participating registries.

Paediatric Section

The incidence and prevalence tables in Section D of this annual report were based on data from 27 national or regional registries from 15 countries that provided complete individual patient data in their paediatric population for 2017 to 2022, including Austria, Bosnia and Herzegovina, Denmark, Estonia, France, Greece, Iceland, Norway, Romania, Serbia, Spain (Andalusia), Spain (Aragon), Spain (Asturias), Spain (Basque country), Spain (Canary Islands), Spain (Catalonia), Spain (Community of Madrid), Spain (Extremadura), Spain (Galicia), Spain (La Rioja), Spain (Murcia), Spain (Valencian Region), Sweden, Switzerland, the Netherlands, United Kingdom (England/Northern Ireland/Wales) and United Kingdom (Scotland). Since not all countries provided data or had full coverage over the entire period, and to allow for comparison across years, data are shown both including and excluding the data from these countries. The years for which data was available and/or the coverage are indicated below the tables.

Furthermore, as not every region in Spain had a specialised paediatric centre, paediatric patients could be treated in other regions than their resident region. This would result in an underestimation of the incidence and prevalence in these regions. To allow for this, non-resident paediatric patients were not excluded from the paediatric tables.

Incidence (on day 1) and prevalence on KRT were calculated for subgroups based on age, treatment modality, and primary renal disease. Incidence on KRT by 2-year cohorts was presented for paediatric patients who started KRT between 2017 and 2022. The incidence rate on KRT (Pmarp) was the observed incident count in that particular time period divided by the age-related general population in that time period (for example the total population in 2019 plus the total population in 2020) multiplied by one million. The grouping of primary renal disease codes for the paediatric population is described in Table 7 as it differs from the grouping in the adult population.

Table 7: Grouping of primary renal diseases for paediatric patients in Section D
Groups and codes included

**Primary renal disease group**	**Primary renal disease code** (see appendix 1)
Congenital anomalies of the kidney and urinary tract (CAKUT)	20, 21, 22, 23, 24, 25, 29, 60, 61, 63, and 66
Glomerulonephritis	10, 11, 12, 13, 14, 15, 16, 17, 19, and 86
Cystic kidney disease	40, 41, 42, 43, and 49
Hereditary nephropathy	50, 51, and 59
Ischaemic renal failure	90
Haemolytic uraemic syndrome (HUS)	88
Metabolic disorders	52, 53, 54, 92, and 93
Vasculitis	73, 74, 84, 85, and 87
Miscellaneous	30, 31, 32, 33, 34, 39, 70, 71, 72, 75, 76, 78, 79, 80, 81, 82, 83, 89, 91, 94, 95, 96, and 99
Missing, unknown	missing and 00

The prevalence on KRT was presented on 31st December of each year from 2017 to 2022. The prevalence on KRT by primary renal disease was calculated for patients prevalent on 31st December, 2022.

Bibliography

1) Eurostat: www.ec.europa.eu/eurostat/data/database.
2) SAS Institute Inc., Cary, NC, USA.
3) Altman, D. Practical statistics for medical research. second ed. 1999, London: Chapman & Hall.
4) Bie O, Borgan O and Liestol K. Confidence intervals and confidence bands for the cumulative hazard rate function and their small sample properties. Scandinavian Journal of Statistics 1987; 14: 221-233.
5) U.S. Renal Data System, USRDS 2012 Annual Data Report: Atlas of End-Stage Renal Disease in the United States, National Institutes of Health, National Institute of Diabetes and Digestive and Kidney Diseases, Bethesda, MD, 2012.

Appendices

Appendix 1 - Grouping of primary renal disease codes 1995

1994 code	1995 code	PRD group	Primary renal disease
10	10	I	Glomerulonephritis; histologically NOT examined
11	11	I	Focal segmental glomerulosclerosis with nephrotic syndrome in children
12	12	I	IgA nephropathy (proven by immunofluorescence, not code 76 or 85)
13	13	I	Dense deposit disease; membrano-proliferative GN; type II (proven by immunofluorescence / electron microscopy)
14	14	I	Membranous nephropathy
15	15	I	Membrano-proliferative GN; type I (proven by immunofluorescence / electron microscopy, not code 84 or 89)
16	16	I	Crescentic (extracapillary) glomerulonephritis (type I, II, III)
17	17	I	Focal segmental glomerulosclerosis with nephrotic syndrome in adults
19	19	I	Glomerulonephritis; histologically examined, not given above
20	20	II	Pyelonephritis; cause not specified
21	21	II	Pyelonephritis associated with neurogenic bladder
22	22	II	Pyelonephritis due to congenital obstructive uropathy with/without vesico-ureteric reflux
23	23	II	Pyelonephritis due to acquired obstructive uropathy
24	24	II	Pyelonephritis due to vesico-ureteric reflux without obstruction
25	25	II	Pyelonephritis due to urolithiasis
29	29	II	Pyelonephritis due to other cause
30	30	VII	Interstitial nephritis (not pyelonephritis) due to other cause, or unspecified (not mentioned above)
31	31	VII	Nephropathy (interstitial) due to analgesic drugs
32	32	VII	Nephropathy (interstitial) due to cis-platinum
33	33	VII	Nephropathy (interstitial) due to cyclosporin A
	34	VII	Lead induced nephropathy (interstitial)
39	39	VII	Drug induced nephropathy (interstitial) not mentioned above
40	40	VII	Cystic kidney disease - type unspecified
41	41	III	Polycystic kidneys; adult type (dominant)
42	42	VII	Polycystic kidneys; infantile (recessive)
43	43	VII	Medullary cystic disease; including nephronophthisis
49	49	VII	Cystic kidney disease - other specified type
50	50	VII	Hereditary / Familial nephropathy - type unspecified
51	51	VII	Hereditary nephritis with nerve deafness (Alport's Syndrome)
52	52	VII	Cystinosis
53	53	VII	Primary oxalosis
54	54	VII	Fabry's disease
59	59	VII	Hereditary nephropathy - other specified type
60	60	VII	Renal hypoplasia (congenital) - type unspecified
61	61	VII	Oligomeganephronic hypoplasia
63	63	VII	Congenital renal dysplasia with or without urinary tract malformation
66	66	VII	Syndrome of agenesis of abdominal muscles (Prune Belly)
70	70	V	Renal vascular disease - type unspecified
71	71	IV	Renal vascular disease due to malignant hypertension
72	72	IV	Renal vascular disease due to hypertension
73	73	VII	Renal vascular disease due to polyarteritis
74	74	VII	Wegener's granulomatosis
	75	VII	Ischaemic renal disease/cholesterol embolism (1998 prd code)
76	76	VII	Glomerulonephritis related to liver cirrhosis
	78	VII	Cryoglobulinaemic glomerulonephritis
79	79	V	Renal vascular disease - due to other cause (not given above and not code 84-88)
80	80	VI	Diabetes glomerulosclerosis or diabetic nephropathy - Type I
81	80	VI	Diabetes glomerulosclerosis or diabetic nephropathy - Type II
82	82	VII	Myelomatosis / light chain deposit disease
83	83	VII	Amyloid
84	84	VII	Lupus erythematosus
85	85	VII	Henoch-Schoenlein purpura
86	86	VII	Goodpasture's Syndrome
87	87	VII	Systemic sclerosis (scleroderma)
88	88	VII	Haemolytic Uraemic Syndrome (including Moschcowitz Syndrome)
89	89	VII	Multi-system disease - other (not mentioned above)
90	90	VII	Tubular necrosis (irreversible) or cortical necrosis (different from 88)
91	91	VII	Tuberculosis
92	92	VII	Gout
93	93	VII	Nephrocalcinosis and hypercalcaemic nephropathy
94	94	VII	Balkan nephropathy
95	95	VII	Kidney tumour
96	96	VII	Traumatic or surgical loss of kidney
99	99	VII	Other identified renal disorders
00	00	VIII	Chronic renal failure; aetiology uncertain

PRD group: I: glomerulonephritis / sclerosis; II: pyelonephritis; III: polycystic kidneys, adult type; IV: hypertension; V: renal vascular disease; VI: diabetes; VII: miscellaneous; VIII: unknown

Appendix 2 - Grouping of primary renal disease codes 2018

I: GLOMERULAR DISEASE

Code	Primary renal disease
1003	Adult nephrotic syndrome - no histology
1472	Anti-Glomerular basement membrane (GBM) disease / Goodpasture's syndrome - histologically proven
1464	Anti-Glomerular basement membrane (GBM) disease / Goodpasture's syndrome - no histology
1440	Churg-Strauss syndrome - histologically proven
1438	Churg-Strauss syndrome - no histology
1088	Congenital nephrotic syndrome (CNS) - congenital infection
1057	Congenital nephrotic syndrome (CNS) - diffuse mesangial sclerosis
1042	Congenital nephrotic syndrome (CNS) - Finnish type - histologically proven
1035	Congenital nephrotic syndrome (CNS) - Finnish type - no histology
1061	Congenital nephrotic syndrome (CNS) - focal segmental glomerulosclerosis (FSGS)
1026	Congenital nephrotic syndrome (CNS) - no histology
1570	Cryoglobulinaemia secondary to hepatitis C - histologically proven
1562	Cryoglobulinaemia secondary to hepatitis C - no histology
1591	Cryoglobulinaemia secondary to systemic disease - histologically proven
1589	Cryoglobulinaemia secondary to systemic disease - no histology
1074	Denys-Drash syndrome
1331	Diffuse endocapillary glomerulonephritis
1558	Essential mixed cryoglobulinaemia - histologically proven
1543	Essential mixed cryoglobulinaemia - no histology
1308	Familial focal segmental glomerulosclerosis (FSGS) - autosomal dominant - histologically proven
1298	Familial focal segmental glomerulosclerosis (FSGS) - autosomal dominant - no histology
1280	Familial focal segmental glomerulosclerosis (FSGS) - autosomal recessive - histologically proven
1279	Familial focal segmental glomerulosclerosis (FSGS) - autosomal recessive - no histology
1144	Familial IgA nephropathy - histologically proven
1137	Familial IgA nephropathy - no histology
1354	Focal and segmental proliferative glomerulonephritis
3754	Focal segmental glomerulosclerosis (FSGS) secondary to HIV
3765	Focal segmental glomerulosclerosis (FSGS) secondary to lithium
1320	Focal segmental glomerulosclerosis (FSGS) secondary to obesity - histologically proven
1312	Focal segmental glomerulosclerosis (FSGS) secondary to obesity - no histology
3777	Focal segmental glomerulosclerosis (FSGS) secondary to sickle cell
1377	Glomerulonephritis - histologically indeterminate
3749	Glomerulonephritis - no histology
1365	Glomerulonephritis - secondary to systemic disease
1417	Granulomatosis with polyangiitis - histologically proven
1401	Granulomatosis with polyangiitis - no histology
1515	Henoch-Schönlein purpura / nephritis - histologically proven
1504	Henoch-Schönlein purpura / nephritis - no histology
1251	Idiopathic rapidly progressive (crescentic) glomerulonephritis
1128	IgA nephropathy - histologically proven
1116	IgA nephropathy - no histology
1163	IgA nephropathy secondary to liver cirrhosis - histologically proven
1159	IgA nephropathy secondary to liver cirrhosis - no histology
1171	IgM - associated nephropathy
1205	Membranous nephropathy - drug induced
1185	Membranous nephropathy - idiopathic
1214	Membranous nephropathy - infection associated
1192	Membranous nephropathy - malignancy associated
1349	Mesangial proliferative glomerulonephritis
1222	Mesangiocapillary glomerulonephritis type 1
1233	Mesangiocapillary glomerulonephritis type 2 (dense deposit disease)
1246	Mesangiocapillary glomerulonephritis type 3
1429	Microscopic polyangiitis - histologically proven
1100	Minimal change nephropathy - histologically proven
1090	Minimal change nephropathy - no histology
3615	Nephrotic syndrome of childhood - no trial of steroids - no histology
3604	Nephrotic syndrome of childhood - steroid resistant - no histology
1019	Nephrotic syndrome of childhood - steroid sensitive - no histology
1455	Polyarteritis nodosa
1267	Primary focal segmental glomerulosclerosis (FSGS)
1536	Renal scleroderma / systemic sclerosis - histologically proven
1527	Renal scleroderma / systemic sclerosis - no histology
1493	Systemic lupus erythematosus / nephritis - histologically proven
1486	Systemic lupus erythematosus / nephritis - no histology
1383	Systemic vasculitis - ANCA negative - histologically proven
3847	Systemic vasculitis - ANCA negative - no histology
3852	Systemic vasculitis - ANCA positive - histologically proven
1396	Systemic vasculitis - ANCA positive - no histology

II: TUBULOINTERSTITIAL DISEASE

Code	Primary renal disease
1768	Acquired obstructive nephropathy due to neurogenic bladder
1752	Acquired obstructive uropathy / nephropathy
2196	Acute urate nephropathy - histologically proven
2183	Acute urate nephropathy - no histology
1995	Aristolochic acid nephropathy (Balkan / Chinese herb / endemic nephropathy) - histologically proven
1982	Aristolochic acid nephropathy (Balkan / Chinese herb / endemic nephropathy) - no histology
1710	Bladder exstrophy
1845	Calcium oxalate urolithiasis
1832	Calculus nephropathy / urolithiasis
2203	Chronic urate nephropathy - histologically proven
3636	Chronic urate nephropathy - no histology
1625	Congenital dysplasia / hypoplasia
1706	Congenital neurogenic bladder
1660	Congenital pelvi-ureteric junction obstruction
1673	Congenital vesico-ureteric junction obstruction
2005	Drug-induced tubulointerstitial nephritis - no histology
2014	Drug-induced tubulointerstitial nephritis - histologically proven
1850	Enteric hyperoxaluria
1911	Familial interstitial nephropathy - histologically proven
1907	Familial interstitial nephropathy - no histology
1618	Familial reflux nephropathy
1656	Glomerulocystic disease
2257	Hantavirus nephropathy
3662	Hypercalcaemic nephropathy
1813	Idiopathic retroperitoneal fibrosis
2177	Lead induced nephropathy - histologically proven
2165	Lead induced nephropathy - no histology
2242	Leptospirosis
1866	Magnesium ammonium phosphate (struvite) urolithiasis
1723	Megacystis-megaureter
1639	Multicystic dysplastic kidneys
2098	Nephropathy due to aminoglycosides - histologically proven
2080	Nephropathy due to aminoglycosides - no histology
2112	Nephropathy due to amphotericin - histologically proven
2108	Nephropathy due to amphotericin - no histology
2033	Nephropathy due to analgesic drugs - histologically proven
2022	Nephropathy due to analgesic drugs - no histology
2051	Nephropathy due to ciclosporin - histologically proven
2046	Nephropathy due to ciclosporin - no histology
2131	Nephropathy due to cisplatin - histologically proven
2120	Nephropathy due to cisplatin - no histology
2154	Nephropathy due to lithium - histologically proven
2149	Nephropathy due to lithium - no histology
2079	Nephropathy due to tacrolimus - histologically proven
2067	Nephropathy due to tacrolimus - no histology
2288	Nephropathy related to HIV - histologically proven
2274	Nephropathy related to HIV - no histology
1799	Obstructive nephropathy due to bladder cancer
1809	Obstructive nephropathy due to other malignancies
1781	Obstructive nephropathy due to prostate cancer
1775	Obstructive nephropathy due to prostatic hypertrophy
1734	Oligomeganephronia
2300	Other specific infection
1687	Posterior urethral valves
1602	Primary reflux nephropathy - sporadic
2219	Radiation nephritis
2226	Renal / perinephric abscess
3627	Renal cysts and diabetes syndrome
1641	Renal dysplasia due to fetal ACE-inhibitor exposure
1747	Renal papillary necrosis
3796	Renal papillary necrosis caused by analgesics
3783	Renal papillary necrosis caused by diabetes
3806	Renal papillary necrosis caused by sickle cell
1976	Renal sarcoidosis - histologically proven
1969	Renal sarcoidosis - no histology

II: TUBULOINTERSTITIAL DISEASE (continued)

Code	Primary renal disease
2235	Renal tuberculosis
3689	Retroperitoneal fibrosis secondary to drugs
1821	Retroperitoneal fibrosis secondary to malignancies
3670	Retroperitoneal fibrosis secondary to peri-aortitis
2290	Schistosomiasis
1694	Syndrome of agenesis of abdominal muscles - prune belly syndrome
1897	Tubulointerstitial nephritis - histologically proven
1884	Tubulointerstitial nephritis - no histology
1930	Tubulointerstitial nephritis associated with autoimmune disease - histologically proven
1924	Tubulointerstitial nephritis associated with autoimmune disease - no histology
1953	Tubulointerstitial nephritis with uveitis (TINU) - histologically proven
1948	Tubulointerstitial nephritis with uveitis (TINU) - no histology
1878	Uric acid urolithiasis
2261	Xanthogranulomatous pyelonephritis

III: DIABETES MELLITUS

Code	Primary renal disease
2328	Diabetic nephropathy in type I diabetes - histologically proven
2316	Diabetic nephropathy in type I diabetes - no histology
2344	Diabetic nephropathy in type II diabetes - histologically proven
2337	Diabetic nephropathy in type II diabetes - no histology

IV: HYPERTENSION / RENAL VASCULAR DISEASE

Code	Primary renal disease
2392	Ageing kidney - no histology
2448	Atheroembolic renal disease - histologically proven
2430	Atheroembolic renal disease - no histology
2363	Chronic hypertensive nephropathy - histologically proven
2359	Chronic hypertensive nephropathy - no histology
2453	Fibromuscular dysplasia of renal artery
2407	Ischaemic nephropathy - no histology
2411	Ischaemic nephropathy / microvascular disease - histologically proven
2385	Malignant hypertensive nephropathy / accelerated hypertensive nephropathy - histologically proven
2371	Malignant hypertensive nephropathy / accelerated hypertensive nephropathy - no histology
2469	Renal arterial thrombosis / occlusion
2424	Renal artery stenosis

V: OTHER SYSTEMIC DISEASES AFFECTING THE KIDNEY

Code	Primary renal disease
2513	AA amyloid secondary to chronic inflammation
2521	AL amyloid secondary to plasma cell dyscrasia
2623	Atypical haemolytic uraemic syndrome (HUS) - diarrhoea negative
2482	Cardiorenal syndrome
2652	Congenital haemolytic uraemic syndrome (HUS)
2566	Familial AA amyloid secondary to familial Mediterranean fever / TRAPS (Hibernian fever) - histologically proven
2550	Familial AA amyloid secondary to familial Mediterranean fever / TRAPS (Hibernian fever) - no histology
2545	Familial amyloid secondary to protein mutations - histologically proven
2532	Familial amyloid secondary to protein mutations - no histology
2668	Familial haemolytic uraemic syndrome (HUS)
2675	Familial thrombotic thrombocytopenic purpura (TTP)
2610	Haemolytic uraemic syndrome (HUS) - diarrhoea associated
2647	Haemolytic uraemic syndrome (HUS) secondary to systemic disease
2495	Hepatorenal syndrome
2606	Immunotactoid / fibrillary nephropathy
2597	Light chain deposition disease
2584	Myeloma cast nephropathy - histologically proven
2578	Myeloma kidney - no histology
2681	Nephropathy due to eclampsia
3834	Nephropathy due to pre-eclampsia
2509	Renal amyloidosis
2476	Renal vein thrombosis
2702	Sickle cell nephropathy - histologically proven
2699	Sickle cell nephropathy - no histology
2634	Thrombotic thrombocytopenic purpura (TTP)

VI: FAMILIAL / HEREDITARY NEPHROPATHIES

Code	Primary renal disease
3071	Alagille syndrome
2760	Alport syndrome - histologically proven
2756	Alport syndrome - no histology
3118	Apparent mineralocorticoid excess
2718	Autosomal dominant (AD) polycystic kidney disease
2725	Autosomal dominant (AD) polycystic kidney disease type I
2739	Autosomal dominant (AD) polycystic kidney disease type II
2741	Autosomal recessive (AR) polycystic kidney disease
3085	Bartter syndrome
2773	Benign familial haematuria
3322	Branchio-oto-renal syndrome
2794	Cystic kidney disease
2964	Cystinosis
2955	Cystinuria
2929	Dent disease
3028	Distal renal tubular acidosis (RTA) - type I
3037	Distal renal tubular acidosis with sensorineural deafness - gene mutations
3230	Fabry disease - histologically proven
3224	Fabry disease - no histology
3173	Familial hypercalciuric hypocalcaemia
3160	Familial hypocalciuric hypercalcaemia
3187	Familial hypomagnesaemia
3379	Familial nephropathy
3314	Frasier syndrome
3092	Gitelman syndrome
3125	Glucocorticoid suppressible hyperaldosteronism
3305	Horse-shoe kidney
2993	Hypophosphataemic rickets autosomal recessive (AR)
2986	Hypophosphataemic rickets X-linked (XL)
3139	Inherited / genetic diabetes mellitus type II
2940	Inherited aminoaciduria
2972	Inherited renal glycosuria
3810	Kidney stones due to ARPT deficiency
3351	Lawrence-Moon-Biedl / Bardet-Biedl syndrome
3059	Lesch Nyhan syndrome - hypoxanthine guanine phosphoribosyl transferase deficiency
3102	Liddle syndrome
2938	Lowe syndrome (oculocerebrorenal syndrome)
2804	Medullary cystic kidney disease type I
2815	Medullary cystic kidney disease type II
3295	Medullary sponge kidneys
3367	Mitochondrial cytopathy
3253	Nail-patella syndrome
3044	Nephrogenic diabetes insipidus
2836	Nephronophthisis
2843	Nephronophthisis - type 1 (juvenile type)
2858	Nephronophthisis - type 2 (infantile type)
2862	Nephronophthisis - type 3 (adolescent type)
2870	Nephronophthisis - type 4 (juvenile type)
2889	Nephronophthisis - type 5
2891	Nephronophthisis - type 6
3063	Phosphoribosyl pyrophosphate synthetase (PRPPS) superactivity
2901	Primary Fanconi syndrome
3194	Primary hyperoxaluria
3207	Primary hyperoxaluria type I
3211	Primary hyperoxaluria type II
3731	Primary hyperoxaluria type III
3000	Primary renal tubular acidosis (RTA)
3016	Proximal renal tubular acidosis (RTA) - type II
3141	Pseudohypoaldosteronism type 1
3156	Pseudohypoaldosteronism type 2 (Gordon syndrome)
3658	Renal coloboma syndrome
3269	Rubinstein-Taybi syndrome
2787	Thin basement membrane disease
3346	Townes-Brocks syndrome
3276	Tuberous sclerosis
2917	Tubular disorder as part of inherited metabolic diseases
2827	Uromodulin-associated nephropathy (familial juvenile hyperuricaemic nephropathy)
3282	Von Hippel-Lindau disease
3333	Williams syndrome
3248	Xanthinuria

VII: MISCELLANEOUS RENAL DISORDERS

Code	Primary renal disease
3442	Acute cortical necrosis
3380	Acute kidney injury
3403	Acute kidney injury due to circulatory failure
3398	Acute kidney injury due to hypovolaemia
3435	Acute kidney injury due to nephrotoxicity
3426	Acute kidney injury due to rhabdomyolysis
3419	Acute kidney injury due to sepsis
3457	Acute pyelonephritis
3564	Chronic kidney disease (CKD) / chronic renal failure (CRF) - aetiology uncertain / unknown - histologically proven
3555	Chronic kidney disease (CKD) / chronic renal failure (CRF) - aetiology uncertain / unknown - no histology
3529	Chronic kidney disease (CKD) / chronic renal failure (CRF) caused by tumour nephrectomy
3540	Chronic kidney disease (CKD) / chronic renal failure (CRF) due to donor nephrectomy
3538	Chronic kidney disease (CKD) / chronic renal failure (CRF) due to traumatic loss of kidney
3708	Chronic renal failure
3643	Chronic renal failure due to systemic infection
3572	Haematuria and proteinuria - no histology
3823	Infiltration by lymphoma - histologically proven
3712	Isolated haematuria - no histology
3720	Isolated proteinuria - no histology
3461	Kidney tumour
3501	Mesoblastic nephroma - histologically proven
3474	Renal cell carcinoma - histologically proven
3691	Renal failure
3517	Single kidney identified in adulthood
3488	Transitional cell carcinoma - histologically proven
3490	Wilms tumour - histologically proven

Appendix 3 - Grouping of causes of death

1994 code	1995 code	COD group	Cause of death
0	0	XI	Cause of death uncertain / not determined
11	11	I	Myocardial ischaemia and infarction
12	12	X	Hyperkalaemia
13	13	X	Haemorrhagic pericarditis
14	14	II	Other causes of cardiac failure
15	15	III	Cardiac arrest / sudden death; other cause or unknown
16	16	II	Hypertensive cardiac failure
17	17	X	Hypokalaemia
18	18	II	Fluid overload / pulmonary oedema
21	21	X	Pulmonary embolus
22	22	IV	Cerebro-vascular accident, other cause or unspecified
23	23	X	Gastro-intestinal haemorrhage
24	24	X	Haemorrhage from graft site
25	25	X	Haemorrhage from vascular access or dialysis circuit
26	26	X	Haemorrhage from ruptured vascular aneurysm (not code 22 or 23)
27	27	X	Haemorrhage from surgery (not code 23, 24 or 26)
28	28	X	Other haemorrhage (not codes 23-27)
29	29	X	Mesenteric infarction
31	31	V	Pulmonary infection (bacterial - not code 73)
32	32	V	Pulmonary infection (viral)
33	33	V	Pulmonary infection (fungal or protozoal; parasitic)
34		V	Infections elsewhere except virus hepatitis
35	35	V	Septicaemia
36	36	V	Tuberculosis (lung)
37	37	V	Tuberculosis (elsewhere)
38	38	V	Generalized viral infection
39	39	V	Peritonitis (all causes except for Peritoneal Dialysis)
41	41	X	Liver disease due to hepatitis B virus
42	42	X	Liver disease due to other viral hepatitis
43	43	X	Liver disease due to drug toxicity
44	44	X	Cirrhosis - not viral
45	45	X	Cystic liver disease
46	46	X	Liver failure - cause unknown
51	51	VI	Patient refused further treatment for ESRD
52	52	VI	Suicide
53	53	VII	ESRD treatment ceased for any other reason
	54	VII	ESRD treatment withdrawn for medical reasons
61		X	Uremia caused by graft failure
62	62	X	Pancreatitis
63	63	X	Bone marrow depression
64	64	VIII	Cachexia
66	66	IX	Malignant disease, possibly induced by immunosuppressive therapy
67	67	IX	Malignant disease: solid tumors except those of 66
	68	IX	Malignant disease: lymphoproliferative disorders except those of 66
69	69	X	Dementia
70	70	V	Peritonitis (sclerosing, with peritoneal dialysis)
71	71	X	Perforation of peptic ulcer
72	72	X	Perforation of colon
73	73	X	Chronic obstructive airways disease
81	81	X	Accident related to ESRD treatment (not code 25)
82	82	X	Accident unrelated to ESRD treatment
	100	V	Peritonitis (bacterial, with peritoneal dialysis)
	101	V	Peritonitis (fungal, with peritoneal dialysis)
	102	V	Peritonitis (due to other cause, with peritoneal dialysis)
99	99	X	Other identified cause of death

COD group: I: myocardial ischaemia and infarction; II: heart failure; III: cardiac arrest, other cause / unknown; IV: cerebrovascular accident; V: infection; VI: suicide / refusal treatment; VII: withdrawal; VIII: cachexia; IX: malignancies; X: miscellaneous; XI: unknown / unavailable

Appendix 4 - Event type codes

Event code	Event
001	Home haemodialysis
002	Centre haemodialysis
003	Haemofiltration
004	Haemodiafiltration
009	Haemodialysis, type unknown
011	Continuous Ambulatory Peritoneal Dialysis (CAPD)
012	Intermittent Ambulatory Peritoneal Dialysis (CAPD)
013	Continuous Automated Peritoneal Dialysis (APD)
014	Intermittent Automated Peritoneal Dialysis (APD)
019	Peritoneal Dialysis, type unknown
090	Unknown dialysis
020	Transplantation, living donor
021	Transplantation, living related donor
022	Transplantation, living unrelated donor
023	Transplantation, deceased donor
029	Transplantation, donor type unknown
060	Transplantation follow-up (still functioning)
061	Graft failure
031	Treatment type unknown / unavailable
051	Recovery Renal Function
052	Death
053	Transfer out to other registry
054	Transfer in from other registry
055	Lost to follow-up
071	Limited care / stopped treatment (without recovery of renal function)

Appendix 5 - Renal registries contributing data for the different types of analyses

	Registries providing individual patient data			Registries providing aggregated data	Registries providing paedriatic data
	Incidence and prevalence	Survival	Expected remaining lifetimes	Incidence and prevalence	Incidence and prevalence
Austria	+	+	+	-	+
Belarus	-	-	-	+	-
Belgium, Dutch-speaking	+	+	+	-	-
Belgium, French-speaking	+	+	+	-	-
Bosnia and Herzegovina	+	+	+	-	+
Croatia	-	-	-	+	-
Cyprus	-	-	-	+	-
Czech Republic	-	-	-	+	-
Denmark	+	+	+	-	+
Estonia	+	+	+	-	+
Finland	-	-	-	+	-
France (17 of 18 regions)	+	+	+	-	+
Greece	+	+	+	-	+
Hungary	-	-	-	+	-
Iceland	+	+	+	-	+
Israel	-	-	-	+	-
Italy (8 of 20 regions)	-	-	-	+	-
Kosovo	-	-	-	+	-
Latvia	-	-	-	+	-
Lithuania	-	-	-	+	-
Montenegro	+	-	-	-	-
North Macedonia	-	-	-	+	-
Norway	+	+	+	-	+
Poland	-	-	-	+	-
Portugal	-	-	-	+	-
Romania	+	-	-	-	+
Serbia	+	-	-	-	+
Slovakia	-	-	-	+	-
Spain, Andalusia	+	+	+	+	+
Spain, Aragon	+	+	+	+	+
Spain, Asturias	+	+	+	+	+
Spain, Balearic Islands	-	-	-	+	-
Spain, Basque country	+	+	+	+	+
Spain, Canary Islands	+	+	+	+	+
Spain, Cantabria	+	+	+	+	-
Spain, Castile and León	+	+	+	+	-
Spain, Castile-La Mancha	+	+	+	+	-
Spain, Catalonia	+	+	+	+	+
Spain, Ceuta	-	-	-	+	-
Spain, Community of Madrid	+	+	+	+	+
Spain, Extremadura	+	+	+	+	+
Spain, Galicia	+	+	+	+	+
Spain, La Rioja	+	-	-	+	+
Spain, Melilla	-	-	-	+	-
Spain, Murcia	+	+	+	+	+
Spain, Navarre	+	+	+	+	-
Spain, Valencian region	+	+	+	+	+
Sweden	+	+	+	-	+
Switzerland	+	-	-	-	+
the Netherlands	+	+	+	-	+
Tunisia, Sfax region	-	-	-	+	-
Turkey	-	-	-	+	-
Ukraine	-	-	-	+	-
United Kingdom, England	+	+	+	-	+
United Kingdom, Northern Ireland	+	+	+	-	+
United Kingdom, Scotland	+	+	+	-	+
United Kingdom, Wales	+	+	+	-	+

ERA Registry
Amsterdam UMC, location AMC
Department of Medical Informatics
PO Box 22700
1100 DE Amsterdam
the Netherlands

August 2024

Amsterdam UMC
University Medical Centers
